# One hundred and three new species of *Trigonopterus* weevils from Sulawesi

**DOI:** 10.3897/zookeys.828.32200

**Published:** 2019-03-07

**Authors:** Alexander Riedel, Raden Pramesa Narakusumo

**Affiliations:** 1 Museum of Natural History Karlsruhe, Erbprinzenstr. 13, D-76133 Karlsruhe, Germany Museum of Natural History Karlsruhe Karlsruhe Germany; 2 Museum Zoologicum Bogoriense, Research Center for Biology, Indonesian Institute of Sciences (LIPI), Jl. Raya Jakarta-Bogor km 46, Cibinong 16911, Indonesia Research Center for Biology, Indonesian Institute of Sciences Jl. Raya Jakarta-Bogor Indonesia

**Keywords:** Celebes, Coleoptera, conservation, *cox1*, Cryptorhynchinae, Curculionidae, DNA barcoding, endemism, hyperdiverse, integrative taxonomy, morphology, Southeast Asia, turbo-taxonomy, Wallacea, weevils

## Abstract

The genus *Trigonopterus* Fauvel, 1862 is highly diverse in Melanesia, the Moluccas, and the Sunda Islands. Only one species, *Trigonopterusfulvicornis* (Pascoe, 1885) was so far recorded from Sulawesi. Based on focused field-work the fauna from Sulawesi and nearby islands is here revised. We redescribe *T.allotopus* Riedel newly recorded for Sulawesi and describe an additional 103 new species: *T.abnormis***sp. n.**, *T.adspersus***sp. n.**, *T.ambangensis***sp. n.**, *T.ampanensis***sp. n.**, *T.analis***sp. n.**, *T.arachnobas***sp. n.**, *T.armipes***sp. n.**, *T.artemis***sp. n.**, *T.asterix***sp. n.**, *T.barbipes***sp. n.**, *T.bonthainensis***sp. n.**, *T.carinirostris***sp. n.**, *T.castaneipennis***sp. n.**, *T.celebensis***sp. n.**, *T.cirripes***sp. n.**, *T.collaris***sp. n.**, *T.costatulus***sp. n.**, *T.curvipes***sp. n.**, *T.crenulatus***sp. n.**, *T.cricki***sp. n.**, *T.darwini***sp. n.**, *T.ejaculatorius***sp. n.**, *T.fuscipes***sp. n.**, *T.gracilipes***sp. n.**, *T.heberti***sp. n.**, *T.hirsutus***sp. n.**, *T.humilis***sp. n.**, *T.hypocrita***sp. n.**, *T.idefix***sp. n.**, *T.impressicollis***sp. n.**, *T.incendium***sp. n.**, *T.incognitus***sp. n.**, *T.indigenus***sp. n.**, *T.inhonestus***sp. n.**, *T.invalidus***sp. n.**, *T.jasminae***sp. n.**, *T.klabatensis***sp. n.**, *T.kolakensis***sp. n.**, *T.kotamobagensis***sp. n.**, *T.laevigatus***sp. n.**, *T.lampros***sp. n.**, *T.latipennis***sp. n.**, *T.lompobattangensis***sp. n.**, *T.luwukensis***sp. n.**, *T.mahawuensis***sp. n.**, *T.manadensis***sp. n.**, *T.mangkutanensis***sp. n.**, *T.matalibaruensis***sp. n.**, *T.mesai***sp. n.**, *T.minahassae***sp. n.**, *T.moatensis***sp. n.**, *T.modoindingensis***sp. n.**, *T.nanus***sp. n.**, *T.nitidulus***sp. n.**, *T.obelix***sp. n.**, *T.ovalipunctatus***sp. n.**, *T.ovatulus***sp. n.**, *T.pagaranganensis***sp. n.**, *T.palopensis***sp. n.**, *T.paracollaris***sp. n.**, *T.pauper***sp. n.**, *T.pendolensis***sp. n.**, *T.posoensis***sp. n.**, *T.prismae***sp. n.**, *T.procurtus***sp. n.**, *T.pseudallotopus***sp. n.**, *T.pseudanalis*, **sp. n.**, *T.pseudovatulus***sp. n.**, *T.pseudovalipunctatus***sp. n.**, *T.pseudofulvicornis***sp. n.**, *T.pseudomanadensis***sp. n.**, *T.pseudosimulans***sp. n.**, *T.pumilus***sp. n.**, *T.rantepao***sp. n.**, *T.reticulatus***sp. n.**, *T.rhombiformis***sp. n.**, *T.rotundatus***sp. n.**, *T.rotundulus***sp. n.**, *T.rudis***sp. n.**, *T.rufipes***sp. n.**, *T.sampunensis***sp. n.**, *T.sampuragensis***sp. n.**, *T.satyrus***sp. n.**, *T.scabripes***sp. n.**, *T.scaphiformis***sp. n.**, *T.scitulus***sp. n.**, *T.selayarensis***sp. n.**, *T.serripes***sp. n.**, *T.seticnemis***sp. n.**, *T.silvicola***sp. n.**, *T.squalidulus***sp. n.**, *T.sulawesiensis***sp. n.**, *T.suturatus***sp. n.**, *T.tatorensis***sp. n.**, *T.tenuipes***sp. n.**, *T.tomohonensis***sp. n.**, *T.toraja***sp. n.**, *T.vicinus***sp. n.**, *T.viduus***sp. n.**, *T.volcanorum***sp. n.**, *T.wangiwangiensis***sp. n.**, *T.watsoni***sp. n.**, and *T.yoda***sp. n.** All new species are authored by the taxonomist-in-charge, Alexander Riedel.

## Introduction

*Trigonopterus* Fauvel, a genus of flightless weevils placed in the subfamily Cryptorhynchinae of Curculionidae (Alonso-Zarazaga and Lyal 1999) currently comprises 341 species, herein brought to 444 species. It ranges from Sumatra to Samoa, and from the Philippines to Northern Australia. Its center of origin and presumably its highest diversity is in the Papuan region (Riedel et al. 2013b; Riedel et al. 2014; Tänzler et al. 2012; Tänzler et al. 2016; Toussaint et al. 2017; unpublished data). The Sunda Islands further west harbor a less diverse fauna of *Trigonopterus*; still, 99 described species have been recorded from Sumatra, Borneo, Java, and the Lesser Sunda Islands (Riedel et al. 2014). The island of Sulawesi formerly known as “Celebes” is located between these two regions (Lohman et al. 2011; Stelbrink et al. 2012), and a rich fauna can be expected from this geographic position (Whitten et al. 2012). However, only one single species, *T.fulvicornis* (Pascoe, 1885) has been described from Sulawesi to date (Riedel 2011). Our own field-work soon revealed this pattern as a sampling artefact and numerous undescribed Sulawesi *Trigonopterus* were included in the phylogeny of Tänzler et al. (2016) [supplement figure S1].

The purpose of the present publication is to make available a substantial number of names of Sulawesi *Trigonopterus* and to provide a preliminary baseline for their taxonomy. Species of the smaller offshore islands of Selayar and Wangi Wangi are also included.

We follow the previously established approach of accelerated or “fast-track” taxonomy that combines the benefits of molecular and morphological systematics (Riedel et al. 2013a, b, 2014, 2016). The taxonomic information on the genus is bundled on open access websites, i.e., species-ID (http://species-id.net/wiki/Trigonopterus), respectively wikispecies (https://species.wikimedia.org/wiki/Trigonopterus) .

The phylogenetic relationships of the species are indicated by a provisional catalogue of species groups. The name of one existing subgenus (i.e., *Mimidotasia* Voss) is being used, but we refrain from describing additional subgenera at this point. Instead, the use of informal species groups appears more adequate, as this system can be adjusted without the restrictions of nomenclature in future. Moreover, there is a lesser demand for reflecting the same hierarchy, i.e., morphologically diverse clades can be subdivided into a greater number of groups while morphologically uniform clades are left undivided.

## Materials and methods

This study is based on 3,953 specimens of *Trigonopterus*, most of them collected specifically for a larger project on this genus. Specimens were collected by tipping foliage over a beating sheet or by sifting the litter of primary forests with subsequent extraction by hand or using eclectors (Besuchet et al. 1987). Holotypes were selected from the 514 sequenced specimens; their DNA had been extracted nondestructively as described by Riedel et al. (2010). The genitalia of most specimens did not require maceration after DNA-extraction; they could be directly stained with an alcoholic Chlorazol Black solution and stored in glycerol in microvials attached to the pin of the specimens. Genitalia of collection specimens or specimens whose abdominal muscle tissue was not sufficiently digested after DNA extraction were macerated with 10% KOH and rinsed in diluted acetic acid before staining. Illustrations of habitus and genitalia were prepared from holotypes. Finally, type series were supplemented with specimens stored in ethanol and older material from the dry collection. As always the case in paratypes, there is a chance that some of these are incorrectly assigned; this is especially true for specimens without sequence data as an identification based on external morphological characters is more prone to error than an identification based on a *cox1* sequence (Tänzler et al. 2012). Type depositories are cited using the following abbreviations:

**ARC** Alexander Riedel Collection, stored in SMNK, Germany

**MZB** LIPI Research Center of Biology, Division of Zoology, Museum Zoologicum Bogoriense, Widyasatwaloka, Cibinong, Indonesia

**SMNK** Staatliches Museum für Naturkunde, Karlsruhe, Germany

**ZSM** Zoologische Staatssammlung, München, Germany

The methods applied for DNA sequencing and sequence analysis are described by Riedel et al. (2010) and Tänzler et al. (2012). Morphological descriptions are limited to major diagnostic characters as outlined by Riedel et al. (2013a, b). Negative character states (i.e., the absence of a character) are only mentioned explicitly where it appears appropriate. In groups comprising hundreds of species enumerating the absence of rare character states leads to inflated descriptions that distract the reader from the important information, i.e., the diagnostic characters present in a given species.

Morphological terminology follows Beutel and Leschen (2005) and Leschen et al. (2009), i.e., the terms “mesoventrite” / “metaventrite” are used instead of “mesosternite” / “metasternite”, and “mesanepisternum” / “metanepisternum” instead of “mesepisternum” / “metepisternum”; “penis” is used instead of “aedeagus” as the tegmen is usually without useful characters in *Trigonopterus* and therefore omitted from species descriptions. Specimens were examined with a Leica MZ16 dissecting microscope and a fluorescent desk lamp for illumination. Measurements were taken with the help of an ocular grid. The length of the body was measured in dorsal aspect from the elytral apex to the front of the pronotum. Legs were described in an idealized laterally extended position; there is a dorsal / ventral and an anterior / posterior surface. Habitus illustrations were compiled using a DFC495 camera with L.A.S. 4.8.0 software adapted to a Z6 APO (all from Leica Microsystems, Heerbrugg, Switzerland). Photographic illustrations of genitalia were made using a DFC450 camera with L.A.S. 4.8.0 software adapted to an Axio Imager M2 microscope (Carl Zeiss Microscopy), with 5×, respectively 10× A-Plan lenses; resulting image stacks were compiled using the Helicon Focus 6.7.1 Pro software (Helicon Soft Ltd). For photography genitalia were temporarily embedded in glycerol gelatin as described by Riedel (2005), with their longitudinal axis somewhat lifted anteriorly, to adequately illustrate structures of the curved down apex. All photographs were enhanced using Adobe Photoshop CS2 and CS6. However, care was taken not to obscure or alter any features of the specimens illustrated. Sequence data were submitted to the European Molecular Biology Laboratory (EMBL), respectively to GenBank of NCBI (National Center for Biotechnology Information) and the accession numbers are provided under each species e.g., as “(EMBL # LN884964)”.

Newly identified species are provided with a species number which was consistently used before a scientific name became available, e.g. in phylogenies (Tänzler et al. 2016) or for sequence data entries. To allow later interpretation of such data, these species numbers are given in “Notes”. Species groups were defined in a combined approach to agree with monophyletic clades of our molecular phylogenies (Tänzler et al. 2016: fig. S1; unpublished data) and to reflect morphological characters. Since the weevil´s life style on foliage versus edaphic in leaf litter has a great impact on its outer appearance, it was attempted to delineate groups of species with a single life style.

The abbreviation “Gn.” stands for “Gunung”, the Indonesian word for mountain and “Pc.” stands for “Puncak” (peak or pass). The Indonesian provinces of Sulawesi were transliterated as follows: “Sulawesi Selatan” = S-Sulawesi, “Sulawesi Tenggara” = SE-Sulawesi, “Sulawesi Tengah” = C-Sulawesi, “Sulawesi Utara” = N-Sulawesi.

## Taxonomy

### Trigonopterus

Taxon classificationAnimaliaColeopteraCurculionidae

Fauvel, 1862

#### Type species.

*Trigonopterusinsignis* Fauvel, 1862, by monotypy.

**Diagnosis**. Fully apterous genus of Cryptorhynchinae. Length 1.5–6.0 mm. Rostrum in repose not reaching center of mesocoxa. Scutellar shield completely absent externally. Mesothoracic receptacle deep, posteriorly closed. Metanepisternum completely absent externally. Elytra with nine striae (sometimes superficially effaced). Tarsal claws minute. Usually body largely unclothed, without dense vestiture. For additional information, see http://species-id.net/wiki/Trigonopterus.

##### Descriptions of the species

### 1. Trigonopterus abnormis

Taxon classificationAnimaliaColeopteraCurculionidae

Riedel sp. n.

http://zoobank.org/1393EC13-E94B-4BFC-B8A9-56BD94D724E5

#### Diagnostic description.

**Figure 1.**
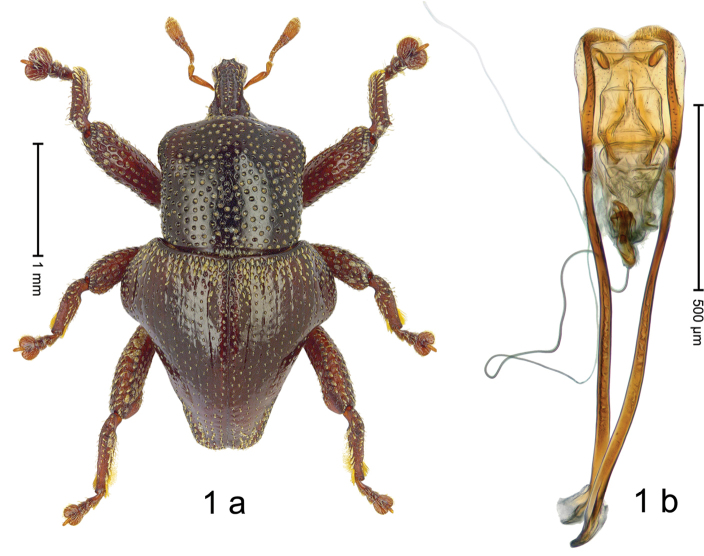
*Trigonopterusabnormis* Riedel, sp. n., holotype; **a** habitus **b** penis.

***Holotype***, male (Fig. 1a). Length 3.00 mm. Color of antennae and legs ferruginous; remainder black. Body shape dominated by laterally protruding humeri; in dorsal aspect with marked constriction between pronotum and elytron; in profile dorsally convex. Rostrum in basal 1/2 dorsally with median ridge and pair of submedian ridges; anteriorly coarsely punctate; furrows with rows of erect subclavate scales; epistome with transverse ridge and submedian pair of denticles. Pronotum anteriorly with lateral flanges; disk subquadrate, coarsely punctate; interspaces subglabrous. Elytra with humeri markedly protruding; densely irregularly punctate with small punctures, near base punctures larger; striae indistinct, some marked by hairlines; sutural interval in basal 1/3 swollen, densely irregularly punctate; swollen humeri densely coarsely punctate; subbasally with yellowish almond-shaped scales; subapically with sparse narrow scales. Femora with anteroventral ridge distinct, crenate, in meso- and metafemur ending with denticle; anterior surface punctate-reticulate, each puncture with a scale, dorsally scales erect and subclavate. Metafemur with dorsoposterior edge serrate; subapically with stridulatory patch. Metatibia with posterior surface densely setose in subapical 1/2, with erect scales in basal 1/2. Abdominal ventrites 1–2 concave, subglabrous. Abdominal ventrite 5 flat, densely coarsely punctate. Penis (Fig. 1b) with body differentiated into hyaline lateral flanges and sclerotized inner frame; sides of body weakly diverging; apex with median constriction; apodemes 3.1 × as long as body of penis; transfer apparatus complex, asymmetrical; ductus ejaculatorius without distinct bulbus. ***Intraspecific variation***. Length 2.20–3.00 mm. Female rostrum dorsally flattened, with ridges less distinct; epistome indistinct. Female pronotum without lateral flanges. Female elytra without swollen humeri.

#### Material examined.

***Holotype*** (MZB): ARC3142 (EMBL # LN884964), SE-Sulawesi Prov., Kendari, road from Wawotobi to Lasolo, 03°44.142'S 122°13.670'E, 482 m, sifted, 17-IV-2013. ***Paratypes*** (SMNK, MZB): 5 exx, ARC3143 (GenBank # MK260434), ARC3144 (GenBank # MK260436), ARC3145 (GenBank # MK260435), same data as holotype.

#### Distribution.

SE-Sulawesi Prov. (Kendari). Elevation ca. 480 m.

#### Biology.

In leaf litter of lowland forest.

#### Etymology.

This epithet is based on the Latin adjective *abnormis*, -*e* (abnormal) and refers to the species´ unusual body shape.

#### Notes.

*Trigonopterusabnormis* sp. n. was coded as “*Trigonopterus* sp. 481”.

### 2. Trigonopterus adspersus

Taxon classificationAnimaliaColeopteraCurculionidae

Riedel sp. n.

http://zoobank.org/0903BE32-9040-4803-87CF-33B73D6F848B

#### Diagnostic description.

**Figure 2.**
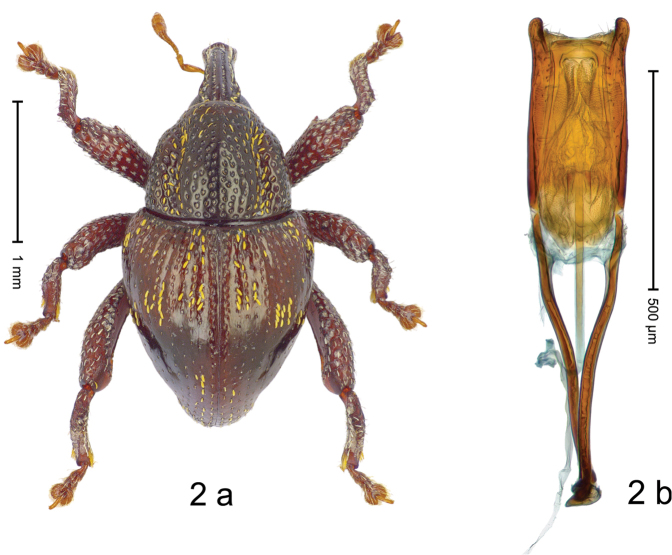
*Trigonopterusadspersus* Riedel, sp. n., holotype; **a** habitus **b** penis.

***Holotype***, male (Fig. 2a). Length 2.30 mm. Color of antennae ferruginous; legs and basal 1/2 of elytra dark ferruginous; remainder black. Body subovate; in dorsal aspect and in profile with moderate constriction between pronotum and elytron. Rostrum dorsally with median and pair of submedian ridges; intervening furrows with sparse, erect scales; epistome weakly swollen, simple. Pronotum with lateral edges weakly converging, with weak subapical constriction; disk with pair of distinct longitudinal impressions, lined with sparse yellow almond-shaped scales; medially broadly swollen, densely punctate with coarse punctures, with narrow subglabrous midline. Elytra punctate with small punctures; near base and at humeral angles more densely punctate with coarse punctures; striae hardly marked by hairlines; with patches of sparse yellow recumbent scales. Femora dentate, with acute tooth; anteroventral ridges crenate. Metafemur dorsally denticulate; subapically with stridulatory patch. Dorsoposterior edge of tibiae subbasally denticulate. Metatibia weakly curved. Abdominal ventrites 1–2 concave, subglabrous, with sparse scales; ventrite 5 concave, with sparse punctures. Penis (Fig. 2b) with sides of body subparallel, apex subtruncate, with few short setae; apodemes 1.6 × as long as body of penis; transfer apparatus flagelliform, subequal to length of body of penis; ductus ejaculatorius without distinct bulbus.

#### Material examined.

***Holotype*** (MZB): ARC3146 (EMBL # LN884965), SE-Sulawesi Prov., Kendari, road from Wawotobi to Lasolo, 03°44.142'S 122°13.670'E, 482 m, sifted, 17-IV-2013.

#### Distribution.

SE-Sulawesi Prov. (Kendari). Elevation ca. 480 m.

#### Biology.

In leaf litter of lowland forest.

#### Etymology.

This epithet is based on the Latin participle *adspersus* (sprinkled, strewn on) and refers to the species´ integument with scattered scales.

#### Notes.

*Trigonopterusadspersus* Riedel, sp. n. was coded as “*Trigonopterus* sp. 489”.

### 3. Trigonopterus allotopus

Taxon classificationAnimaliaColeopteraCurculionidae

Riedel

Trigonopterus allotopus Riedel: Riedel et al. 2014: 12–13.

#### Diagnostic description.

**Figure 3:**
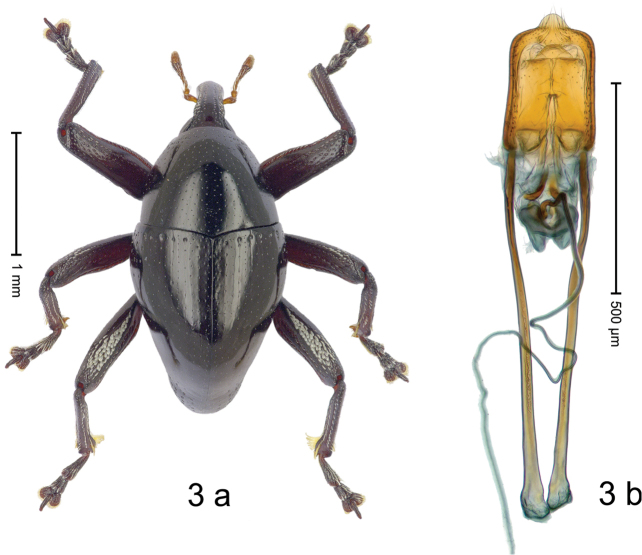
. *Trigonopterusallotopus* Riedel, Kolaka (ARC3164); **a** habitus **b** penis.

Fig. 3a. Length 2.40–2.63 mm. Color of antennae ferruginous, legs dark ferruginous or black; remainder black. Body ovate, almost without constriction between pronotum and elytron; in profile dorsally convex. Rostrum dorsally punctate, weakly rugose; dorsolateral furrows with rows of piliform scales. Eyes with dorsal margin bordered by furrow, continuous with forehead, not carinate. Pronotum subglabrous, sparsely punctate with minute punctures, laterally above procoxa with sparse coarse punctures. Elytra subglabrous, striae hardly visible but with few deeper punctures along basal margin and near apex; striae 7–9 at humerus marked by coarse punctures. Femora subglabrous, with minute punctures; with anteroventral ridge distinct, simple. Posterior surface of metafemur with two longitudinal furrows; with simple dorsoposterior edge; subapically without stridulatory patch. Mesotibia basally rounded; males subapically with uncus and larger premucro, females with minute premucro. Male metatibia subapically with fringe of curved, white setae, missing in female; with uncus, without premucro. Abdominal ventrite 2 swollen, with posterior edge projecting, in males medially forming common cavity with ventrite 1; ventrite 5 dull, microreticulate, punctate, with shallow subapical depression. Penis (Fig. 3b). Apex symmetrical, with median triangular extension; apodemes 2.8 × as long as body of penis; transfer apparatus dentiform, apically bordered by pair of L-shaped sclerites; ductus ejaculatorius with indistinct bulbus.

#### Material examined.

***Holotype*** (MZB): ARC1513 (EMBL # LM655611), West Nusa Tenggara Prov., Sumbawa, Batu Dulang, Mt Batu Pasak, sample 2, 08°37.028'S 117°15.783'E, 1305 m, 12-IV-2010. Other material examined (SMNK): SE-Sulawesi Prov.: 4 exx, ARC3163 (GenBank # MK260197), ARC3164 (GenBank # MK260199), ARC3165 (GenBank # MK260198), Kolaka, km 45 on Kendari-road, Talodo village, 04°00.686'S 121°50.198'E to 04°00.673'S 121°49.869'E, 450–565 m, 20-IV-2013.

#### Distribution.

West Nusa Tenggara Prov. (Sumbawa); SE-Sulawesi Prov. (Kolaka). Elevation 565–1305 m.

#### Notes.

This species originally described from Sumbawa Island was coded as “*Trigonopterus* sp. 331” by Tänzler et al. (2014). It is closely related to *T.pseudallotopus* Riedel, sp. n., from which it differs by ca. 5.8–6.4% p-distance of *cox1* and morphologically by the deeply impressed striae 7–9 of the elytral humeri.

### 4. Trigonopterus ambangensis

Taxon classificationAnimaliaColeopteraCurculionidae

Riedel sp. n.

http://zoobank.org/179145C5-3FD4-4075-ADFE-698FFBAE82D5

#### Diagnostic description.

**Figure 4.**
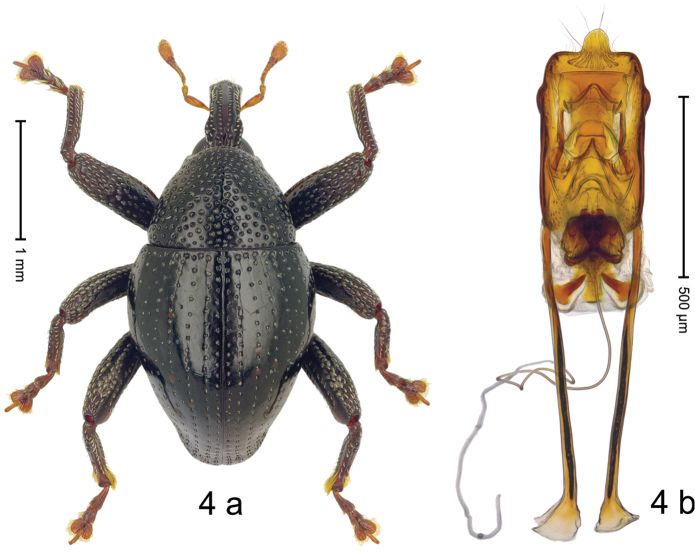
*Trigonopterusambangensis* Riedel, sp. n., holotype; **a** habitus **b** penis.

***Holotype***, male (Fig. 4a). Length 2.60 mm. Color of antennae and tarsi ferruginous; remainder black. Body subovate; in dorsal aspect with weak constriction between pronotum and elytron; in profile dorsally convex. Rostrum dorsally with broad median costa and pair of submedian ridges; intervening furrows each with row of subrecumbent scales. Pronotum with disk densely punctate with coarse punctures; interspaces between punctures subglabrous, smaller than punctures´ diameter. Elytra with striae marked by distinct rows of small punctures; basal margin bordered by transverse row of deeper punctures joining stria 8; sutural interval with additional row, other intervals subglabrous, with few interspersed punctures. Femora edentate; anterior surface coarsely punctate, each puncture with recumbent white narrow scale. Metafemur with dorsoposterior edge denticulate; subapically with stridulatory patch. Metatibia subapically with dense cluster of setae. Abdominal ventrites 1–2 concave, subglabrous, laterally with suberect scales; ventrite 5 at middle with deep depression, subapically densely punctate and laterally with few erect scales. Penis (Fig. 4b) with sides of body weakly subparallel, in apical 1/3 with lateral swelling; apex subtruncate, with median extension and sparse setae; apodemes 1.5 × as long as body of penis; transfer apparatus complex; ductus ejaculatorius without bulbus. ***Intraspecific variation***. Length 2.40–2.76 mm. Female rostrum with low, widened median costa; separated from submedian costae by row of punctures. Female abdominal ventrite 5 flat, evenly punctate, with sparse scales.

#### Material examined.

***Holotype*** (MZB): ARC5250 (GenBank # MK260278), N-Sulawesi Prov., NE of Kotamobagu, Modoinding, Gn. Ambang, east slope, 00°45.71'N 124°25.41'E, 1500 m, hand-collected, 25-XI-2012. ***Paratypes*** (ARC, MZB, SMNK): N-Sulawesi Prov., NE of Kotamobagu, Modoinding, Gn. Ambang: 1 ex, ARC0341 (EMBL # HG939577), 00°45.533'N 123°25.383'E, 1480 m, 20-VI-2006; 1 ex, ARC5240 (GenBank # MK260276), 00°46.86'N 124°29.42'E, 1480 m, 28-XII-2012; 2 exx, ARC5251 (GenBank # MK260275), ARC5252 (GenBank # MK260277), same data as holotype; 1 ex, 1450 m, sifted, 06-XII-1999; 6 exx, 1200–1450 m, beaten, 12-XII-1999.

#### Distribution.

N-Sulawesi Prov. (Mt Ambang). Elevation 1480–1500 m.

#### Biology.

On foliage in montane forests.

#### Etymology.

This epithet is a Latinized adjective based on the type locality Mt Ambang.

#### Notes.

*Trigonopterusambangensis* Riedel, sp. n. was coded as “*Trigonopterus* sp. 393”.

### 5. Trigonopterus ampanensis

Taxon classificationAnimaliaColeopteraCurculionidae

Riedel sp. n.

http://zoobank.org/DEA64FC0-2AC2-4D18-8903-D0A855A86717

#### Diagnostic description.

**Figure 5.**
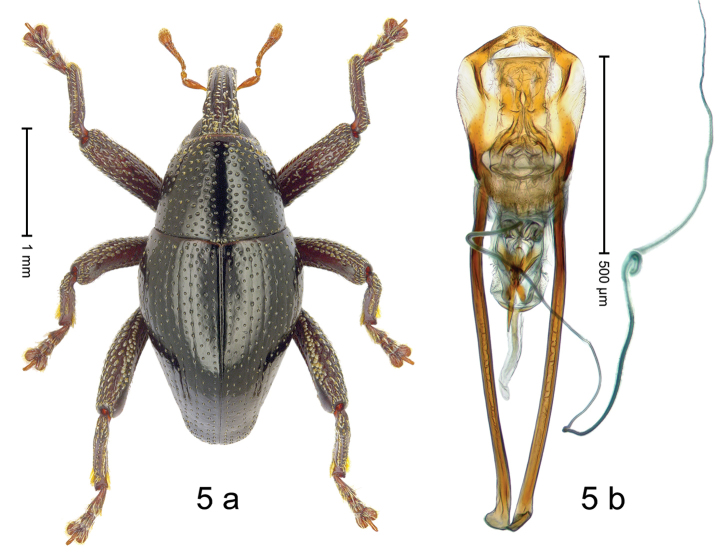
*Trigonopterusampanensis* Riedel, sp. n., holotype; **a** habitus **b** penis.

***Holotype***, male (Fig. 5a). Length 2.95 mm. Color of antennae and tarsi ferruginous; legs dark ferruginous; remainder black. Body subrhomboid; in dorsal aspect with very weak constriction between pronotum and elytron; in profile dorsally convex. Rostrum dorsally with median and pair of submedian carinae; intervening furrows with rows of subrecumbent silvery scales; epistome indistinct, somewhat polished, sparsely setose. Pronotum with disk densely punctate; interspaces between punctures subglabrous, subequal to punctures´ diameter; each puncture with fine, recumbent seta; median line impunctate; laterally with weak subapical constriction, anteriorly with sparse subclavate scales. Elytra irregularly punctate with small punctures; interspaces subglabrous; striae 2–6 marked by fine hairlines. Femora edentate; anteroventral ridge of mesofemur and metafemur crenate; anterior surface coarsely punctate-reticulate, each puncture with subrecumbent narrow scale. Metafemur dorsally with 2–3 rows of suberect silvery scales; dorsoposterior edge denticulate; subapically with stridulatory patch. Metatibia subapically with weak dorsal constriction. Abdominal ventrites 1–2 concave, sublaterally with erect silvery scales; ventrite 5 flat, punctate. Penis (Fig. 5b) with sides diverging, body with lateral flanges; apex subangulate, without setae; apodemes 2.8 × as long as body of penis; transfer apparatus spiniform, directed basad in repose, subequal in length to body of penis; ductus ejaculatorius with indistinct bulbus. ***Intraspecific variation***. Length 2.73–3.03 mm. Female rostrum slender, dorsally subglabrous, with submedian row of punctures and sublateral furrows.

#### Material examined.

***Holotype*** (MZB): ARC3256 (GenBank # MK260561), C-Sulawesi Prov., Ampana, Bongka, Mire, 01°08.399'S 121°26.545'E to 01°08.202'S 121°26.673'E, 400–450 m, beaten, 19-V-2013. ***Paratypes*** (MZB, SMNK): C-Sulawesi Prov., Ampana: 39 exx, ARC3257 (GenBank # MK260562), ARC3258 (GenBank # MK260564), same data as holotype; 2 exx, Mire, Bongka, 01°08.399'S 121°26.545'E, 426 m, sifted, 19-V-2013; 22 exx, ARC3236 (GenBank # MK260563), ARC3237 (GenBank # MK260559), ARC3238 (GenBank # MK260560), Tanjung Api, 00°49.855'S 121°36.493'E to 00°49.687'S 121°36.560'E, 130–166 m, 18-V-2013.

#### Distribution.

S-Sulawesi Prov. (Ampana). Elevation 160–430 m.

#### Biology.

On foliage in lowland forests.

#### Etymology.

This epithet is a Latinized adjective based on the town of Ampana.

#### Notes.

*Trigonopterusampanensis* Riedel, sp. n. was coded as “*Trigonopterus* sp. 523”.

### 6. Trigonopterus analis

Taxon classificationAnimaliaColeopteraCurculionidae

Riedel sp. n.

http://zoobank.org/F7AFE612-F2D1-4AED-A5C4-3B0212B6F5CE

#### Diagnostic description.

**Figure 6.**
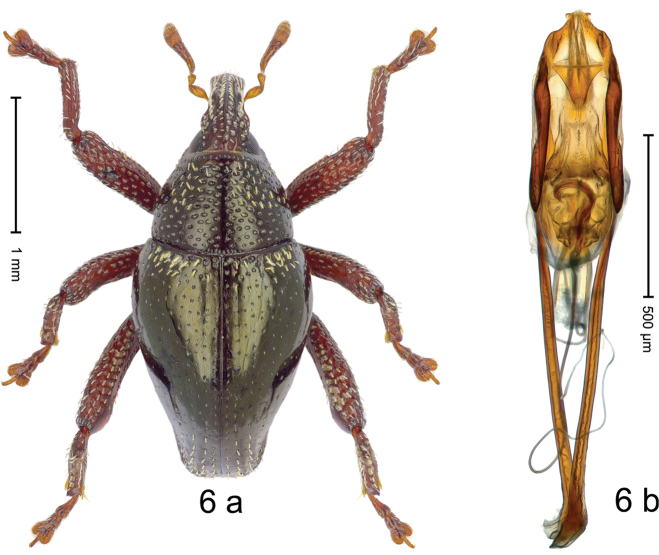
*Trigonopterusanalis* Riedel, sp. n., holotype; **a** habitus **b** penis.

***Holotype***, male (Fig. 6a). Length 2.54 mm. Color of antennae and legs ferruginous; remainder black with slight bronze luster. Body subovate; in dorsal aspect with weak constriction between pronotum and elytron; in profile dorsally convex. Rostrum dorsally with median and pair of submedian carinae; intervening furrows with rows of erect yellowish scales; epistome indistinct, sparsely setose. Pronotum with disk densely, coarsely punctate; interspaces between punctures subglabrous, subequal or less than punctures´ diameter; sublaterally some punctures with clavate, yellowish scale; median line impunctate. Elytra irregularly punctate with small punctures; interspaces subglabrous; basal margin bordered by deeper punctures and almond-shaped yellowish scales; near apex striae more distinctly impressed, with sparse rows of yellowish scales; apex subtruncate. Femora with anteroventral ridge ending with blunt tooth; anterior surface coarsely punctate, each puncture with narrow yellowish scale. Metafemur with dorsoposterior ridge denticulate, subapically with stridulatory patch. Meso- and metatibia with dorsal edge irregularly serrate. Metatibia ventrally with fine ridge; subapically at base of uncus with fine pointed brush of long setae. Abdominal ventrites 1–2 concave, sublaterally behind metacoxa with patch of erect white scales; ventrite 5 concave, subglabrous, microreticulate, in basal 1/2 with median carina, apex curved ventrad forming thin bilobed lamina. Penis (Fig. 6b) with body differentiated into weakly sinuate inner frame and narrow lateral membranous lobes; converging towards weakly anchor-shaped apex; apodemes 1.8 × as long as body of penis; transfer apparatus hook-shaped, ca. 0.6 × length of body; ductus ejaculatorius without distinct bulbus. ***Intraspecific variation***. Length 2.38–2.54 mm. Female rostrum slender, dorsally subglabrous, with submedian and sublateral rows of minute punctures. Female abdominal ventrite 5 simple, flat, with sparse scales.

#### Material examined.

***Holotype*** (MZB): ARC3179 (EMBL # LN884969), SE-Sulawesi Prov., N Kolaka, Uluwolo village, 03°48.073'S 121°16.856'E, 182 m, sifted, 21-IV-2013. ***Paratype*** (SMNK): 1 ex, ARC3180 (PCR failed), same data as holotype.

#### Distribution.

SE-Sulawesi Prov. (Kolaka). Elevation ca. 180 m.

#### Biology.

In leaf litter of lowland forest.

#### Etymology.

This epithet is based on the Latin adjective *analis*, *e* (pertaining to the anus) and refers to the peculiar morphology of ventrite 5.

#### Notes.

*Trigonopterusanalis* Riedel, sp. n. was coded as “*Trigonopterus* sp. 473”. This species is very closely related to *T.pseudanalis* Riedel, sp. n., from which it differs by ca. 11.7–12.7% p-distance of *cox1*, by the apex of the penis, and by a more slender body shape with a less densely punctate pronotum.

### 7. Trigonopterus arachnobas

Taxon classificationAnimaliaColeopteraCurculionidae

Riedel sp. n.

http://zoobank.org/3CF8926F-808D-443B-B852-97043703A6C0

#### Diagnostic description.

**Figure 7:**
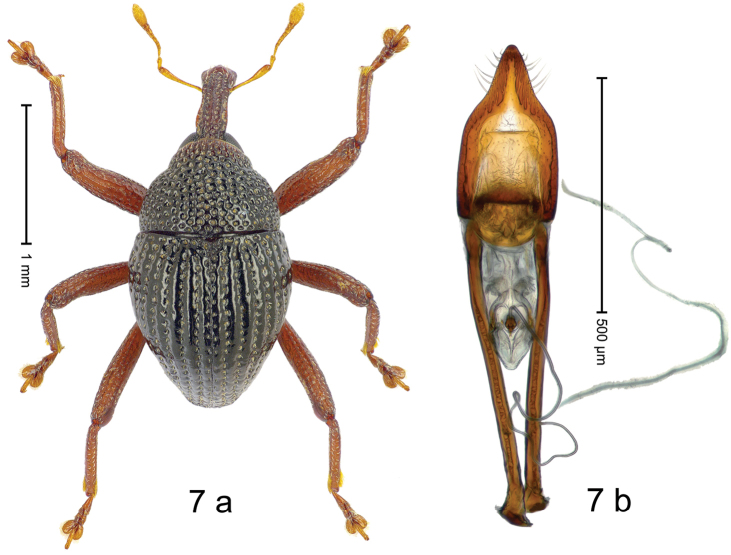
. *Trigonopterusarachnobas* Riedel, sp. n., holotype; **a** habitus **b** penis.

***Holotype***, male (Fig. 7a). Length 2.04 mm. Color of antennae yellowish; legs and rostrum dark ferruginous; remainder black. Body subovate; in profile with distinct constriction between pronotum and elytron. Rostrum long; dorsally with median ridge and pair of sublateral somewhat irregular ridges; intervening furrows with punctures and sparse rows of setae; epistome simple, subglabrous. Pronotum with disk densely, coarsely punctate; interspaces between punctures reticulate, wrinkled. Elytra with striae deeply impressed, intervals costate, subglabrous; punctures each with short, suberect seta. Legs long. Femora edentate. Anteroventral ridges indistinct, simple; anterior surface of femora coriaceous, punctate. Metafemur subapically with stridulatory patch. Abdominal ventrite 1 weakly concave, microreticulate, with coarse punctures; ventrite 5 subglabrous, with subquadrate depression. Penis (Fig. 7b) with sides subparallel in basal 1/2; in apical 1/3 converging in sinuate line forming long, pointed, downwardly curved extension, laterally with sparse setae; basal orifice with well-sclerotized ventral plate; apodemes 2.4 × as long as body of penis; transfer apparatus dentiform, directed basad in repose; ductus ejaculatorius with indistinct bulbus. ***Intraspecific variation***. Length 1.98–2.04 mm.

#### Material examined.

***Holotype*** (MZB): ARC3014 (GenBank # MK260543), S-Sulawesi Prov., Tanah Toraja, Rantepao, Gn. Karre (= Gn. Wokim), 02°59.013'S 120°02.251'E, 1423 m, sifted, 06-V-2013. ***Paratypes*** (MZB, SMNK): 2 exx, ARC3015 (GenBank # MK260546), same data as holotype; 2 exx, ARC6024 (GenBank # MK260544), ARC6025 (GenBank # MK260545), Tanah Toraja, Rantepao, Gn. Karre (= Gn. Wokim), 02°59.021'S 120°02.523'E, 1456 m, 06-V-2013, sifted.

#### Distribution.

S-Sulawesi Prov. (Tanah Toraja). Elevation 1420–1460 m.

#### Biology.

In leaf litter of montane forest.

#### Etymology.

This epithet is based on the genus name *Arachnobas* Boisduval and is treated as a noun in apposition. It refers to the superficial resemblance of the species´ habitus.

#### Notes.

*Trigonopterusarachnobas* Riedel, sp. n. was coded as “*Trigonopterus* sp. 517”.

### 8. Trigonopterus armipes

Taxon classificationAnimaliaColeopteraCurculionidae

Riedel sp. n.

http://zoobank.org/8DF8DE54-6853-4903-BE18-034953EE8AF7

#### Diagnostic description.

**Figure 8.**
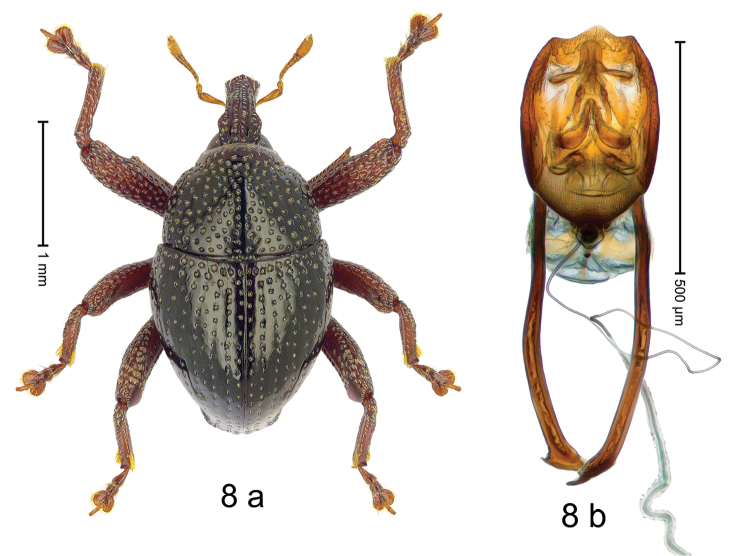
*Trigonopterusarmipes* Riedel, sp. n., holotype; **a** habitus **b** penis.

***Holotype***, male (Fig. 8a). Length 2.39 mm. Color of antennae and legs ferruginous; remainder black. Body subovate; in profile dorsally convex, with weak constriction between pronotum and elytron. Rostrum dorsally with median and pair of submedian ridges; intervening furrows with sparse rows of suberect setae; epistome posteriorly with transverse, angulate ridge. Pronotum with weak subapical constriction; densely punctate disk forming broad ridge with impunctate median line; interspaces between punctures subglabrous, subequal to or smaller than punctures´ diameter. Elytra with striae marked by rows of small punctures; basal margin bordered by transverse row of slightly deeper punctures; near humerus striae 7–9 with large, coarse punctures; sutural interval with additional row, other intervals subglabrous, with few interspersed punctures. Femora with anteroventral ridges crenate, ending with acute tooth; anterior surface coarsely punctate, each puncture with subrecumbent yellowish seta. Metafemur with dorsoposterior edge denticulate; subapically with stridulatory patch. Abdominal ventrite 1 and anterior portion of ventrite 2 cavernous, subglabrous; ventrite 2 posteriorly forming rim; ventrite 5 broadly concave, sparsely punctate. Penis (Fig. 8b) with sides of body rounded to medially pointed apex; apodemes 2.0 × as long as body of penis; transfer apparatus with complex sclerites; ductus ejaculatorius with indistinct bulbus. ***Intraspecific variation***. Length 2.13–2.39 mm. Female rostrum dorsally punctate-rugose, with median costa; epistome indistinct. Female abdominal ventrite 5 flat.

#### Material examined.

***Holotype*** (MZB): ARC2900 (EMBL # LN884944), N-Sulawesi Prov., Kotamobagu, Modoinding, Lake Moat area, 00°42.862'N 124°28.356'E, 1024 m, beaten, 19-V-2012. ***Paratypes*** (MZB, SMNK): N-Sulawesi Prov.: 2 exx, ARC2901 (GenBank # MK260296), ARC2902 (GenBank # MK260297), same data as holotype; 7 exx, Kotamobagu, Modoinding, Lake Moat area, 00°42.862'N 124°28.356'E, 1024 m, sifted, 19-V-2012; 5 exx, ARC0333 (PCR failed), ARC0334 (GenBank # MK260299), ARC0335 (GenBank # MK260298), ARC0336 (PCR failed), Gn. Bonde near Matayangan, Bogani Nani Wartabone N.P., 00°27.300'N 123°56.950'E, 908 m, 18-VI-2006.

#### Distribution.

N-Sulawesi Prov. (Lake Moat, Mt Bonde). Elevation: 910–1020 m.

#### Biology.

On foliage and in leaf litter in montane forests.

#### Etymology.

This epithet is a combination of the Latin nouns *arma* (weapon) and *pes* (foot, leg). It is treated as a noun in apposition.

#### Notes.

*Trigonopterusarmipes* Riedel, sp. n. was coded as “*Trigonopterus* sp. 397”. Populations from Lake Moat and Mt Bonde exhibit 10.7–11.1% p-distance in *cox1* sequences, but no significant morphological differences could be found.

### 9. Trigonopterus artemis

Taxon classificationAnimaliaColeopteraCurculionidae

Riedel sp. n.

http://zoobank.org/6A038220-5377-4C4F-9DD8-DC9289B0DBF3

#### Diagnostic description.

**Figure 9.**
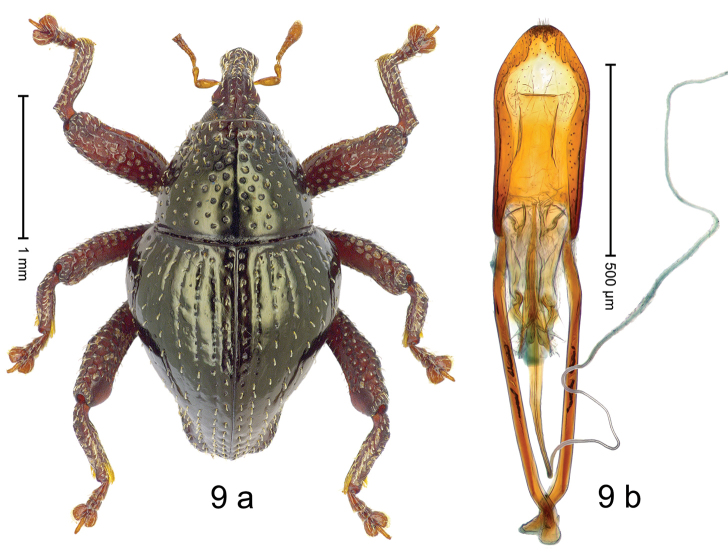
*Trigonopterusartemis* Riedel, sp. n., holotype; **a** habitus **b** penis.

***Holotype***, male (Fig. 9a). Length 2.53 mm. Color of antennae and tarsi ferruginous; remainder black with bronze luster. Body subovate; in dorsal aspect with marked constriction between pronotum and elytron; in profile dorsally convex. Rostrum dorsally punctate-rugose, in basal 1/2 with median and pair of indistinct submedian ridges, anteriorly coarsely punctate-rugose, with sparse, erect, subclavate scales; epistome posteriorly with transverse, subangulate ridge. Pronotum with disk densely punctate; anteriorly punctures larger and containing each one suberect, clavate scale; interspaces between punctures subglabrous, anteriorly smaller, posteriorly larger than punctures´ diameter. Elytra with striae marked by fine lines and rows of fine punctures; with suberect, narrow scales, becoming denser towards apex; sutural interval with row of small punctures, other intervals subglabrous; apex laterally swollen, subtruncate. Femora edentate; surface densely coarsely punctate, each puncture with suberect scale, with sparse denticles. Metafemur subapically with stridulatory patch. Abdominal ventrites 1–2 cavernous, subglabrous, lateral rims with sparse erect scales; ventrite 5 dull, microreticulate, at middle with shallow impression. Penis (Fig. 9b) with sides of body subparallel, converging in apical 1/3; median extension with sparse short setae; apodemes 1.4 × as long as body of penis; transfer apparatus spear-shaped, 0.7 × as long as body of penis; ductus ejaculatorius basally sclerotized, with indistinct bulbus. ***Intraspecific variation***. Length 2.53–2.93 mm. Female rostrum slender, dorsally coarsely punctate-rugose with median and pair of submedian ridges; epistome simple. Female abdominal ventrite 5 flat, with sparse erect scales.

#### Material examined.

***Holotype*** (MZB): ARC2803 (GenBank # MK260270), N-Sulawesi Prov., Airmadidi, Gn. Klabat, 01°26.635'N 125°00.823'E, 1031 m, sifted, 16-V-2012. ***Paratypes*** (SMNK): N-Sulawesi Prov.: 2 exx, ARC2779 (GenBank # MK260269), ARC2780 (GenBank # MK260268), same data as holotype.

#### Distribution.

N-Sulawesi Prov. (Mt Klabat). Elevation ca. 1030 m.

#### Biology.

In leaf litter of montane forest.

#### Etymology.

This epithet is the name of the Greek goddess of hunting and nature, Ártemis. It is a noun in apposition.

#### Notes.

*Trigonopterusartemis* Riedel, sp. n. was coded as “*Trigonopterus* sp. 390”.

### 10. Trigonopterus asterix

Taxon classificationAnimaliaColeopteraCurculionidae

Riedel sp. n.

http://zoobank.org/D9607DCA-15DB-4EE2-BC5F-B005A0169F5E

#### Diagnostic description.

**Figure 10.**
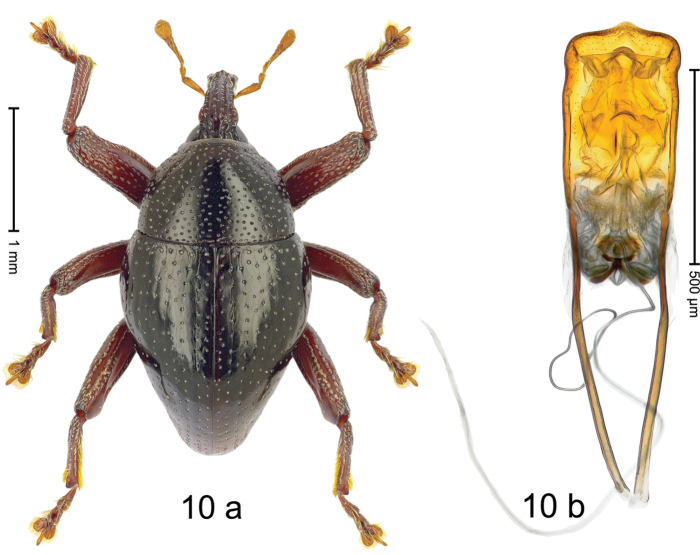
*Trigonopterusasterix* Riedel, sp. n., holotype; **a** habitus **b** penis.

***Holotype***, male (Fig. 10a). Length 2.68 mm. Color of antennae and legs ferruginous; remainder black. Body subovate; in dorsal aspect with weak constriction between pronotum and elytron; in profile dorsally convex. Rostrum dorsally with median and pair of submedian ridges; intervening furrows each with sparse row of recumbent piliform scales; epistome posteriorly with subangulate, transverse, irregularly interrupted ridge. Pronotum with disk punctate with small punctures; each puncture containing a single seta; interspaces subglabrous. Elytra punctate with small punctures; striae marked by fine hairlines; irregular intervals each with additional sparse row of punctures; interspaces between punctures subglabrous; stria 8 along humerus with four large, coarse punctures, externally bordered by weakly raised interval 9. Femora edentate; anterior surface punctate with small punctures, each with recumbent piliform scale. Metafemur subapically with stridulatory patch. Protibia widened to apex. Posterior face of metatibia in apical 1/2 with dense fringe of yellowish setae. Abdominal ventrites 1–2 concave, subglabrous; ventrite 5 with deep, subglabrous pit, laterally and subapically punctate and sparsely setose. Penis (Fig. 10b) with sides of body subparallel; apex subangulate, without setae; body dorsally with median ridge, forming angulate profile; apodemes 2.0 × as long as body; transfer apparatus complex, with basal sclerite shaped as an inverted V; ductus ejaculatorius with indistinct bulbus. ***Intraspecific variation***. Length 2.40–2.81 mm. Female rostrum slender, dorsally with median row of punctures and pair of sublateral furrows; epistome simple. Female metatibia only subapically setose. Female ventrite 5 flat, subglabrous, sparsely punctate.

#### Material examined.

***Holotype*** (MZB): ARC3007 (GenBank # MK260221), S-Sulawesi Prov., Tanah Toraja, Rantepao, Gn. Karre (= Gn. Wokim), 02°59.013'S 120°02.251'E, 1423 m, beaten, 06-V-2013. ***Paratypes*** (MZB, SMNK): S-Sulawesi Prov.: 6 exx, ARC2993 (GenBank # MK260222), ARC5988 (GenBank # MK260216), ARC5992 (GenBank # MK260226), Tanah Toraja, Rantepao, Gn. Karre (= Gn. Wokim), 02°59.021'S 120°02.523'E, 1456 m, 06-V-2013, beaten; 1 ex, ARC ARC6028 (GenBank # MK260225), Tanah Toraja, Rantepao, Gn. Karre (= Gn. Wokim), 02°59.021'S 120°02.523'E, 1456 m, 06-V-2013, sifted; 4 exx, Tanah Toraja, Rantepao, Gn. Karre (= Gn. Wokim), 02°58.846'S 120°02.158'E, 1396 m, beaten, 06-V-2013; 4 exx, ARC3008 (GenBank # MK260220), ARC6003 (GenBank # MK260227), same data as holotype; 24 exx, ARC2824 (GenBank # MK260223), ARC2825 (GenBank # MK260219), ARC2826 (GenBank # MK260218), ARC2827 (GenBank # MK260217), ARC6050 (GenBank # MK260224), Pc. Palopo, Gn. Sampuna, 02°56.539'S 120°05.320'E to 02°56.545'S 120°05.595'E, 1038–1101 m, 29-V-2012, beaten; 1 ex, ARC6005 (GenBank # MK260228), Pc. Palopo, Gn. Sampuna, 02°56.539'S 120°05.320'E to 02°56.545'S 120°05.595'E, 1038–1101 m, 02-V-2013, beaten.

#### Distribution.

S-Sulawesi Prov. (Rantepao, Pc. Palopo). Elevation 1100–1460 m.

#### Biology.

On foliage in montane forests.

#### Etymology.

This epithet is based on a character of the French Asterix comics. It is a noun in apposition.

#### Notes.

*Trigonopterusasterix* Riedel, sp. n. was coded as “*Trigonopterus* sp. 378”. This species is very closely related to *T.fuscipes* Riedel, sp. n., from which it differs by ca. 12% p-distance of *cox1* and by the structure of the male genitalia.

### 11. Trigonopterus barbipes

Taxon classificationAnimaliaColeopteraCurculionidae

Riedel sp. n.

http://zoobank.org/1DBB9806-71AF-43F2-9F82-F60B56742406

#### Diagnostic description.

**Figure 11.**
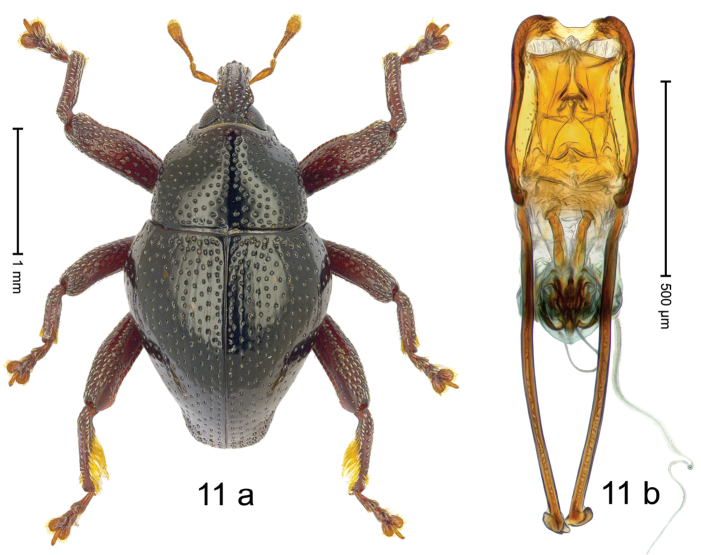
*Trigonopterusbarbipes* Riedel, sp. n., holotype; **a** habitus **b** penis.

***Holotype***, male (Fig. 11a). Length 2.65 mm. Color of antennae and legs ferruginous; remainder black. Body subovate; in dorsal aspect with distinct constriction between pronotum and elytron; in profile with weak constriction. Rostrum dorsally with median costa and pair of submedian ridges; intervening furrows each with row of suberect scales; apical 1/3 punctate-rugose, with indistinct epistome. Pronotum with weak subapical constriction; disk densely punctate; interspaces between punctures subglabrous; indistinct lateral edges more densely punctate, median line impunctate. Elytra irregularly punctate; some striae marked by hardly visible hairlines; basal margin bordered by denser punctures; stria 8 along humerus with six large, coarse punctures. Femora edentate; anteroventral ridge of mesofemur and metafemur crenate; anterior surface of metafemur coarsely punctate, each puncture with subrecumbent silvery scale. Metafemur subapically with stridulatory patch. Metatibia near middle with dense, beard-like cluster of long setae; subapically ventral surface subglabrous, lined by setose fringes. Abdominal ventrites 1–2 concave, punctate, with sparse suberect lanceolate scales; ventrite 5 with distinct, round depression, with suberect lanceolate scales. Penis (Fig. 11b) with sides weakly sinuate; apex with median notch, without setae; apodemes 1.7 × as long as body of penis; transfer apparatus complex; ductus ejaculatorius with indistinct bulbus. ***Intraspecific variation***. Length 2.50–2.65 mm.

#### Material examined.

***Holotype*** (MZB): ARC3048 (GenBank # MK260526), S-Sulawesi Prov., Tanah Toraja, Bittuang, Gn. Ponding, 02°56.446'S 119°38.075'E, 1625 m, beaten, 09-V-2013. ***Paratypes*** (MZB, SMNK): 2 exx, ARC3047 (EMBL # LN884953), ARC3049 (GenBank # MK260525), same data as holotype.

#### Distribution.

S-Sulawesi Prov. (Tanah Toraja). Elevation ca. 1625 m.

#### Biology.

On foliage in montane forests.

#### Etymology.

This epithet is a combination of the Latin nouns *barba* (beard) and *pes* (foot). It refers to the setose metatibia. Invariable.

#### Notes.

*Trigonopterusbarbipes* Riedel, sp. n. was coded as “*Trigonopterus* sp. 505”.

### 12. Trigonopterus bonthainensis

Taxon classificationAnimaliaColeopteraCurculionidae

Riedel sp. n.

http://zoobank.org/51AB1F69-4C28-420C-9656-829D6EBD2B71

#### Diagnostic description.

**Figure 12.**
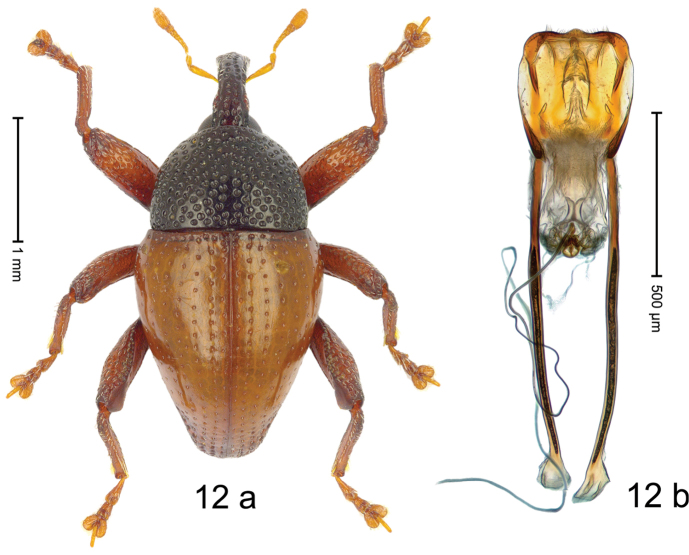
*Trigonopterusbonthainensis* Riedel, sp. n., holotype; **a** habitus **b** penis.

***Holotype***, male (Fig. 12a). Length 2.65 mm. Color of antennae, legs, and elytra orange-ferruginous; pterothorax and abdomen dark ferruginous; head and prothorax black. Body subovate; in profile dorsally convex. Rostrum dorsally coarsely punctate; sublaterally with sparse row of piliform scales; epistome short, indistinct. Pronotum with disk densely coarsely punctate; interspaces between punctures reticulate, subglabrous. Elytra with striae marked by rows of small punctures; intervals flat; basal margin bordered by transverse row of deeper punctures; stria 8 along humerus with coarse punctures. Femora edentate. Meso- and metafemur with anteroventral ridge crenate. Metafemur with dorsoposterior edge weakly denticulate; subapically with stridulatory patch. Metaventrite anteriorly at middle with puncture bearing fine pointed brush of setae. Abdominal ventrites 1–2 weakly concave, at middle subglabrous, sublaterally and posteriorly with dense coarse punctures; ventrite 5 flat, coarsely punctate. Penis (Fig. 12b) with sides of body in basal 1/2 diverging, with lateral flanges, subapically converging to subangulate apex, with sparse short setae; apodemes 3.0 × as long as body of penis; transfer apparatus small; ductus ejaculatorius without bulbus. ***Intraspecific variation***. Length 2.53–2.65 mm. Female rostrum slender, dorsally subglabrous, with two submedian rows of punctures, with pair of sublateral furrows.

#### Material examined.

***Holotype*** (MZB): ARC3205 (GenBank # MK260504), S-Sulawesi Prov., Gn. Lompobattang, Malakaji, Parambintolo, 05°23.545'S 119°55.517'E, 1750–1850 m, 27-IV-2013, beaten. ***Paratype*** (SMNK): S-Sulawesi Prov.: 1 ex, ARC3197 (EMBL # LN884974), Gn. Lompobattang, Malakaji, Parambintolo, 05°23.545'S 119°55.517'E, 1803 m, sifted, 27-IV-2013.

#### Distribution.

S-Sulawesi Prov. (Gn. Lompobattang). Elevation ca. 1800 m.

#### Biology.

Presumably mostly on foliage in montane forests.

#### Etymology.

This epithet is a Latinized adjective based on the historical locality of “Bonthain”. “The Peak of Bonthain” is identical with Mt Lompobattang.

#### Notes.

*Trigonopterusbonthainensis* Riedel, sp. n. was coded as “*Trigonopterus* sp. 500”.

### 13. Trigonopterus carinirostris

Taxon classificationAnimaliaColeopteraCurculionidae

Riedel sp. n.

http://zoobank.org/7386C1F9-C392-4F11-8808-EDADFCE644A6

#### Diagnostic description.

**Figure 13.**
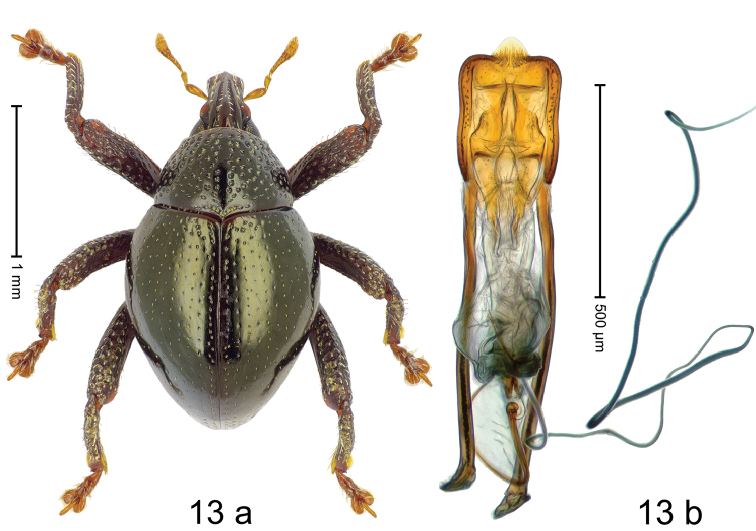
*Trigonopteruscarinirostris* Riedel, sp. n., holotype; **a** habitus **b** penis.

***Holotype***, male (Fig. 13a). Length 2.04 mm. Color of antennae and tarsi ferruginous; remainder black with bronze luster. Body subglobose; in dorsal aspect with weak constriction between pronotum and elytron; in profile dorsally convex. Rostrum dorsally with distinct median carina and pair of submedian carinae; median carina widening posteriorly, extending as wide glabrous costa onto forehead; intervening furrows each with sparse row of erect scales; epistome short, indistinct. Pronotum with disk coarsely punctate; interspaces subglabrous; basal margin medially extended, at middle subangulate. Elytra densely punctate with small punctures; interspaces subglabrous. Femora edentate; anteroventral ridge distinct, crenate; anterior surface rugose-punctate, dorsally with erect clavate scales. Metafemur dorsally denticulate; subapically with stridulatory patch. Tibial base dentiform, when leg extended tibial tooth overlapping femoral apex. Metatibia with dorsal edge denticulate. Abdominal ventrites 1–2 concave, subglabrous; ventrite 5 flat, in basal 1/2 with pair of shallow sublateral depressions, subapically punctate. Penis (Fig. 13b) with sides of body subparallel; apex with median, subtriangular extension; apodemes 2.8 × as long as body of penis; transfer apparatus flagelliform, ca. 2.5 × length of body of penis; ductus ejaculatorius with indistinct bulbus. ***Intraspecific variation***. Length 1.90–2.06 mm. Female rostrum dorsally flattened, with ridges less distinct. Female abdominal ventrite 5 flat.

#### Material examined.

***Holotype*** (MZB): ARC3221 (GenBank # MK260428), S-Sulawesi Prov., Mangkutana, 02°20.306'S 120°46.792'E, 909 m, sifted, 14-V-2013. ***Paratypes*** (MZB, SMNK): S-Sulawesi Prov.: 4 exx, ARC3229 (GenBank # MK260427), ARC3230 (GenBank # MK260429), ARC3231 (GenBank # MK260430), Mangkutana, 02°20.203'S 120°46.878'E, 903 m, sifted, 14-V-2013.

#### Distribution.

S-Sulawesi Prov. (Mangkutana). Elevation 900–910 m.

#### Biology.

In leaf litter of montane forest.

#### Etymology.

This epithet is an adjectival combination of the Latin nouns *carina* (keel) and *rostrum* (snout) with the second adjectival declension ending -*is* and refers to the dorsal sculpture of the rostrum.

#### Notes.

*Trigonopteruscarinirostris* Riedel, sp. n. was coded as “*Trigonopterus* sp. 479”.

### 14. Trigonopterus castaneipennis

Taxon classificationAnimaliaColeopteraCurculionidae

Riedel sp. n.

http://zoobank.org/91E966AB-EC57-4A75-967B-262C289AE1C7

#### Diagnostic description.

**Figure 14.**
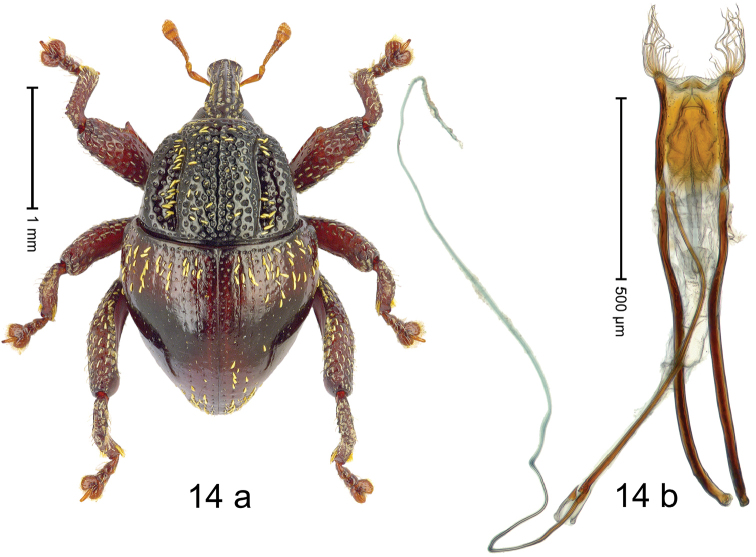
*Trigonopteruscastaneipennis* Riedel, sp. n., holotype; **a** habitus **b** penis.

***Holotype***, male (Fig. 14a). Length 2.55 mm. Color of antennae ferruginous; legs and base of elytra dark ferruginous; remainder black. Body subovate; in dorsal aspect with moderate constriction between pronotum and elytron; in profile dorsally convex. Rostrum dorsally with median and pair of submedian ridges; intervening furrows with sparse, erect scales; epistome short, posteriorly with transverse ridge. Pronotum with lateral edges weakly converging, before apex rounded to subapical constriction; disk with pair of distinct longitudinal impressions, medially broadly swollen, densely punctate with coarse punctures, narrow interspaces subglabrous, with narrow median costa; with yellowish slender recumbent scales. Elytra sparsely punctate with minute punctures; at humeral angles with some coarse punctures; striae hardly marked by hairlines; in basal 1/3 and subapically with sparse yellow recumbent scales. Femora dentate, with acute tooth; anteroventral ridge of meso- and metafemur crenate. Metafemur with dorsoposterior edge denticulate; subapically with stridulatory patch. Metatibia subbasally widened, dorsal edge serrate. Metatibia curved. Abdominal ventrites 1–2 concave, subglabrous, sparsely punctate, with few scattered piliform scales; ventrite 5 concave, sparsely punctate. Penis (Fig. 14b) with sides of body subparallel, subapically widened forming knobs with tufts of long curly setae; apodemes 3.3 × as long as body of penis; transfer apparatus flagelliform, 3.5 × longer than body of penis; ductus ejaculatorius with indistinct bulbus. ***Intraspecific variation***. Length 2.25–2.55 mm. Female rostrum dorsally with median and pair of submedian glabrous costae; epistome simple. Female ventrite 5 flat.

#### Material examined.

***Holotype*** (MZB): ARC2832 (GenBank # MK260387), S-Sulawesi Prov., Pc. Palopo, Gn. Sampuna, 02°56.545'S 120°05.595'E, 1101 m, sifted, 29-V-2012. ***Paratypes*** (MZB, SMNK): S-Sulawesi Prov.: 9 exx, ARC2833 (GenBank # MK260389), ARC2834 (GenBank # MK260390), ARC2835 (GenBank # MK260388), same data as holotype; 20 exx, Pc. Palopo, Gn. Sampuna, 02°56.545'S 120°05.595'E, 1101 m, sifted, 02-V-2013.

#### Distribution.

S-Sulawesi Prov. (Pc. Palopo). Elevation ca. 1100 m.

#### Biology.

In leaf litter of montane forest.

#### Etymology.

This epithet is an adjectival combination of the Latin adjective *castaneus*, -*a*, -*um* (chestnut brown) and noun *penna* (wing, elytron), with the second declension adjectival ending -*is*.

#### Notes.

*Trigonopteruscastaneipennis* Riedel, sp. n. was coded as “*Trigonopterus* sp. 419”.

### 15. Trigonopterus celebensis

Taxon classificationAnimaliaColeopteraCurculionidae

Riedel sp. n.

http://zoobank.org/54CE7E8A-01A5-4CC2-8C52-87C7D49E2B8D

#### Diagnostic description.

**Figure 15.**
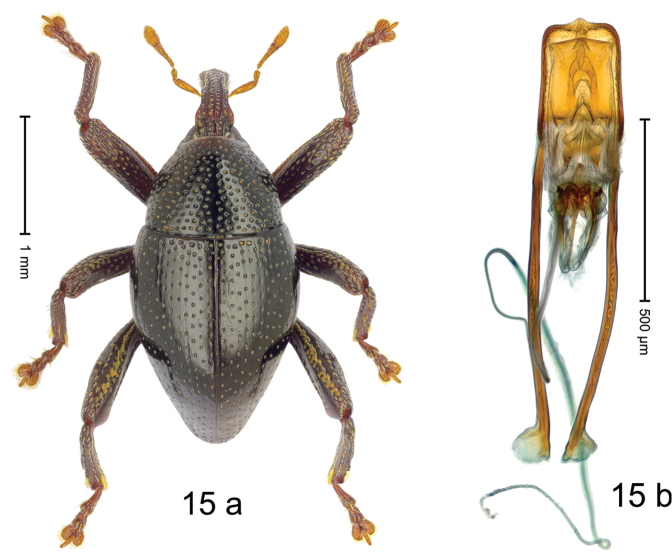
*Trigonopteruscelebensis* Riedel, sp. n., holotype; **a** habitus **b** penis.

***Holotype***, male (Fig. 15a). Length 2.58 mm. Color of antennae and tarsi ferruginous; remainder black. Body subovate; in profile dorsally convex. Rostrum dorsally with median carina; in basal 1/2 with submedian ridges; intervening furrows with sparse rows of suberect setae; epistome indistinct. Pronotum with disk densely punctate except along impunctate median line; interspaces between punctures subglabrous, subequal to or larger than punctures´ diameter. Elytra irregularly punctate with small punctures; some striae marked by hardly visible hairlines. Femora edentate. Meso- and metafemur with anteroventral ridge weakly crenate. Metafemur with dorsoposterior edge denticulate-crenate; subapically with stridulatory patch. Metatibia with dorsal edge weakly denticulate, especially near middle. Abdominal ventrites 1–2 concave, subglabrous; ventrite 5 at middle with shallow depression. Penis (Fig. 15b) with sides of body subparallel; apex subangulate, with short median extension; apodemes 3.0 × as long as body of penis; transfer apparatus spiniform, directed basad in repose by brace-like sclerites; ductus ejaculatorius with distinct bulbus. ***Intraspecific variation***. Length 2.58–2.75 mm. Female rostrum slender, dorsally subglabrous, with two submedian rows of punctures, with pair of sublateral furrows. Female abdominal ventrite 5 flat.

#### Material examined.

***Holotype*** (MZB): ARC3262 (GenBank # MK260521), C-Sulawesi Prov., Luwuk, Salodi, Gn. Taluanjang, 00°49.202'S 122°52.395'E to 00°49.307'S 122°52.322'E, 600–760 m, beaten, 21-V-2013. ***Paratypes*** (MZB, SMNK): C-Sulawesi Prov.: 13 exx, ARC3261 (GenBank # MK260520), ARC3263 (GenBank # MK260518), ARC3264 (GenBank # MK260519), same data as holotype; 1 ex, Luwuk, Salodi, Gn. Taluanjang, 00°49.307'S 122 52.322'E, 760 m, sifted, 21-V-2013.

#### Distribution.

C-Sulawesi Prov. (Luwuk). Elevation 600–760 m.

#### Biology.

On foliage in lowland forests.

#### Etymology.

This epithet is a Latinized adjective based on the former name of Sulawesi, Celebes.

#### Notes.

*Trigonopteruscelebensis* Riedel, sp. n. was coded as “*Trigonopterus* sp. 503”.

### 16. Trigonopterus cirripes

Taxon classificationAnimaliaColeopteraCurculionidae

Riedel sp. n.

http://zoobank.org/F7C2B959-C114-4340-85CC-3FDDAD0D7091

#### Diagnostic description.

**Figure 16.**
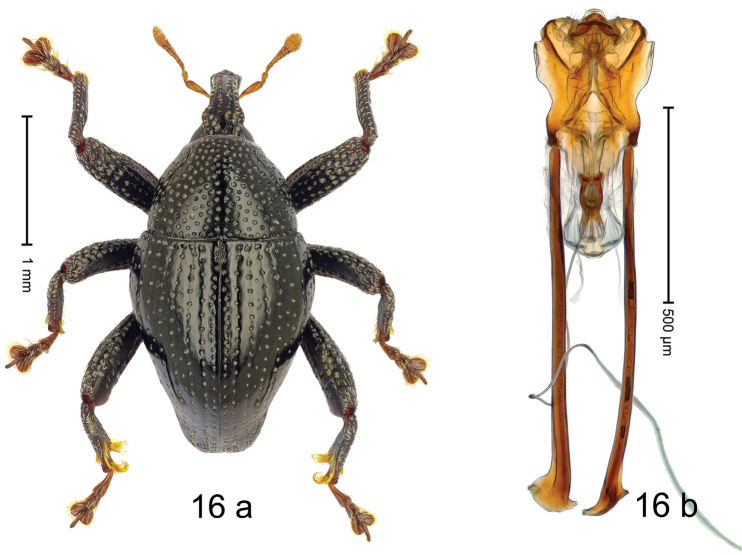
*Trigonopteruscirripes* Riedel, sp. n., holotype; **a** habitus **b** penis.

***Holotype***, male (Fig. 16a). Length 2.54 mm. Color of antennae and tarsi ferruginous; remainder black. Body subovate; in dorsal aspect with weak constriction between pronotum and elytron; in profile dorsally convex. Rostrum dorsally with broad median costa and pair of submedian ridges; intervening furrows each with sparse row of subrecumbent setae; epistome weakly scabrous. Pronotum with disk densely punctate; interspaces between punctures subglabrous, subequal to or smaller than punctures´ diameter. Elytra with striae marked by fine lines and rows of small punctures; basal margin bordered by transverse row of deeper punctures; sutural interval with additional row, other intervals subglabrous, with few interspersed punctures; stria 8 along humerus with five coarse punctures. Femora edentate; anteroventral ridge of mesofemur and metafemur crenate; anterior surface coarsely punctate, each puncture with recumbent narrow scale. Metafemur with dorsoposterior edge denticulate; subapically with stridulatory patch. Metatibia subapically concave, subglabrous, with two dense clusters of setae, near tarsal articulation setae strongly incurved. Abdominal ventrites 1–2 concave, subglabrous; ventrite 5 with apical 1/2 bent ventrad; subapically punctate. Penis (Fig. 16b) with sides of body constricted in basal 1/2, diverging to shallow subapical constriction; ostium at middle with large sclerite; apodemes 2.5 × as long as body of penis; transfer apparatus elongate; ductus ejaculatorius with indistinct bulbus. ***Intraspecific variation***. Length 2.48–2.55 mm. Female rostrum subglabrous, with two submedian rows of punctures, with pair of sublateral furrows. Female abdominal ventrite 5 flat, subapically punctate.

#### Material examined.

***Holotype*** (MZB): ARC2915 (GenBank # MK260283), N-Sulawesi Prov., Kotamobagu, Modoinding, Kakenturan, 00°46.951'N 124°30.427'E to 00°47.053'N 124°30.413'E, 1210–1228 m, beaten, 19-V-2012. ***Paratypes*** (MZB, SMNK): N-Sulawesi Prov.: 13 exx, ARC2916 (EMBL # LN884945), ARC2917 (GenBank # MK260284), ARC2918 (GenBank # MK260285), same data as holotype.

#### Distribution.

N-Sulawesi Prov. (Modoinding). Elevation ca. 1200 m.

#### Biology.

On foliage in montane forests.

#### Etymology.

This epithet is a combination of the Latin nouns *cirrus* (curl) and *pes* (foot, leg). It refers to the male metatibia.

#### Notes.

*Trigonopteruscirripes* Riedel, sp. n. was coded as “*Trigonopterus* sp. 395”.

### 17. Trigonopterus collaris

Taxon classificationAnimaliaColeopteraCurculionidae

Riedel sp. n.

http://zoobank.org/9805629C-D7AA-4AD2-AEB3-E03F1E869421

#### Diagnostic description.

**Figure 17.**
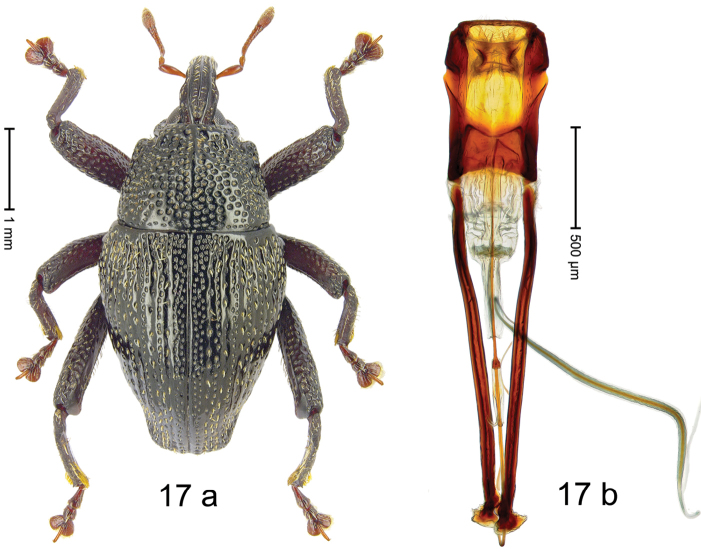
*Trigonopteruscollaris* Riedel, sp. n., holotype; **a** habitus **b** penis.

***Holotype***, male (Fig. 17a). Length 4.16 mm. Color of antennae and tarsi dark ferruginous; remainder black with slightly bronze luster. Body subovate; in dorsal aspect with distinct constriction between pronotum and elytron; in profile dorsally convex. Rostrum dorsally with median and pair of submedian ridges, converging on forehead; intervening furrows each with sparse row of suberect piliform scales; epistome indistinct, sparsely punctate. Pronotum with sides weakly converging in straight line to distinct subapical constriction; disk densely coarsely punctate-reticulate, medially costate; with sparse recumbent narrow scales. Elytra with striae marked by weakly impressed lines and sparse coarse punctures; sutural interval and humerus densely punctate, intervals 2–7 each with single row of punctures; with scattered recumbent narrow scales. Profemur with anteroventral ridge simple, ending with small blunt tooth. Meso- and metafemur with anteroventral ridge crenate, ending with small acute tooth; anterior surface densely punctate, each puncture with recumbent narrow scale. Metafemur with dorsoposterior edge weakly crenate; subapically with stridulatory patch. Metatibia with posterior face subglabrous, with sparse setae, possibly partly abraded. Abdominal ventrites 1–2 medially concave, subglabrous; sublaterally with suberect scales; ventrite 5 flat, coarsely punctate, with dense erect setae. Penis (Fig. 17b) with sides of body subparallel, at middle and subapically with constriction; apex subangulate; sparsely setose; apodemes 2.2 × as long as body; transfer apparatus flagelliform, 1.4 × longer than body of penis; ductus ejaculatorius basally sclerotized, with distinct bulbus. ***Intraspecific variation***. Length 4.08–4.40 mm. Female rostrum slender, in apical 1/2 median and pair of submedian costae dorsally somewhat flattened. Posterior face of metatibia in specimens from Pc. Palopo covered with yellowish setae.

#### Material examined.

***Holotype*** (MZB): ARC3019 (GenBank # MK260247), S-Sulawesi Prov., Tanah Toraja, Rantepao, Gn. Karre (= Gn. Wokim), 02°59.013'S 120°02.251'E, 1423 m, sifted, 06-V-2013. ***Paratypes*** (MZB, SMNK): S-Sulawesi Prov.: 1 ex, ARC3020 (GenBank # MK260242), Tanah Toraja, Rantepao, Gn. Karre (= Gn. Wokim), 02°58.846'S 120°02.158'E, 1396 m, sifted, 06-V-2013; 1 ex, ARC3013 (GenBank # MK260244), Tanah Toraja, Rantepao, Gn. Karre (= Gn. Wokim), 02°59.021'S 120°02.523'E, 1456 m, sifted, 06-V-2013; 3 exx, ARC2836 (GenBank # MK260245), ARC3085 (GenBank # MK260243), ARC3086 (GenBank # MK260246), Pc. Palopo, Gn. Sampuna, 02°56.545'S 120°05.595'E, 1101 m, sifted, 29-V-2012.

#### Distribution.

S-Sulawesi Prov. (Tanah Toraja, Pc. Palopo). Elevation 1100–1420 m.

#### Biology.

In leaf litter of montane forest.

#### Etymology.

This epithet is based on the Latin adjective *collaris* (pertaining to the neck) and refers to the species´ subapically constricted pronotum.

#### Notes.

*Trigonopteruscollaris* Riedel, sp. n. was coded as “*Trigonopterus* sp. 384”. This species is closely related to *T.paracollaris* Riedel, sp. n., from which it can be distinguished by the elytral sculpture and the morphology of the male genitalia.

### 18. Trigonopterus costatulus

Taxon classificationAnimaliaColeopteraCurculionidae

Riedel sp. n.

http://zoobank.org/0414154D-8533-4834-B0CF-CB00D8CD1513

#### Diagnostic description.

**Figure 18.**
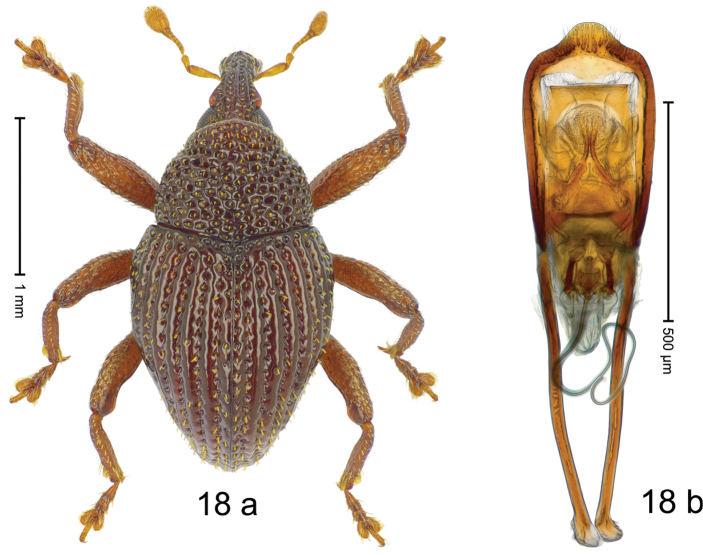
*Trigonopteruscostatulus* Riedel, sp. n., holotype; **a** habitus **b** penis.

***Holotype***, male (Fig. 18a). Length 2.28 mm. Color of antennae and legs ferruginous; remainder dark ferruginous. Body subovate; in dorsal aspect and in profile with weak constriction between pronotum and elytron. Rostrum dorsally with median ridge and pair of submedian ridges; intervening furrows with sparse rows of suberect setae; epistome posteriorly with transverse, angulate ridge. Pronotum with weak subapical constriction; disk coarsely punctate-reticulate; each puncture with suberect, weakly spatulate scale. Elytra with striae deeply impressed, containing rows of punctures each with short, suberect scale; intervals costate, subglabrous; sutural interval raised, with additional row of punctures; apex produced ventrad. Metathoracic spiracle located on laterally projecting denticle. Femora edentate. Metafemur subapically with stridulatory patch. Mesotibia apically confluent with enlarged, curved uncus. Abdominal ventrites 1–2 medially deeply impressed, with dense coarse punctures; ventrite 5 medially with distinct subglabrous depression, laterally coarsely punctate. Penis (Fig. 18b) with sides of body weakly diverging; apex subangulate, sparsely setose; apodemes 1.6 × as long as body of penis; transfer apparatus complex; ductus ejaculatorius with apical part broken and missing. ***Intraspecific variation***. Length 2.22–2.60 mm. Female rostrum slender, dorsally with ridges more rounded; epistome indistinct. Female elytral apex ventrally concave, with sutural interval in profile forming subangulate projection. Female abdominal ventrite 5 with shallow median depression, evenly coarsely punctate.

#### Material examined.

***Holotype*** (MZB): ARC3075 (GenBank # MK260537), S-Sulawesi Prov., Tanah Toraja, Ponding, Baruppu, 02°48.010'S 119°47.560'E, 2199 m, sifted, 04-V-2013. ***Paratypes*** (ARC, MZB, SMNK): S-Sulawesi Prov., Tanah Toraja: 7 exx, ARC3073 (GenBank # MK260536), ARC3074 (GenBank # MK260535), same data as holotype; 6 exx, ARC3068 (EMBL # LN884956), ARC3069 (GenBank # MK260534), Ponding, Baruppu, 02°48.339'S 119°47.880'E, 2113 m, sifted, 04-V-2013; 15 exx, Ponding, Baruppu, 02°49.662'S 119°48.871'E, 2173 m, sifted, 05-V-2013; 23 exx, Ponding, Baruppu, 02°47.918'S 119°47.408'E, 2233 m, sifted, 04-V-2013; 4 exx, Tanah Toraja, Pulu Pulu, 2400 m, sifted, 15–16-VIII-1990.

#### Distribution.

S-Sulawesi Prov. (Tanah Toraja). Elevation 2100–2400 m.

#### Biology.

In leaf litter of montane forest.

#### Etymology.

This epithet is based on the diminutive form of the Latin adjective *costatus*, -*a*, -*um* (ribbed) and refers to the species´ small size and its elytral sculpture.

#### Notes.

*Trigonopteruscostatulus* Riedel, sp. n. was coded as “*Trigonopterus* sp. 511”.

### 19. Trigonopterus curvipes

Taxon classificationAnimaliaColeopteraCurculionidae

Riedel sp. n.

http://zoobank.org/5CE695CE-624D-4AAA-82CF-5D72400A9956

#### Diagnostic description.

**Figure 19.**
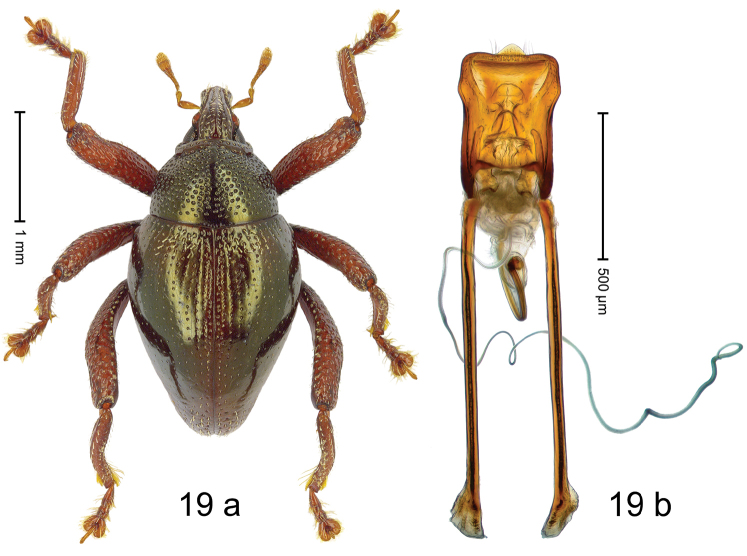
*Trigonopteruscurvipes* Riedel, sp. n., holotype; **a** habitus **b** penis.

***Holotype***, male (Fig. 19a). Length 2.75 mm. Color of antennae and legs ferruginous; remainder black with bronze luster. Body subovate; in dorsal aspect with weak constriction between pronotum and elytron; in profile dorsally flat, convex to apex. Rostrum dorsally with median carina and pair of submedian carinae; intervening furrows with sparse rows of suberect yellowish scales; epistome short, indistinct; base of rostrum swollen, in profile dorsally convex, arching over forehead. Pronotum laterally with distinct subapical constriction; disk densely punctate; interspaces subglabrous, reticulate; with subglabrous median ridge. Elytra densely punctate with small punctures; striae indistinct; intervals with rows of small punctures; subbasally and subapically with sparse yellowish scales. Hind legs long. Femora edentate; anteroventral ridge distinct, weakly crenate; anterior surface punctate-reticulate, each puncture with narrow scale. Metafemur dorsally weakly denticulate, with row of erect clavate scales; subapically with stridulatory patch. Metatibia curved, subapically with few long setae. Thoracic venter and abdominal ventrites 1–2 sparsely setose with long erect setae. Abdominal ventrite 5 at middle with subglabrous concavity, basal margin at middle with short extension. Penis (Fig. 19b) with sides of body in basal 1/2 subparallel, subapically widened; apex sparsely setose, with median, subtriangular extension; apodemes 2.4 × as long as body of penis; transfer apparatus flagelliform, ca. 1.5 × length of body of penis; ductus ejaculatorius with indistinct bulbus. ***Intraspecific variation***. Length 2.60–2.75 mm. Female rostrum dorsally flattened, with ridges less distinct. Female thoracic and abdominal venter subglabrous. Female abdominal ventrite 5 flat.

#### Material examined.

***Holotype*** (MZB): ARC3227 (GenBank # MK260432), S-Sulawesi Prov., Mangkutana, 02°20.203'S 120°46.878'E, 903 m, sifted, 14-V-2013. ***Paratypes*** (SMNK): 2 exx, ARC3225 (GenBank # MK260431), ARC3226 (GenBank # MK260433), same data as holotype. Other material examined: 1 ex, ARC3209 (EMBL # LN884975), C-Sulawesi Prov., Pendolo, Gn. Sampuraga, 02°12.165'S 120°45.567'E, 934 m, sifted, 13-V-2013.

#### Distribution.

S-/C-Sulawesi Prov. (Mangkutana, Pendolo). Elevation 900–930 m.

#### Biology.

In leaf litter of montane forest.

#### Etymology.

This epithet is a combination of the Latin adjective *curvus*, -*a*, -*um* (curved, bent) and the noun pes (foot, leg). It refers to the curved metatibia. A noun in apposition.

#### Notes.

*Trigonopteruscurvipes* Riedel, sp. n. was coded as “*Trigonopterus* sp. 480”. The form “480-A” represented by a female specimen from Pendolo differs ca. 11% p-distance of *cox1* and may represent a separate species.

### 20. Trigonopterus crenulatus

Taxon classificationAnimaliaColeopteraCurculionidae

Riedel sp. n.

http://zoobank.org/81656BD8-D6C3-4B63-A110-0A06F3E4BEDF

#### Diagnostic description.

**Figure 20.**
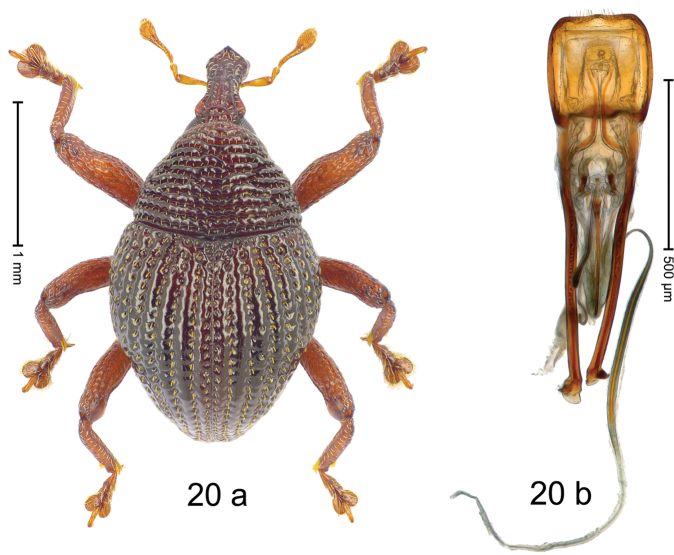
*Trigonopteruscrenulatus* Riedel, sp. n., holotype; **a** habitus **b** penis.

***Holotype***, male (Fig. 20a). Length 2.30 mm. Color of antennae and legs ferruginous, remainder dark ferruginous. Body subovate; in profile with distinct constriction between pronotum and elytron. Rostrum dorsally with median and pair of submedian ridges; intervening furrows with rows of punctures and sparse setae; epistome short, posteriorly with transverse, subangulate ridge extended into median denticle. Pronotum with very weak subapical constriction; disk with transverse, crenulate costae separated by deep furrows; costae with rows of anteriad directed setae. Elytra with striae deeply impressed, with dense rows of punctures; punctures each with narrow, suberect scale; intervals costate, subglabrous. Metathoracic spiracle located on laterally projecting denticle. Femora with anterior surface punctate-rugose. Profemur edentate. Mesofemur and metafemur with small tooth; metafemur subapically with stridulatory patch. Abdominal ventrite 1 concave, microreticulate, with coarse punctures; ventrite 5 at middle with distinct depression, coarsely punctate. Penis (Fig. 20b) with sides of body subparallel; apex subtruncate, with sparse short setae; basal orifice with transverse brace; apodemes 2.8 × as long as body of penis; endophallus with pair of elongate sclerites; transfer apparatus flagelliform, 2.0 × longer than body; ductus ejaculatorius with distinct bulbus. ***Intraspecific variation***. Length 2.05–2.30 mm. Female rostrum dorsally subglabrous, punctate; epistome indistinct. Female abdominal ventrite 5 flat, coarsely punctate.

#### Material examined.

***Holotype*** (MZB): ARC3009 (GenBank # MK260552), S-Sulawesi Prov., Rantepao, Gn. Karre (= Gn. Wokim), 02°59.021'S 120°02.523'E, 1456 m, sifted, 06-V-2013. ***Paratypes*** (MZB, SMNK): 69 exx, ARC3010 (GenBank # MK260555), ARC3011 (GenBank # MK260554), ARC3012 (GenBank # MK260553), same data as holotype.

#### Distribution.

S-Sulawesi Prov. (Tanah Toraja). Elevation ca. 1460 m.

#### Biology.

In leaf litter of montane forest.

#### Etymology.

This epithet is the Latin adjective *crenulatus*, -*a*, -*um* (with small notches, rounded projections) and refers to the sculpture of the pronotum.

#### Notes.

*Trigonopteruscrenulatus* Riedel, sp. n. was coded as “*Trigonopterus* sp. 520”.

### 21. Trigonopterus cricki

Taxon classificationAnimaliaColeopteraCurculionidae

Riedel sp. n.

http://zoobank.org/237FC65A-7B34-4BD8-AB59-60A05F6FF0E6

#### Diagnostic description.

**Figure 21.**
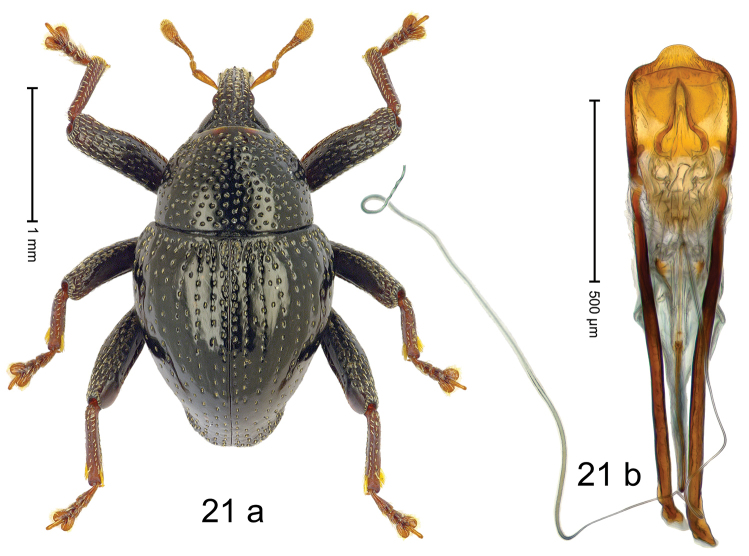
*Trigonopteruscricki* Riedel, sp. n., holotype; **a** habitus **b** penis.

***Holotype***, male (Fig. 21a). Length 2.38 mm. Color of antennae, tibiae and tarsi ferruginous; remainder black. Body subovate; in dorsal aspect and in profile with weak constriction between pronotum and elytron. Rostrum dorsally with median and pair of submedian ridges; intervening furrows with sparse rows of piliform scales; epistome short, posteriorly with five denticles. Pronotum with disk densely punctate with small punctures; each puncture containing fine, recumbent seta; interspaces between punctures subglabrous. Elytra with striae marked by rows of small punctures; basal margin costate; sutural interval subbasally swollen, coarsely punctate; other intervals subglabrous; humerus with irregular, coarse punctures. Femora edentate; anteroventral ridge distinct, weakly crenate; anterior surface densely coarsely punctate, each puncture with recumbent piliform scale. Metafemur subapically with stridulatory patch. Abdominal ventrites 1–2 concave, microreticulate, subglabrous; ventrite 5 at middle with subtrapezoid impression, microreticulate, punctate. Penis (Fig. 21b) with subangulate extension, medially weakly rounded, without setae; apodemes 3.0 × as long as body of penis; transfer apparatus flagelliform, 2.5 × longer than body of penis; ductus ejaculatorius with indistinct bulbus. ***Intraspecific variation***. Length 2.38–2.40 mm. Female rostrum more slender; dorsally in apical 1/2 subglabrous, with two submedian rows of punctures, with pair of sublateral furrows. Female abdominal ventrite 5 flat, coarsely punctate.

#### Material examined.

***Holotype*** (MZB): ARC3036 (EMBL # LN884951), S-Sulawesi Prov., Tanah Toraja, Bittuang, Gn. Ponding, 02°56.446'S 119°38.075'E, 1625 m, beaten, 09-V-2013. ***Paratypes*** (SMNK): 2 exx, ARC3039 (GenBank # MK260502), ARC6032 (GenBank # MK260503), same data as holotype.

#### Distribution.

S-Sulawesi Prov. (Tanah Toraja). Elevation ca. 1625 m.

#### Biology.

On foliage in montane forests.

#### Etymology.

This species is named in honor of Nobel laureate Francis HC Crick for his contribution in the discovery of the structure of DNA. An invariable genitive.

#### Notes.

*Trigonopteruscricki* Riedel, sp. n. was coded as “*Trigonopterus* sp. 499”.

### 22. Trigonopterus darwini

Taxon classificationAnimaliaColeopteraCurculionidae

Riedel sp. n.

http://zoobank.org/2DE322F0-B934-4A86-A3FE-204575336118

#### Diagnostic description.

**Figure 22.**
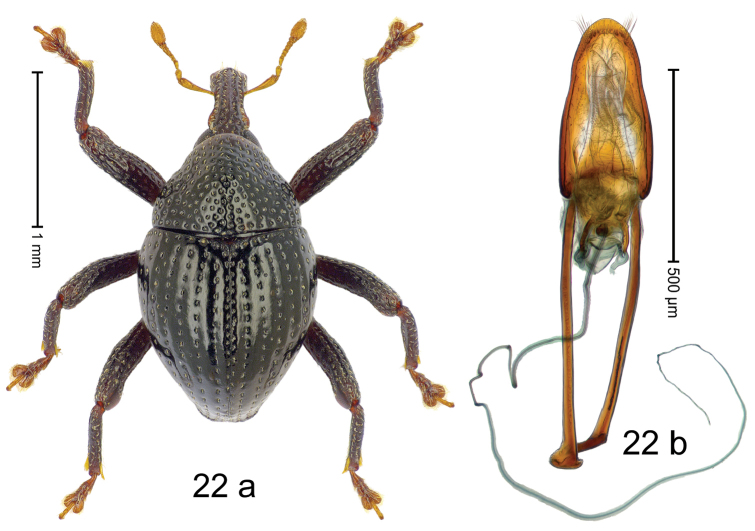
*Trigonopterusdarwini* Riedel, sp. n., holotype; **a** habitus **b** penis.

***Holotype***, male (Fig. 22a). Length 1.90 mm. Color of antennae yellowish; tarsi ferruginous; remainder black. Body subovate; in dorsal aspect with weak constriction, in profile with marked constriction between pronotum and elytron. Rostrum dorsally with median costa and pair of submedian ridges; intervening furrows with rows of punctures containing each one indistinct seta; epistome simple, subglabrous. Pronotum with disk dorsally swollen, densely punctate; interspaces between punctures subglabrous. Elytra with striae marked by dense rows of deep punctures; sutural interval with row of small punctures, other intervals subglabrous, weakly costate; basal margin bordered by transverse row of punctures. Profemur with anteroventral ridge simple; meso- and metafemur with minute denticle in apical 1/2; anterior surface of femora punctate, wrinkled, microreticulate. Metafemur subapically with stridulatory patch. Abdominal ventrite 1 microreticulate, concave, with coarse punctures; ventrite 5 microreticulate, with shallow concavity, sparsely punctate. Penis (Fig. 22b) with sides converging; apex rounded, sublaterally with sparse fringe of setae; apodemes 1.4 × as long as body of penis; transfer apparatus dentiform, directed basad in repose; ductus ejaculatorius with indistinct bulbus. ***Intraspecific variation***. Length 1.90–2.28 mm. Female rostrum more slender; dorsally in apical 1/2 subglabrous, with lateral and sublateral rows of punctures. Female abdominal ventrite 5 microreticulate, sparsely punctate, with sparse suberect setae.

#### Material examined.

***Holotype*** (MZB): ARC2860 (GenBank # MK260334), C-Sulawesi Prov., Pendolo, Gn. Sampuraga, 02°12.476'S 120°45.506'E, 1050 m, sifted, 31-V-2012. ***Paratypes*** (MZB, SMNK): C-Sulawesi Prov.: 4 exx, ARC2861 (GenBank # MK260335), ARC6067 (GenBank # MK260337), ARC6068 (GenBank # MK260336), same data as holotype; 1 ex, ARC3210 (GenBank # MK260333), Pendolo, Gn. Sampuraga, 02°12.308'S 120°45.544'E, 1011 m, sifted, 13-V-2013.

#### Distribution.

C-Sulawesi Prov. (Pendolo). Elevation 1010–1050 m.

#### Biology.

In leaf litter of montane forest.

#### Etymology.

This species is named in honor of Charles Darwin for providing an evolutionary foundation to the science of taxonomy. An invariable genitive.

#### Notes.

*Trigonopterusdarwini* Riedel, sp. n. was coded as “*Trigonopterus* sp. 407”.

### 23. Trigonopterus ejaculatorius

Taxon classificationAnimaliaColeopteraCurculionidae

Riedel sp. n.

http://zoobank.org/B0620ADD-D4B0-418E-8545-CD6A9416E712

#### Diagnostic description.

**Figure 23.**
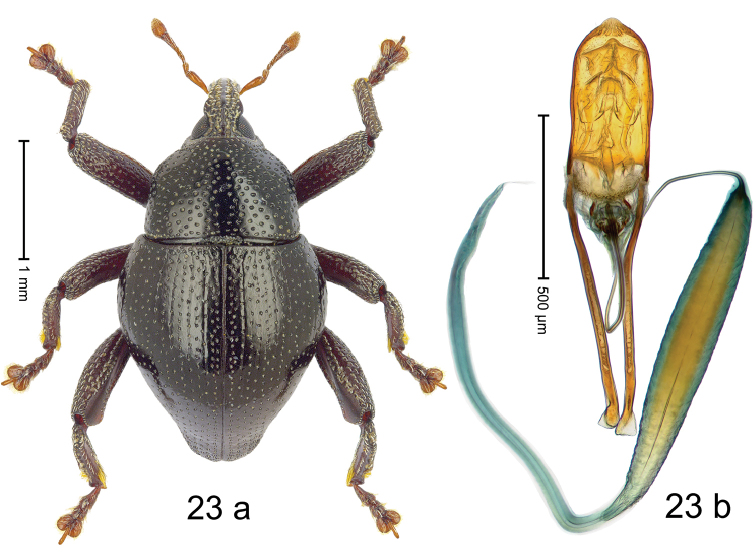
*Trigonopterusejaculatorius* Riedel, sp. n., holotype; **a** habitus **b** penis.

***Holotype***, male (Fig. 23a). Length 2.75 mm. Color of antennae ferruginous; legs dark ferruginous; remainder black. Body subovate; in dorsal aspect with distinct constriction between pronotum and elytron, in profile dorsally convex. Rostrum dorsally with median costa and pair of submedian ridges; intervening furrows with rows of silvery elongate scales; in profile median costa forming subangular hump at junction with forehead; epistome indistinct. Pronotum with disk densely punctate; interspaces between punctures subglabrous; median line impunctate. Elytra densely irregularly punctate with small punctures; striae marked by hardly visible hairlines; intervals flat; stria 7 and 8 along humerus with 5–6 coarse punctures. Femora edentate. Meso- and metafemur with anteroventral ridge crenate; anterior surface of femora densely punctate, each puncture with narrow recumbent scale. Metafemur with dorsoposterior edge weakly crenate; subapically with stridulatory patch. Metatibia with dorsal edge in basal 1/2 denticulate. Abdominal ventrites 1–2 weakly concave, subglabrous, laterally microreticulate, with sparse erect scales; ventrite 5 at middle impressed, concave, subglabrous, sublaterally and subapically densely punctate. Penis (Fig. 23b) with sides subparallel; apex subangulate, weakly pointed, without setae; apodemes 2.0 × as long as body of penis; transfer apparatus spiniform, directed basad in repose; ductus ejaculatorius with distinct, swollen bulbus.

#### Material examined.

***Holotype*** (MZB): ARC3094 (GenBank # MK260527), S-Sulawesi Prov., Mangkutana, 02°20.306'S 120°46.792'E to 02°20.203'S 120°46.878'E, 850–910 m, beaten, 14-V-2013.

#### Distribution.

S-Sulawesi Prov. (Mangkutana). Elevation ca. 850–910 m.

#### Biology.

On foliage in montane forests.

#### Etymology.

This epithet is based on the ductus ejaculatorius which is markedly swollen in males of this species. It is treated as an adjective.

#### Notes.

*Trigonopterusejaculatorius* Riedel, sp. n. was coded as “*Trigonopterus* sp. 507”. It is closely related to *T.vicinus* Riedel, sp. n. from which it differs by 17.3% p-distance of *cox1* and can be distinguished by the subangular basal hump of the rostral profile.

### 24. Trigonopterus fuscipes

Taxon classificationAnimaliaColeopteraCurculionidae

Riedel sp. n.

http://zoobank.org/E56071FB-B7A5-433C-AA59-2E7AEC15D999

#### Diagnostic description.

**Figure 24.**
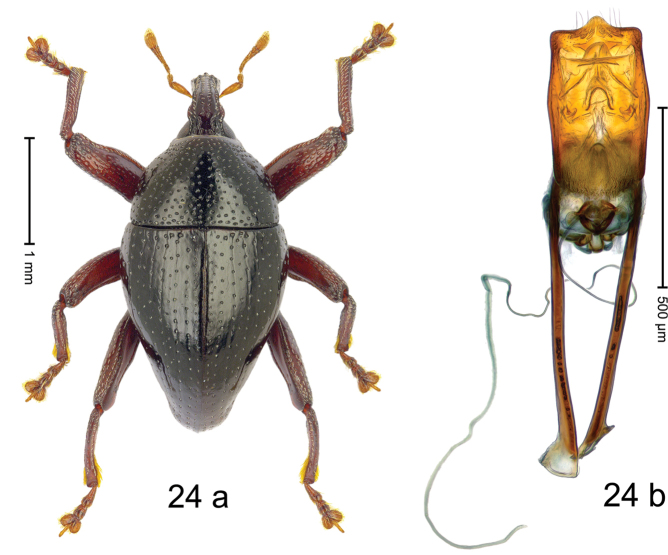
*Trigonopterusfuscipes* Riedel, sp. n., holotype; **a** habitus **b** penis.

***Holotype***, male (Fig. 24a). Length 2.75 mm. Color of antennae and legs ferruginous; remainder black. Body subovate; in dorsal aspect with weak constriction between pronotum and elytron; in profile dorsally convex. Rostrum dorsally with median and pair of submedian ridges; intervening furrows each with sparse row of recumbent piliform scales; epistome with one median denticle and two less distinct pairs of submedian / sublateral denticles. Pronotum with disk punctate with small punctures; interspaces subglabrous. Elytra punctate with small punctures; striae marked by fine hairlines; irregular intervals each with additional sparse row of punctures; interspaces between punctures subglabrous; stria 8 and 9 along humerus with somewhat larger punctures. Femora edentate; anterior surface punctate with small punctures, each with recumbent piliform scale. Metafemur subapically with stridulatory patch. Protibia widened to apex. Posterior face of metatibia in apical 1/2 with dense yellowish setae. Abdominal ventrites 1–2 concave, subglabrous; ventrite 5 with deep, subglabrous pit, subapically punctate. Penis (Fig. 24b) with sides of body subparallel; apex with median subangulate extension, sparsely setose; apodemes 2.5 × as long as body; transfer apparatus complex, with anchor-shaped basal sclerite; ductus ejaculatorius with indistinct bulbus. ***Intraspecific variation***. Length 2.70–2.75 mm. Female rostrum slender, with median row of punctures and pair of sublateral furrows; epistome simple. Female metatibia only subapically setose.

#### Material examined.

***Holotype*** (MZB): ARC2994 (GenBank # MK260571), S-Sulawesi Prov., Tanah Toraja, Rantepao, Gn. Karre (= Gn. Wokim), 02°59.021'S 120°02.523'E, 1456 m, beaten, 06-V-2013. ***Paratypes*** (MZB, SMNK): S-Sulawesi Prov., Tanah Toraja, Rantepao, Gn. Karre (= Gn. Wokim): 24 exx, ARC2995 (GenBank # MK260570), ARC5987 (GenBank # MK260575), ARC5989 (GenBank # MK260574), ARC5991 (GenBank # MK260572), same data as holotype; 1 ex, ARC6026 (GenBank # MK260569), 02°59.021'S 120°02.523'E, 1456 m, sifted, 06-V-2013; 18 exx, ARC6002 (GenBank # MK260577), ARC6004 (GenBank # MK260573), 02°59.013'S 120°02.251'E, 1423 m, beaten, 06-V-2013; 20 exx, ARC6012 (GenBank # MK260578), ARC6013 (GenBank # MK260576), 02°58.846'S 120°02.158'E, 1396 m, beaten, 06-V-2013.

#### Distribution.

S-Sulawesi Prov. (Tanah Toraja). Elevation 1400–1460 m.

#### Biology.

On foliage in montane forests.

#### Etymology.

This epithet is a combination of the Latin adjective *fuscus*, -*a*, -*um* (dark, tawny) and the noun *pes* (foot, leg). A noun in apposition.

#### Notes.

*Trigonopterusfuscipes* Riedel, sp. n. was coded as “*Trigonopterus* sp. 526”. This species is very closely related to *T.asterix* Riedel, sp. n., from which it differs by ca. 12% p-distance of *cox1* and by the structure of the male genitalia.

### 25. Trigonopterus gracilipes

Taxon classificationAnimaliaColeopteraCurculionidae

Riedel sp. n.

http://zoobank.org/0EC4E275-EF1A-4987-9F05-242EB6D6FD2D

#### Diagnostic description.

**Figure 25.**
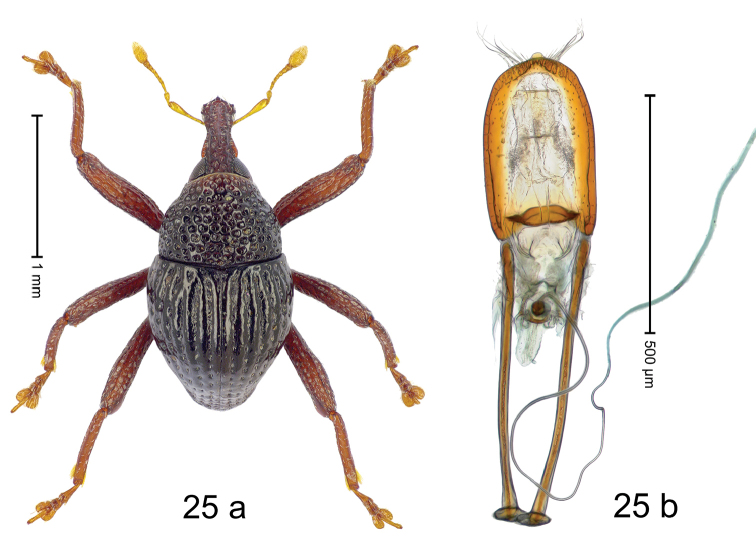
*Trigonopterusgracilipes* Riedel, sp. n., holotype; **a** habitus **b** penis.

***Holotype***, male (Fig. 25a). Length 1.82 mm. Color of antennae yellowish; legs ferruginous; remainder black. Body subovate; in dorsal aspect with weak constriction, in profile with distinct constriction between pronotum and elytron. Rostrum long; at middle with gentle constriction; dorsally with median and pair of submedian, somewhat irregular ridges; intervening furrows with punctures and sparse indistinct setae; epistome posteriorly with angulate ridge bearing median and pair of sublateral denticles. Pronotum with disk densely, coarsely punctate; interspaces between punctures reticulate, subglabrous. Elytra with punctures large, deeply impressed; dorsally intervals dorsally costate, subglabrous; sutural interval basally curving laterad. Legs long, almost spider-like. Femora with very indistinct anteroventral ridge ending with minute denticle; anterior and dorsal surface punctate-rugose, with longitudinal wrinkles. Metafemur subapically with stridulatory patch. Abdominal ventrites 1–2 dull, microreticulate, with coarse punctures; ventrite 5 flat, microreticulate, dull. Penis (Fig. 25b) with body curved ventrad, in basal 1/2 sides subparallel, converging to acute apex; subapically with fringe of long setae; basal orifice with well-sclerotized ventral rim; apodemes 2.1 × as long as body of penis; transfer apparatus indistinct; ductus ejaculatorius with indistinct bulbus.

#### Material examined.

***Holotype*** (MZB): ARC2881 (GenBank # MK260366), N-Sulawesi Prov., Kotamobagu, Matalibaru, 00°32.445'N 124°14.185'E, 956 m, beaten, 20-V-2012.

#### Distribution.

N-Sulawesi Prov. (Kotamobagu). Elevation ca. 960 m.

#### Biology.

On foliage in montane forests.

#### Etymology.

This epithet is a combination of the Latin adjective *gracilis* (slender) and the noun pes (foot, leg). It refers to the species´ long, slender legs. A noun in apposition.

#### Notes.

*Trigonopterusgracilipes* Riedel, sp. n. was coded as “*Trigonopterus* sp. 414”.

### 26. Trigonopterus heberti

Taxon classificationAnimaliaColeopteraCurculionidae

Riedel sp. n.

http://zoobank.org/6572C721-CBCB-4D69-BB4C-A8B68FA1E98F

#### Diagnostic description.

**Figure 26.**
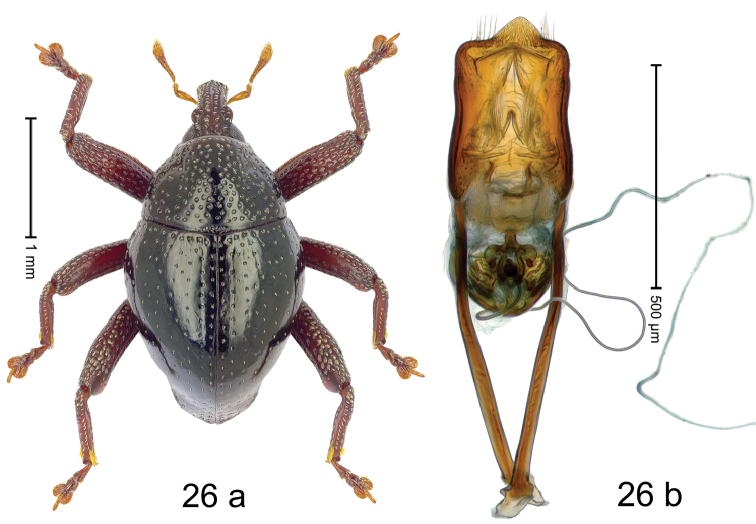
*Trigonopterusheberti* Riedel, sp. n., holotype; **a** habitus **b** penis.

***Holotype***, male (Fig. 26a). Length 2.46 mm. Color of antennae and legs ferruginous; remainder black. Body subovate; in dorsal aspect with weak constriction between pronotum and elytron, in profile dorsally convex. Rostrum dorsally with median and pair of submedian ridges; intervening furrows with sparse rows of suberect setae; epistome short, posteriorly with transverse, angulate ridge. Pronotum with median, densely punctate swelling; submedially disk with pair of broad, sparsely punctate depressions; interspaces between punctures subglabrous. Elytra with striae marked by rows of minute punctures; basal margin bordered by transverse row of deeper punctures; stria 8 along humerus with six large, coarse punctures; sutural interval with additional row, other intervals subglabrous, with few interspersed punctures; elytral apex subangulate. Femora edentate; anterior surface densely coarsely punctate, each puncture with recumbent scale. Metafemur with dorsoposterior edge denticulate, with silvery-yellowish scales upcurved; subapically with stridulatory patch. Abdominal ventrite 1 and anterior portion of ventrite 2 concave, cavernous, subglabrous; ventrite 2 posteriorly forming rim; ventrite 5 broadly concave, sparsely punctate. Penis (Fig. 26b) subapically with fringe of sparse setae, medially with angulate extension; dorsum with submedian brushes of setae; apodemes 1.7 × as long as body of penis; transfer apparatus with complex sclerites; ductus ejaculatorius without bulbus. ***Intraspecific variation***. Length 2.26–2.46 mm. Female rostrum in apical 1/2 slender, subglabrous, with two submedian rows of punctures, with pair of sublateral furrows. Female abdominal ventrite 5 flat, sparsely punctate, setose.

#### Material examined.

***Holotype*** (MZB): ARC2885 (GenBank # MK2 60352), N-Sulawesi Prov., Kotamobagu, Matalibaru, 00°32.445'N 124°14.185'E, 956 m, beaten, 20-V-2012. ***Paratypes*** (MZB, SMNK): N-Sulawesi Prov.: 1 ex, ARC2887 (GenBank # MK260348), same data as holotype; 3 exx, ARC2903 (GenBank # MK260349), ARC2904 (GenBank # MK260350), ARC2910 (GenBank # MK260353), Kotamobagu, Modoinding, Lake Moat area, 00°42.862'N 124°28.356'E, 1024 m, sifted, 19-V-2012; 1 ex, ARC2756 (GenBank # MK260351), Tomohon, Rurukan, Gn. Mahawu, 01°21.409'N 124°51.535'E to 01°21.370'N 124°51.676'E, 1126–1231 m, 14-V-2012.

#### Distribution.

N-Sulawesi Prov. (Kotamobagu, Lake Moat, Tomohon). Elevation 960–1130 m.

#### Biology.

On foliage in montane forests.

#### Etymology.

This species is named in honor of Prof. Paul DN Hebert (University of Guelph) for establishing “DNA barcoding” as a tool in taxonomic practice. An invariable genitive.

#### Notes.

*Trigonopterusheberti* Riedel, sp. n. was coded as “*Trigonopterus* sp. 411”.

### 27. Trigonopterus hirsutus

Taxon classificationAnimaliaColeopteraCurculionidae

Riedel sp. n.

http://zoobank.org/5C69DEE2-52CE-4576-BAFE-BC7CB937662E

#### Diagnostic description.

**Figure 27.**
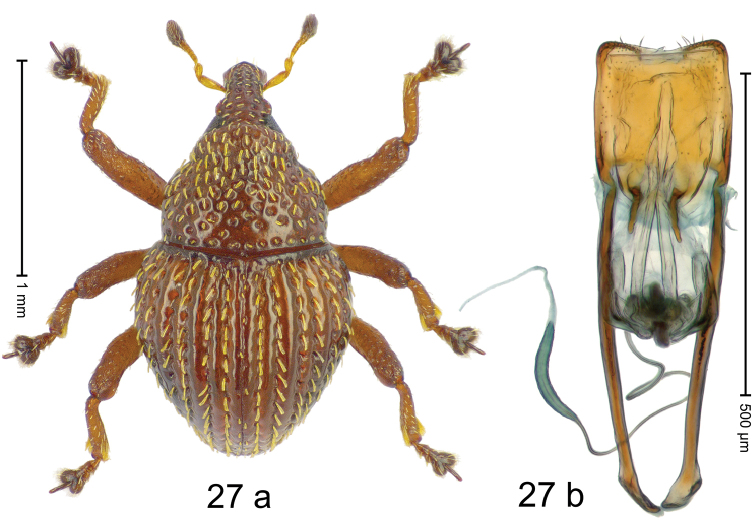
*Trigonopterushirsutus* Riedel, sp. n., holotype; **a** habitus **b** penis.

***Holotype***, male (Fig. 27a). Length 1.56 mm. Color ferruginous, tarsi and antennal club darker. Body subovate; in dorsal aspect with distinct constriction between pronotum and elytron; in profile dorsally flat, convex to apex. Rostrum dorsally with median and pair of submedian ridges; intervening furrows with rows of coarse punctures and sparse setae; epistome short, posteriorly with transverse, subangulate ridge. Pronotum with weak subapical constriction; disk densely punctate with coarse punctures; each puncture with spatulate, anteriad directed, yellowish scale. Elytra with striae impressed, marked by rows of coarse punctures; punctures each with spatulate, yellowish scale; intervals costate, subglabrous. Metathoracic spiracle located on laterally projecting denticle. Femora edentate; surface dull, microreticulate. Metafemur subapically with stridulatory patch. Abdominal ventrites 1–2 concave, microreticulate, punctate; ventrite 5 flat, coarsely densely punctate, dull. Penis (Fig. 27b) with sides of body subparallel; apex subtruncate, with few short setae, medially membranous; apodemes 2.4 × as long as body of penis; endophallus with pair of elongate sclerites; transfer apparatus short flagelliform, subequal to body of penis; ductus ejaculatorius with distinct bulbus. ***Intraspecific variation***. Length 1.40–1.63 mm. Female rostrum dorsally subglabrous, punctate; epistome indistinct. Female abdominal ventrite 5 flat, coarsely punctate.

#### Material examined.

***Holotype*** (MZB): ARC3060 (GenBank # MK260447), S-Sulawesi Prov., Tanah Toraja, Bittuang, Gn. Karoa, 02°55.032'S 119°40.365'E, 1992 m, sifted, 10-V-2013. ***Paratypes*** (ARC, MZB, SMNK): S-Sulawesi, Tanah Toraja: 2 exx, ARC3061 (GenBank # MK260446), ARC3062 (GenBank # MK260448), same data as holotype; 10 exx, ARC3076 (GenBank # MK260450), ARC3077 (GenBank # MK260451), Ponding, Baruppu, 02°48.010'S 119°47.560'E, 2199 m, sifted, 04-V-2013; 6 exx, Tanah Toraja, Ponding, Baruppu, 02°48.597'S 119°47.611'E, 1963 m, sifted, 03-V-2013; 42 exx, ARC3081 (GenBank # MK260452), ARC3082 (GenBank # MK260453), ARC3083 (GenBank # MK260454), ARC3084 (GenBank # MK260455), Ponding, Baruppu, 02°49.748'S 119°48.995'E, 2139 m, 05-V-2013; 7 exx, Tanah Toraja, Ponding, Baruppu, 02°48.339'S 119°47.880'E, 2113 m, sifted, 04-V-2013; 11 exx, Ponding, Baruppu, 02°49.662'S 119°48.871'E, 2173 m, sifted, 05-V-2013; 24 exx, Ponding, Baruppu, 02°47.918'S 119°47.408'E, 2233 m, sifted, 04-V-2013; 13 exx, Tanah Toraja, Pulu Pulu, 2400 m, sifted, 15–16-VIII-1990; 8 exx, Tanah Toraja, Pulu Pulu, 2400 m, sifted, 13–16-VIII-1990; 1 ex, ARC3018 (GenBank # MK260449), Rantepao, Gn. Karre (= Gn. Wokim), 02°59.021'S 120°02.523'E, 1456 m, sifted, 06-V-2013.

#### Distribution.

S-Sulawesi Prov. (Tanah Toraja). Elevation 1460–2400 m.

#### Biology.

In leaf litter of montane forest.

#### Etymology.

This epithet is the Latin adjective *hirsutus*, -*a*, -*um* (rough, bristly) and refers to the species´ elytral vestiture.

#### Notes.

*Trigonopterushirsutus* Riedel, sp. n. was coded as “*Trigonopterus* sp. 485”. Most of the specimens examined are females (2 males / 74 females); the reason for this unequal proportion is unknown.

### 28. Trigonopterus humilis

Taxon classificationAnimaliaColeopteraCurculionidae

Riedel sp. n.

http://zoobank.org/34BACAC4-74ED-4490-A117-4BEE925C13D5

#### Diagnostic description.

**Figure 28.**
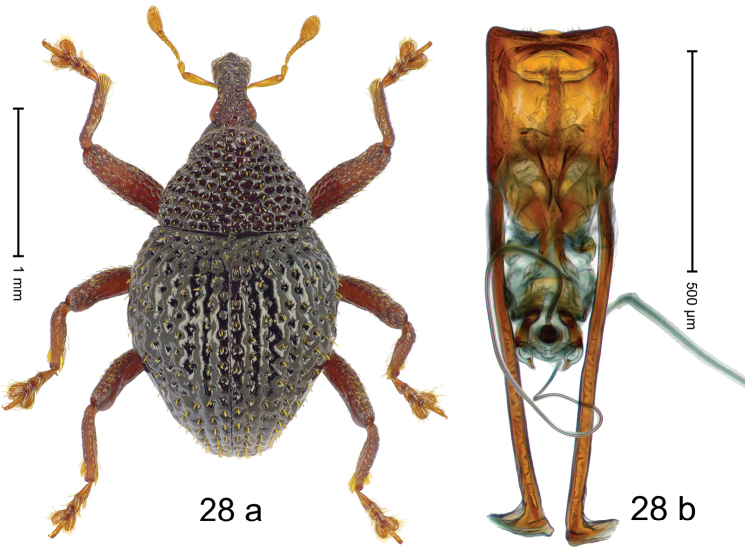
*Trigonopterushumilis* Riedel, sp. n., holotype; **a** habitus **b** penis.

***Holotype***, male (Fig. 28a). Length 2.30 mm. Color of antennae and legs ferruginous; remainder black. Body subovate; in dorsal aspect and in profile with distinct constriction between pronotum and elytron. Rostrum at middle with constriction, apical 1/2 slightly widening, spatulate; dorsally with median ridge and pair of submedian ridges; intervening furrows with rows of punctures and sparse setae; epistome short, posteriorly with transverse, subangulate ridge. Pronotum with weak subapical constriction; disk coarsely punctate, interspaces reticulate-scabrous; each puncture with erect piliform scale. Elytra markedly convex; coarsely punctate; interspaces somewhat irregularly tuberculate-costate, subglabrous; punctures each with suberect piliform scale. Metathoracic spiracle located on laterally projecting denticle. Femora edentate; anterior surface dull, weakly punctate-rugose, with sparse piliform scales. Metafemur with anteroventral ridge ending with indistinct blunt tooth; subapically with stridulatory patch. Abdominal ventrite 1 flat, with transverse row of coarse punctures; ventrite 5 with transverse depression, punctate. Penis (Fig. 28b) with sides subparallel, apex subangulate, with sparse setae; in apical 1/3 ventral surface with transverse blade-like process; apodemes 2.5 × as long as body of penis; transfer apparatus complex, symmetrical; ductus ejaculatorius with indistinct bulbus.

#### Material examined.

***Holotype*** (MZB): ARC3059 (GenBank # MK260558), S-Sulawesi Prov., Tanah Toraja, Bittuang, Gn. Karoa, 02°55.032'S 119°40.365'E, 1992 m, sifted, 10-V-2013.

#### Distribution.

S-Sulawesi Prov. (Tanah Toraja). Elevation ca. 2000 m.

#### Biology.

In leaf litter of montane forest.

#### Etymology.

This epithet is the Latin adjective *humilis*, -*e* (close to the ground, small) and refers to the species´ small size and edaphic habitat.

#### Notes.

*Trigonopterushumilis* Riedel, sp. n. was coded as “*Trigonopterus* sp. 522”.

### 29. Trigonopterus hypocrita

Taxon classificationAnimaliaColeopteraCurculionidae

Riedel sp. n.

http://zoobank.org/3D38A342-94DD-425E-88E9-27AD1B4A2237

#### Diagnostic description.

**Figure 29.**
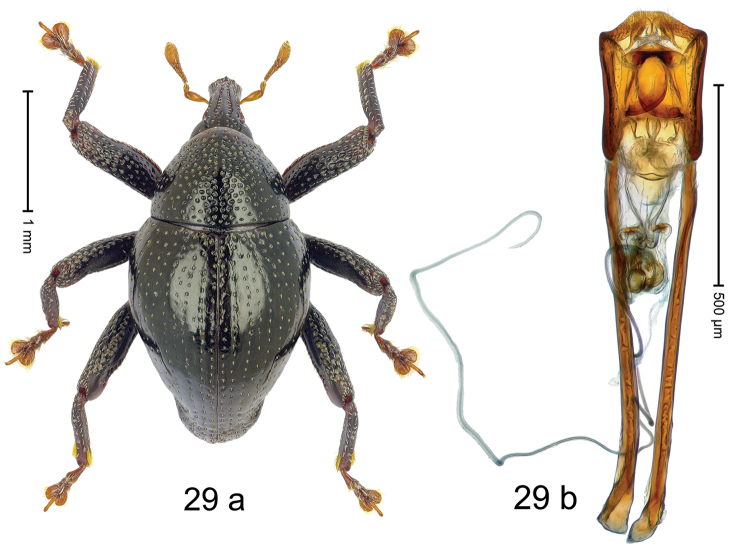
*Trigonopterushypocrita* Riedel, sp. n., holotype; **a** habitus **b** penis.

***Holotype***, male (Fig. 29a). Length 2.63 mm. Color of antennae and tarsi ferruginous; remainder black. Body subovate; in dorsal aspect and in profile with distinct constriction between pronotum and elytron. Rostrum dorsally with median and pair of submedian costae widening towards forehead; intervening furrows with sparse rows of thin setae; epistome posteriorly with transverse, angulate ridge. Pronotum with disk densely punctate; interspaces between punctures subglabrous, laterally subequal to punctures´ diameter; dorsally punctures denser, interspaces reticulate; median line impunctate. Elytra with striae marked by dense rows of minute punctures; along humerus with confused, coarse punctures; intervals subglabrous, with interspersed rows of punctures. Femora edentate; anterior surface coarsely punctate, each puncture with recumbent seta; anteroventral ridge crenate. Metafemur with dorsoposterior edge denticulate; subapically with stridulatory patch. Mesotibia with enlarged, curved uncus. Abdominal ventrite 1 concave; ventrite 2 flat, subglabrous; ventrite 5 with large, deep concavity, punctate, microreticulate, anteriorly with dense erect scales. Penis (Fig. 29b) with sides weakly diverging to apical 1/3, markedly converging to subtruncate apex; body with pair of overlapping crescent-shaped sclerites; apodemes 2.7 × as long as body of penis; transfer apparatus small, directed ventrad; ductus ejaculatorius with indistinct bulbus. ***Intraspecific variation***. Length 2.58–2.63 mm.

#### Material examined.

***Holotype*** (MZB): ARC3123 (GenBank # MK260312), C-Sulawesi Prov., Pendolo, Gn. Sampuraga, 02°15.407'S 120°47.051'E, 1406 m, beaten, 16-V-2013. ***Paratypes*** (SMNK): C-Sulawesi Prov: 1 ex, ARC2866 (GenBank # MK260311), Pendolo, Gn. Sampuraga, 02°15.407'S 120°47.051'E, 1406 m, beaten, 31-V-2012.

#### Distribution.

C-Sulawesi Prov. (Pendolo). Elevation ca. 1400 m.

#### Biology.

On foliage in montane forests.

#### Etymology.

This epithet is the Latin noun *hypocrita* (mime) and refers to its superficial resemblance to other species.

#### Notes.

*Trigonopterushypocrita* Riedel, sp. n. was coded as “*Trigonopterus* sp. 402”.

### 30. Trigonopterus idefix

Taxon classificationAnimaliaColeopteraCurculionidae

Riedel sp. n.

http://zoobank.org/961F1622-CC34-442C-8890-B4C9E66E3CF2

#### Diagnostic description.

**Figure 30.**
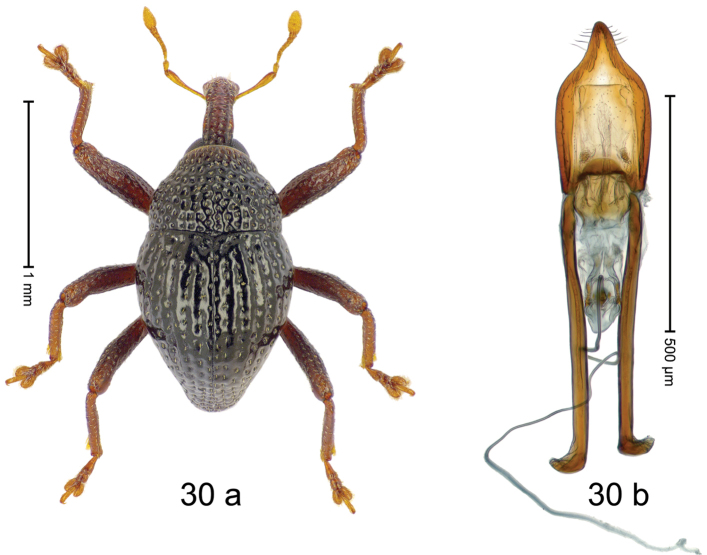
*Trigonopterusidefix* Riedel, sp. n., holotype; **a** habitus **b** penis.

***Holotype***, male (Fig. 30a). Length 1.60 mm. Color of antennae yellowish; legs and rostrum dark ferruginous; remainder black. Body subovate; in dorsal aspect with weak constriction, in profile with distinct constriction between pronotum and elytron. Rostrum long; subapically slightly widened; dorsally with median costa and pair of sublateral ridges; intervening furrows with rows of punctures containing each one indistinct seta; epistome simple, subglabrous. Pronotum with disk densely, coarsely punctate; interspaces between punctures reticulate, subglabrous. Elytra with striae deeply impressed, intervals subglabrous, costate; punctures each with short, suberect seta; sutural interval basally curving laterad. Legs long. Profemur with anteroventral ridge simple; meso- and metafemur with indistinct denticle at middle; anterior surface of femora coriaceous, punctate. Metafemur subapically with stridulatory patch. Abdominal ventrite 1 dull, microreticulate, weakly concave, with coarse punctures; ventrite 5 subglabrous, weakly microreticulate, with shallow concavity. Penis (Fig. 30b) with sides subparallel in basal 1/2, towards apex converging in sinuate line; apex with long, pointed, downwardly curved extension, laterally with sparse setae; basal orifice with well-sclerotized ventral rim; apodemes 1.6 × as long as body of penis; transfer apparatus dentiform, directed basad in repose; ductus ejaculatorius with indistinct bulbus. ***Intraspecific variation***. Length 1.60–1.88 mm. Female rostrum in apical 1/2 subglabrous, with lateral and sublateral rows of punctures. Female abdominal ventrite 5 flat, sparsely punctate.

#### Material examined.

***Holotype*** (MZB): ARC2842 (GenBank # MK260327), S-Sulawesi Prov., Pc. Palopo, Gn. Sampuna, 02°56.539'S 120°05.320'E, 1038 m, sifted, 29-V-2012. ***Paratypes*** (MZB, SMNK): S-Sulawesi Prov.: 5 exx, ARC3087 (GenBank # MK260329), ARC3088 (GenBank # MK260330), ARC3089 (GenBank # MK260331), Pc. Palopo, Gn. Sampuna, 02°56.545'S 120°05.595'E, 1101 m, sifted, 02-V-2013; 28 exx, ARC3016 (GenBank # MK260326), ARC6022 (GenBank # MK260332), ARC6023 (GenBank # MK260328),Tanah Toraja, Rantepao, Gn. Karre (= Gn. Wokim), 02°59.021'S 120°02.523'E, 1456 m, sifted, 06-V-2013.

#### Distribution.

S-Sulawesi Prov. (Tanah Toraja, Pc. Palopo). Elevation 1040–1460 m.

#### Biology.

In leaf litter of montane forest.

#### Etymology.

This epithet is based on a character of the French Asterix comics. It is a noun in apposition.

#### Notes.

*Trigonopterusidefix* Riedel, sp. n. was coded as “*Trigonopterus* sp. 406”.

### 31. Trigonopterus impressicollis

Taxon classificationAnimaliaColeopteraCurculionidae

Riedel sp. n.

http://zoobank.org/EBEAF140-BE40-4802-B1A3-8477C58276C4

#### Diagnostic description.

**Figure 31.**
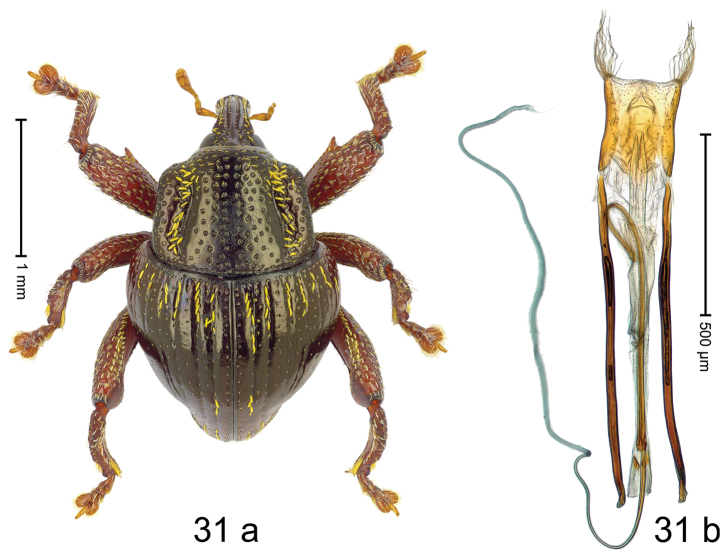
*Trigonopterusimpressicollis* Riedel, sp. n., holotype; **a** habitus **b** penis.

***Holotype***, male (Fig. 31a). Length 2.26 mm. Color of antennae, legs, and elytral humeri ferruginous; remainder black. Body subovate; in dorsal aspect with moderate constriction between pronotum and elytron; in profile dorsally convex. Rostrum dorsally with median carina and pair of submedian ridges; intervening furrows with sparse, erect scales; epistome short, posteriorly with transverse ridge. Pronotum with lateral edges weakly converging, with weak subapical constriction; disk with pair of distinct longitudinal impressions, lined with yellow elongate scales; medially broadly swollen, densely punctate, interspaces subglabrous, with narrow median costa. Elytra sparsely punctate with minute punctures; striae marked by impressed lines; in basal 1/3 and subapically with sparse yellow recumbent scales. Femora dentate, with acute tooth; anteroventral ridge of metafemur crenate. Metafemur with dorsoposterior edge weakly denticulate; subapically with stridulatory patch. Meso- and metatibia subbasally slightly widened, dorsal edge serrate. Metatibia curved. Abdominal ventrites 1–2 concave, subglabrous, sparsely punctate, with few scattered piliform scales; ventrite 5 concave, punctate, with sparse erect setae. Penis (Fig. 31b) with sides of body subparallel; apex subtruncate, laterally with tufts of long curly setae; apodemes 4.0 × as long as body of penis; transfer apparatus flagelliform, 3.5 × longer than body of penis; ductus ejaculatorius with indistinct bulbus. ***Intraspecific variation***. Length 1.90–2.38 mm. Color of elytra ferruginous, partly ferruginous, or black. Female rostrum slender, dorsally in apical 1/2 subglabrous, with submedian row of punctures and with pair of sublateral furrows.

#### Material examined.

***Holotype*** (MZB): ARC3222 (GenBank # MK260468), S-Sulawesi Prov., Mangkutana, 02°20.203'S 120°46.878'E, 903 m, sifted, 14-V-2013. ***Paratypes*** (MZB, SMNK): S-Sulawesi Prov.: 14 exx, ARC3223 (GenBank # MK260472), ARC3224 (GenBank # MK260471), ARC6042 (GenBank # MK260470), ARC6043 (GenBank # MK260469), same data as holotype.

#### Distribution.

S-Sulawesi Prov. (Mangkutana). Elevation ca. 900 m.

#### Biology.

In leaf litter of montane forest.

#### Etymology.

This epithet is an adjectival combination of the Latin participle *impressus*, -*a*, -*um* (imprinted), the noun *collum* (neck), and the second adjectival declension ending -*is*, and refers to the pair of impressions on the pronotum.

#### Notes.

*Trigonopterusimpressicollis* Riedel, sp. n. was coded as “*Trigonopterus* sp. 491”.

### 32. Trigonopterus incendium

Taxon classificationAnimaliaColeopteraCurculionidae

Riedel sp. n.

http://zoobank.org/955B25AD-C35F-4EEC-B13F-095CB8F6C539

#### Diagnostic description.

**Figure 32.**
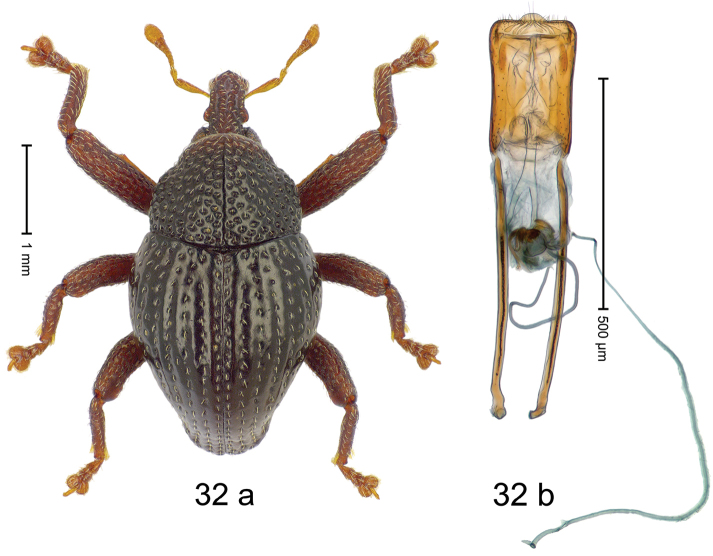
*Trigonopterusincendium* Riedel, sp. n., holotype; **a** habitus **b** penis.

***Holotype***, male (Fig. 32a). Length 1.83 mm. Color of antennae, legs and head ferruginous; remainder black. Body subovate; in dorsal aspect and in profile with weak constriction between pronotum and elytron. Rostrum in front of eye with distinct lateral constriction; dorsally with median and pair of submedian, somewhat irregular ridges; intervening furrows with sparse rows of suberect setae; epistome short, posteriorly with transverse ridge. Pronotum with disk densely punctate with coarse punctures; interspaces between punctures microreticulate; each puncture with fine, recumbent seta; median line costate. Elytra with striae impressed, marked by rows of distinct punctures; punctures each with short, subrecumbent seta; sutural interval punctate, other intervals subglabrous. Femora with small, blunt tooth; anterior surface coarsely rugose-punctate, microgranulate, with sparse recumbent piliform scales. Metafemur subapically with stridulatory patch. Abdominal ventrite 1 medially deeply impressed, behind metacoxa with angular knob; ventrite 5 at middle with distinct depression, coarsely punctate, microgranulate. Penis (Fig. 32b) with sides of body subparallel, slightly diverging; apex subtruncate with short median extension; apodemes 2.0 × as long as body of penis; endophallus containing hyaline Y-shaped structure; transfer apparatus short flagelliform, 0.8 × length of body of penis; ductus ejaculatorius with indistinct bulbus. ***Intraspecific variation***. Length 1.63–1.89 mm. Female rostrum with ridges less distinct, ending in apical 1/2; epistome indistinct. Female abdominal ventrite 5 flat.

#### Material examined.

***Holotype*** (MZB): ARC3247 (EMBL # LN884980), C-Sulawesi Prov., Ampana, Tanjung Api, 00°49.855'S 121°36.493'E, 166 m, sifted, 18-V-2013. ***Paratypes*** (MZB, SMNK, ZSM): C-Sulawesi, Ampana, Tanjung Api: 12 exx, ARC3248 (GenBank # MK260411), ARC3249 (GenBank # MK260412), same data as holotype; 13 exx, ARC3250 (GenBank # MK260410), 00°49.771'S 121°36.527'E, 137 m, sifted, 18-V-2013; 29 exx, 00°49.687'S 121°36.560'E, 130 m, sifted, 18-V-2013.

#### Distribution.

C-Sulawesi Prov. (Ampana). Elevation 130–170 m.

#### Biology.

In leaf litter of lowland forest.

#### Etymology.

This epithet is the Latin noun *incendium* (burning, arson) in apposition and refers to the type locality Tanjung Api (Cape of Fire) with flames of natural gas.

#### Notes.

*Trigonopterusincendium* Riedel, sp. n. was coded as “*Trigonopterus* sp. 471”.

### 33. Trigonopterus incognitus

Taxon classificationAnimaliaColeopteraCurculionidae

Riedel sp. n.

http://zoobank.org/DBE171E0-3687-4352-B952-5E77DF98A28F

#### Diagnostic description.

**Figure 33.**
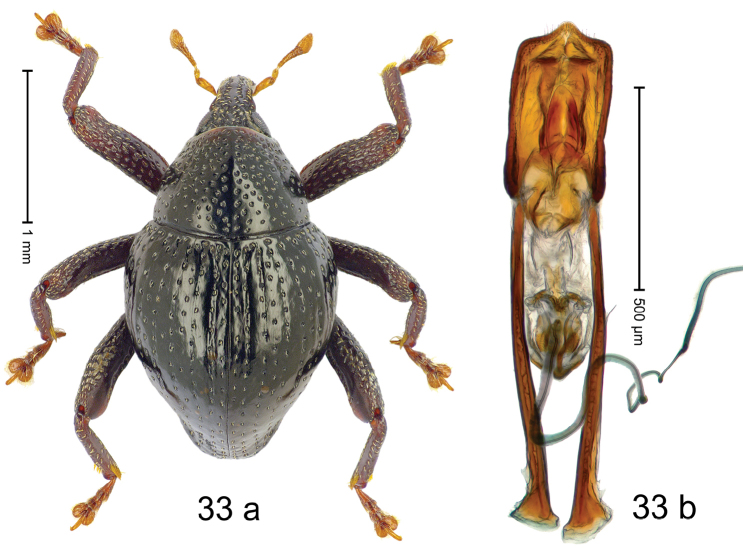
*Trigonopterusincognitus* Riedel, sp. n., holotype; **a** habitus **b** penis.

***Holotype***, male (Fig. 33a). Length 2.24 mm. Color of antennae and tarsi ferruginous; legs dark ferruginous; remainder black. Body subovate; in dorsal aspect and in profile with weak constriction between pronotum and elytron. Rostrum dorsally with rows of coarse punctures each containing one recumbent seta; medially with indistinct subglabrous costa; epistome indistinct, with median denticle. Pronotum with disk densely punctate; each puncture containing fine, recumbent seta; interspaces between punctures subglabrous. Elytra with striae marked by rows of small punctures and fine hairlines, more distinctly impressed near base; intervals subglabrous, with interspersed punctures; stria 8 along humerus with row of coarse punctures. Femora edentate. Meso- and metafemur with anteroventral ridge weakly crenate; anterior surface densely coarsely punctate-rugose, each puncture with recumbent piliform scale. Metafemur with dorsoposterior edge simple, indistinct; subapically with stridulatory patch. Abdominal ventrites 1–2 concave, subglabrous, microreticulate, weakly rugose; ventrite 5 with median depression bordered by sublateral swellings, punctate, microreticulate. Penis (Fig. 33b) with sides of body subparallel; apex subangulate, with sparse setae; apodemes 2.2 × as long as body of penis; endophallus with several sclerites; transfer apparatus anchor-shaped; ductus ejaculatorius with indistinct bulbus. ***Intraspecific variation***. Length 2.24–2.38 mm. Female rostrum slender; dorsally subglabrous, with submedian rows of minute punctures, sublaterally coarsely punctate.

#### Material examined.

***Holotype*** (MZB): ARC3025 (EMBL # LN884950), S-Sulawesi Prov., Tanah Toraja, Bittuang, Gn. Ponding, 02°56.446'S 119°38.075'E, 1625 m, sifted, 09-V-2013. ***Paratypes*** (MZB, SMNK): 2 exx, ARC3026 (GenBank # MK260531), ARC6031 (GenBank # MK260532),same as holotype; 2 exx, ARC3037 (GenBank # MK260533), ARC3038 (GenBank # MK260529), Tanah Toraja, Bittuang, Gn. Ponding, 02°56.446'S 119°38.075'E, 1625 m, beaten; 1 ex, ARC2996 (GenBank # MK260530), Tanah Toraja, Rantepao, Gn. Karre (= Gn. Wokim), 02°59.021'S 120°02.523'E, 1456 m, beaten, 06-V-2013.

#### Distribution.

S-Sulawesi Prov. (Tanah Toraja). Elevation 1460–1625 m.

#### Biology.

Uncertain if more often on foliage or in leaf litter.

#### Etymology.

This epithet is the Latin adjective *incognitus*, -*a*, -*um* (unknown).

#### Notes.

*Trigonopterusincognitus* Riedel, sp. n. was coded as “*Trigonopterus* sp. 510”.

### 34. Trigonopterus indigenus

Taxon classificationAnimaliaColeopteraCurculionidae

Riedel sp. n.

http://zoobank.org/AB740FCF-9088-4F59-A9B4-49C77605CBC0

#### Diagnostic description.

**Figure 34.**
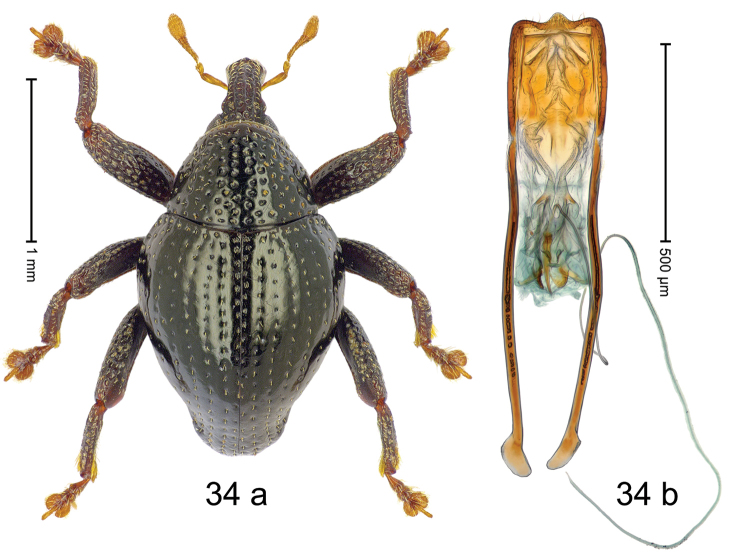
*Trigonopterusindigenus* Riedel, sp. n., holotype; **a** habitus **b** penis.

***Holotype***, male (Fig. 34a). Length 2.12 mm. Color of antennae and tarsi ferruginous; remainder black. Body subovate; in dorsal aspect with weak constriction between pronotum and elytron; in profile dorsally convex. Rostrum dorsally with median and pair of submedian ridges becoming less distinct and replaced by coarse punctures anteriorly; epistome posteriorly with transverse ridge. Pronotum with disk densely punctate with coarse punctures; interspaces between punctures subglabrous, subequal to punctures´ diameter. Elytra with striae marked by small punctures; basal margin bordered by transverse row of deeper punctures joining stria 8; along humerus row of coarse punctures externally bordered by costa; in basal 1/2 suture bordered by row of small punctures; intervals subglabrous. Mesosternal receptacle ventrally with sparse golden scales. Anteroventral ridge of mesofemur and metafemur ending with blunt tooth. Metafemur with dorsoposterior edge denticulate; subapically with stridulatory patch. Abdominal ventrite 1 subglabrous, medially flat behind metacoxa convex; ventrite 2 costate; ventrite 5 flat, subglabrous. Penis (Fig. 34b) with sides of body subparallel; apex subtruncate with short median subacute extension; apodemes 2.8 × as long as body of penis; transfer apparatus complex, partly membranous; ductus ejaculatorius with indistinct bulbus. ***Intraspecific variation***. Length 2.10–2.20 mm. Female rostrum slender, dorsally subglabrous, sublaterally punctate and with pair of indistinct furrows.

#### Material examined.

***Holotype*** (MZB): ARC2767 (GenBank # MK260264), N-Sulawesi Prov., Tomohon, Rurukan, Gn. Mahawu, 01°21.697'N 124°51.885'E, 1176 m, sifted, 14-V-2012. ***Paratypes*** (ARC, MZB, SMNK, ZSM): N-Sulawesi Prov: 13 exx, ARC2768 (GenBank # MK260261), ARC2769 (GenBank # MK260263), same data as holotype; 2 exx, ARC6046 (GenBank # MK260267), ARC6047 (GenBank # MK260265), Tomohon, Rurukan, Gn. Mahawu, 01°21.409'N 124°51.535'E, 1166 m, 14-V-2012, sifted; 16 exx, Tomohon, Rurukan, Gn. Mahawu, 1200 m, 30-XI-1999, sifted; 7 exx, Tomohon, Rurukan, Gn. Mahawu, 01°21.409'N 124°51.535'E, 1126 m, 14-V-2012; 5 exx, Gn. Mahawu, Tomohon, 01°20.951'N 124°52.207'E, 1230 m, 14-V-2012, sifted; 13 exx, Gn. Mahawu, Tomohon, 01°20.791'N 124°52.268'E, 1230 m, sifted, 14-V-2012; 15 exx, Gn. Mahawu, Tomohon, 01°20.844'N 124°52.253'E, 1209 m, sifted, 15-V-2012; 12 exx, Gn. Mahawu, Tomohon, 01°20.819'N 124°52.194'E,1278 m, sifted, 15-V-2012; 4 exx, Gn. Mahawu, Tomohon, 01°20.751'N 124°52.129'E, 1305 m, sifted, 15-V-2012; 2 exx, ARC2919 (GenBank # MK260262), ARC2920 (GenBank # MK260266), Kotamobagu, Modoinding, Kakenturan, 00°47.053'N 124°30.413'E, 1228 m, sifted, 19-V-2012.

#### Distribution.

N-Sulawesi Prov. (Tomohon, Modoinding). Elevation 1170–1305 m.

#### Biology.

In leaf litter of montane forest.

#### Etymology.

This epithet is the Latin adjective *indigenus*, -*a*, -*um* (native).

#### Notes.

*Trigonopterusindigenus* Riedel, sp. n. was coded as “*Trigonopterus* sp. 389”.

### 35. Trigonopterus inhonestus

Taxon classificationAnimaliaColeopteraCurculionidae

Riedel sp. n.

http://zoobank.org/4244BDF3-DB06-451F-AFAD-FB09B8398E70

#### Diagnostic description.

**Figure 35.**
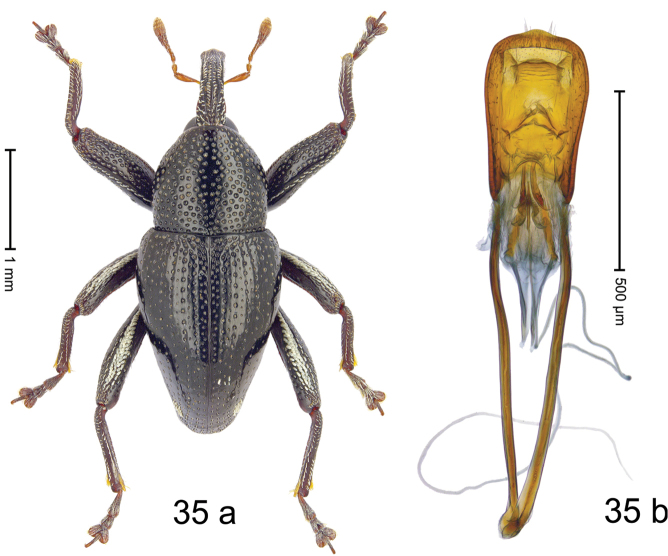
*Trigonopterusinhonestus* Riedel, sp. n., holotype; **a** habitus **b** penis.

***Holotype***, male (Fig. 35a). Length 3.09 mm. Color of antennae ferruginous; tarsi and tibiae dark ferruginous; remainder black. Body elongate; in dorsal aspect and in profile with distinct constriction between pronotum and elytron. Rostrum slender, dorsally with median carina and pair of submedian ridges; intervening furrows with sparse rows cream-colored scales; subapically sparsely punctate-rugose, epistome indistinct. Pronotum with disk densely punctate; punctures becoming larger towards sides; interspaces between punctures subglabrous, reticulate. Elytra with striae marked by fine hairlines and rows of punctures; intervals 1–3 irregularly punctate with smaller punctures; stria 9 along humerus with seven large, coarse punctures, externally bordered by costa; intervals subglabrous; intervals 2–3 subbasally with patch of recumbent cream-colored scales; subapically intervals 3–7 with another patch of recumbent cream-colored scales; intervals 2–3 behind middle with few scattered scales. Femora edentate. Profemur in basal 1/3 posteriorly with callus. Meso- and metafemur on dorsal edge densely squamose with cream-colored scales; anteroventral ridge simple, distinct. Metafemur subapically with indistinct stridulatory patch. Mesocoxa with dense cluster of erect white scales. Abdominal ventrites 1–2 concave, subglabrous microreticulate, sublaterally with coarse punctures; ventrite 5 with shallow impression, at middle, subglabrous, sublaterally coarsely punctate. Penis (Fig. 35b) with sides diverging, anteriorly converging to subangulate apex, with sparse setae; apodemes 2.3 × as long as body of penis; transfer apparatus symmetrical, spiniform, shorter than lateral guiding pieces; ductus ejaculatorius without bulbus. ***Intraspecific variation***. Length 2.51–3.41 mm. Dorsal surface of females rather polished with smaller punctures. Female rostrum more slender, in apical 1/2 subglabrous, dorsally with submedian row of punctures, with sublateral furrow. Elytra with patches of scales slightly smaller or larger, but usually present; rarely abraded. Female mesocoxa with indistinct patch of short scales. Female abdominal ventrites 1–2 flat, subglabrous. Female ventrite 5 flat, punctate.

#### Material examined.

***Holotype*** (MZB): ARC2748 (GenBank # MK260641), N-Sulawesi Prov., Tomohon, Rurukan, Gn. Mahawu, 01°21.409'N 124°51.535'E to 01°21.370'N 124°51.676'E, 1126–1231 m, beaten, 14-V-2012. ***Paratypes*** (ARC, MZB, SMNK, ZSM): S-Sulawesi: 49 exx, ARC2811 (EMBL # LT603143), ARC2812 (GenBank # MK260642), ARC2813 (PCR failed), Pc. Palopo, Gn. Sampuna, 02°56.539'S 120°05.320'E to 02°56.545'S 120°05.595'E, 1038–1101 m, 29-V-2012, beaten; 8 exx, Pc. Palopo, Gn. Sampuna, 800–1050 m, 15–16-IX-1997; 8 exx, 15 km W Palopo, 11–19-VIII-1990; 22 exx, ARC3030 (GenBank # MK260653), ARC3031 (GenBank # MK260650), ARC3032 (GenBank # MK260649), Tanah Toraja, Bittuang, Gn. Ponding, 02°56.446'S 119°38.075'E, 1625 m, beaten, 09-V-2013; 25 exx, ARC3051 (GenBank # MK260651), ARC3052 (GenBank # MK260652), Tanah Toraja, Bittuang, Gn. Karoa, 02°55.270'S 119°40.179'E to 02°55.032'S 119°40.365'E, 1800–2000 m, beaten, 10-V-2013; 1 ex, Tanah Toraja, Bittuang, Gn. Karoa, 02°55.270'S 119°40.179'E, 1836 m, sifted, 10-V-2013; 1 ex, ARC3208 (GenBank # MK260646), Gn. Rantemario, Melaning, 03°19.573'S 119°57.217'E, 1205 m, 07-V-2013; C-Sulawesi: 1 ex, ARC2867 (GenBank # MK260639), Pendolo, Gn. Sampuraga, 02°13.053'S 120°45.653'E, 1178 m, 31-V-2012, beaten; 9 exx, ARC3110 (GenBank # MK260644), ARC3111 (GenBank # MK260643), Pendolo, Boe, 02°05.405'S 120°38.551'E to 02°05.446'S 120°38.519'E, 750–950 m, beaten, 15-V-2013; 2 exx, Pendolo, Boe, 02°05.446'S 120°38.519'E, 915 m, sifted, 15-V-2013; 1 ex, Pendolo, Boe, 21-VIII-1990; 2 exx, Pendolo, ca. 15 km -> Mangkutana, 22-VIII-1990; 2 exx, ARC2879 (GenBank # MK260638), ARC2880 (GenBank # MK260656), Tentena, Taripa, 01°49.858'S 120°47.108'E to 01°50.040'S 120°46.666'E, 896–911 m, 02-VI-2012, beaten; 2 exx, Tentena, Tonusu, 20 km on Bada road, 19–29-IV-1994; N-Sulawesi Prov.: 58 exx, ARC2749 (GenBank # MK260640), same data as holotype; 45 exx, Tomohon, Rurukan, Gn. Mahawu, 01°20.951'N 124°52.207'E, 1230 m, beaten, 14-V-2012; 1 ex, Gn. Mahawu, Tomohon, 01°20.951'N 124°52.207'E, 1230 m, 14-V-2012, sifted; 1 ex, Gn. Mahawu, Tomohon, 01°20.791'N 124°52.268'E,1230 m, sifted, 14-V-2012; 1 exx, Gn. Mahawu, Tomohon, 01°20.751'N 124°52.129'E, 1305 m, sifted, 15-V-2012; 1 ex, Tomohon, Rurukan, Gn. Mahawu, 01°21.409'N 124°51.535'E to 01°21.370'N 124°51.676'E, 1126–1231 m, beaten, 14-V-2012; 143 exx, Tomohon, Rurukan, Gn. Mahawu, 01°20.844'N 124°52.253'E to 01°20.751'N 124°52.129'E, 1209–1305 m, beaten, 15-V-2012; 110 exx, Tomohon, Rurukan, Gn. Mahawu, 1150–1200 m, 30-XI-1999; 2 exx, ARC2799 (GenBank # MK260645), Airmadidi, Gn. Klabat, 01°26.368'N 125°00.609'E to 01°26.505'N 125°00.636'E, 872–923 m, 16-V-2012, beaten; 26 exx, Gn. Soputan, Kawangkoan, Tombasi, 1200–1400 m, 02-XII-1999, beaten; 1 ex, ARC2912 (GenBank # MK260647), Kotamobagu, Modoinding, Kakenturan, 00°46.951'N 124°30.427'E to 00°47.053'N 124°30.413'E, 1210–1228 m, 19-V-2012, beaten; 13 exx, Gn. Ambang, Modoinding, 1200–1450 m, 12-XII-1999; 11 exx, ARC2882 (GenBank # MK260655), ARC2883 (GenBank # MK260654), ARC2884 (GenBank # MK260648), Kotamobagu, Matalibaru, 00°32.445'N 124°14.185'E, 956 m, 20-V-2012, beaten; 8 exx, Kotamobagu, Matalibaru, 700–950 m, 10-XII-1999; 4 exx, Matalibaru, road to Torosik, Gn. Tongara, 900–950 m, 08-XII-1999.

#### Distribution.

S-Sulawesi (Gn. Rantemario, Tanah Toraja, Pc. Palopo), C-Sulawesi (Pendolo, Tentena), N-Sulawesi Prov. (Tomohon, Gn. Klabat, Gn. Soputan, Modoinding, Kotamobagu). Elevation 910–1840 m.

#### Biology.

Beaten from lower vegetation of montane forests that often includes tall grasses (e.g., *Saccharum* sp.). This species may develop in grasses, like some other members of the *T.honestus*-group.

#### Etymology.

This epithet is the Latin adjective *inhonestus*, -*a*, -*um* (disgraceful) and refers to the name of *T.honestus* (Pascoe), a sibling species.

#### Notes.

*Trigonopterusinhonestus* Riedel, sp. n. was coded as “*Trigonopterus* sp. 946”. This species is closely related to *T.honestus* (Pascoe) which occurs in North Maluku and can be distinguished by a longer flagelliform transfer apparatus of the male genitalia, by a slightly larger body size, and by the absence of scale patches near the elytral base. It is the only member of the *T.honestus* group currently known from Sulawesi. It occupies a relatively wide range from Mt Rantemario in South Sulawesi to Tomohon in the north. Different populations are genetically structured but appear conspecific.

### 36. Trigonopterus invalidus

Taxon classificationAnimaliaColeopteraCurculionidae

Riedel sp. n.

http://zoobank.org/990C1308-78A9-43FF-B08F-1F8F8220AF7C

#### Diagnostic description.

**Figure 36.**
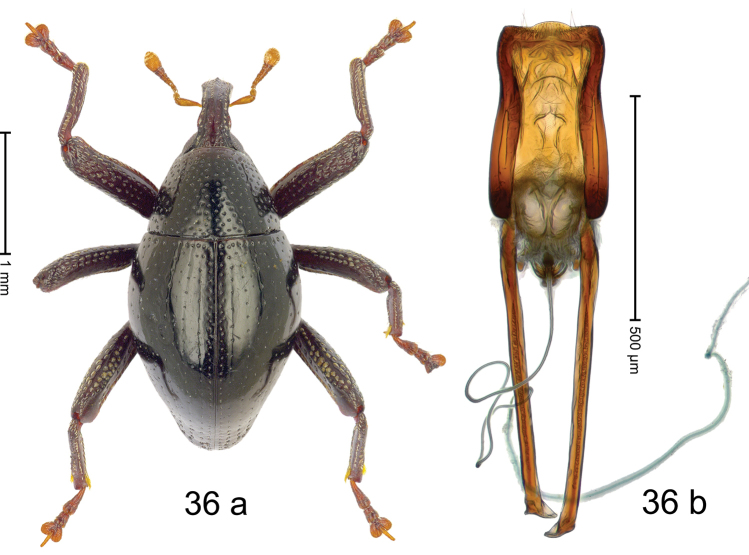
*Trigonopterusinvalidus* Riedel, sp. n., holotype; **a** habitus **b** penis.

***Holotype***, male (Fig. 36a). Length 2.43 mm. Color of antennae and tarsi ferruginous; remainder black. Body subovate; in profile dorsally convex. Rostrum dorsally with median carina; in basal 1/2 with submedian ridges; intervening furrows with sparse rows of piliform scales; epistome indistinct. Pronotum with disk densely punctate with small punctures; interspaces between punctures subglabrous. Elytra with striae marked by minute punctures and indistinct hairlines; basal margin bordered by transverse row of deeper punctures, curving along humeri; intervals flat, subglabrous. Femora edentate. Meso- and metafemur with anteroventral ridge distinct, simple. Metafemur with anterior surface coarsely punctate, each puncture with elongate recumbent silvery scale; with dorsoposterior edge weakly denticulate; subapically with stridulatory patch. Abdominal ventrites 1–2 concave, subglabrous; ventrite 5 flat, coarsely punctate. Penis (Fig. 36b) with sides converging in basal 1/2, anteriorly subparallel; apex subangulate, with few setae; basal orifice of body ventrally with angulate extensions; apodemes 2.2 × as long as body of penis; transfer apparatus complex, small, elongate; ductus ejaculatorius with indistinct bulbus. ***Intraspecific variation***. Length 2.43–2.55 mm. Female rostrum slender, dorsally subglabrous, with two submedian rows of punctures, with pair of sublateral furrows. Female abdominal ventrite 5 flat, punctate, with sparse scales.

#### Material examined.

***Holotype*** (MZB): ARC3189 (EMBL # LN884972), S-Sulawesi Prov., Selayar Is, Pagarangan, 06°18.334'S 120°30.794'E, 545 m, beaten, 24-IV-2013. ***Paratypes*** (MZB, SMNK): 3 exx, ARC3190 (GenBank # MK260517), ARC3191 (GenBank # MK260516), same data as holotype.

#### Distribution.

S-Sulawesi Prov. (Selayar Is). Elevation ca. 545 m.

#### Biology.

On foliage in lowland forests.

#### Etymology.

This epithet is the Latin adjective *invalidus*, -*a*, -*um* (invalid, weak) and refers to the state of the holotype.

#### Notes.

*Trigonopterusinvalidus* Riedel, sp. n. was coded as “*Trigonopterus* sp. 502”.

### 37. Trigonopterus jasminae

Taxon classificationAnimaliaColeopteraCurculionidae

Riedel sp. n.

http://zoobank.org/6F156BB2-B021-4B6E-942F-B3B4A2D8B89E

#### Diagnostic description.

**Figure 37.**
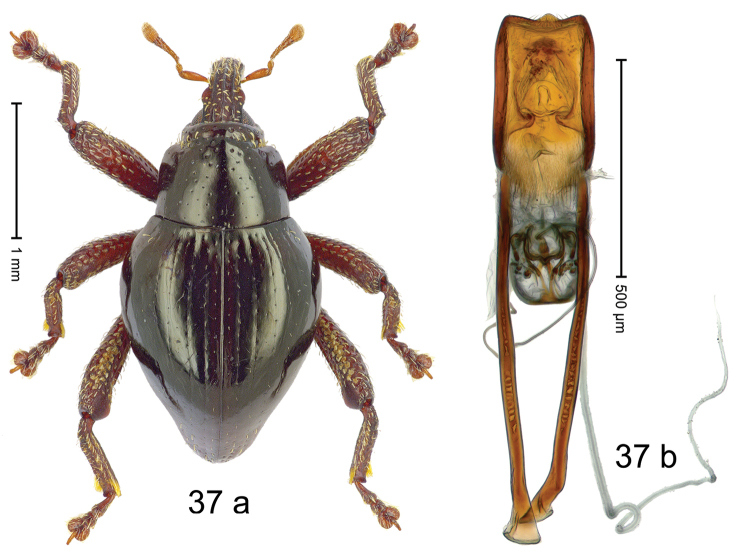
*Trigonopterusjasminae* Riedel, sp. n., holotype; **a** habitus **b** penis.

***Holotype***, male (Fig. 37a). Length 2.53 mm. Color of antennae and legs ferruginous; remainder black. Body subovate; in dorsal aspect with marked constriction between pronotum and elytron; in profile dorsally weakly convex. Rostrum dorsally with median carina and pair of submedian carinae; intervening furrows each with sparse row of elongate, suberect, yellowish scales; epistome indistinct, subglabrous, sparsely punctate. Pronotum with marked subapical constriction, with sparse suberect yellowish scales; disk subglabrous, with few minute punctures; subapically with sparse course punctures. Elytra with striae 1–3 marked by fine hairlines and minute punctures, striae 4–6 marked by minute punctures only; subbasally with few yellowish scales; laterally subglabrous. Femora edentate; anteroventral ridges simple; anterior surface coarsely punctate, especially towards apex, with subrecumbent narrow yellowish scales. Metafemur with dorsoposterior edge denticulate; subapically with stridulatory patch. Metatibia with dorsal edge weakly denticulate; uncus small, weakly curved inwards. Abdominal ventrites 1–2 deeply concave, cavernous, subglabrous; ventrite 5 flat, with sparse suberect yellowish scales. Penis (Fig. 37b) with sides of body subparallel; apex with sparse setae, medially with small angulate extension; apodemes 2.4 × as long as body of penis; transfer apparatus with narrow lyriform frame; ductus ejaculatorius with indistinct bulbus. ***Intraspecific variation***. Length 2.55–3.00 mm. Female rostrum in apical 1/2 with two submedian rows of punctures, with pair of sublateral furrows; basally with median and pair of submedian carinae, intervening furrows with suberect, yellowish scales directed posteriad.

#### Material examined.

***Holotype*** (MZB): ARC2806 (EMBL # LN884930), SE-Sulawesi Prov., Wakatobi, Wangi Wangi Is, Matahora, 05°20.302'S 123°36.680'E, 32 m, sifted, 26-V-2012. ***Paratypes*** (MZB, SMNK): N-Sulawesi Prov.: 19 exx, ARC2804 (GenBank # MK260363), ARC2805 (GenBank # MK260364), ARC2807 (GenBank # MK260365), same data as holotype.

#### Distribution.

SE-Sulawesi Prov. (Wangi Wangi Is). Elevation ca. 30 m.

#### Biology.

In thin leaf litter of lowland forest.

#### Etymology.

This species is named for my daughter Jasmin, who found the first specimen of this species. The epithet is a noun in the genitive case. Invariable.

#### Notes.

*Trigonopterusjasminae* Riedel, sp. n. was coded as “*Trigonopterus* sp. 413”.

### 38. Trigonopterus klabatensis

Taxon classificationAnimaliaColeopteraCurculionidae

Riedel sp. n.

http://zoobank.org/E999C99F-76FB-4521-BD89-D9259768DD30

#### Diagnostic description.

**Figure 38.**
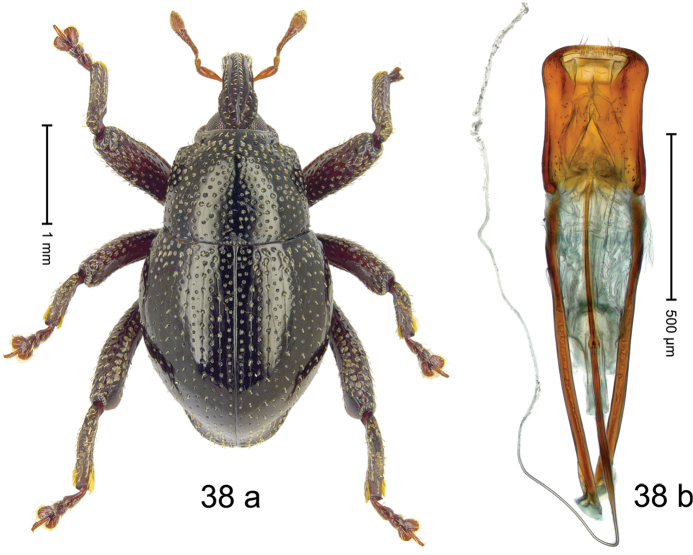
*Trigonopterusklabatensis* Riedel, sp. n., holotype; **a** habitus **b** penis.

***Holotype***, male (Fig. 38a). Length 3.30 mm. Color of antennae ferruginous; legs dark ferruginous; remainder black. Body subovate; in dorsal aspect with weak constriction between pronotum and elytron; in profile dorsally weakly convex. Rostrum dorsally with median carina and pair of submedian carinae, intervening furrows each with sparse row of suberect piliform scales; epistome indistinct. Pronotum with indistinct lateral edges weakly converging to subapical constriction; disk densely punctate, punctures anteriorly and laterally larger, bearing each one suberect transparent scale; median line impunctate. Elytra irregularly punctate; striae marked by hairlines; subapically punctures bearing short suberect transparent scale. Femora with anteroventral ridge ending with denticle, in meso- and metafemur crenate; anterior surface densely coarsely punctate, each puncture with suberect scale. Metafemur with dorsoposterior edge denticulate; subapically with stridulatory patch. Abdominal ventrites 1–2 concave, subglabrous, laterally with flange; ventrite 5 flat, punctate, with sparse suberect scales. Penis (Fig. 38b) with sides of body subparallel, in apical 1/2 weakly widening to rounded apex; apodemes 2.6 × as long as body; transfer apparatus flagelliform, slightly longer than body of penis; ductus ejaculatorius basally swollen, sclerotized, without bulbus.

#### Material examined.

***Holotype*** (MZB): ARC2787 (GenBank # MK260241), N-Sulawesi Prov., Airmadidi, Gn. Klabat, 01°26.635'N 125°00.823'E, 1031 m, sifted, 16-V-2012.

#### Distribution.

N-Sulawesi Prov. (Mt Klabat). Elevation ca. 1030 m.

#### Biology.

In leaf litter of montane forest.

#### Etymology.

This epithet is a Latinized adjective based on the type locality, Mt Klabat.

#### Notes.

*Trigonopterusklabatensis* Riedel, sp. n. was coded as “*Trigonopterus* sp. 383”.

### 39. Trigonopterus kolakensis

Taxon classificationAnimaliaColeopteraCurculionidae

Riedel sp. n.

http://zoobank.org/9353BAB1-AC67-489C-B832-CFD6C6227D1C

#### Diagnostic description.

**Figure 39.**
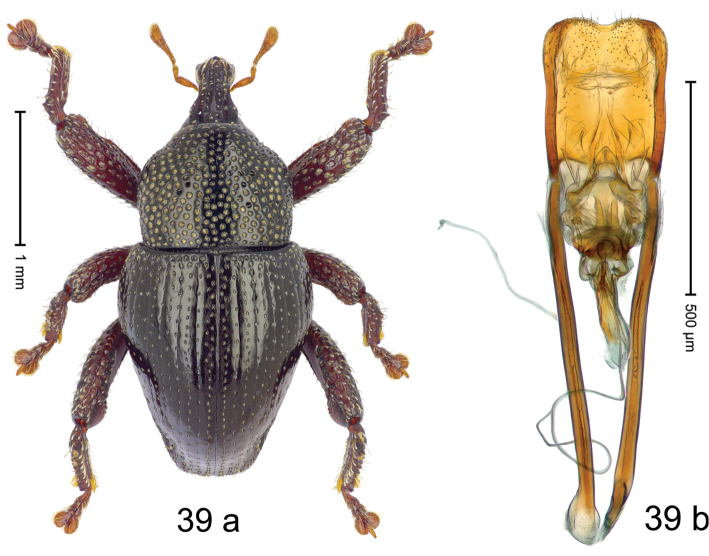
*Trigonopteruskolakensis* Riedel, sp. n., holotype; **a** habitus **b** penis.

***Holotype***, male (Fig. 39a). Length 2.65 mm. Color of antennae and legs ferruginous; remainder black. Body elongate; in dorsal aspect with distinct constriction between pronotum and elytron; in profile dorsally convex. Rostrum dorsally with median carina and pair of submedian ridges; intervening furrows with rows of erect elongate scales; epistome short, posteriorly with transverse, subangulate ridge, medially with denticle. Pronotum with disk densely punctate; interspaces between punctures subglabrous, subequal to or smaller than punctures´ diameter. Elytra densely irregularly punctate with small punctures; along basal margin and humeri with punctation more densely; striae marked by fine hairlines; apex subtruncate, with sparse scales. Femora of meso- and metafemur with anteroventral ridges crenate, ending with small tooth; anterior surface coarsely punctate, reticulate, each puncture with long subclavate scale. Metafemur with dorsoposterior edge serrate; subapically with stridulatory patch. Dorsal edge of tibiae denticulate, subbasally with distinct tooth. Abdominal ventrites 1–2 medially flat, subglabrous; ventrite 5 flat, coarsely punctate. Penis (Fig. 39b) with sides of body subparallel, slightly diverging; apex bisinuate, with sparse short setae; apodemes 2.5 × as long as body of penis; transfer apparatus complex; ductus ejaculatorius without bulbus. ***Intraspecific variation***. Length 1.98–2.83 mm. Female rostrum more slender, dorsally punctate-rugose; epistome indistinct.

#### Material examined.

***Holotype*** (MZB): ARC3159 (EMBL # LN884967), SE-Sulawesi Prov., Kolaka, km 45 on Kendari-road, Talodo village, 04°00.686'S 121°50.198'E, 515 m, sifted, 20-IV-2013. ***Paratypes*** (MZB, SMNK, ZSM): SE-Sulawesi Prov., Kolaka, km 45 on Kendari-road, Talodo village: 54 exx, ARC3160 (GenBank # MK260439), ARC3161 (GenBank # MK260438), same data as holotype; 40 exx, ARC3162 (GenBank # MK260437), 04°00.763'S 121°50.175'E, 471 m, sifted, 20-IV-2013; 4 exx, 04°00.673'S 121°49.869'E, 565 m, sifted, 20-IV-2013.

#### Distribution.

SE-Sulawesi Prov. (Kolaka). Elevation 470–565 m.

#### Biology.

In leaf litter of lowland forest.

#### Etymology.

This epithet is a Latinized adjective based on the type locality.

#### Notes.

*Trigonopteruskolakensis* Riedel, sp. n. was coded as “*Trigonopterus* sp. 482”.

### 40. Trigonopterus kotamobagensis

Taxon classificationAnimaliaColeopteraCurculionidae

Riedel sp. n.

http://zoobank.org/0E28EA4A-968D-4F69-A3DF-AB3AE6DE45FF

#### Diagnostic description.

**Figure 40.**
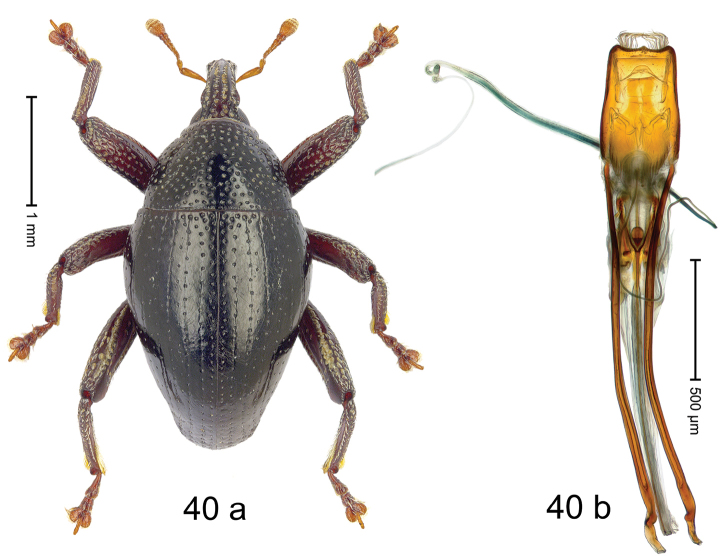
*Trigonopteruskotamobagensis* Riedel, sp. n., holotype; **a** habitus **b** penis.

***Holotype***, male (Fig. 40a). Length 3.00 mm. Color of antennae ferruginous; legs dark ferruginous; remainder black. Body subovate; in profile dorsally convex, elytral base weakly projecting. Rostrum dorsally with median and pair of shorter submedian ridges; intervening furrows each with sparse row of piliform scales; in apical 1/3 with median costa and two indistinct pairs of submedian / sublateral denticles; epistome indistinct. Pronotum with disk densely punctate; median line impunctate; interspaces between punctures subglabrous, subequal to or smaller than punctures´ diameter. Elytra punctate with small punctures; basal margin bordered by transverse row of denser punctures; striae marked by hairlines; interspaces between punctures subglabrous; striae 7–9 with larger punctures, stria 8 along humerus with six large, coarse punctures. Femora edentate. Metafemur with dorsoposterior edge denticulate, with row of suberect transparent scales; subapically with stridulatory patch. Dorsal edge of tibiae with subbasal angulation. Posterior face of metatibia in apical 1/2 with dense yellowish setae. Abdominal ventrites 1–2 concave, subglabrous; ventrite 5 flat, subglabrous, apex sparsely punctate, curved ventrad. Penis (Fig. 40b) with sides of body subparallel; apex subtruncate, with short median extension, with dense fringe of curved setae; apodemes 3.5 × as long as body; transfer apparatus flagelliform, supported by M-shaped sclerite, directed basad in repose, 3.5 × longer than body of penis; ductus ejaculatorius with distinct bulbus.

#### Material examined.

***Holotype*** (MZB): ARC2888 (EMBL # LN884943), N-Sulawesi Prov., Kotamobagu, Matalibaru, 00°32.445'N 124°14.185'E, 956 m, beaten, 20-V-2012.

#### Distribution.

N-Sulawesi Prov. (Kotamobagu). Elevation 960 m.

#### Biology.

On foliage in montane forests.

#### Etymology.

This epithet is a Latinized adjective based on the type locality.

#### Notes.

*Trigonopteruskotamobagensis* Riedel, sp. n. was coded as “*Trigonopterus* sp. 380”.

### 41. Trigonopterus laevigatus

Taxon classificationAnimaliaColeopteraCurculionidae

Riedel sp. n.

http://zoobank.org/E84E9B2A-4A68-4DCF-904D-748385456AFE

#### Diagnostic description.

**Figure 41.**
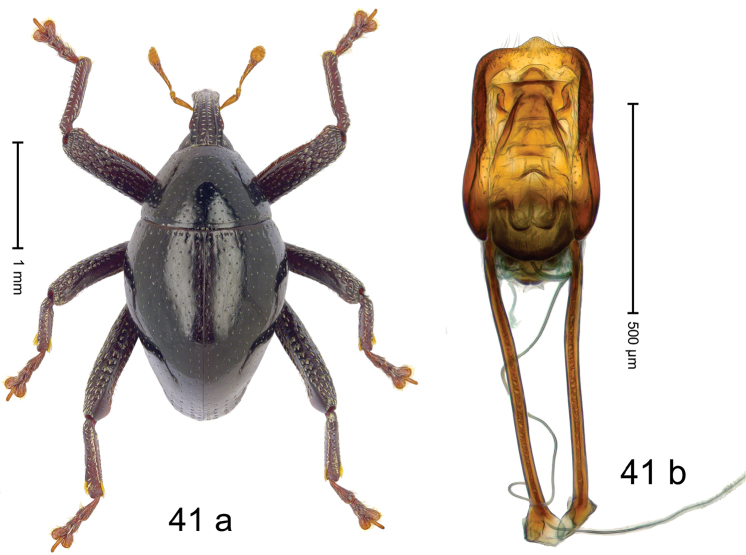
*Trigonopteruslaevigatus* Riedel, sp. n., holotype; **a** habitus **b** penis.

***Holotype***, male (Fig. 41a). Length 2.63 mm. Color of antennae and tarsi ferruginous; remainder black. Body subovate; in profile dorsally convex. Rostrum dorsally with median carina and pair of submedian ridges; intervening furrows with sparse rows of elongate suberect scales. Pronotum with disk punctate with minute punctures; interspaces between punctures subglabrous. Elytra irregularly punctate with minute punctures; basal margin bordered by transverse row of deeper punctures, continuing laterad along humerus; stria 8 near humerus with five large punctures. Femora edentate. Meso- and metafemur with anteroventral ridge crenate. Metafemur with anterior surface coarsely punctate, reticulate, each puncture with elongate silvery scale; with dorsoposterior edge denticulate; subapically with stridulatory patch. Metatibia with dorsal edge in apical 1/3 forming denticle, subapically with dorsal contour concave. Abdominal ventrites 1–2 concave, subglabrous; ventrite 5 with broad transverse ovate depression, punctate. Penis (Fig. 41b) with sides converging in basal 1/2, anteriorly subparallel; apex with subangulate extension, with sparse setae; apodemes 2.1 × as long as body of penis; transfer apparatus spiniform, directed basad in repose, attached to anchor-shaped supporting sclerite; ductus ejaculatorius with indistinct bulbus. ***Intraspecific variation***. Length 2.53–2.70 mm. Female rostrum slender, dorsally subglabrous, with two submedian rows of punctures, with pair of sublateral furrows. Female abdominal ventrite 5 flat, punctate, with sparse erect scales.

#### Material examined.

***Holotype*** (MZB): ARC3147 (GenBank # MK260510), SE-Sulawesi Prov., Kendari, road from Wawotobi to Lasolo, 03°44.142'S 122°13.670'E, 482 m, beaten, 17-IV-2013. ***Paratypes*** (MZB, SMNK): SE-Sulawesi Prov.: 5 exx, ARC3148 (GenBank # MK260511), ARC3149 (GenBank # MK260512), ARC3153 (GenBank # MK260513), ARC3154 (GenBank # MK260508), same data as holotype; 5 exx, Kendari, road from Wawotobi to Lasolo, 03°46.422'S 122°12.576'E, 334 m, beaten, 17-IV-2013; 2 exx, Kendari, road from Wawotobi to Lasolo, 03°45.086'S 122°12.976'E, 339 m, beaten, 17-IV-2013; 21 exx, ARC3156 (GenBank # MK260509), ARC3157 (GenBank # MK260505), ARC3158 (GenBank # MK260515), Kolaka, km 28 on Kendari-road, Mowewe village, beaten, 04°01.063'S 121°44.485'E to 04°01.298'S 121°44.873'E, 312–481 m, 19-IV-2013; 3 exx, ARC3168 (GenBank # MK260507), ARC3169 (GenBank # MK260506), Kolaka, km 45 on Kendari-road, Talodo village, beaten, 04°00.686'S 121°50.198'E to 04°00.673'S 121°49.869'E, 450–565 m, 20-IV-2013; 3 exx, ARC3183 (EMBL # LN884971), ARC3184 (GenBank # MK260514), N Kolaka, Uluwolo village, 03°48.073'S 121°16.856'E, 100–200 m, beaten, 21-IV-2013.

#### Distribution.

SE-Sulawesi Prov. (Kendari, Kolaka). Elevation 200–480 m.

#### Biology.

On foliage in lowland forests.

#### Etymology.

This epithet is the Latin adjective *laevigatus*, -*a*, -*um* (smooth, slippery).

#### Notes.

*Trigonopteruslaevigatus* Riedel, sp. n. was coded as “*Trigonopterus* sp. 501”.

### 42. Trigonopterus lampros

Taxon classificationAnimaliaColeopteraCurculionidae

Riedel sp. n.

http://zoobank.org/908A22DF-77F1-46DB-97AF-EAA3813CFC7C

#### Diagnostic description.

**Figure 42.**
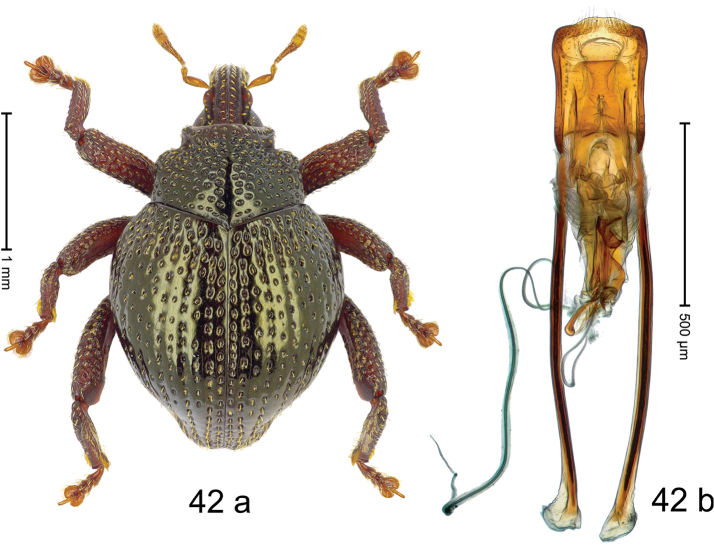
*Trigonopteruslampros* Riedel, sp. n., holotype; **a** habitus **b** penis.

***Holotype***, male (Fig. 42a). Length 2.49 mm. Color of antennae light ferruginous; legs and rostrum dark ferruginous; remainder black with bronze luster. Body subglobose; in dorsal aspect with distinct constriction between pronotum and elytron; in profile dorsally convex. Rostrum dorsally with distinct median carina and pair of submedian ridges; intervening furrows each with row of erect, clavate scales; epistome short, subglabrous, indistinct. Pronotum laterally with marked subapical constriction; disk coarsely punctate; interspaces subglabrous, reticulate; with subglabrous median costa. Elytra with basal margin subangulate; densely punctate with coarse punctures, especially near base where punctures partly confluent; interspaces subglabrous; each puncture with suberect to subrecumbent, weakly clavate, scale. Femora edentate; anteroventral ridge distinct; anterior surface rugose-punctate, microgranulate, dull, with sparse subrecumbent scales. Metafemur dorsally denticulate; subapically with stridulatory patch. Metatibia with dorsal edge denticulate; ventrally with fringe of sparse setae. Abdominal ventrites 1–2 concave, subglabrous, sparsely punctate, with sparse suberect scales; ventrite 5 flat, densely coarsely punctate, with suberect slender scales. Penis (Fig. 42b) with sides of body subparallel, slightly widening before weakly extended apex; apodemes 2.9 × as long as body of penis; transfer apparatus complex, asymmetrical; ductus ejaculatorius with distinct bulbus. ***Intraspecific variation***. Length 2.18–2.49 mm. Female rostrum dorsally flattened, with ridges less distinct; with median costa and pair of submedian costae less distinct, anteriorly separated by row of punctures.

#### Material examined.

***Holotype*** (MZB): ARC3196 (EMBL # LN884973), S-Sulawesi Prov., Selayar Is, Bahorea, 06°20.484'S 120°30.127'E, 368 m, sifted, 25-IV-2013. ***Paratype*** (SMNK): 1 ex, ARC3188 (GenBank # MK260426), S-Sulawesi Prov., Selayar Is, Pagarangan, 06°18.334'S 120°30.794'E, 545 m, 24-IV-2013.

#### Distribution.

S-Sulawesi Prov. (Selayar Is). Elevation 370–545 m.

#### Biology.

In leaf litter of lowland forest.

#### Etymology.

This epithet is based on the Greek adjective *lampros* (shining, bright) and refers to the species´ metallic luster. To be treated as a noun in apposition.

#### Notes.

*Trigonopteruslampros* Riedel, sp. n. was coded as “*Trigonopterus* sp. 478”.

### 43. Trigonopterus latipennis

Taxon classificationAnimaliaColeopteraCurculionidae

Riedel sp. n.

http://zoobank.org/6F51741C-6E73-435D-B7B3-55B0D2A91CF0

#### Diagnostic description.

**Figure 43.**
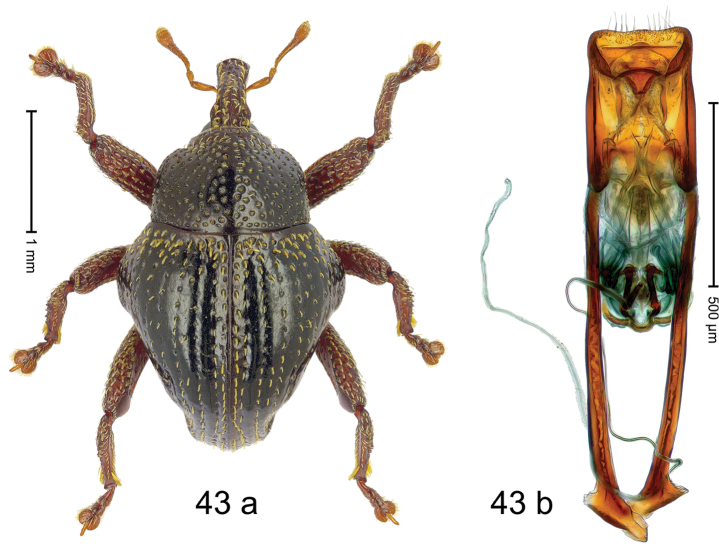
*Trigonopteruslatipennis* Riedel, sp. n., holotype; **a** habitus **b** penis.

***Holotype***, male (Fig. 43a). Length 2.68 mm. Color of antennae and legs ferruginous; remainder black. Body subrhomboid; in dorsal aspect with constriction between pronotum and elytron; in profile dorsally convex. Rostrum dorsally with median and pair of submedian ridges; intervening furrows with rows of suberect yellowish scales; epistome short, subglabrous, posteriorly with transverse ridge. Pronotum with lateral edges subparallel, weakly converging, rounded to subapical constriction; disk densely punctate, forming broad ridge with impunctate median line; anteriorly some punctures bearing long, slender scales. Elytra with humeri laterally markedly protruding; striae marked by small punctures; stria 1 more distinctly impressed; intervals subglabrous, with sparse recumbent yellowish scales; humeri densely irregularly punctate; along base transversely impressed, with punctures larger, scales denser and wider. Femora edentate; anteroventral ridge distinct, simple. Metafemur with dorsoposterior edge denticulate; subapically with stridulatory patch. Metatibia in basal 1/2 with dorsal edge denticulate; in apical 1/2 slender, posteroventrally densely clothed with short yellowish setae. Abdominal ventrites 1–2 concave, subglabrous; ventrite 5 flat, sparsely punctate. Penis (Fig. 43b) with sides of body subparallel; apex weakly rounded, with setae; apodemes 2.5 × as long as body of penis; transfer apparatus complex; ductus ejaculatorius with indistinct bulbus. ***Intraspecific variation***. Length 2.14–2.68 mm. Female body more slender, subovate, humeri not protruding; female rostrum subglabrous with submedian row of punctures and sublateral pair of furrows; epistome simple.

#### Material examined.

***Holotype*** (MZB): ARC2773 (GenBank # MK260383), N-Sulawesi Prov., Airmadidi, Gn. Klabat, 01°26.505'N 125°00.636'E, 923 m, sifted, 16-V-2012. ***Paratypes*** (MZB, SMNK): N-Sulawesi Prov.: 5 exx, ARC2774 (GenBank # MK260382), ARC2775 (GenBank # MK260378), ARC2776 (GenBank # MK260381), same data as holotype; 3 exx, ARC2794 (GenBank # MK260380), ARC2796 (GenBank # MK260379), Airmadidi, Gn. Klabat, 01°26.430'N 125°00.622'E, 889 m, sifted, 17-V-2012; 4 exx, ARC0329 (GenBank # MK260386), ARC0330 (EMBL # HG939576), ARC0331 (GenBank # MK260385), ARC0332 (GenBank # MK260384), Bitung, Gn. Dua Saudara, 01°29.417'N 125°09.150'E, sifted, 25-VI-2006.

#### Distribution.

N-Sulawesi Prov. (Mt Klabat, Bitung). Elevation 890–920 m.

#### Biology.

In leaf litter of montane forest.

#### Etymology.

This epithet is an adjectival combination of the Latin adjective *latus*, -*a*, -*um* (wide), the noun *penna* (wing, elytron) and the second adjectival declension ending -*is*, and refers to the body shape of this species.

#### Notes.

*Trigonopteruslatipennis* Riedel, sp. n. was coded as “*Trigonopterus* sp. 418”.

### 44. Trigonopterus lompobattangensis

Taxon classificationAnimaliaColeopteraCurculionidae

Riedel sp. n.

http://zoobank.org/0C7F2E43-CB50-4D82-922A-9A2B81B91A75

#### Diagnostic description.

**Figure 44.**
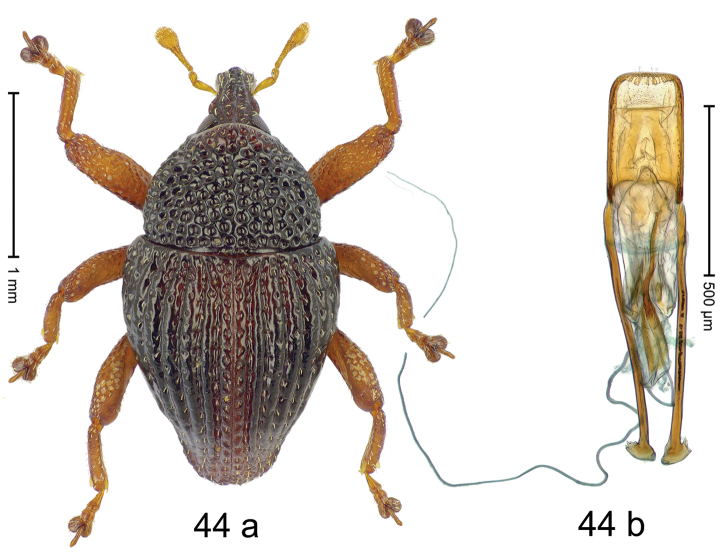
*Trigonopteruslompobattangensis* Riedel, sp. n., holotype; **a** habitus **b** penis.

***Holotype***, male (Fig. 44a). Length 2.24 mm. Color of antennae and legs ferruginous; remainder dark ferruginous to black. Body subovate; in dorsal aspect with weak constriction between pronotum and elytron; in profile dorsally convex, with very weak constriction between pronotum and elytron. Rostrum dorsally coarsely rugose punctate; with sparse suberect setae; epistome posteriorly with transverse ridge, medially and laterally with small denticle. Pronotum with weak subapical constriction; disk coarsely punctate-reticulate; with irregular median ridge. Elytra with striae deeply impressed, containing rows of punctures each with short, suberect seta; humerus irregularly scabrous; sutural interval punctate, other intervals costate, subglabrous, with sparse punctures. Femora with small, blunt denticle; anterior surface rugose-punctate, dull, with sparse recumbent piliform scales. Metafemur subapically with stridulatory patch. Abdominal ventrites 1–2 concave, microreticulate, with coarse punctures; ventrite 5 flat, at middle with shallow impression, microreticulate, coarsely punctate. Penis (Fig. 44b) with sides of body subparallel; apex rounded; apodemes 2.8 × as long as body of penis; transfer apparatus spiniform to short flagelliform, 1.7 × length of body of penis; ductus ejaculatorius without bulbus. ***Intraspecific variation***. Length 2.12–2.24 mm. Female rostrum slender, dorsally with ridges less distinct, with median costa and submedian row of coarse punctures; epistome indistinct. Female abdominal ventrite 5 flat.

#### Material examined.

***Holotype*** (MZB): ARC3198 (GenBank # MK260424), S-Sulawesi Prov., Gn. Lompobattang, Malakaji, Parambintolo, 05°23.545'S 119°55.517'E, 1803 m, sifted, 27-IV-2013. ***Paratype*** (SMNK): 1 ex, ARC3199 (GenBank # MK260425), same data as holotype.

#### Distribution.

S-Sulawesi Prov. (Gn. Lompobattang). Elevation ca. 1800 m.

#### Biology.

In leaf litter of montane forest.

#### Etymology.

This epithet is a Latinized adjective based on Mt Lompobattang.

#### Notes.

*Trigonopteruslompobattangensis* Riedel, sp. n. was coded as “*Trigonopterus* sp. 477”.

### 45. Trigonopterus luwukensis

Taxon classificationAnimaliaColeopteraCurculionidae

Riedel sp. n.

http://zoobank.org/ACABCF52-81B7-4D80-9638-E59671BEC74C

#### Diagnostic description.

**Figure 45.**
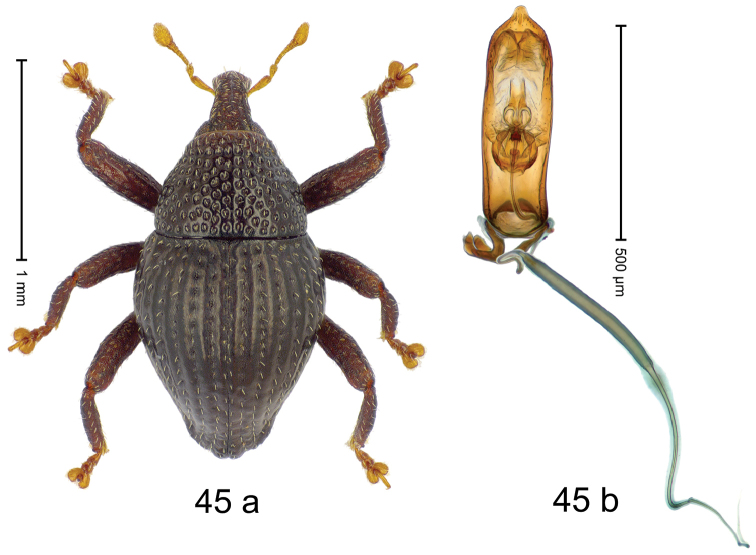
*Trigonopterusluwukensis* Riedel, sp. n., holotype; **a** habitus **b** penis.

***Holotype***, male (Fig. 45a). Length 1.61 mm. Color of antennae light ferruginous; legs and head dark ferruginous; remainder black. Body subovate; in dorsal aspect and in profile with constriction between pronotum and elytron. Rostrum dorsally coarsely punctate-rugose, with median ridge; with sparse rows of suberect setae; epistome indistinct, sparsely punctate. Pronotum with weak subapical constriction; disk coarsely punctate, interspaces reticulate; each puncture with suberect, curved seta. Elytra with striae deeply impressed; each puncture with suberect, curved seta; intervals costate, subglabrous, weakly microreticulate. Meso- and metafemur with small tooth, profemur edentate. Metafemur subapically without stridulatory patch. Abdominal ventrite 5 large, flat, markedly microreticulate. Penis (Fig. 45b) hardly asymmetrical; tip medially weakly extended; basal orifice ventrally simple; apodemes shorter than body of penis (0.6 ×); ductus ejaculatorius with distinct bulbus. ***Intraspecific variation***. Length 1.61–1.82 mm. Female rostrum dorsally subglabrous, densely punctate; epistome indistinct.

#### Material examined.

***Holotype*** (MZB): ARC3274 (EMBL # LN884982), C-Sulawesi Prov., Luwuk, Salodi, Gn. Taluanjang, 00°49.202'S 122°52.395'E, 702 m, sifted, 21-V-2013. ***Paratypes*** (MZB, SMNK): 5 exx, ARC3275 (GenBank # MK260406), ARC3276 (GenBank # MK260405), same data as holotype.

#### Distribution.

C-Sulawesi Prov. (Luwuk). Elevation ca. 700 m.

#### Biology.

In leaf litter of lower montane forest.

#### Etymology.

This epithet is a Latinized adjective based on the town of Luwuk.

#### Notes.

*Trigonopterusluwukensis* Riedel, sp. n. was coded as “*Trigonopterus* sp. 466”.

### 46. Trigonopterus mahawuensis

Taxon classificationAnimaliaColeopteraCurculionidae

Riedel sp. n.

http://zoobank.org/4CDD6C21-46CA-4DB1-9AF0-3ED04765E96F

#### Diagnostic description.

**Figure 46.**
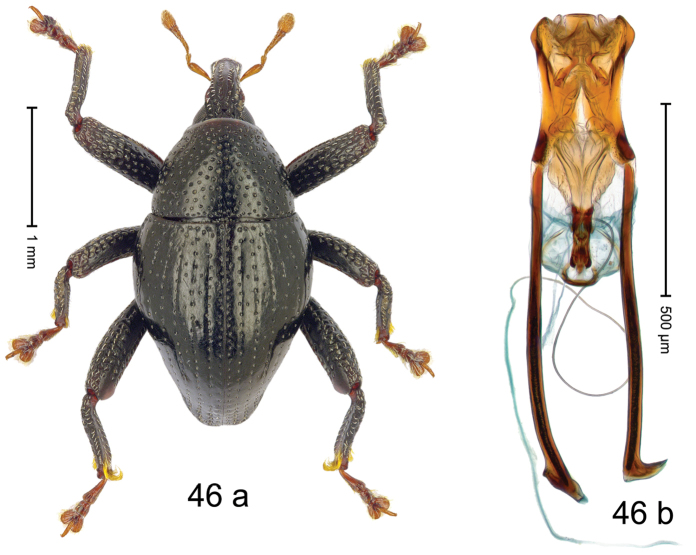
*Trigonopterusmahawuensis* Riedel, sp. n., holotype; **a** habitus **b** penis.

***Holotype***, male (Fig. 46a). Length 2.58 mm. Color of antennae and tarsi ferruginous; remainder black. Body subovate; in dorsal aspect with weak constriction between pronotum and elytron; in profile dorsally convex. Rostrum dorsally with median costa and pair of submedian ridges; intervening furrows each with row of subrecumbent setae. Pronotum with disk densely punctate; interspaces between punctures subglabrous, subequal to or smaller than punctures´ diameter. Elytra with striae marked by fine lines and rows of small punctures; basal margin bordered by transverse row of deeper punctures; stria 8 along humerus with six larger punctures, externally bordered by costa; sutural interval with additional row of small punctures, other intervals subglabrous, with few interspersed punctures. Femora edentate; anteroventral ridge of mesofemur and metafemur crenate; anterior surface coarsely punctate, each puncture with recumbent narrow scale. Metafemur with dorsoposterior edge denticulate; subapically with stridulatory patch. Metatibia subapically near tarsal articulation with dense cluster of strongly incurved setae. Abdominal ventrites 1–2 concave, subglabrous; ventrite 5 with apical 1/2 bent ventrad; sparsely punctate. Penis (Fig. 46b) with sides of body concave, widening to apex; with ostium protruding over ventral rim of penis, without setae; apodemes 2.2 × as long as body of penis; transfer apparatus complex; ductus ejaculatorius without bulbus. ***Intraspecific variation***. Length 2.20–2.63 mm. Female rostrum with low, widened median costa; separated from submedian costae by row of punctures. Female abdominal ventrite 5 flat, subapically with sparse recumbent scales.

#### Material examined.

***Holotype*** (MZB): ARC2763 (GenBank # MK260279), N-Sulawesi Prov., Tomohon, Rurukan, Gn. Mahawu, 01°20.844'N 124°52.253'E to 01°20.751'N 124°52.129'E, 1209–1305 m, beaten, 15-V-2012. ***Paratypes*** (MZB, SMNK): N-Sulawesi Prov.: 81 exx, ARC2764 (GenBank # MK260282), same data as holotype; 35 exx, ARC2750 (GenBank # MK260280), ARC2751 (GenBank # MK260281), Tomohon, Rurukan, Gn. Mahawu, 01°21.409'N 124°51.535'E to 01°21.370'N 124°51.676'E, 1126–1231 m, beaten, 14-V-2012; 27 exx, Tomohon, Rurukan, Gn. Mahawu, 01°20.951'N 124°52.207'E, 1230 m, beaten, 14-V-2012; 5 exx, Gn. Mahawu, Tomohon, 01°20.951'N 124°52.207'E, 1230 m, 14-V-2012, sifted; 1 ex, Gn. Mahawu, Tomohon, 01°20.819'N 124°52.194'E,1278 m, sifted, 15-V-2012; 26 exx, Tomohon, Rurukan, Gn. Mahawu, 1150–1200 m, beaten, 30-XI-1999; 10 exx, Tomohon, Rurukan, Gn. Mahawu, 1150–1200 m, beaten, 30-XI-1999 / 03-XII-1999; 11 exx, Gn. Soputan, Kawangkoan, Tombasi, 1200–1400 m, beaten, 02-XII-1999.

#### Distribution.

N-Sulawesi Prov. (Tomohon, Mt Soputan). Elevation 1200–1280 m.

#### Biology.

On foliage in montane forests.

#### Etymology.

This epithet is a Latinized adjective based on the type locality Mt Mahawu.

#### Notes.

*Trigonopterusmahawuensis* Riedel, sp. n. was coded as “*Trigonopterus* sp. 394”.

### 47. Trigonopterus manadensis

Taxon classificationAnimaliaColeopteraCurculionidae

Riedel sp. n.

http://zoobank.org/88CD1735-590E-4646-9295-30B0DA2E28AF

#### Diagnostic description.

**Figure 47.**
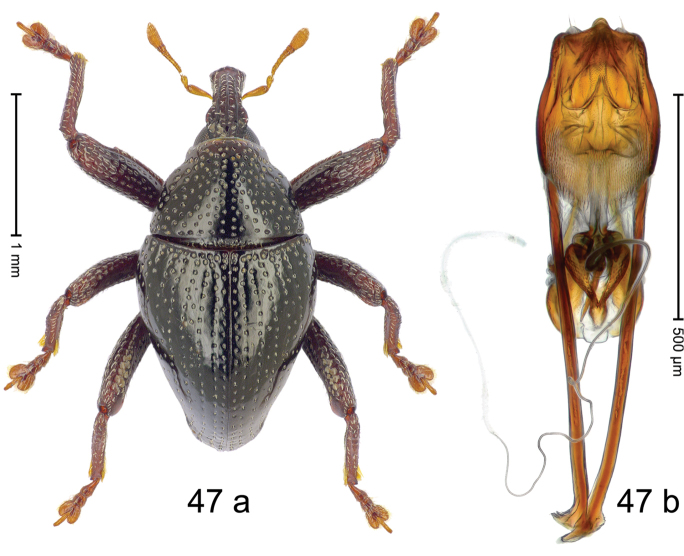
*Trigonopterusmanadensis* Riedel, sp. n., holotype; **a** habitus **b** penis.

***Holotype***, male (Fig. 47a). Length 2.26 mm. Color of antennae and tarsi ferruginous; remainder black. Body subovate; in profile dorsally convex. Rostrum dorsally with median and pair of submedian ridges; intervening furrows with sparse rows of suberect setae; epistome short, posteriorly with transverse, angulate ridge. Pronotum with weak subapical constriction; disk densely punctate; in apical 1/2 forming broad ridge with impunctate median line; interspaces between punctures subglabrous, subequal to or smaller than punctures´ diameter. Elytra with striae marked by rows of small punctures; stria 8 and 9 along humerus with large, coarse punctures; sutural interval with additional row, other intervals subglabrous, with few interspersed punctures; elytral base weakly costate. Femora with anteroventral ridges ending with small tooth; anterior surface coarsely punctate, each puncture with recumbent yellowish seta. Metafemur with dorsoposterior edge denticulate; subapically with stridulatory patch. Abdominal ventrite 1 and anterior portion of ventrite 2 concave, subglabrous; ventrite 2 posteriorly forming rim; ventrite 5 weakly concave, almost flat; sparsely punctate. Penis (Fig. 47b) with sides of body in basal 1/2 rounded, converging to pointed apex; in profile body of penis markedly curved ventrad; apodemes 2.2 × as long as body of penis; transfer apparatus complex; ductus ejaculatorius without bulbus. ***Intraspecific variation***. Length 2.22–2.36 mm. Female rostrum dorsally punctate-rugose, with median costa; epistome indistinct. Female abdominal ventrite 5 flat, subapically with sparse recumbent scales.

#### Material examined.

***Holotype*** (MZB): ARC2753 (GenBank # MK260295), N-Sulawesi Prov., Tomohon, Rurukan, Gn. Mahawu, 01°21.409'N 124°51.535'E to 01°21.370'N 124°51.676'E, 1126–1231 m, beaten, 14-V-2012. ***Paratypes*** (MZB, SMNK): N-Sulawesi Prov.: 2 exx, ARC2752 (GenBank # MK260294), ARC2760 (GenBank # MK260287), same data as holotype; 10 exx, ARC2765 (GenBank # MK260288), ARC2766 (GenBank # MK260289), Tomohon, Rurukan, Gn. Mahawu, 01°21.697'N 124°51.885'E, 1176 m, 14-V-2012; 8 exx, Gn. Mahawu, Tomohon, 01°20.844'N 124°52.253'E, 1209 m, sifted, 15-V-2012; 7 exx, Tomohon, Rurukan, Gn. Mahawu, 1200 m, beaten, 30-XI-1999; 3 exx, Gn. Mahawu, Tomohon, 01°20.951'N 124°52.207'E, 1230 m, 14-V-2012, sifted; 11 exx, ARC6051 (GenBank # MK260293), ARC6052 (GenBank # MK260286), Gn. Mahawu, Tomohon, 01°20.791'N 124°52.268'E, 1230 m, sifted, 14-V-2012; 1 ex, Gn. Mahawu, Tomohon, 01°20.819'N 124°52.194'E, 1278 m, sifted, 15-V-2012; 1 ex, ARC6048 (GenBank # MK260292), Tomohon, Rurukan, Gn. Mahawu, 01°21.409'N 124°51.535'E, 1166 m, 14-V-2012, sifted; 2 exx, Airmadidi, Gn. Klabat, 01°26.430'N 125°00.622'E, 889 m, sifted, 17-V-2012; 3 exx, Airmadidi, Gn. Klabat, 01°26.505'N 125°00.636'E, 923 m, sifted, 16-V-2012; 5 exx, Airmadidi, Gn. Klabat, 01°26.619'N 125°00.805'E, 1018 m, sifted, 17-V-2012; 3 exx, ARC2789 (GenBank # MK260291), Airmadidi, Gn. Klabat, 01°26.635'N 125°00.823'E, 1031 m, sifted, 16-V-2012; 2 exx, Airmadidi, Gn. Klabat, 01°26.988'N 125°01.244'E, 1392 m, sifted, 17-V-2012; 1 ex, ARC0327 (GenBank # MK260290), Bitung, Gn. Dua Saudara, 01°29.417'N 125°09.150'E, sifted, 25-VI-2006.

#### Distribution.

N-Sulawesi Prov. (Tomohon, Mt Klabat, Mt Dua Saudara). Elevation 890–1390 m.

#### Biology.

On foliage in montane forests, rarely in leaf litter.

#### Etymology.

This epithet is a Latinized adjective based on the town of Manado.

#### Notes.

*Trigonopterusmanadensis* Riedel, sp. n. was coded as “*Trigonopterus* sp. 396”.

### 48. Trigonopterus mangkutanensis

Taxon classificationAnimaliaColeopteraCurculionidae

Riedel sp. n.

http://zoobank.org/DAEA1F6F-B019-478C-B122-0E22E0B91730

#### Diagnostic description.

**Figure 48.**
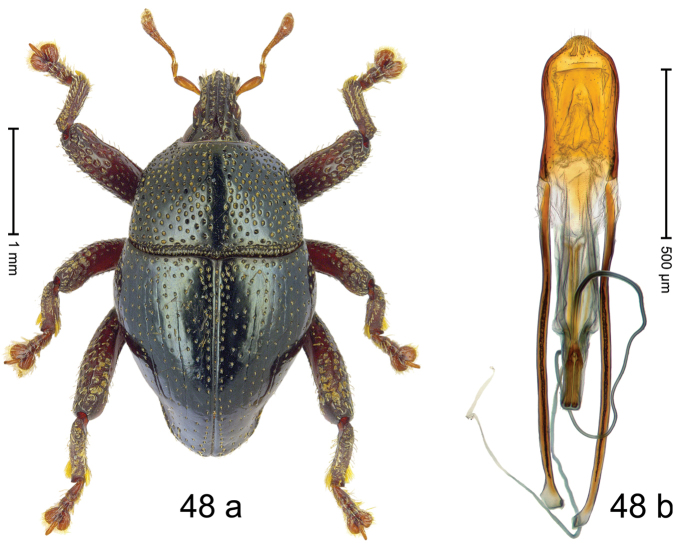
*Trigonopterusmangkutanensis* Riedel, sp. n., holotype; **a** habitus **b** penis.

***Holotype***, male (Fig. 48a). Length 2.98 mm. Color of antennae and legs ferruginous; remainder black with slight bluish luster. Body subovate; in dorsal aspect with weak constriction between pronotum and elytron, in profile dorsally convex. Rostrum dorsally with median carina and pair of distinct sublateral ridges; interspaces with sparse rows of erect piliform scales; epistome short, posteriorly with transverse ridge, with median denticle and pair of larger lateral denticles. Eyes with dorsal margin bordered by furrow. Pronotum with disk densely punctate, partly reticulate; median line glabrous. Elytra irregularly punctate with small punctures, subapically with few deeper punctures; some striae marked by fine hairlines. Femora edentate; anteroventral ridge distinct, simple. Metafemur with dorsoposterior ridge denticulate, subapically with stridulatory patch, anterior surface coarsely rugose-punctate, each puncture with slender subrecumbent to suberect scale. Abdominal ventrites 1–2 concave, subglabrous, microreticulate, with sparse erect scales; ventrite 5 flat, coarsely punctate, sparsely setose. Penis (Fig. 48b) with body subparallel, apex subangulate, medially sparsely setose; apodemes 2.6 × as long as body of penis; transfer apparatus flagelliform, 1.7 × longer than body of penis; ductus ejaculatorius without bulbus.

#### Material examined.

***Holotype*** (MZB): ARC3234 (EMBL # LN884978), S-Sulawesi Prov., Mangkutana, 02°20.203'S 120°46.878'E, 903 m, sifted, 14-V-2013.

#### Distribution.

S-Sulawesi Prov. (Mangkutana). Elevation ca. 900 m.

#### Biology.

In leaf litter of montane forest.

#### Etymology.

This epithet is a Latinized adjective based on the type locality.

#### Notes.

*Trigonopterusmangkutanensis* Riedel, sp. n. was coded as “*Trigonopterus* sp. 470”.

### 49. Trigonopterus matalibaruensis

Taxon classificationAnimaliaColeopteraCurculionidae

Riedel sp. n.

http://zoobank.org/28976E8E-A34B-466D-970D-5004ACB60FBB

#### Diagnostic description.

**Figure 49.**
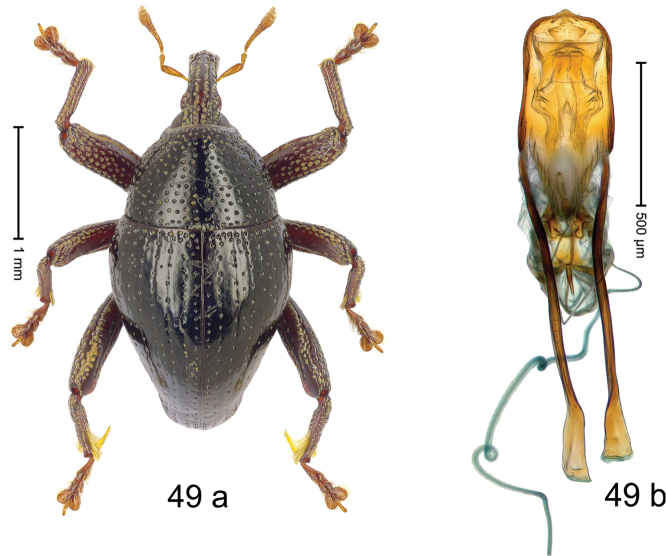
*Trigonopterusmatalibaruensis* Riedel, sp. n., holotype; **a** habitus **b** penis.

***Holotype***, male (Fig. 49a). Length 2.81 mm. Color of antennae ferruginous; legs dark ferruginous; remainder black. Body subovate; in profile dorsally convex. Rostrum dorsally with median and pair of submedian costae separated by row of coarse punctures; with sparse rows of suberect setae; epistome sparsely punctate, with minute median denticle. Pronotum with disk densely punctate; median line impunctate; interspaces between punctures subglabrous, subequal to punctures´ diameter. Elytra with striae marked by rows of small punctures and hairlines; along basal margin with transverse row of denser punctures; stria 8 along humerus with larger punctures; intervals flat, subglabrous, with few punctures. Femora edentate. Metafemur with dorsoposterior edge denticulate; subapically with stridulatory patch. Posterior face of metatibia in apical 1/2 with dense long setae, subapically forming inward curved brush. Abdominal ventrites 1–2 concave, microreticulate, with erect, slender scales; ventrite 5 concave, subglabrous, with sparse punctures. Penis (Fig. 49b) with sides of body subparallel, converging to subacute apex; apodemes 2.8 × as long as body of penis; transfer apparatus flagelliform, supported by complex sclerites, directed basad in repose, 1.5 × longer than body of penis; ductus ejaculatorius with indistinct bulbus. ***Intraspecific variation***. Length 2.65–2.88 mm. Female rostrum slender, dorsally subglabrous, with rows of small punctures; epistome simple. Female metatibia subapically only with small patch of setae, without curved brushes. Female abdominal ventrites 1–2 flat, nude; ventrite 5 flat.

#### Material examined.

***Holotype*** (MZB): ARC2889 (GenBank # MK260235), N-Sulawesi Prov., Kotamobagu, Matalibaru, 00°32.445'N 124°14.185'E, 956 m, beaten, 20-V-2012. ***Paratypes*** (SMNK): 2 exx, ARC2890 (GenBank # MK260236), ARC2891 (GenBank # MK260237), same data as holotype.

#### Distribution.

N-Sulawesi Prov. (Kotamobagu). Elevation 960 m.

#### Biology.

On foliage in montane forests.

#### Etymology.

This epithet is a Latinized adjective based on the town Matalibaru which is close to the type locality.

#### Notes.

*Trigonopterusmatalibaruensis* Riedel, sp. n. was coded as “*Trigonopterus* sp. 381”.

### 50. Trigonopterus mesai

Taxon classificationAnimaliaColeopteraCurculionidae

Riedel sp. n.

http://zoobank.org/5DD35D8B-BEA7-448E-BF70-068AD2F93950

#### Diagnostic description.

**Figure 50.**
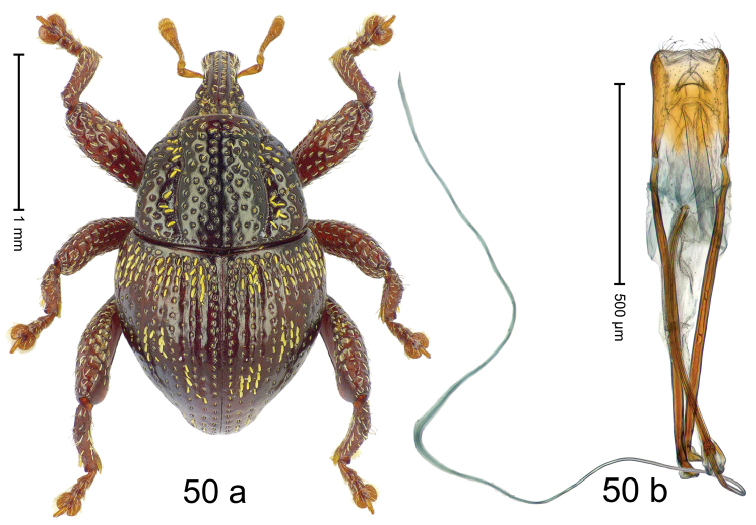
*Trigonopterusmesai* Riedel, sp. n., holotype; **a** habitus **b** penis.

***Holotype***, male (Fig. 50a). Length 2.04 mm. Color of antennae and legs ferruginous; elytra dark ferruginous; remainder black. Body subovate; in dorsal aspect with moderate constriction between pronotum and elytron; in profile dorsally convex. Rostrum dorsally with median and pair of submedian ridges; intervening furrows with sparse, erect scales; epistome short, posteriorly with transverse ridge. Pronotum with lateral edges weakly converging, with weak subapical constriction; disk with pair of distinct longitudinal impressions, lined with sparse yellow almond-shaped scales; medially broadly swollen, densely punctate with coarse punctures, with narrow subglabrous midline. Elytra densely irregularly punctate; striae 1–3 weakly impressed; with sparse yellow recumbent scales, especially in basal 1/3 and near middle. Femora dentate, with acute tooth; anteroventral ridge of meso- and metafemur crenate. Metafemur with dorsoposterior edge denticulate; subapically with stridulatory patch. Dorsoposterior edge of tibiae subbasally denticulate. Metatibia weakly curved. Abdominal ventrites 1–2 concave, subglabrous, with few coarse punctures; ventrite 5 at middle weakly concave, with sparse coarse punctures and sparse erect setae. Penis (Fig. 50b) with sides of body subparallel, apex subtruncate, with sparse setae; apodemes 3.4 × as long as body of penis; transfer apparatus flagelliform, 3.0 × longer than body of penis; ductus ejaculatorius with indistinct bulbus. ***Intraspecific variation***. Length 1.92–2.20 mm. Elytra with or without yellow scales. Female rostrum slender, dorsally in apical 1/2 subglabrous, with submedian row of punctures and with pair of sublateral furrows.

#### Material examined.

***Holotype*** (MZB): ARC3267 (EMBL # LN884981), C-Sulawesi Prov., Luwuk, Salodi, Gn. Taluanjang, 00°49.202'S 122°52.395'E, 702 m, sifted, 21-V-2013. ***Paratypes*** (MZB, SMNK): C-Sulawesi Prov.: 6 exx, ARC3268 (GenBank # MK260467), ARC3269 (GenBank # MK260466), ARC6020 (GenBank # MK260465), ARC6021 (GenBank # MK260464), same data as holotype; 7 exx, Luwuk, Salodi, Gn. Taluanjang, 00°49.307'S 122°52.322'E, 760 m, sifted, 21-V-2013.

#### Distribution.

C-Sulawesi Prov. (Luwuk). Elevation 700–760 m.

#### Biology.

In leaf litter of montane forest.

#### Etymology.

This epithet is a patronym in honor of “Mesa”, Raden Pramesa Narakusumo, curator of Coleoptera at the Indonesian National Zoological Collection. An invariable genitive.

#### Notes.

*Trigonopterusmesai* Riedel, sp. n. was coded as “*Trigonopterus* sp. 490”.

### 51. Trigonopterus minahassae

Taxon classificationAnimaliaColeopteraCurculionidae

Riedel sp. n.

http://zoobank.org/73330AB6-235C-466F-927B-306B77DD061B

#### Diagnostic description.

**Figure 51.**
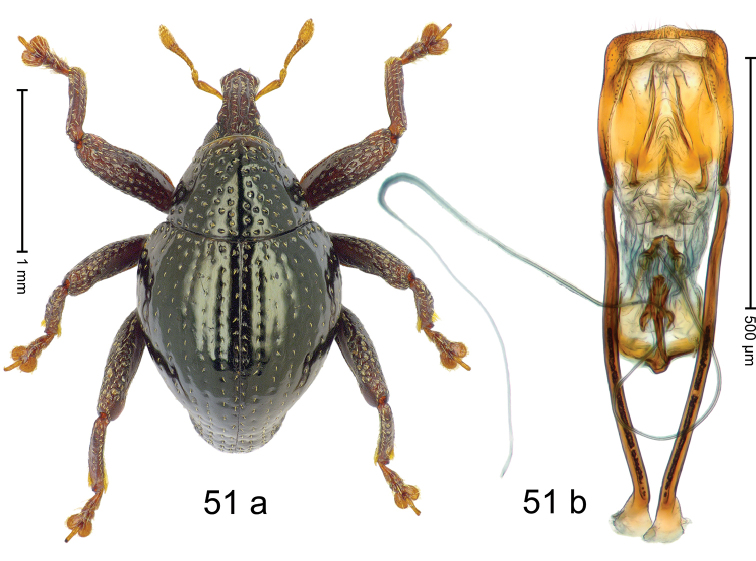
*Trigonopterusminahassae* Riedel, sp. n., holotype; **a** habitus **b** penis.

***Holotype***, male (Fig. 51a). Length 2.00 mm. Color of antennae ferruginous; legs and head dark ferruginous; remainder black with weak bronze luster. Body subovate; in dorsal aspect with weak constriction between pronotum and elytron; in profile dorsally convex. Rostrum dorsally with median and pair of submedian ridges; intervening furrows with sparse rows of setae; epistome posteriorly with indistinct transverse ridge. Pronotum with disk punctate; median line weakly costate; interspaces between punctures subglabrous, subequal to or smaller than punctures´ diameter. Elytra with striae marked by small punctures; intervals flat, subglabrous; basal margin bordered by transverse row of deeper punctures joining stria 8; along humerus externally bordered by costa. Femora edentate. Metafemur with dorsoposterior edge weakly denticulate; subapically with stridulatory patch. Abdominal ventrites 1–2 impressed, weakly concave, subglabrous; ventrite 5 at middle with shallow pit, subglabrous. Penis (Fig. 51b) with sides of body subparallel, apex subangulate, with sparse setae; apodemes 2.6 × as long as body; transfer apparatus complex, anchor-shaped; ductus ejaculatorius with indistinct bulbus. ***Intraspecific variation***. Length 2.10–2.28 mm. Female rostrum slender, dorsally subglabrous, sparsely punctate; in basal 1/3 with median and pair of submedian costae; epistome simple.

#### Material examined.

***Holotype*** (MZB): ARC2791 (GenBank # MK260250), N-Sulawesi Prov., Airmadidi, Gn. Klabat, 01°26.635'N 125°00.823'E, 1031 m, sifted, 16-V-2012. ***Paratypes*** (MZB, SMNK): N-Sulawesi Prov.: 7 exx, ARC0319 (GenBank # MK260253), ARC0320 (GenBank # MK260251), ARC0321 (GenBank # MK260254), ARC0322 (GenBank # MK260255), Bitung, Gn. Dua Saudara, 01°29.417'N 125°09.150'E, 907 m, 24-VI-2006; 2 exx, Airmadidi, Gn. Klabat, 01°26.430'N 125°00.622'E, 889 m, sifted, 17-V-2012; 7 exx, ARC2793 (GenBank # MK260252), same data as holotype; 4 exx, Airmadidi, Gn. Klabat, 01°26.505'N 125°00.636'E, 923 m, sifted, 16-V-2012; 6 exx, Airmadidi, Gn. Klabat, 01°26.619'N 125°00.805'E, 1018 m, sifted, 17-V-2012.

#### Distribution.

N-Sulawesi Prov. (Mt Dua Saudara, Mt Klabat). Elevation 890–1030 m.

#### Biology.

In leaf litter of montane forest.

#### Etymology.

This epithet is based on Minahassa region of North Sulawesi. An invariable genitive.

#### Notes.

*Trigonopterusminahassae* Riedel, sp. n. was coded as “*Trigonopterus* sp. 386”.

### 52. Trigonopterus moatensis

Taxon classificationAnimaliaColeopteraCurculionidae

Riedel sp. n.

http://zoobank.org/BAC62C84-DC02-4E84-87E4-3B5B895AF5D5

#### Diagnostic description.

**Figure 52.**
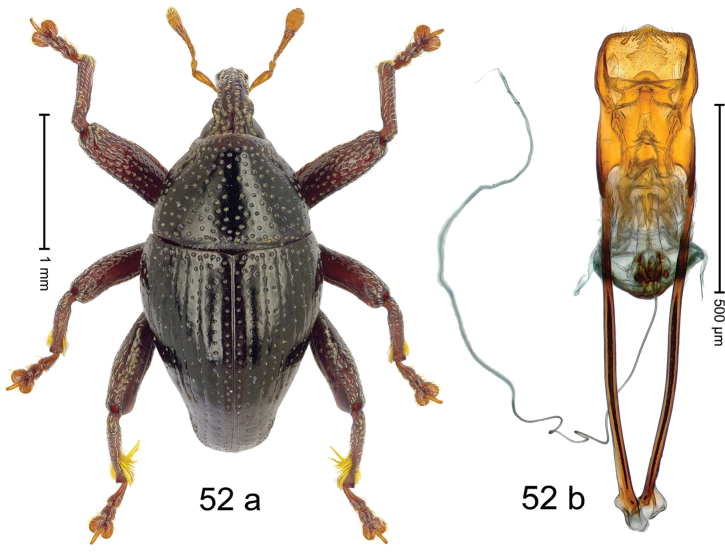
*Trigonopterusmoatensis* Riedel, sp. n., holotype; **a** habitus **b** penis.

***Holotype***, male (Fig. 52a). Length 2.48 mm. Color of antennae ferruginous; legs dark ferruginous; remainder black. Body subovate; in profile dorsally convex. Rostrum dorsally with median costa and pair of submedian costae, in apical 1/2 continued as fine ridges; with sparse rows of suberect piliform scales; epistome sparsely punctate, with minute median denticle. Pronotum with disk densely punctate; median line sparsely punctate; interspaces between punctures subglabrous, subequal to punctures´ diameter. Elytra with striae marked by rows of small punctures and hairlines except stria 1 without hairline; along basal margin with transverse row of denser punctures; stria 8 along humerus with larger punctures; intervals flat, subglabrous, with few interspersed punctures. Femora edentate. Metafemur with dorsoposterior edge denticulate; subapically with stridulatory patch. Posterior face of metatibia in apical 1/2 with dense long setae, subapically forming inwards curved brush. Abdominal ventrites 1–2 concave, subglabrous; ventrite 5 concave, subglabrous. Penis (Fig. 52b) with sides of body weakly constricted near middle; apex subangulate, with sparse setae; apodemes 2.2 × as long as body; transfer apparatus complex; ductus ejaculatorius with indistinct bulbus. ***Intraspecific variation***. Length 2.40–2.68 mm.

#### Material examined.

***Holotype*** (MZB): ARC2908 (GenBank # MK260238), N-Sulawesi Prov., Kotamobagu, Modoinding, Lake Moat area, 00°42.862'N 124°28.356'E, 1024 m, beaten, 19-V-2012. ***Paratypes*** (MZB, SMNK): 4 exx, ARC2907 (GenBank # MK260240), ARC2909 (GenBank # MK260239), same data as holotype.

#### Distribution.

N-Sulawesi Prov. (Lake Moat). Elevation ca. 960 m.

#### Biology.

On foliage in montane forests.

#### Etymology.

This epithet refers to Lake Moat which is close to the type locality. A variable Latinized adjective.

#### Notes.

*Trigonopterusmoatensis* Riedel, sp. n. was coded as “*Trigonopterus* sp. 382”.

### 53. Trigonopterus modoindingensis

Taxon classificationAnimaliaColeopteraCurculionidae

Riedel sp. n.

http://zoobank.org/979D045D-8B3D-4496-882F-F89FD34712F4

#### Diagnostic description.

**Figure 53.**
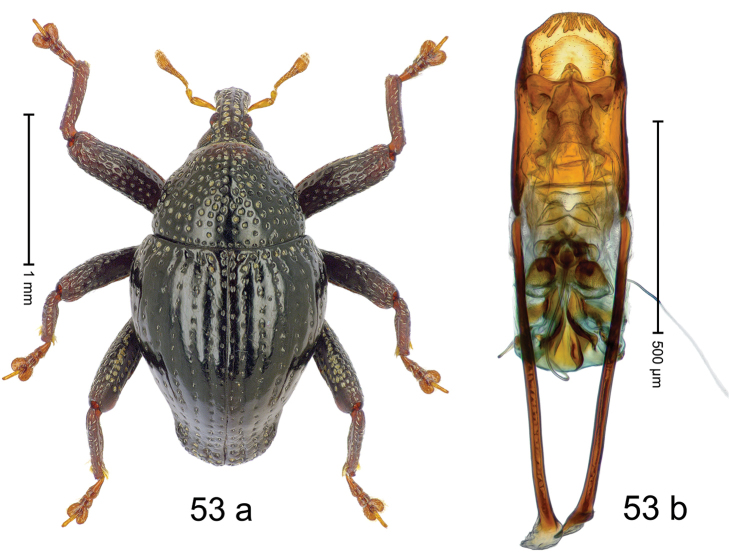
*Trigonopterusmodoindingensis* Riedel, sp. n., holotype; **a** habitus **b** penis.

***Holotype***, male (Fig. 53a). Length 2.24 mm. Color of antennae ferruginous; tarsi and tibiae dark ferruginous; remainder black. Body subovate; in dorsal aspect with distinct constriction between pronotum and elytron; in profile dorsally convex, with weak constriction between pronotum and elytron. Rostrum with lateral flanges in front of eyes; dorsally with weak median and pair of submedian ridges; intervening furrows with rows of coarse punctures and rows of thin setae; epistome short, posteriorly with transverse, angulate ridge. Pronotum with disk densely punctate with coarse punctures; interspaces between punctures subglabrous, reticulate; median line weakly costate. Elytra with striae weakly impressed, with rows of small punctures; along base weakly transversely impressed, with row of coarse punctures; stria 8 along humerus with large, coarse punctures; intervals subglabrous. Femora edentate; anteroventral ridge distinct; anterior surface coarsely punctate, each puncture with recumbent piliform scale. Metafemur with dorsoposterior edge denticulate; subapically with stridulatory patch. Abdominal ventrites 1–2 flat, subglabrous, with lateral rim; ventrite 5 with shallow impression, subglabrous, near base punctate. Penis (Fig. 53b) with sides weakly converging, apex subangulate, medially rounded, without setae; apodemes 1.6 × as long as body of penis; transfer apparatus symmetrical, complex; ductus ejaculatorius without bulbus. ***Intraspecific variation***. Length 2.24–2.40 mm.

#### Material examined.

***Holotype*** (MZB): ARC2897 (GenBank # MK260627), N-Sulawesi Prov., Kotamobagu, Modoinding, Lake Moat area, 00°42.862'N 124°28.356'E, 1024 m, sifted, 19-V-2012. ***Paratypes*** (SMNK): 3 exx, ARC2898 (GenBank # MK260626), ARC2899 (GenBank # MK260625), same data as holotype.

#### Distribution.

N-Sulawesi Prov. (Lake Moat). Elevation ca. 1020 m.

#### Biology.

In leaf litter of montane forest.

#### Etymology.

This epithet is a Latinized adjective based on the type locality.

#### Notes.

*Trigonopterusmodoindingensis* Riedel, sp. n. was coded as “*Trigonopterus* sp. 580”.

### 54. Trigonopterus nanus

Taxon classificationAnimaliaColeopteraCurculionidae

Riedel sp. n.

http://zoobank.org/A111977C-D358-4F16-BDAB-23B511702426

#### Diagnostic description.

**Figure 54.**
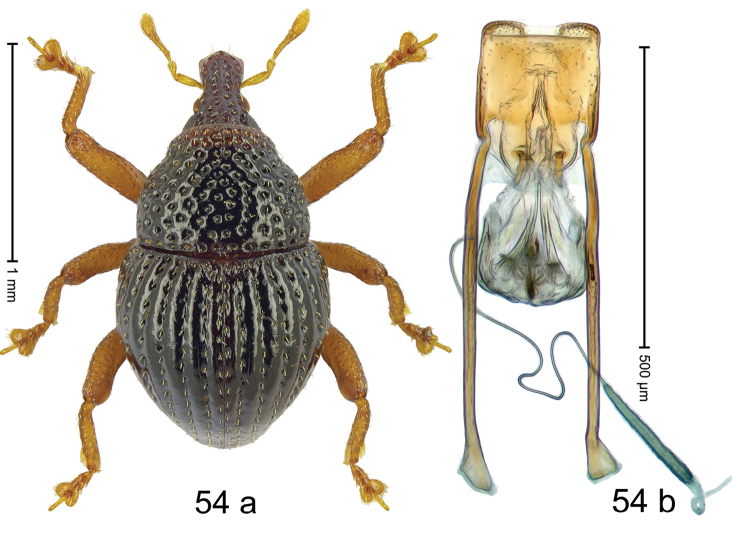
*Trigonopterusnanus* Riedel, sp. n., holotype; **a** habitus **b** penis.

***Holotype***, male (Fig. 54a). Length 1.49 mm. Color of antennae and legs ferruginous; remainder black. Body subovate; in dorsal aspect with distinct constriction between pronotum and elytron; in profile with weak constriction between pronotum and elytron. Rostrum dorsally punctate-rugose, microreticulate, with median ridge and rows of coarse punctures, each with suberect seta; epistome short, posteriorly with transverse, subangulate ridge. Pronotum with weak subapical constriction; disk densely punctate with coarse punctures; each puncture with single seta. Elytra with striae impressed, containing punctures with short suberect setae; intervals costate, subglabrous. Femora edentate; surface dull, microreticulate. Metafemur subapically with stridulatory patch. Abdominal ventrites 1–2 concave, subglabrous, microreticulate; ventrite 5 flat, coarsely punctate, microreticulate. Penis (Fig. 54b) with sides of body subparallel; apex subtruncate, with few short setae, medially membranous; apodemes 3.3 × as long as body of penis; endophallus with pair of elongate sclerites; transfer apparatus short flagelliform, subequal to body of penis; ductus ejaculatorius with distinct bulbus. ***Intraspecific variation***. Length 1.35–1.59 mm. Female rostrum dorsally subglabrous, punctate; epistome indistinct.

#### Material examined.

***Holotype*** (MZB): ARC3201 (GenBank # MK260457), S-Sulawesi Prov., Gn. Lompobattang, Malakaji, Parambintolo, 05°23.545'S 119°55.517'E, 1803 m, sifted, 27-IV-2013. ***Paratypes*** (MZB, SMNK): 10 exx, ARC3202 (GenBank # MK260459), ARC3203 (GenBank # MK260456), ARC3204 (GenBank # MK260458), same data as holotype.

#### Distribution.

S-Sulawesi Prov. (Gn. Lompobattang). Elevation ca. 1800 m.

#### Biology.

In leaf litter of montane forest.

#### Etymology.

This epithet is the Latin noun *nanus* (dwarf) and refers to the small body size of this species.

#### Notes.

*Trigonopterusnanus* Riedel, sp. n. was coded as “*Trigonopterus* sp. 486”.

### 55. Trigonopterus nitidulus

Taxon classificationAnimaliaColeopteraCurculionidae

Riedel sp. n.

http://zoobank.org/A7FA7A13-766C-4E42-B309-B76696D20D9C

#### Diagnostic description.

**Figure 55.**
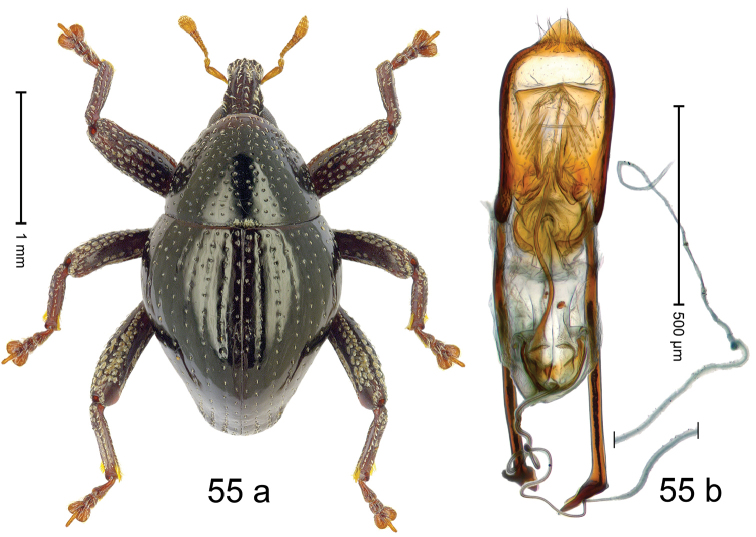
*Trigonopterusnitidulus* Riedel, sp. n., holotype; **a** habitus **b** penis.

***Holotype***, male (Fig. 55a). Length 2.50 mm. Color of antennae, tibiae, and tarsi ferruginous; remainder black. Body subovate; in profile dorsally convex. Rostrum dorsally with median and pair of submedian ridges; behind epistome scabrous; with sparse rows of erect, subclavate scales; epistome posteriorly with transverse, angulate ridge. Pronotum with disk densely punctate; dorsally punctures small; interspaces between punctures subglabrous; laterally punctures coarse. Elytra with striae marked by small punctures and weakly impressed lines, subapically more deeply entrenched; stria 8 along humerus with large, coarse punctures; intervals subglabrous; subapically profile of ventral margin crenate. Femora edentate; anterior surface coarsely punctate, each puncture with recumbent scale. Metafemur with dorsoposterior edge denticulate; subapically with stridulatory patch. Abdominal ventrites 1–2 concave, subglabrous; ventrite 5 flat, microgranulate, dull. Penis (Fig. 55b) with sides of body subparallel, converging to subtriangular apex; subapically with sparse setae; dorsally with patches of setae pointing to midline; apodemes 1.4 × as long as body of penis; transfer apparatus flagelliform, ca. 1.2 × longer than body; ductus ejaculatorius with indistinct bulbus. ***Intraspecific variation***. Length 2.33–2.58 mm. Female rostrum with dorsal ridges only at base, anteriorly subglabrous, with submedian rows of punctures and sublateral furrows; epistome simple. Female abdominal ventrite 5 sparsely punctate, sublaterally with sparse erect scales.

#### Material examined.

***Holotype*** (MZB): ARC2797 (GenBank # MK260301), N-Sulawesi Prov., Airmadidi, Gn. Klabat, 01°26.368'N 125°00.609'E to 01°26.505'N 125°00.636'E, 872–923 m, beaten, 16-V-2012. ***Paratypes*** (MZB, SMNK): N-Sulawesi Prov.: 1 ex, ARC2798 (GenBank # MK260302), same data as holotype; 1 ex, Airmadidi, Gn. Klabat, 01°26.430'N 125°00.622'E, 889 m, sifted, 17-V-2012; 1 ex, Airmadidi, Gn. Klabat, 01°26.505'N 125°00.636'E, 923 m, sifted, 16-V-2012; 1 ex, Airmadidi, Gn. Klabat, 01°26.368'N 125°00.609'E to 01°26.505'N 125°00.636'E, 872–923 m, 16-V-2012, beaten; 4 exx, Airmadidi, Gn. Klabat, 01°26.505'N 125°00.636'E to 01°26.635'N 125°00.823'E, 923–1031 m, 16-V-2012, beaten; 1 ex, ARC0349 (GenBank # HG939578), Bitung, Gn. Dua Saudara, 750 m, 24-VI-2006, sifted.

#### Distribution.

N-Sulawesi Prov. (Mt Dua Saudara, Mt Klabat). Elevation 750–920 m.

#### Biology.

Uncertain if more frequently on foliage or in leaf litter.

#### Etymology.

This epithet is the diminutive form of the Latin adjective *nitidus*, -*a*, -*um* (polished) and refers to the species´ small size and its elytral sculpture.

#### Notes.

*Trigonopterusnitidulus* Riedel, sp. n. was coded as “*Trigonopterus* sp. 400”.

### 56. Trigonopterus obelix

Taxon classificationAnimaliaColeopteraCurculionidae

Riedel sp. n.

http://zoobank.org/19308561-E86B-4127-BD3D-0E3BF297E67A

#### Diagnostic description.

**Figure 56.**
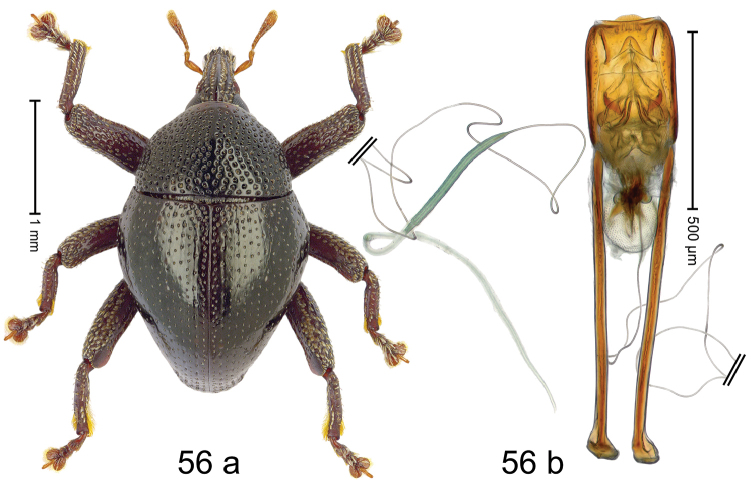
*Trigonopterusobelix* Riedel, sp. n., holotype; **a** habitus **b** penis.

***Holotype***, male (Fig. 56a). Length 2.65 mm. Color of antennae ferruginous; legs dark ferruginous; remainder black. Body broadly subovate; in dorsal aspect with weak constriction between pronotum and elytron; in profile dorsally convex. Rostrum dorsally with median and pair of submedian carinae; intervening furrows each with row of erect scales; epistome with one median denticle and two pairs of submedian / sublateral denticles. Pronotum with disk densely punctate with coarse, almost confluent punctures; each puncture containing a single seta; narrow interspaces subglabrous; medially with indistinct costa. Elytra densely irregularly punctate with small punctures; striae indistinct; interspaces between punctures subglabrous; striae 7–9 with larger punctures; stria 8 along humerus with 5 large, coarse punctures. Femora edentate; anterior surface densely coarsely punctate, each puncture with suberect scale. Metafemur with dorsoposterior edge denticulate; subapically with stridulatory patch. Posterior face of metatibia in apical 1/2 with dense yellowish setae. Abdominal ventrites 1–2 concave, subglabrous, microreticulate, with sparse coarse punctures, sublaterally each with blunt tooth; in profile ventrite 2 subangularly projecting; ventrite 5 with median impression bordered by submedian carinae; medially punctate, interspaces subglabrous. Penis (Fig. 56b) with sides of body subparallel; apex subangulate medially rounded, without setae; apodemes 2.9 × as long as body; transfer apparatus complex, symmetrical; ductus ejaculatorius extremely long, with distinct bulbus. ***Intraspecific variation***. Length 2.48–2.75 mm. Female rostrum slender, dorsally in apical 1/2 subglabrous, with submedian row of punctures and sublateral pair of furrows; in basal 1/2 with median and pair of submedian glabrous costae; epistome simple. Female abdominal ventrite 5 flat.

#### Material examined.

***Holotype*** (MZB): ARC2817 (GenBank # MK260203), S-Sulawesi Prov., Pc. Palopo, Gn. Sampuna, 02°56.539'S 120°05.320'E to 02°56.545'S 120°05.595'E, 1038–1101 m, beaten, 29-V-2012. ***Paratypes*** (MZB, SMNK, ZSM): S-Sulawesi Prov., Pc. Palopo, Gn. Sampuna: 46 exx, ARC2818 (GenBank # MK260202), ARC2819 (EMBL # LN884932), same data as holotype; 18 exx, Pc. Palopo, Gn. Sampuna, 02°56.539'S 120°05.320'E, 1038 m, sifted, 29-V-2012; 15 exx, 02°56.539'S 120°05.320'E to 02°56.545'S 120°05.595'E, 1038–1101 m, beaten, 02-V-2013; 11 exx, ARC2840 (GenBank # MK260201), ARC2841 (GenBank # MK260200), 02°56.545'S 120°05.595'E, 1101 m, sifted, 29-V-2012; 23 exx, 02°56.545'S 120°05.595'E, 1101 m, sifted, 02-V-2013.

#### Distribution.

S-Sulawesi Prov. (Pc. Palopo). Elevation 1040–1100 m.

#### Biology.

On foliage in montane forests, but also in leaf litter.

#### Etymology.

This epithet is based on a character of the French Asterix comics. It is a noun in apposition.

#### Notes.

*Trigonopterusobelix* Riedel, sp. n. was coded as “*Trigonopterus* sp. 376”. This species is closely related to *T.posoensis* Riedel, sp. n., from which it differs by ca. 15.0–15.5% p-distance of *cox1*, and by the rounded profile of abdominal ventrite 2.

### 57. Trigonopterus ovalipunctatus

Taxon classificationAnimaliaColeopteraCurculionidae

Riedel sp. n.

http://zoobank.org/C80D8DE7-A6C0-409E-A6FF-9FAC6456B112

#### Diagnostic description.

**Figure 57.**
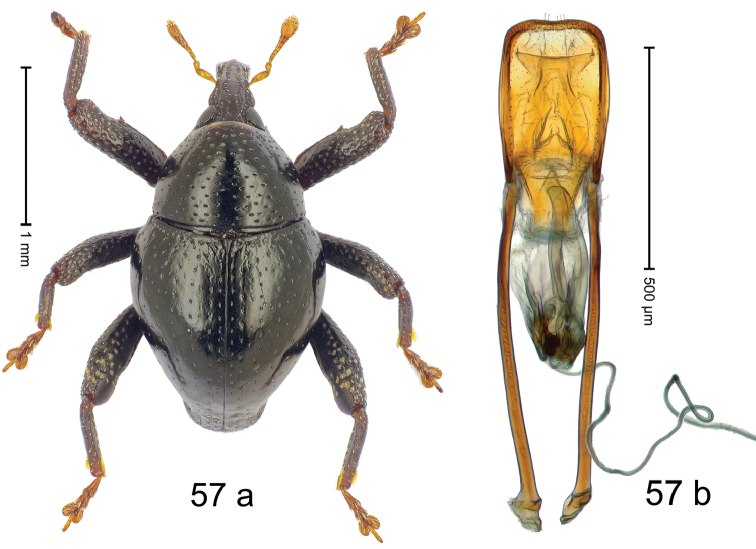
*Trigonopterusovalipunctatus* Riedel, sp. n., holotype; **a** habitus **b** penis.

***Holotype***, male (Fig. 57a). Length 2.03 mm. Color of antennae and tarsi ferruginous; remainder black. Body subovate; in dorsal aspect with weak constriction between pronotum and elytron; in profile dorsally convex, with very weak constriction between pronotum and elytron. Rostrum dorsally punctate-rugose; median costa indistinct, with pair of irregular submedian ridges; epistome indistinct, subglabrous, with weak median denticle. Pronotum with ovate punctures of transverse orientation; interspaces subglabrous. Elytra with striae marked by rows of small punctures; sutural interval with additional row, other intervals subglabrous, with sparse minute punctures. Femora dentate with small tooth; anteroventral ridge of meso-and metafemur weakly crenate; anterior surface densely punctate, microreticulate, each puncture with short recumbent seta. Metafemur subapically with stridulatory patch. Abdominal ventrites 1–2 concave, subglabrous, with sparse punctures; ventrite 5 flat, densely punctate, markedly microreticulate. Penis (Fig. 57b) with body subparallel, apex rounded; apodemes 2.4 × as long as body of penis; transfer apparatus short spiniform; ductus ejaculatorius with indistinct bulbus. ***Intraspecific variation***. Length 2.03–2.22 mm. Female rostrum subglabrous, with sparse minute punctures.

#### Material examined.

***Holotype*** (MZB): ARC3126 (GenBank # MK260422), C-Sulawesi Prov., Pendolo, Gn. Sampuraga, 02°15.407'S 120°47.051'E, 1406 m, beaten, 16-V-2013. ***Paratypes*** (MZB, SMNK): C-Sulawesi Prov.: 4 exx, ARC3125 (EMBL # LN884959), ARC3127 (GenBank # MK260421), same data as holotype; 2 exx, ARC6069 (EMBL # MK260420), ARC6070 (EMBL # MK260423), Pendolo, Gn. Sampuraga, 02°15.407'S 120°47.051'E, 1406 m, beaten, 31-V-2012.

#### Distribution.

C-Sulawesi Prov. (Pendolo). Elevation ca. 1410 m.

#### Biology.

On foliage in montane forests.

#### Etymology.

This epithet is an adjectival combination of the Latin adjective *ovalis*, -*e* (ovate) and the participle *punctatus* (punctate). It refers to the peculiar shape of punctures on the pronotum.

#### Notes.

*Trigonopterusovalipunctatus* Riedel, sp. n. was coded as “*Trigonopterus* sp. 476”.

### 58. Trigonopterus ovatulus

Taxon classificationAnimaliaColeopteraCurculionidae

Riedel sp. n.

http://zoobank.org/A6F6DE94-F34A-4F06-9584-E0A1C149AA36

#### Diagnostic description.

**Figure 58.**
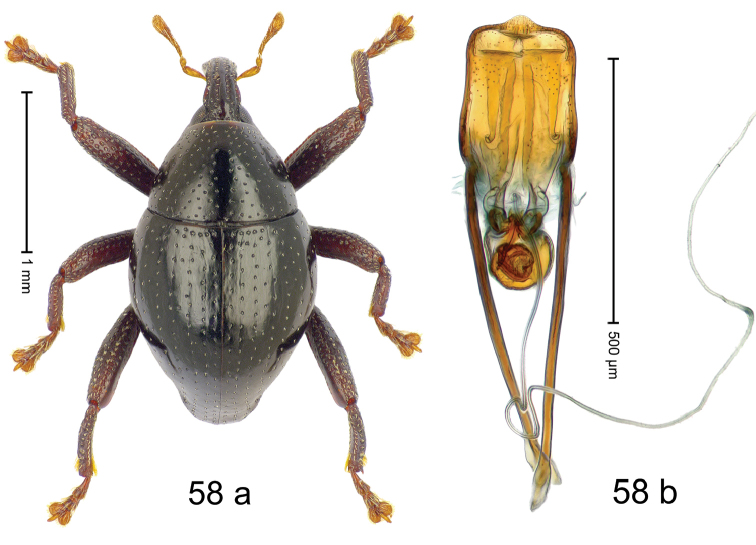
*Trigonopterusovatulus* Riedel, sp. n., holotype; **a** habitus **b** penis.

***Holotype***, male (Fig. 58a). Length 1.98 mm. Color of antennae and tarsi ferruginous; tibiae and femora dark ferruginous; remainder black. Body subovate; in dorsal aspect with weak constriction between pronotum and elytron; in profile dorsally convex. Rostrum dorsally with median costa and pair of submedian ridges, weakly microreticulate; intervening furrows with sparse rows of piliform, recumbent scales; epistome in apical 1/3 indistinct, weakly swollen, subglabrous, sparsely setose. Pronotum with disk punctate with small punctures; interspaces subglabrous, weakly microreticulate. Elytra with striae marked by rows of small punctures; intervals subglabrous; near base with few additional, slightly larger punctures. Femora with anteroventral ridge distinct, ending in apical 1/3 with small blunt tooth; anterior surface densely punctate, microreticulate, each puncture with narrow recumbent scale. Metafemur subapically with extensive stridulatory patch. Metatibia with dorsal edge weakly denticulate, with small supra-uncal spine. Abdominal ventrites 1–2 weakly concave, subglabrous; ventrite 5 with shallow impression, densely punctate, covered with suberect scales, scales larger sublaterally. Penis (Fig. 58b) with body subparallel, apex with subangulate extension, without setae; apodemes 2.3 × as long as body of penis; transfer apparatus complex, asymmetrical, with subrotund capsule; ductus ejaculatorius without bulbus. ***Intraspecific variation***. Length 1.75–2.34 mm. Female rostrum dorsally with submedian row of punctures, with sublateral furrow. Femora with anteroventral ridge ending with small blunt tooth or rounded.

#### Material examined.

***Holotype*** (MZB): ARC2990 (GenBank # MK260608), S-Sulawesi Prov., Tanah Toraja, Rantepao, Gn. Karre (= Gn. Wokim), 02°59.021'S 120°02.523'E, 1456 m, beaten, 06-V-2013. ***Paratypes*** (MZB, SMNK): S-Sulawesi Prov.: 10 exx, ARC2991 (GenBank # MK260607), ARC2992 (GenBank # MK260624), ARC3000 (GenBank # MK260611), same data as holotype; 1 ex, ARC6027 (GenBank # MK260609), Tanah Toraja, Rantepao, Gn. Karre (= Gn. Wokim), 02°59.021'S 120°02.523'E, 1456 m, sifted, 06-V-2013; 2 exx, Tanah Toraja, Rantepao, Gn. Karre (= Gn. Wokim), 02°59.013'S 120°02.251'E, 1423 m, beaten, 06-V-2013; 3 exx, ARC6037 (GenBank # MK260621), ARC6038 (GenBank # MK260619), ARC6039 (GenBank # MK260618), Tanah Toraja, Rantepao, Gn. Karre (= Gn. Wokim), 02°58.846'S 120°02.158'E, 1396 m, beaten, 06-V-2013; C-Sulawesi Prov.: 46 exx, ARC2856 (GenBank # MK260615), ARC2857 (GenBank # MK260612), ARC2858 (GenBank # MK260616), ARC2859 (GenBank # MK260617), ARC2862 (GenBank # MK260622), Pendolo, Gn. Sampuraga, 02°12.476'S 120°45.506'E, 1050 m, beaten, 31-V-2012; 50 exx, ARC6044 (GenBank # MK260623), ARC6045 (GenBank # MK260620), Pendolo, Gn. Sampuraga, 02°12.165'S 120°45.567'E to 02°12.308'S 120°45.544'E, beaten, 934–1011 m, 13-V-2013; 3 exx, ARC3100 (GenBank # MK260614), ARC3101 (GenBank # MK260613), ARC3102 (GenBank # MK260610), Pendolo, Boe, 02°05.405'S 120°38.551'E to 02°05.446'S 120°38.519'E, 750–950 m, beaten, 15-V-2013.

#### Distribution.

S-Sulawesi Prov. (Tanah Toraja); C-Sulawesi Prov. (Pendolo). Elevation 910–1450 m.

#### Biology.

On foliage in montane forests.

#### Etymology.

This epithet is the diminutive form of the Latin adjective *ovatus*, -*a*, -*um* (egg-shaped) and refers to the species´ body form and small size. It is a variable adjective as well.

#### Notes.

*Trigonopterusovatulus* Riedel, sp. n. was coded as “*Trigonopterus* sp. 535”. This species is very closely related to *T.pseudovatulus* Riedel, sp. n., from which it cannot be separated by morphological characters. It differs by ca. 17.1–17.3% p-distance of *cox1*, correlated by marked differences in the nuclear genes 28S and CAD. Two allopatric populations occur at Gn. Karre and at the mountains north of Lake Poso. The latter population is sympatric with *T.pseudovatulus* Riedel, sp. n.. In the future, more comprehensive studies could lead to the conclusion that these populations also constitute distinct species.

### 59. Trigonopterus pagaranganensis

Taxon classificationAnimaliaColeopteraCurculionidae

Riedel sp. n.

http://zoobank.org/57FE6FFB-8272-4755-B07A-C1746EE81D65

#### Diagnostic description.

**Figure 59.**
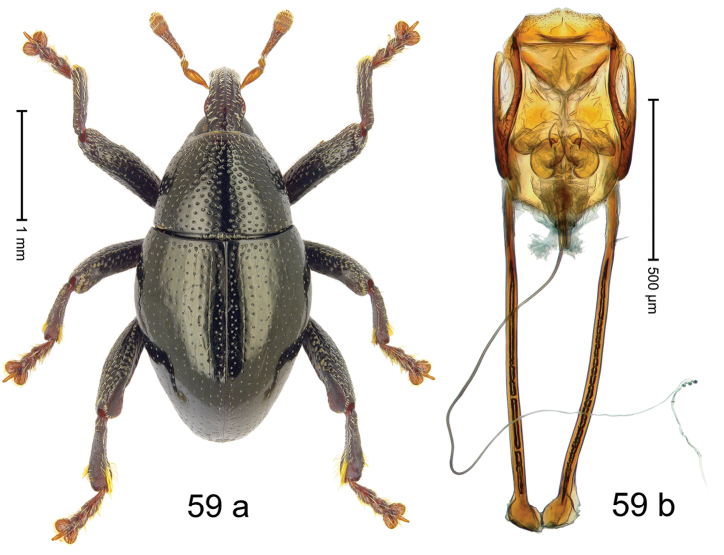
*Trigonopteruspagaranganensis* Riedel, sp. n., holotype; **a** habitus **b** penis.

***Holotype***, male (Fig. 59a). Length 2.88 mm. Color of antennae and tarsi ferruginous; remainder black. Body subovate; in dorsal aspect and in profile with very weak constriction between pronotum and elytron. Rostrum dorsally with median carina and pair of submedian ridges; intervening furrows with rows of subrecumbent yellowish scales; epistome indistinct, somewhat subglabrous, sparsely setose. Pronotum with disk densely punctate; interspaces between punctures subglabrous, subequal to punctures´ diameter; each puncture with fine, recumbent seta; median line impunctate. Elytra irregularly punctate with small punctures; interspaces subglabrous; some striae marked by fine hairlines. Femora dentate with acute tooth; anterior surface coarsely punctate, each puncture with narrow recumbent scale. Metafemur subapically with stridulatory patch. Metatibia clavate, in apical 1/2 widened, in apical 1/3 ventrally with brush of long setae. Metaventrite medially with short median carina. Abdominal ventrites 1–2 concave, sublaterally covered with erect plumose scales; ventrite 5 at middle with shallow concavity, sublaterally with patches of erect scales. Penis (Fig. 59b) with body differentiated into lateral struts and sinuate inner frame, apex subangulate; apodemes 2.1 × as long as body of penis; transfer apparatus complex; ductus ejaculatorius without bulbus. ***Intraspecific variation***. Length 2.74–2.88 mm. Female rostrum dorsally subglabrous, with submedian and sublateral rows of punctures. Female metatibia apically less swollen, without brush of long setae. Female abdominal ventrites 1–2 weakly concave, without erect plumose scales; female abdominal ventrite 5 flat, densely punctate, with scattered minute scales.

#### Material examined.

***Holotype*** (MZB): ARC3192 (GenBank # MK260414), S-Sulawesi Prov., Selayar Is, Pagarangan, 06°18.334'S 120°30.794'E, 545 m, beaten, 24-IV-2013. ***Paratypes*** (MZB, SMNK): S-Sulawesi Prov., Selayar Is: 20 exx, ARC3193 (GenBank # MK260416), ARC3194 (GenBank # MK260413), same data as holotype; 3 exx, ARC3195 (GenBank # MK260415), Bahorea, 06°20.484'S 120°30.127'E, 368 m, beaten 25-IV-2013.

#### Distribution.

S-Sulawesi Prov. (Selayar Is). Elevation 370–545 m.

#### Biology.

On foliage in lowland forests.

#### Etymology.

This epithet is a Latinized adjective based on the type locality.

#### Notes.

*Trigonopteruspagaranganensis* Riedel, sp. n. was coded as “*Trigonopterus* sp. 472”.

### 60. Trigonopterus palopensis

Taxon classificationAnimaliaColeopteraCurculionidae

Riedel sp. n.

http://zoobank.org/2D7AE6A2-1F15-4D44-9751-5D6C93FAF8E4

#### Diagnostic description.

**Figure 60.**
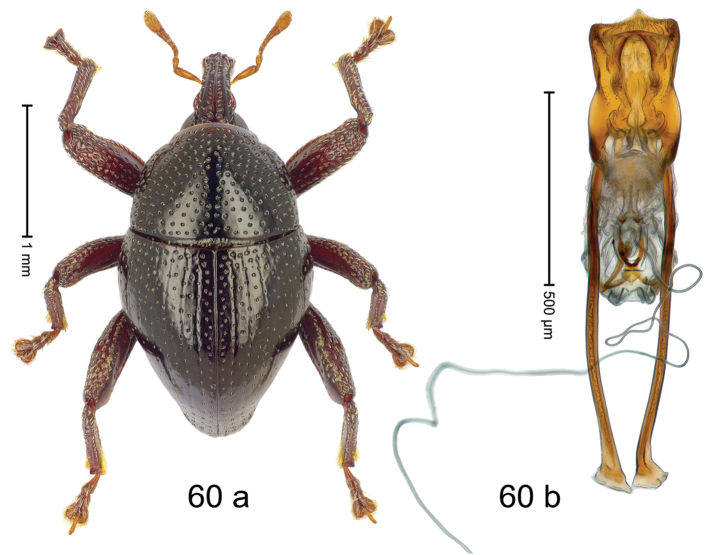
*Trigonopteruspalopensis* Riedel, sp. n., holotype; **a** habitus **b** penis.

***Holotype***, male (Fig. 60a). Length 2.45 mm. Color of antennae ferruginous; legs dark ferruginous; remainder black. Body subovate; in dorsal aspect with moderate constriction between pronotum and elytron; in profile dorsally convex. Rostrum dorsally with median and pair of submedian ridges; intervening furrows each with sparse row of suberect piliform scales; epistome with one median denticle and two pairs of submedian / sublateral denticles. Pronotum with disk densely punctate; each puncture containing a single seta; median line impunctate; interspaces between punctures subglabrous, subequal to punctures´ diameter. Elytra densely punctate with small punctures; striae marked by hairlines; interspaces between punctures subglabrous; striae 7–9 with larger punctures, stria 8 along humerus with seven large, coarse punctures. Femora edentate; anteroventral ridge shortened in apical 1/3. Metafemur with dorsoposterior edge weakly denticulate; subapically with stridulatory patch. Abdominal ventrites 1–2 concave, subglabrous; behind metacoxa with lateral flange; in profile ventrite 2 weakly projecting, rounded; ventrite 5 flat, subglabrous, sparsely punctate. Penis (Fig. 60b) with sides of body constricted at middle; apex pointed; dorsally body near middle with pair of densely setose patches; apodemes 2.7 × as long as body of penis; transfer apparatus spiniform, directed basad in repose, supported by X-shaped sclerite; ductus ejaculatorius with indistinct bulbus. ***Intraspecific variation***. Length 2.45–2.98 mm. Female rostrum slender, dorsally subglabrous, anteriorly with rows of punctures, near base with median and pair of submedian costae; epistome simple.

#### Material examined.

***Holotype*** (MZB): ARC2839 (GenBank # MK260234), S-Sulawesi Prov., Pc. Palopo, Gn. Sampuna, 02°56.545'S 120°05.595'E, 1101 m, beaten, 29-V-2012. ***Paratypes*** (MZB, SMNK, ZSM): S-Sulawesi Prov., Pc. Palopo, Gn. Sampuna: 3 exx, ARC2838 (GenBank # MK260229), ARC6072 (GenBank # MK260233), ARC6073 (GenBank # MK260232), same data as holotype; 11 exx, ARC2814 (GenBank # MK260230), ARC2815 (EMBL # LN884931), ARC2816 (GenBank # MK260231), 02°56.539'S 120°05.320'E to 02°56.545'S 120°05.595'E, 1038–1101 m, beaten, 29-V-2012; 13 exx, 02°56.545'S 120°05.595'E, 1101 m, sifted, 02-V-2013.

#### Distribution.

S-Sulawesi Prov. (Pc. Palopo). Elevation 1040–1100 m.

#### Biology.

On foliage in montane forests, rarely in leaf litter.

#### Etymology.

This epithet is a Latinized adjective based on the type locality Puncak Palopo.

#### Notes.

*Trigonopteruspalopensis* Riedel, sp. n. was coded as “*Trigonopterus* sp. 379”.

### 61. Trigonopterus paracollaris

Taxon classificationAnimaliaColeopteraCurculionidae

Riedel sp. n.

http://zoobank.org/91BDBA22-A340-44C5-9A72-29D631278F41

#### Diagnostic description.

**Figure 61.**
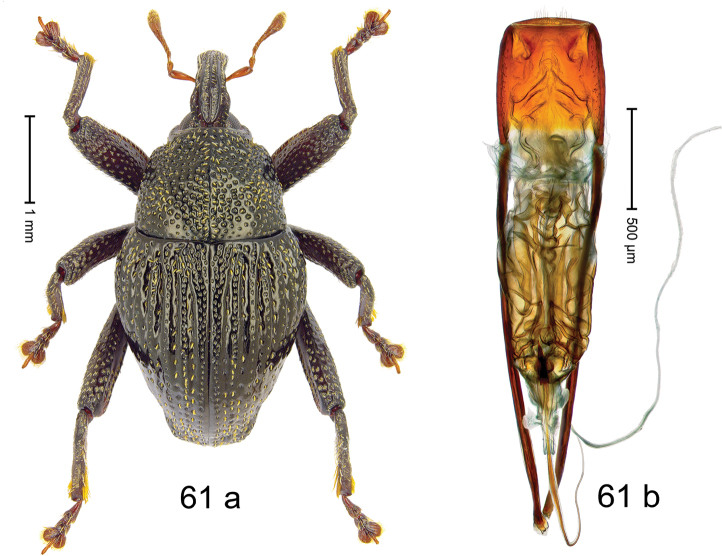
*Trigonopterusparacollaris* Riedel, sp. n., holotype; **a** habitus **b** penis.

***Holotype***, male (Fig. 61a). Length 3.68 mm. Color of antennae and tarsi ferruginous; remainder black with slight bronze luster. Body subovate; in dorsal aspect with distinct constriction between pronotum and elytron; in profile dorsally convex. Rostrum dorsally with median and pair of submedian ridges, converging on forehead; intervening furrows each with sparse row of suberect piliform scales. Pronotum with sides converging in straight line to distinct subapical constriction; disk densely coarsely punctate-reticulate, medially costate; with scattered recumbent narrow scales. Elytra with striae deeply impressed; intervals costate, subglabrous, sutural interval densely punctate, intervals 2–7 each with single row of punctures; with scattered recumbent narrow scales. Profemur with anteroventral ridge simple, ending with small blunt tooth. Meso- and metafemur with anteroventral ridge crenate, ending with small acute tooth; anterior surface densely punctate, each puncture with recumbent narrow scale. Metafemur with dorsoposterior edge weakly crenate; subapically with stridulatory patch. Metatibia subbasally widened, dorsal contour subangulate. Abdominal ventrites 1–2 medially concave, subglabrous; sublaterally with suberect scales; ventrite 5 at middle with shallow pit, coarsely punctate, microreticulate, sparsely setose. Penis (Fig. 61b) with sides of body subparallel, weakly converging to subangulate apex, sparsely setose; endophallus brownish, with numerous folds; apodemes 3.2 × as long as body of penis; transfer apparatus spiniform; ductus ejaculatorius basally sclerotized, without bulbus.

#### Material examined.

***Holotype*** (MZB): ARC3024 (GenBank # MK260583), S-Sulawesi Prov., Tanah Toraja, Bittuang, Gn. Ponding, 02°56.446'S 119°38.075'E, 1625 m, sifted, 09-V-2013.

#### Distribution.

S-Sulawesi Prov. (Tanah Toraja). Elevation ca. 1625 m.

#### Biology.

In leaf litter of montane forest.

#### Etymology.

This epithet is a Latinized adjective based on the Greek prefix *para* (next to; nearby) and the name of *T.collaris* Riedel, sp. n., a sibling species.

#### Notes.

*Trigonopterusparacollaris* Riedel, sp. n. was coded as “*Trigonopterus* sp. 528”. This species is closely related to *T.collaris* Riedel, sp. n., from which it can be distinguished by the elytral sculpture and the morphology of the male genitalia.

### 62. Trigonopterus pauper

Taxon classificationAnimaliaColeopteraCurculionidae

Riedel sp. n.

http://zoobank.org/A25F64D4-961A-49BA-9352-FD13E9E591C3

#### Diagnostic description.

**Figure 62.**
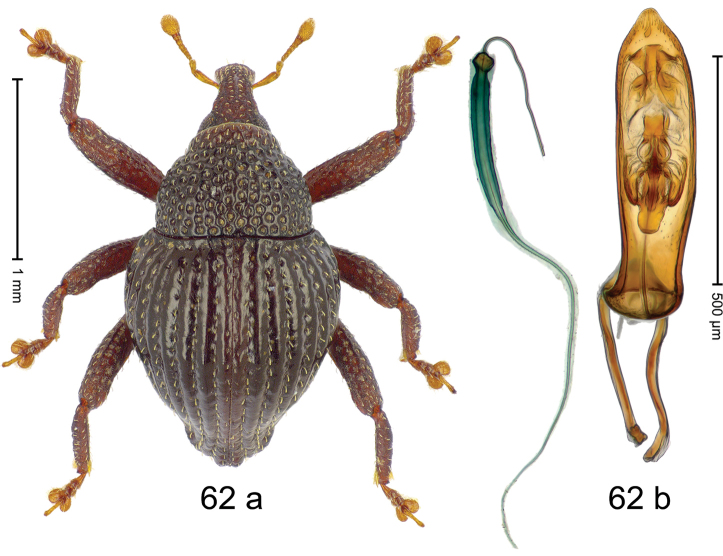
*Trigonopteruspauper* Riedel, sp. n., holotype; **a** habitus **b** penis.

***Holotype***, male (Fig. 62a). Length 1.94 mm. Color of antennae light ferruginous; legs and head dark ferruginous; remainder black. Body subovate; in dorsal aspect and in profile with distinct constriction between pronotum and elytron. Rostrum dorsally coarsely punctate-rugose, with distinct median ridge; with sparse rows of suberect setae; epistome indistinct, subglabrous. Pronotum with weak subapical constriction; disk coarsely punctate, interspaces reticulate; each puncture with erect, slender, curved scale. Elytra with striae deeply impressed; each puncture with erect, slender, curved scale; intervals costate, subglabrous. Meso- and metafemur with small tooth, profemur edentate. Metafemur subapically without stridulatory patch. Abdominal ventrite 5 flat, microreticulate, sparsely setose. Penis (Fig. 62b) hardly asymmetrical; tip medially weakly extended; basal orifice ventrally simple; apodemes shorter than body of penis (0.7 ×); ductus ejaculatorius with distinct bulbus. ***Intraspecific variation***. Length 1.68–2.18 mm. Color as in holotype, or darker, with only antenna and tarsi ferruginous, remainder black. Female rostrum dorsally in apical 1/2 subglabrous, densely punctate; epistome simple.

#### Material examined.

***Holotype*** (MZB): ARC2771 (GenBank # MK260400), N-Sulawesi Prov., Tomohon, Rurukan, Gn. Mahawu, 01°21.409'N 124°51.535'E, 1126 m, 14-V-2012. ***Paratypes*** (MZB, SMNK, ZSM): N-Sulawesi: 2 exx, ARC2777 (GenBank # MK260394), ARC2778 (GenBank # MK260395), Airmadidi, Gn. Klabat, 01°26.505'N 125°00.636'E, 923 m, 16-V-2012, sifted; 12 exx, ARC6056 (GenBank # MK260399), ARC6057 (GenBank # MK260401), Airmadidi, Gn. Klabat, 01°26.430’ N 125°00.622'E, 889 m, sifted, 17-V-2012; 1 ex, Airmadidi, Gn. Klabat, 01°26.619'N 125°00.805'E, 1018 m, sifted, 17-V-2012; 7 exx, ARC2781 (GenBank # MK260396), ARC2782 (GenBank # MK260397), ARC2783 (GenBank # MK260398), Airmadidi, Gn. Klabat, 01°26.635'N 125°00.823'E, 1031 m, 16-V-2012, sifted; 5 exx, ARC0323 (EMBL # LN884929), ARC0324 (GenBank # MK260402), ARC0325 (GenBank # MK260403), ARC0326 (EMBL # HG939575), Bitung, Gn. Dua Saudara, 01°29.417'N 125°09.150'E, 950 m, 24-VI-2006, sifted; 3 exx, ARC0350 (GenBank # MK260404), Bitung, Gn. Dua Saudara, 750 m, 24-VI-2006, sifted.

#### Distribution.

N-Sulawesi Prov. (Tomohon, Gn. Klabat, Bitung). Elevation 750–1130 m.

#### Biology.

In leaf litter of montane forest.

#### Etymology.

This epithet is the Latin noun *pauper* (poor man).

#### Notes.

*Trigonopteruspauper* Riedel, sp. n. was coded as “*Trigonopterus* sp. 427”.

### 63. Trigonopterus pendolensis

Taxon classificationAnimaliaColeopteraCurculionidae

Riedel sp. n.

http://zoobank.org/6DA462D3-6D91-4B01-85A9-3A3022EE5277

#### Diagnostic description.

**Figure 63.**
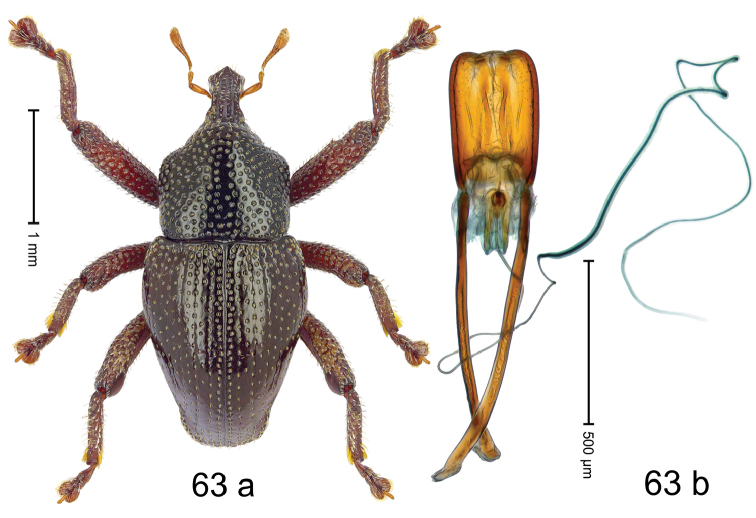
*Trigonopteruspendolensis* Riedel, sp. n., holotype; **a** habitus **b** penis.

***Holotype***, male (Fig. 63a). Length 2.85 mm. Color of antennae and legs ferruginous; remainder black. Body elongate; in dorsal aspect with distinct constriction between pronotum and elytron; in profile dorsally flat. Rostrum dorsally with median ridge and pair of submedian ridges; intervening furrows with rows of erect setae, towards base with elongate scales; epistome short, posteriorly with transverse angulate ridge, with median denticle. Pronotum with disk subquadrate, abruptly rounded to weak subapical constriction; densely punctate; each puncture with fine, recumbent seta; interspaces between punctures subglabrous. Elytra densely irregularly punctate; some striae marked by fine hairlines; apex subtruncate; apex and base with sparse scales. Foreleg relatively long. Femora with small, acute tooth; anteroventral ridges of meso- and metafemur crenate; anterior surface coarsely punctate, reticulate, each puncture with long subclavate scale. Metafemur subapically with stridulatory patch. Dorsal edge of tibiae subbasally with tooth. Abdominal ventrites 1–2 concave, microreticulate, with sparse punctures bearing erect elongate scales; ventrite 5 with shallow median depression, coarsely punctate, with sparse erect scales. Penis (Fig. 63b) with sides of body subparallel; converging to sparsely setose, rounded apex; in profile with apex markedly curved ventrad; apodemes 2.5 × as long as body of penis; transfer apparatus short flagelliform, 0.5 × as long as body of penis; ductus ejaculatorius with indistinct bulbus. ***Intraspecific variation***. Length 2.23–2.85 mm. Female rostrum more slender, epistome indistinct. Female foreleg shorter. Female abdominal ventrite 5 flat.

#### Material examined.

***Holotype*** (MZB): ARC3132 (EMBL # LN884961), C-Sulawesi Prov., Pendolo, Boe, 02°05.405'S 120°38.551'E, 857 m, sifted, 15-V-2013. ***Paratypes*** (ARC, MZB, SMNK): C-Sulawesi Prov.: 21 exx, ARC3133 (GenBank # MK260487), ARC3134 (GenBank # MK260485), ARC6035 (GenBank # MK260484), ARC6036 (GenBank # MK260486), same data as holotype; 1 ex, Pendolo, Boe, 21-VIII-1990, sifted.

#### Distribution.

C-Sulawesi Prov. (Pendolo). Elevation ca. 860 m.

#### Biology.

In leaf litter of montane forest.

#### Etymology.

This epithet is a Latinized adjective based on the type locality.

#### Notes.

*Trigonopteruspendolensis* Riedel, sp. n. was coded as “*Trigonopterus* sp. 495”.

### 64. Trigonopterus posoensis

Taxon classificationAnimaliaColeopteraCurculionidae

Riedel sp. n.

http://zoobank.org/8CAF82B8-656E-4CAC-A34B-094B92DA5818

#### Diagnostic description.

**Figure 64.**
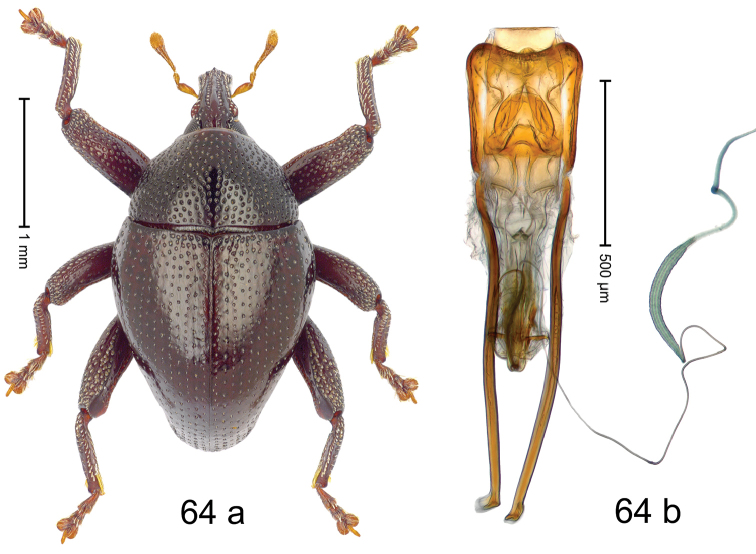
*Trigonopterusposoensis* Riedel, sp. n., holotype; **a** habitus **b** penis.

***Holotype***, male (Fig. 64a). Length 2.60 mm. Color of antennae ferruginous; legs dark ferruginous; remainder black. Body subovate; in dorsal aspect with moderate constriction between pronotum and elytron; in profile dorsally convex. Rostrum dorsally with median and pair of submedian ridges; intervening furrows each with sparse row of erect scales; epistome scabrous, with median ridge. Pronotum with disk densely punctate with coarse, almost confluent punctures; each puncture containing single, minute seta; narrow interspaces subglabrous; medially with indistinct costa. Elytra densely irregularly punctate with small punctures; striae indistinct; interspaces between punctures subglabrous; striae 7–9 with larger punctures; stria 8 along humerus with seven large, coarse punctures. Femora edentate; anterior surface densely coarsely punctate, each puncture with suberect scale. Metafemur with dorsoposterior edge simple; subapically with stridulatory patch. Abdominal ventrites 1–2 concave, subglabrous, with sparse coarse punctures; in profile ventrite 2 rounded; ventrite 5 at middle with shallow impression, densely coarsely punctate, sublaterally with sparse erect scales. Penis (Fig. 64b) with sides of body diverging; apex bent ventrad, subangulate, with short subacute median extension, with sparse setae; apodemes 2.8 × as long as body; transfer apparatus complex, with basal sclerites and flagelliform part, subequal to body of penis; ductus ejaculatorius with distinct bulbus. ***Intraspecific variation***. Length 2.60–2.78 mm. Female rostrum slender, dorsally with median and pair of submedian glabrous costae; epistome simple, punctate-rugose. Female abdominal ventrites 1–2 almost flat.

#### Material examined.

***Holotype*** (MZB): ARC2873 (EMBL # LN884941), C-Sulawesi Prov., Tentena, Taripa, 01°51.589'S 120°48.353'E to 01°50.886'S 120°48.336'E, 680–770 m, beaten, 02-VI-2012. ***Paratypes*** (ARC, MZB, SMNK, ZSM): C-Sulawesi Prov., Lake Poso area: 4 exx, ARC2874 (GenBank # MK260213), ARC2875 (GenBank # MK260214), same data as holotype; 2 exx, Tentena, Taripa, 01°49.858'S 120°47.108'E to 01°50.040'S 120°46.666'E, 896–911 m, beaten, 02-VI-2012; 11 exx, ARC3121 (GenBank # MK260204), ARC3122 (GenBank # MK260215), Pendolo, Gn. Sampuraga, 02°14.099'S 120°46.631'E, 1245 m, beaten, 13-V-2013; 1 ex, Pendolo, Gn. Sampuraga, 02°14.099'S 120°46.631'E, 1245 m, sifted, 13-V-2013; 239 exx, ARC3107 (GenBank # MK260209), ARC3108 (GenBank # MK260212), ARC3109 (GenBank # MK260207), ARC3119 (GenBank # MK260206), ARC3120 (GenBank # MK260205), ARC5984 (GenBank # MK260208), ARC5985 (GenBank # MK260210), ARC5986 (GenBank # MK260211), Pendolo, Boe, 02°05.405'S 120°38.551'E to 02°05.446'S 120°38.519'E, 750–950 m, 15-V-2013, beaten; 11 exx, Pendolo, Boe, 02°05.440'S 120°38.537'E, 901 m, sifted, 15-V-2013; 4 exx, Pendolo, Boe, 02°05.446'S 120°38.519'E, 915 m, sifted, 15-V-2013; 10 exx, Pendolo, Boe, 02°05.405'S 120°38.551'E, 857 m, 15-V-2013, beaten; 37 exx, Pendolo, Boe, 21-VIII-1990; 1 ex, Pendolo, 15 km -> Mangkutana, 22-VIII-1990.

#### Biology.

On foliage in lower montane forests.

#### Distribution.

C-Sulawesi Prov. (Pendolo, Tentena). Elevation 680–1245 m.

#### Etymology.

This epithet is a Latinized adjective that refers to Lake Poso which is close to the type locality.

#### Notes.

*Trigonopterusposoensis* Riedel, sp. n. was coded as “*Trigonopterus* sp. 377”. This species is very closely related to *T.obelix* Riedel, sp. n., from which it differs by ca. 15.0–15.5% p-distance of *cox1*, and by the subangularly projecting profile of abdominal ventrite 2.

### 65. Trigonopterus prismae

Taxon classificationAnimaliaColeopteraCurculionidae

Riedel sp. n.

http://zoobank.org/425F3CA1-82D2-463F-A28B-683FA2A5BE28

#### Diagnostic description.

**Figure 65.**
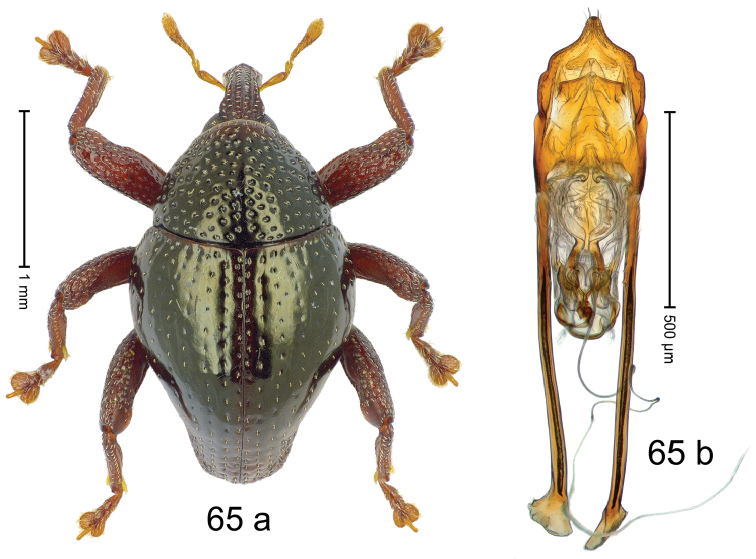
*Trigonopterusprismae* Riedel, sp. n., holotype; **a** habitus **b** penis.

***Holotype***, male (Fig. 65a). Length 2.36 mm. Color of antennae yellowish-ferruginous; legs and head ferruginous; remainder black with bronze luster. Body subovate; in dorsal aspect with weak constriction between pronotum and elytron; in profile dorsally convex. Rostrum dorsally with median and pair of submedian, somewhat irregular ridges; intervening furrows with sparse rows of subrecumbent setae; epistome posteriorly with transverse, angulate ridge. Pronotum with disk densely punctate with coarse punctures except along impunctate median line; interspaces between punctures subglabrous, subequal to or smaller than punctures´ diameter. Elytra with striae marked by small punctures; intervals subglabrous; sutural interval in basal 1/2 with row of small punctures; basal margin bordered by transverse row of punctures; laterally forming ridge along humerus; stria 8 along humerus with four larger punctures. Femora edentate. Meso- and metafemur with anteroventral ridge crenate. Metafemur with dorsoposterior edge weakly denticulate; subapically with stridulatory patch. Abdominal ventrite 1 concave, punctate, with sparse erect piliform scales, microreticulate; ventrite 5 at middle with broad shallow pit, punctate, basally and sublaterally with sparse erect scales. Penis (Fig. 65b) with sides of body subparallel; with narrow lateral flanges; apex with median dentiform extension, sparsely setose; apodemes 2.3 × as long as body of penis; transfer apparatus complex; ductus ejaculatorius with indistinct bulbus. ***Intraspecific variation***. Length 1.76–2.36 mm. Female rostrum in apical 1/2 subglabrous with submedian and sublateral rows of punctures; without distinct epistome. Female abdominal ventrite 1 subglabrous, ventrite 5 flat.

#### Material examined.

***Holotype*** (MZB): ARC3218 (GenBank # MK260539), S-Sulawesi Prov., Mangkutana, 02°20.306'S 120°46.792'E, 909 m, sifted, 14-V-2013. ***Paratypes*** (MZB, SMNK): S-Sulawesi Prov.: 28 exx, ARC3217 (GenBank # MK260538), ARC3219 (GenBank # MK260540), ARC3220 (GenBank # MK260541), same data as holotype; 21 exx, Mangkutana, 02°20.203'S 120°46.878'E, 903 m, sifted, 14-V-2013.

#### Distribution.

S-Sulawesi Prov. (Mangkutana). Elevation ca. 900–910 m.

#### Biology.

In leaf litter of montane forest.

#### Etymology.

This epithet honors the senior author’s wife, Prisma Riedel. An invariable genitive.

#### Notes.

*Trigonopterusprismae* Riedel, sp. n. was coded as “*Trigonopterus* sp. 514”.

### 66. Trigonopterus procurtus

Taxon classificationAnimaliaColeopteraCurculionidae

Riedel sp. n.

http://zoobank.org/6ED58BAB-BF9B-4E92-869C-5446BD7A45E3

#### Diagnostic description.

**Figure 66.**
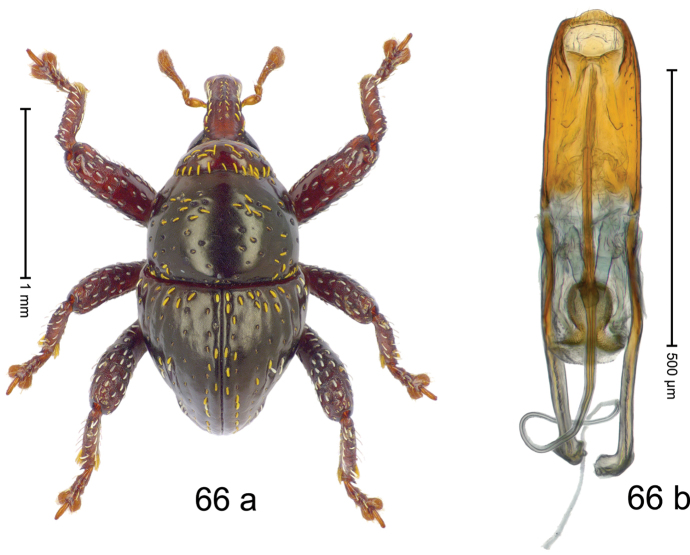
*Trigonopterusprocurtus* Riedel, sp. n., holotype; **a** habitus **b** penis.

***Holotype***, male (Fig. 66a). Length 1.78 mm. Color of antennae ferruginous; legs and rostrum dark ferruginous; remainder black. Body subovate; in dorsal aspect with weak constriction, in profile with distinct constriction between pronotum and elytron. Rostrum long; at level of antennal insertion with shallow lateral constriction, dorsally with median ridge; dorsolaterally with rows of erect yellow scales; epistome simple, subglabrous. Pronotum with distinct subapical constriction bordered by row of yellowish scales; disk with scattered coarse punctures and recumbent yellowish scales; interspaces microreticulate, dull. Elytra sparsely punctate with small punctures, with sparse yellowish or white recumbent scales; interspaces microreticulate, dull; striae indistinct, some obsolete. Femora with indistinct anteroventral ridge ending with indistinct blunt tooth. Metafemur subbasally curved, dorsally rounded, subapically without stridulatory patch. Dorsal edge of tibiae irregularly microdenticulate. Abdominal ventrite 1 concave, subglabrous, sparsely punctate, with few scattered piliform scales; ventrite 5 flat, subapically with weak depression, subglabrous. Penis (Fig. 66b) with sides of body weakly converging to subangulate apex, with sparse thin setae; apodemes 1.8 × as long as body of penis; transfer apparatus flagelliform, 1.4 × longer than body of penis; ductus ejaculatorius without bulbus. ***Intraspecific variation***. Length 1.78–1.79 mm.

#### Material examined.

***Holotype*** (MZB): ARC2892 (GenBank # MK260392), N-Sulawesi Prov., Kotamobagu, Modoinding, Lake Moat area, 00°42.862'N 124°28.356'E, 1024 m, sifted, 19-V-2012. ***Paratype*** (SMNK): 1 ex, ARC2893 (GenBank # MK260391), same data as holotype.

#### Distribution.

N-Sulawesi Prov. (Lake Moat area). Elevation ca. 1020 m.

#### Biology.

In leaf litter of montane forest.

#### Etymology.

This epithet is a combination of the Latin prefix *pro*- (before) and the name of *T.curtus* (Voss). Presumably, this species represents an early offshoot from the *T.curtus*-species-group. A variable adjective.

#### Notes.

*Trigonopterusprocurtus* Riedel, sp. n. was coded as “*Trigonopterus* sp. 420”.

### 67. Trigonopterus pseudallotopus

Taxon classificationAnimaliaColeopteraCurculionidae

Riedel sp. n.

http://zoobank.org/ED1BF737-4FB6-4C5A-9FBE-79FCD78E2E52

#### Diagnostic description.

**Figure 67.**
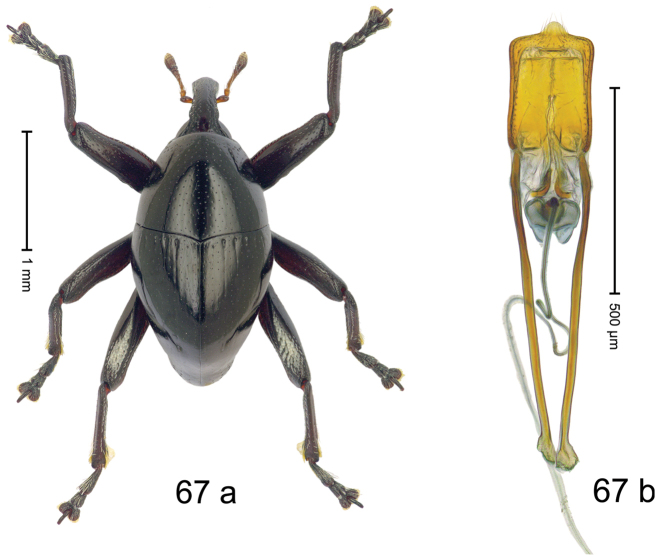
*Trigonopteruspseudallotopus* Riedel, sp. n., holotype; **a** habitus **b** penis.

***Holotype***, male (Fig. 67a). Length 2.40 mm. Color of antennae ferruginous; remainder black. Body ovate, almost without constriction between pronotum and elytron; in profile evenly convex. Rostrum dorsally punctate; with dorsolateral furrows. Eyes with dorsal margin bordered by furrow, continuous with forehead, not carinate. Pronotum subglabrous, sparsely punctate with minute punctures, laterally above procoxa with sparse coarse punctures. Elytra subglabrous, striae hardly visible but marked by few deeper punctures along basal margin and near apex; humeral region subglabrous. Femora subglabrous, with minute punctures; with anteroventral ridge distinct, simple. Posterior surface of metafemur with two longitudinal furrows; with simple dorsoposterior edge; subapically without stridulatory patch. Mesotibia basally rounded; subapically with uncus and larger premucro. Metatibia subapically with fringe of curved, white setae; with uncus, without premucro. Abdominal ventrite 2 swollen, with posterior edge projecting, medially forming common cavity with ventrite 1; ventrite 5 dull, microreticulate, punctate, with shallow subapical depression. Penis (Fig. 67b). Apex symmetrical, with median triangular extension; apodemes 2.7 × as long as body of penis; transfer apparatus dentiform, apically bordered by pair of L-shaped sclerites; ductus ejaculatorius with indistinct bulbus. ***Intraspecific variation***. Length 2.24–2.63 mm. Female mesotibia subapically with uncus and minute premucro. Female metatibia subapically simple, without fringe of long setae. Female abdominal ventrites 1–2 medially flat.

#### Material examined.

***Holotype*** (MZB): ARC2854 (EMBL # LN884937), C-Sulawesi Prov., Pendolo, Gn. Sampuraga, 02°12.476'S 120°45.506'E, 1050 m, 31-V-2012. ***Paratypes*** (MZB, SMNK): S-Sulawesi Prov.: 1 ex, ARC2820 (GenBank # MK260637), Pc. Palopo, Gn. Sampuna, 02°56.539'S 120°05.320'E to 02°56.545'S 120°05.595'E, 1038–1101 m, beaten, 29-V-2012; C-Sulawesi Prov.: 1 ex, ARC2853 (GenBank # MK260633), same data as holotype; 2 exx, ARC3117 (GenBank # MK260631), ARC3118 (GenBank # MK260635), Pendolo, Gn. Sampuraga, 02°12.165'S 120°45.567'E to 02°12.308'S 120°45.544'E, beaten, 934–1011 m, 13-V-2013; 1 ex, ARC3099 (GenBank # MK260634), Pendolo, Boe, 02°05.405'S 120°38.551'E to 02°05.446'S 120°38.519'E, beaten, 750–950 m, 15-V-2013; 2 exx, ARC3265 (GenBank # MK260636), ARC3266 (GenBank # MK260632), Luwuk, Salodi, Gn. Taluanjang, 00°49.202'S 122°52.395'E to 00°49.307'S 122°52.322'E, 600–760 m, beaten, 21-V-2013.

#### Distribution.

S-Sulawesi Prov. (Pc. Palopo); C-Sulawesi Prov. (Pendolo, Luwuk). Elevation 760–1050 m.

#### Biology.

On foliage in montane forests.

#### Etymology.

This epithet is based on the Greek prefix *pseudo*- (false) and the name of *T.allotopus* Riedel, a sibling species. A noun in apposition.

#### Notes.

*Trigonopteruspseudallotopus* Riedel, sp. n. was coded as “*Trigonopterus* sp. 945”. This species is closely related to *T.allotopus* Riedel, from which it differs by ca. 5.8–6.4% p-distance of *cox1* and morphologically by the weakly impressed striae 7–9 of the elytral humeri.

### 68. Trigonopterus pseudanalis

Taxon classificationAnimaliaColeopteraCurculionidae

Riedel sp. n.

http://zoobank.org/7044E7A3-94B5-4479-8766-939F9F4CD65E

#### Diagnostic description.

**Figure 68.**
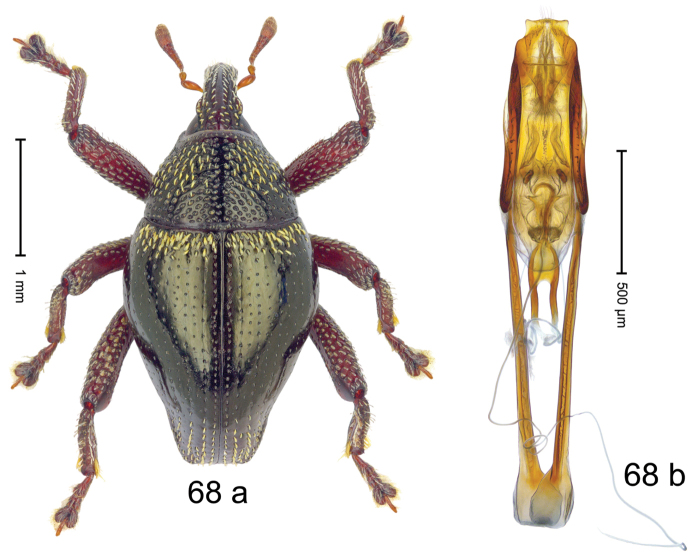
*Trigonopteruspseudanalis* Riedel, sp. n., holotype; **a** habitus **b** penis.

***Holotype***, male (Fig. 68a). Length 2.95 mm. Color of antennae and legs ferruginous; remainder black with slight bronze luster. Body subovate; in dorsal aspect with weak constriction between pronotum and elytron; in profile dorsally convex. Rostrum dorsally with median and pair of submedian ridges; intervening furrows with rows of erect yellowish scales; epistome indistinct, subglabrous, sparsely setose. Pronotum with disk densely, coarsely punctate; punctures subovate, with oblique anteriomesad orientation; interspaces between punctures subglabrous, reticulate; in anterior 1/2 punctures with clavate, yellowish scale; median line impunctate. Elytra irregularly punctate with small punctures; interspaces subglabrous; basal margin bordered by deeper punctures and almond-shaped yellowish scales; near apex striae more distinctly impressed, with sparse rows of yellowish scales; apex subtruncate. Femora with anteroventral ridge ending with small tooth; anterior surface coarsely punctate, each puncture with narrow yellowish scale. Metafemur with dorsoposterior ridge denticulate, subapically with stridulatory patch. Meso- and metatibia with dorsal edge irregularly serrate. Metatibia ventrally with fine ridge; subapically near uncus with fine row of long setae. Abdominal ventrites 1–2 weakly concave, with scattered erect white scales, especially behind metacoxa; ventrite 5 concave, subglabrous, microreticulate, at basal margin with median denticle and pair of sublateral denticles, apex curved ventrad forming truncate lamina. Penis (Fig. 68b) with body at middle with narrow lateral membranous lobes; weakly converging; apex subtruncate, sparsely setose; apodemes 1.7 × as long as body of penis; transfer apparatus hook-shaped, ca. 0.4 × length of body, basally continued as fork-shaped sclerite; ductus ejaculatorius without bulbus. ***Intraspecific variation***. Length 2.18–2.95 mm. Female rostrum slender, dorsally subglabrous, with submedian and sublateral rows of punctures. Female abdominal ventrite 5 weakly concave, punctate, with sparse scales.

#### Material examined.

***Holotype*** (MZB): ARC3213 (GenBank # MK260628), S-Sulawesi, Mangkutana, 02°20.306'S 120°46.792'E, 909 m, sifted, 14-V-2013. ***Paratypes*** (MZB, SMNK): S-Sulawesi: 6 exx, ARC3214 (GenBank # MK260629), ARC3215 (GenBank # MK260630), ARC3216 (PCR failed), same data as holotype; 5 exx, Mangkutana, 02°20.203'S 120°46.878'E, 903 m, sifted, 14-V-2013; C-Sulawesi: 1 ex, ARC3140 (PCR failed), Pendolo, Boe, 02°05.405'S 120°38.551'E, 857 m, sifted, 15-V-2013.

#### Distribution.

S-Sulawesi Prov. (Mangkutana), C-Sulawesi Prov. (Pendolo). Elevation 860–910 m.

#### Biology.

In leaf litter of lowland forest.

#### Etymology.

This epithet is a Latinized adjective based on the Greek prefix *pseudo*- (false) and the name of *T.analis* Riedel, sp. n., a sibling species.

#### Notes.

*Trigonopteruspseudanalis* Riedel, sp. n. was coded as “*Trigonopterus* sp. 944”. This species is very closely related to *T.analis* Riedel, sp. n., from which it differs by ca. 11.7–12.7% p-distance of *cox1*, by the apex of the penis, and by a stouter body shape with a more densely punctate pronotum.

### 69. Trigonopterus pseudovatulus

Taxon classificationAnimaliaColeopteraCurculionidae

Riedel sp. n.

http://zoobank.org/00D09582-E837-45E5-A894-351F321F885A

#### Diagnostic description.

**Figure 69.**
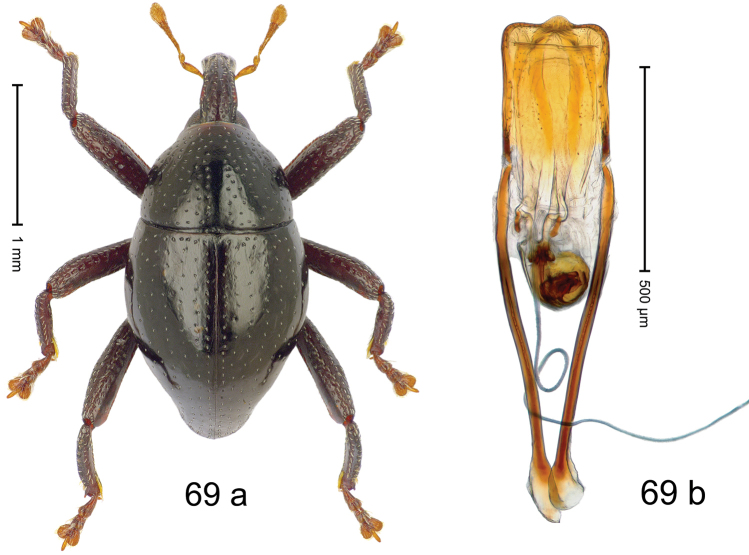
*Trigonopteruspseudovatulus* Riedel, sp. n., holotype; **a** habitus **b** penis.

***Holotype***, male (Fig. 69a). Length 2.35 mm. Color of antennae and tarsi ferruginous; remainder black. Body subovate; in dorsal aspect with weak constriction between pronotum and elytron; in profile dorsally convex. Rostrum dorsally with median costa and pair of submedian ridges, weakly microreticulate; intervening furrows with sparse rows of narrow, recumbent scales; epistome in apical 1/3 indistinct, weakly swollen, subglabrous, with sparse setae. Pronotum with disk punctate with small punctures; interspaces subglabrous, weakly microreticulate. Elytra with striae marked by rows of small punctures; intervals subglabrous; near base with few additional, slightly larger punctures. Femora edentate; anteroventral ridge distinct, ending in apical 1/3; anterior surface densely punctate, microreticulate, each puncture with narrow recumbent scale. Metafemur subapically with extensive stridulatory patch. Metatibia with dorsal edge weakly denticulate, with supra-uncal tooth. Abdominal ventrites 1–2 weakly concave, subglabrous; ventrite 5 with shallow impression, densely punctate, covered with small suberect scales. Penis (Fig. 69b) with body subparallel, apex with subangulate extension, without setae; apodemes 2.7 × as long as body of penis; transfer apparatus complex, asymmetrical, with subrotund capsule; ductus ejaculatorius without bulbus. ***Intraspecific variation***. Length 1.76–2.60 mm. Body of females more slender. Female rostrum dorsally with submedian row of punctures, with sublateral furrow.

#### Material examined.

***Holotype*** (MZB): ARC3128 (GenBank # MK260601), C-Sulawesi Prov., Pendolo, Gn. Sampuraga, 02°15.407'S 120°47.051'E, 1406 m, beaten, 16-V-2013. ***Paratypes*** (MZB, SMNK): 3 exx, ARC3129 (GenBank # MK260600), ARC3130 (GenBank # MK260599), ARC6009 (GenBank # MK260593), ARC6033 (GenBank # MK260606), same data as holotype; 1 ex, ARC2864 (GenBank # MK260594), C-Sulawesi Prov., Pendolo, Gn. Sampuraga, 02°15.407'S 120°47.051'E, 1406 m, beaten, 31-V-2012; 344 exx, ARC3095 (GenBank # MK260605), ARC3096 (GenBank # MK260604), ARC3097 (GenBank # MK260603), ARC3098 (GenBank # MK260602), ARC5978 (PCR failed), ARC5979 (GenBank # MK260598), ARC5980 (PCR failed), ARC5981 (GenBank # MK260597), ARC5982 (GenBank # MK260596), ARC5983 (GenBank # MK260595), S-Sulawesi Prov., Mangkutana, 02°20.306'S 120°46.792'E to 02°20.203'S 120°46.878'E, 850–910 m, beaten, 14-V-2013.

#### Distribution.

S-Sulawesi Prov. (Mangkutana), C-Sulawesi Prov. (Pendolo). Elevation 910–1400 m.

#### Biology.

On foliage in montane forests.

#### Etymology.

This epithet is a Latinized adjective based on the Greek prefix *pseudo*- (false) and the name of *T.ovatulus* Riedel, sp. n., a sibling species.

#### Notes.

*Trigonopteruspseudovatulus* Riedel, sp. n. was coded as “*Trigonopterus* sp. 534”. This species is very closely related to *T.ovatulus* Riedel, sp. n., from which it cannot be separated by morphological characters. It differs by ca. 17.1–17.3% p-distance of *cox1*, correlated by marked differences in the nuclear genes 28S and CAD.

### 70. Trigonopterus pseudovalipunctatus

Taxon classificationAnimaliaColeopteraCurculionidae

Riedel sp. n.

http://zoobank.org/0DEC38E9-5AA4-435B-8839-E9227680D1A9

#### Diagnostic description.

**Figure 70.**
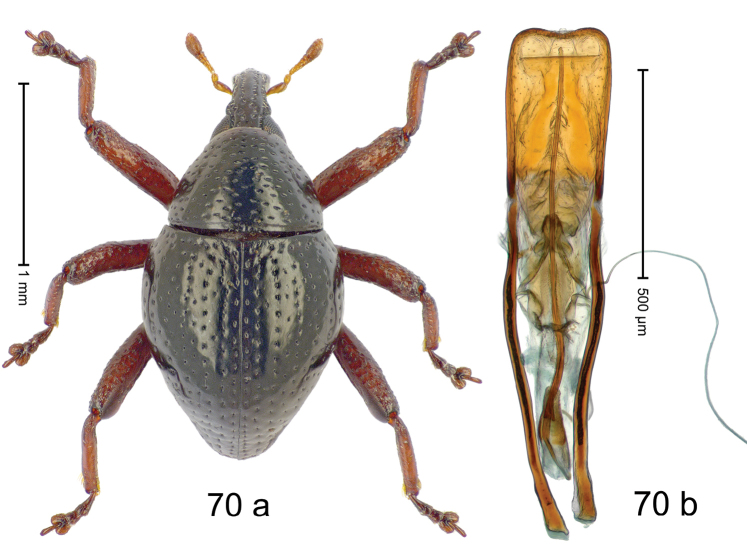
*Trigonopteruspseudovalipunctatus* Riedel, sp. n., holotype; **a** habitus **b** penis.

***Holotype***, male (Fig. 70a). Length 1.78 mm. Color of antennae and legs ferruginous; remainder black. Body subovate; in dorsal aspect with weak constriction between pronotum and elytron, in profile dorsally convex. Rostrum dorsally with median costa and pair of submedian ridges; intervening furrows with sparse rows of short setae; epistome indistinct. Pronotum with ovate punctures of transverse orientation; interspaces subglabrous. Elytra with striae marked by rows of distinct punctures; sutural interval with additional row, other intervals subglabrous, with few interspersed punctures. Femora with anteroventral ridge crenate, ending with blunt angulation; anterior surface weakly rugose, weakly denticulate, microreticulate. Metafemur subapically with stridulatory patch. Abdominal ventrites 1–2 markedly microreticulate, almost impunctate, concave; ventrite 5 coarsely punctate, with depression at middle. Penis (Fig. 70b) with body subparallel, weakly rounded to subtruncate apex; apodemes 2.0 × as long as body of penis; transfer apparatus flagelliform, 2.4 × longer than body of penis; ductus ejaculatorius without bulbus. ***Intraspecific variation***. Length 1.78–1.90 mm. Female rostrum subglabrous, with two submedian rows of punctures, with pair of sublateral furrows.

#### Material examined.

***Holotype*** (MZB): ARC3044 (GenBank # MK260417), S-Sulawesi Prov., Tanah Toraja, Bittuang, Gn. Ponding, 02°56.446'S 119°38.075'E, 1625 m, beaten, 09-V-2013. ***Paratypes*** (MZB, SMNK): S-Sulawesi Prov.: 3 exx, ARC3043 (EMBL # LN884952), ARC3045 (GenBank # MK260418), ARC6029 (GenBank # MK260419), same data as holotype.

#### Distribution.

S-Sulawesi Prov. (Bittuang). Elevation ca. 1625 m.

#### Biology.

On foliage in montane forests.

#### Etymology.

This epithet is a Latinized adjective based on the Greek prefix *pseudo*- (false) and the name of *T.ovalipunctatus* Riedel, sp. n., a sibling species.

#### Notes.

*Trigonopteruspseudovalipunctatus* Riedel, sp. n. was coded as “*Trigonopterus* sp. 474”.

### 71. Trigonopterus pseudofulvicornis

Taxon classificationAnimaliaColeopteraCurculionidae

Riedel sp. n.

http://zoobank.org/CEE8FE95-36F0-40B3-824A-C3BCFD29EDCD

#### Diagnostic description.

**Figure 71.**
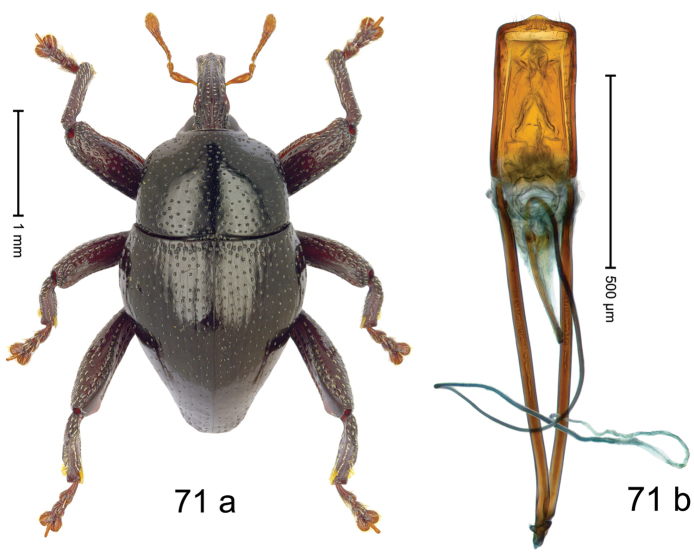
*Trigonopteruspseudofulvicornis* Riedel, sp. n., holotype; **a** habitus **b** penis.

***Holotype***, male (Fig. 71a). Length 2.92 mm. Color of antennae ferruginous; legs dark ferruginous; remainder black. Body subovate; in dorsal aspect with weak constriction between pronotum and elytron; in profile dorsally convex. Rostrum dorsally with median carina and pair of submedian ridges; intervening furrows with sparse rows of suberect narrow scales; epistome indistinct, punctate-rugose. Pronotum with weak lateral flanges, with weak subapical constriction; disk subquadrate, densely punctate with small punctures; interspaces between punctures subglabrous. Elytra irregularly punctate with small punctures; striae marked by hardly visible hairlines; basal margin bordered by slightly larger punctures; stria 8 along humerus with five large, coarse punctures. Femora edentate. Metafemur with anteroventral ridge crenate. Metafemur with dorsoposterior edge weakly crenate; subapically with stridulatory patch. Metatibia with dorsal edge in basal 1/3 weakly denticulate. Abdominal ventrites 1–2 concave, subglabrous; ventrite 5 flat, punctate, with erect scales. Penis (Fig. 71b) with sides of body subparallel; apex subangulate; apodemes 2.4 × as long as body of penis; transfer apparatus flagelliform, 1.3 × as long as body of penis, 0.4 × total length of penis; ductus ejaculatorius with indistinct bulbus. ***Intraspecific variation***. Length 2.56–2.95 mm. Female rostrum slender, dorsally subglabrous, with two submedian rows of minute punctures, with pair of sublateral furrows. Female pronotum more slender. Female abdominal ventrite 5 with sparse recumbent scales.

#### Material examined.

***Holotype*** (MZB): ARC3155 (GenBank # MK260522), SE-Sulawesi Prov., Kendari, road from Wawotobi to Lasolo, 03°45.086'S 122°12.976'E, 339 m, beaten, 17-IV-2013. ***Paratypes*** (MZB, SMNK): 3 exx, same data as holotype; 5 exx, ARC3150 (EMBL # LN884966), ARC3151 (GenBank # MK260524), ARC3152 (GenBank # MK260523), SE-Sulawesi Prov., Kendari, road from Wawotobi to Lasolo, 03°44.142'S 122°13.670'E, 482 m, beaten, 17-IV-2013.

#### Distribution.

SE-Sulawesi Prov. (Kendari). Elevation 340–480 m.

#### Biology.

On foliage in lowland forests.

#### Etymology.

This epithet is a Latinized adjective based on the Greek prefix *pseudo*- (false) and the name of *Trigonopterusfulvicornis* (Pascoe) which is superficially very similar.

#### Notes.

*Trigonopteruspseudofulvicornis* Riedel, sp. n. was coded as “*Trigonopterus* sp. 504”. *Trigonopterusfulvicornis* (Pascoe) differs mainly by the greater length of the transfer apparatus, which is about the penis’ total length in *Trigonopterusfulvicornis* (Pascoe) but only half the penis’ total length in *Trigonopteruspseudofulvicornis* Riedel, sp. n.

### 72. Trigonopterus pseudomanadensis

Taxon classificationAnimaliaColeopteraCurculionidae

Riedel sp. n.

http://zoobank.org/BCAABE0C-ED1C-40BD-9F03-3A4C09B21E4F

#### Diagnostic description.

**Figure 72.**
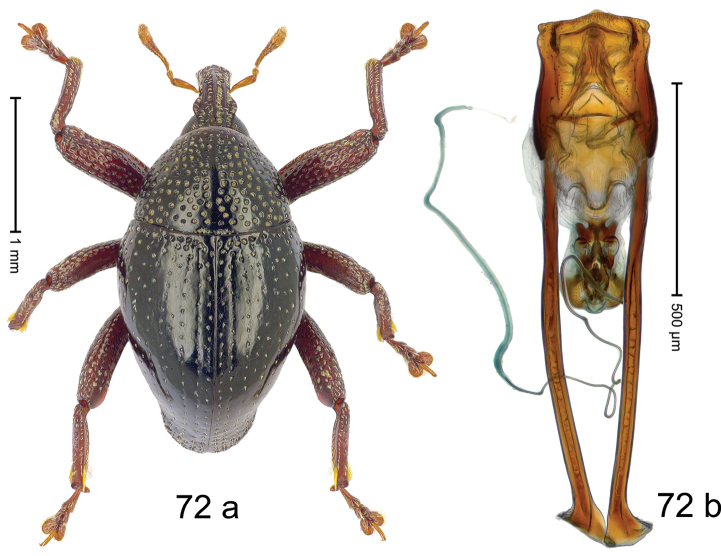
*Trigonopteruspseudomanadensis* Riedel, sp. n., holotype; **a** habitus **b** penis.

***Holotype***, male (Fig. 72a). Length 2.51 mm. Color of antennae and legs ferruginous; remainder black. Body subovate; in profile dorsally convex; with weak constriction between pronotum and elytron. Rostrum dorsally with median and pair of submedian ridges; intervening furrows with sparse rows of suberect setae; epistome swollen, sparsely punctate. Pronotum with disk densely punctate; interspaces between punctures subglabrous, subequal to or smaller than punctures´ diameter; median line weakly costate, impunctate. Elytra with striae marked by rows of small punctures; basal margin weakly costate, bordered by transverse row of deeper punctures; stria 8 and 9 along humerus with large, coarse punctures; sutural interval with additional row of small punctures, other intervals subglabrous, with few interspersed punctures. Femora edentate; anterior surface coarsely punctate, each puncture with recumbent yellowish scale. Metafemur with dorsoposterior edge denticulate; subapically with stridulatory patch. Metatibia with uncus thick, curved ventroposteriad. Abdominal ventrite 1 and anterior portion of ventrite 2 cavernous, subglabrous, microreticulate; ventrite 2 posteriorly forming rim; ventrite 5 in basal 1/2 with weak median ridge and pair of shallow submedian impressions, subapically densely covered with short erect scales. Penis (Fig. 72b) with sides of body subparallel, in apical 1/4 angularly projecting, ventrally converging to acute apex; without setae; apodemes 2.6 × as long as body of penis; transfer apparatus complex; ductus ejaculatorius with indistinct bulbus.

#### Material examined.

***Holotype*** (MZB): ARC2886 (GenBank # MK260300), N-Sulawesi Prov., Kotamobagu, Matalibaru, 00°32.445'N 124°14.185'E, 956 m, beaten, 20-V-2012.

#### Distribution.

N-Sulawesi Prov. (Kotamobagu). Elevation ca. 960 m.

#### Biology.

On foliage in montane forests.

#### Etymology.

This epithet is a combination of the Greek prefix *pseudo*- (false) and the name of *T.manadensis* sp. n., a closely related species. A variable adjective.

#### Notes.

*Trigonopteruspseudomanadensis* Riedel, sp. n. was coded as “*Trigonopterus* sp. 399”.

### 73. Trigonopterus pseudosimulans

Taxon classificationAnimaliaColeopteraCurculionidae

Riedel sp. n.

http://zoobank.org/71216E34-1FD2-44B2-B50F-8CC0532AED3D

#### Diagnostic description.

**Figure 73.**
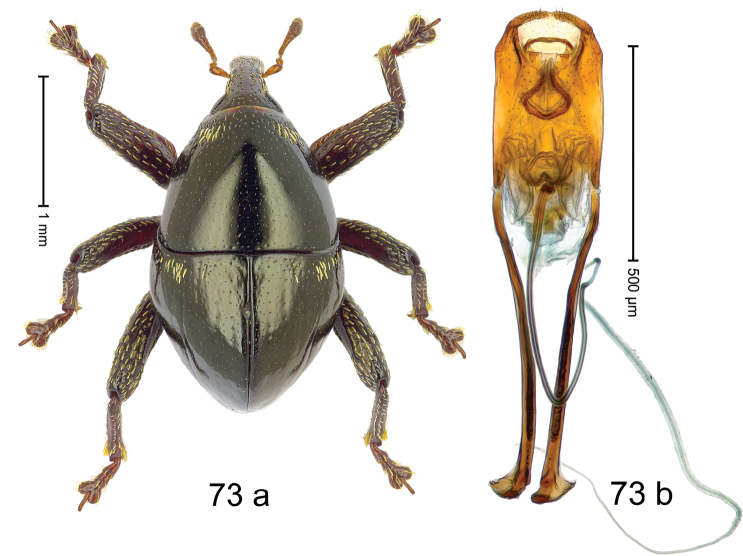
*Trigonopteruspseudosimulans* Riedel, sp. n., holotype; **a** habitus **b** penis.

***Holotype***, male (Fig. 73a). Length 2.53 mm. Color of antennae ferruginous; legs dark ferruginous to black; remainder black. Body subovate; in profile broadly convex, dorsally flat. Rostrum dorsally punctate-rugose, with median subglabrous ridge; with sparse suberect yellow scales; epistome indistinct. Pronotum dorsally subglabrous with sparse minute punctures, anteriorly with small punctures and two patches of sparse recumbent yellowish scales; laterally above procoxa with coarse punctures. Elytra subglabrous with minute punctures, with two patches of sparse recumbent yellowish scales sublaterally near base; striae largely obsolete. Femora edentate; anteroventral ridge distinct, simple. Metafemur with dorsoposterior ridge simple, subapically without stridulatory patch, anterior surface coarsely rugose-punctate, each puncture with narrow recumbent scale. Abdominal ventrites 1–2 subglabrous, microreticulate; ventrite 1 behind metacoxa with angular protrusion, medially impressed; ventrite 2 flat; ventrite 5 swollen, subglabrous, punctate, at middle with shallow impression. Penis (Fig. 73b) with sides of body subparallel, apex rounded, with very few short setae; endophallus near ostium with 8-shaped sclerite; apodemes 2.3 × as long as body of penis; transfer apparatus simple, spiniform; ductus ejaculatorius with indistinct bulbus.

#### Material examined.

***Holotype*** (MZB): ARC2855 (EMBL # LN884938), C-Sulawesi Prov., Pendolo, Gn. Sampuraga, 02°12.476'S 120°45.506'E, 1050 m, beaten, 31-V-2012.

#### Distribution.

C-Sulawesi Prov. (Pendolo). Elevation ca. 1050 m.

#### Biology.

The species most likely inhabits the leaf litter of montane forest; the finding of the holotype on vegetation was probably exceptional.

#### Etymology.

This epithet is a combination of the Greek prefix *pseudo*- (false) and the name of *T.simulans* Riedel, a related species from New Guinea. A variable adjective.

#### Notes.

*Trigonopteruspseudosimulans* Riedel, sp. n. was coded as “*Trigonopterus* sp. 421”.

### 74. Trigonopterus pumilus

Taxon classificationAnimaliaColeopteraCurculionidae

Riedel sp. n.

http://zoobank.org/07C03D68-22CE-43FD-BDCA-23DFCE162A05

#### Diagnostic description.

**Figure 74.**
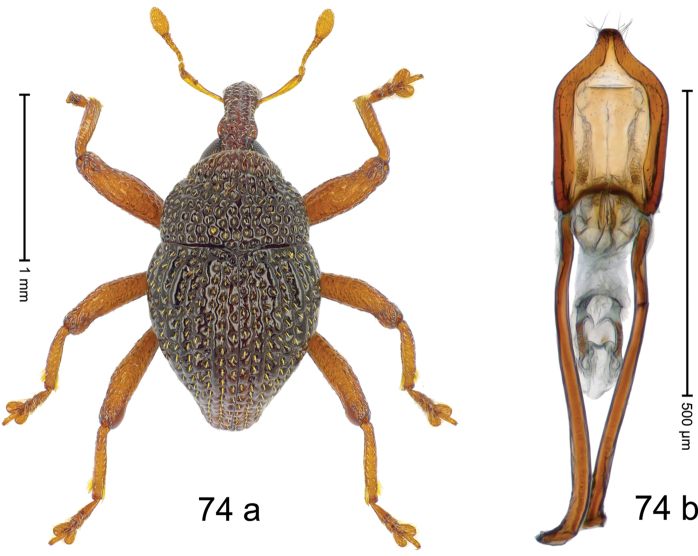
*Trigonopteruspumilus* Riedel, sp. n., holotype; **a** habitus **b** penis.

***Holotype***, male (Fig. 74a). Length 1.67 mm. Color of antennae and legs ferruginous; remainder dark ferruginous to black. Body subovate; in dorsal aspect and in profile with weak constriction between pronotum and elytron. Rostrum dorsally with median ridge and pair of sublateral somewhat irregular ridges; intervening furrows with coarse punctures containing each one indistinct seta; epistome short, subglabrous. Pronotum with disk densely, coarsely punctate; interspaces between punctures reticulate-rugose. Elytra with striae deeply impressed, intervals dorsally tuberculate-costate, subglabrous; punctures each with short, suberect piliform scale. Legs long. Femora edentate. Anteroventral ridges indistinct; anterior surface of femora coriaceous, punctate. Metafemur subapically with stridulatory patch. Abdominal ventrite 1 weakly concave, microreticulate, with coarse punctures; ventrite 5 basally and laterally with coarse punctures, at middle with subglabrous depression. Penis (Fig. 74b) with sides subparallel in basal 1/2; apex forming long, spiniform, markedly downwardly curved extension, laterally with sparse setae; basal orifice with well-sclerotized ventral plate; apodemes 2.3 × as long as body of penis; transfer apparatus and ductus ejaculatorius lost. ***Intraspecific variation***. Length 1.67–1.80 mm. Female rostrum dorsally with rows of coarse punctures, medially with indistinct subglabrous costa; without epistome. Female abdominal ventrite 5 flat, coarsely punctate.

#### Material examined.

***Holotype*** (MZB): ARC3063 (GenBank # MK260547), S-Sulawesi Prov., Tanah Toraja, Bittuang, Gn. Karoa, 02°55.270'S 119°40.179'E, 1836 m, sifted, 10-V-2013. ***Paratypes*** (MZB, SMNK): 3 exx, ARC3064 (GenBank # MK260548), same data as holotype.

#### Distribution.

S-Sulawesi Prov. (Tanah Toraja). Elevation ca. 1840 m.

#### Biology.

In leaf litter of montane forest.

#### Etymology.

This epithet is based on the Latin noun *pumilus* (dwarf) and refers to the species´ small body size.

#### Notes.

*Trigonopteruspumilus* Riedel, sp. n. was coded as “*Trigonopterus* sp. 518”.

### 75. Trigonopterus rantepao

Taxon classificationAnimaliaColeopteraCurculionidae

Riedel sp. n.

http://zoobank.org/028CFE48-40B1-476A-BF3C-FB263FB9A56C

#### Diagnostic description.

**Figure 75.**
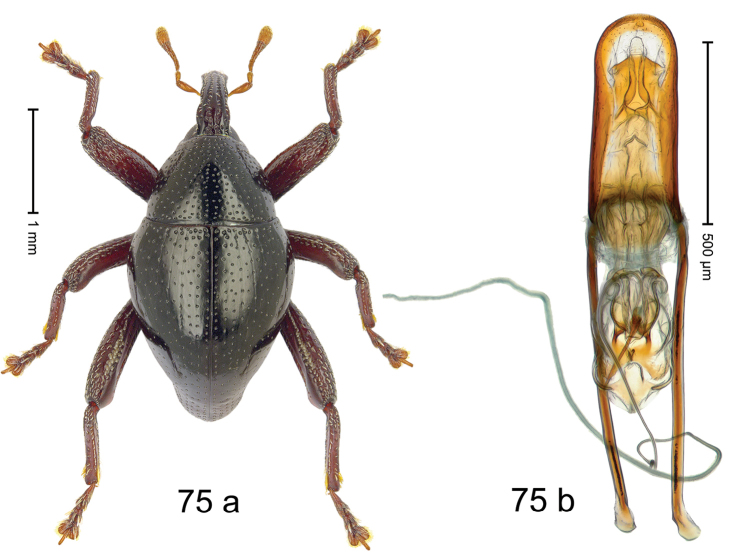
*Trigonopterusrantepao* Riedel, sp. n., holotype; **a** habitus **b** penis.

***Holotype***, male (Fig. 75a). Length 2.73 mm. Color of antennae and legs ferruginous; remainder black. Body subovate; in profile dorsally convex. Rostrum dorsally with median and pair of submedian costae; intervening furrows with sparse rows of suberect piliform scales; epistome indistinct, subglabrous, with sparse setae. Pronotum with disk densely punctate with small punctures; interspaces between punctures subglabrous. Elytra irregularly punctate with small punctures, along basal margin with transverse row of denser punctures; some striae marked by hardly visible hairlines; intervals flat; stria 8 along humerus with seven distinct punctures. Femora with denticle. Meso- and metafemur with anteroventral ridge crenate, ending with denticle in apical 1/3. Metafemur dorsally with row of suberect, silvery scales; dorsoposterior edge crenate in apical 1/3; subapically with stridulatory patch. Metatibia ventrally with row of sparse thin setae; dorsal outline subapically weakly concave. Abdominal ventrites 1–2 concave, subglabrous; in profile ventrite 2 subangularly projecting; ventrite 5 at middle with shallow pit, laterally punctate. Penis (Fig. 75b) with sides of body subparallel; apex rounded, nude; apodemes 1.5 × as long as body of penis; transfer apparatus spiniform, directed basad in repose, surrounded by complex of supporting sclerites; ductus ejaculatorius with indistinct bulbus. ***Intraspecific variation***. Length 2.60–2.78 mm. Female rostrum slender, subglabrous, with two submedian rows of punctures, with pair of sublateral furrows. Female abdominal ventrite 5 flat, coarsely punctate.

#### Material examined.

***Holotype*** (MZB): ARC2987 (GenBank # MK260341), S-Sulawesi Prov., Tanah Toraja, Rantepao, Gn. Karre (= Gn. Wokim), 02°59.021'S 120°02.523'E, 1456 m, beaten, 06-V-2013. ***Paratypes*** (MZB, SMNK, ZSM): S-Sulawesi Prov.: 5 exx, ARC2988 (GenBank # MK260340), ARC2989 (GenBank # MK260338), same data as holotype; 4 exx, ARC5993 (GenBank # MK260344), ARC5994 (GenBank # MK260342), Tanah Toraja, Rantepao, Gn. Karre (= Gn. Wokim), 02°59.013'S 120°02.251'E, 1423 m, beaten, 06-V-2013; 2 exx, Tanah Toraja, Rantepao, Gn. Karre (= Gn. Wokim), 02°58.846'S 120°02.158'E, 1396 m, beaten, 06-V-2013; 14 exx, ARC2821 (EMBL # LN884933), ARC2822 (GenBank # MK260346), ARC6049 (GenBank # MK260345), Pc. Palopo, Gn. Sampuna, 02°56.539'S 120°05.320'E to 02°56.545'S 120°05.595'E, 1038–1101 m, beaten, 29-V-2012; 5 exx, ARC5999 (GenBank # MK260343), ARC6000 (GenBank # MK260339), ARC6001 (GenBank # MK260347), Pc. Palopo, Gn. Sampuna, 02°56.539'S 120°05.320'E to 02°56.545'S 120°05.595'E, 1038–1101 m, beaten, 02-V-2013.

#### Distribution.

S-Sulawesi Prov. (Tanah Toraja, Pc. Palopo). Elevation 1100–1460 m.

#### Biology.

On foliage in montane forests.

#### Etymology.

This epithet is based on the town of Rantepao, center of the Land of Toraja. It is treated as a noun in apposition.

#### Notes.

*Trigonopterusrantepao* Riedel, sp. n. was coded as “*Trigonopterus* sp. 409”. It is closely related to *T.toraja* Riedel, sp. n. from which it can be distinguished by the structure of the penis.

### 76. Trigonopterus reticulatus

Taxon classificationAnimaliaColeopteraCurculionidae

Riedel sp. n.

http://zoobank.org/3820A26F-BE31-45AE-9472-91F1D8AB59C8

#### Diagnostic description.

**Figure 76.**
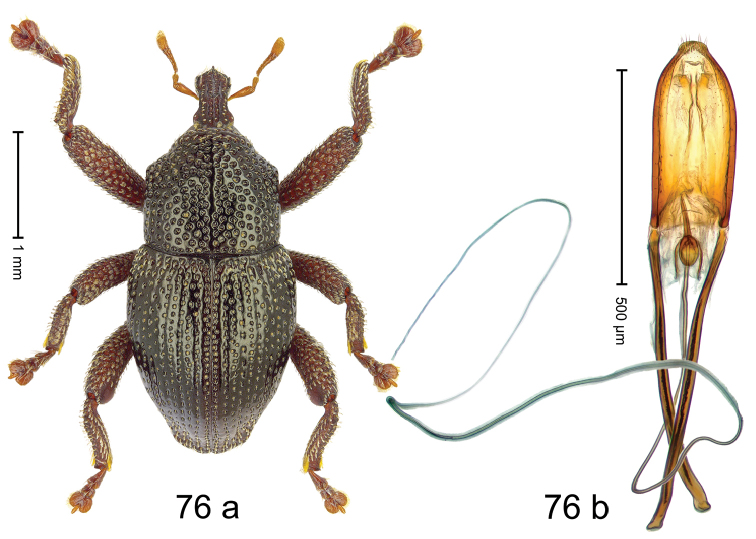
*Trigonopterusreticulatus* Riedel, sp. n., holotype; **a** habitus **b** penis.

***Holotype***, male (Fig. 76a). Length 3.06 mm. Color of antennae and legs ferruginous; remainder black. Body elongate; in dorsal aspect with distinct constriction between pronotum and elytron; in profile dorsally flat. Rostrum dorsally with median ridge and pair of submedian ridges; intervening furrows with rows of erect elongate scales; epistome short, with median denticle, laterally with two denticles on the right and with one denticle on the left of rostral apex. Pronotum with disk subquadrate, anteriorly forming angulation, before converging to weak subapical constriction; densely coarsely punctate; each puncture with fine, recumbent seta; interspaces between punctures reticulate, subglabrous; median line weakly costate, impunctate. Elytra densely irregularly punctate; striae 1–2 weakly impressed; apex subtruncate; apex and base with sparse scales. Foreleg relatively long. Femora with small acute tooth; anteroventral ridges of meso- and metafemur crenate; anterior surface coarsely punctate, reticulate, each puncture with long subclavate scale. Metafemur with dorsoposterior edge weakly denticulate; subapically with stridulatory patch. Dorsal edge of tibiae denticulate. Abdominal ventrites 1–2 weakly concave, with coarse punctures bearing erect elongate scales; ventrite 5 flat, coarsely punctate, with erect scales. Penis (Fig. 76b) with sides of body subparallel, converging to subangulate, sparsely setose apex; apodemes 2.0 × as long as body of penis; transfer apparatus spiniform, 0.5 × as long as body of penis; ductus ejaculatorius with indistinct bulbus. ***Intraspecific variation***. Length 1.95–3.25 mm. Female rostrum more slender, dorsally with rows of coarse punctures and weak longitudinal ridges; epistome indistinct. Female foreleg shorter.

#### Material examined.

***Holotype*** (MZB): ARC3239 (EMBL # LN884979), C-Sulawesi Prov., Ampana, Tanjung Api, 00°49.855'S 121°36.493'E, 166 m, sifted, 18-V-2013. ***Paratypes*** (MZB, SMNK, ZSM): C-Sulawesi Prov.: 38 exx, ARC3240 (GenBank # MK260480), ARC3241 (GenBank # MK260481), ARC3242 (GenBank # MK260482), ARC3243 (GenBank # MK260483), same data as holotype; 12 exx, Ampana, Tanjung Api, 00°49.771'S 121°36.527'E, 137 m, sifted, 18-V-2013; 26 exx, Ampana, Tanjung Api, 00°49.687'S 121°36.560'E, 130 m, sifted, 18-V-2013.

#### Distribution.

SE-Sulawesi Prov. (Ampana). Elevation 130–170 m.

#### Biology.

In leaf litter of lowland forest.

#### Etymology.

This epithet is the Latin adjective *reticulatus*, -*a*, -*um* (reticulated) and refers to the species´ punctate-reticulate integument.

#### Notes.

*Trigonopterusreticulatus* Riedel, sp. n. was coded as “*Trigonopterus* sp. 494”.

### 77. Trigonopterus rhombiformis

Taxon classificationAnimaliaColeopteraCurculionidae

Riedel sp. n.

http://zoobank.org/3E578B9D-3D2B-4E3B-89A4-14A27A3B86AB

#### Diagnostic description.

**Figure 77.**
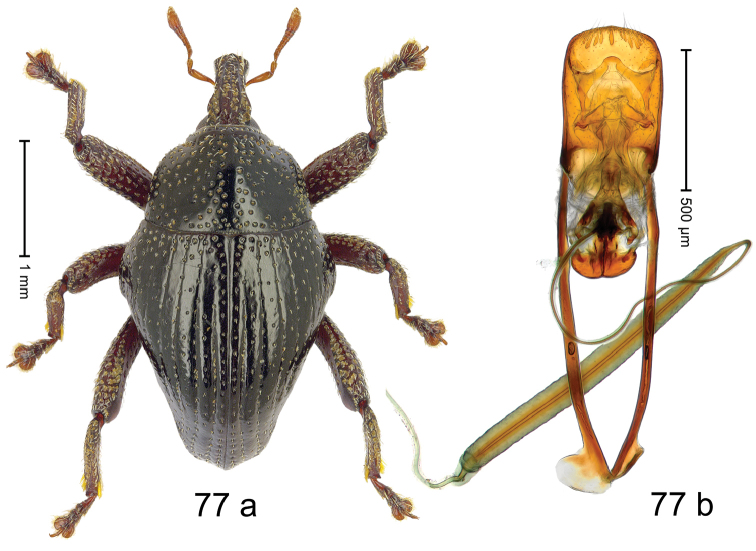
*Trigonopterusrhombiformis* Riedel, sp. n., holotype; **a** habitus **b** penis.

***Holotype***, male (Fig. 77a). Length 2.93 mm. Color of antennae ferruginous; legs dark ferruginous; remainder black. Body subrhomboid; in dorsal aspect humeri projecting, with weak constriction between pronotum and elytron; in profile dorsally convex. Rostrum dorsally in basal 1/2 with median and pair of submedian ridges, intervening furrows and anteriorly punctate, with sparse suberect elongate scales; epistome posteriorly with transverse ridge. Pronotum with lateral edges weakly converging, before apex rounded to subapical constriction; disk punctate, forming broad ridge with impunctate median line; in basal 1/2 with pair of subglabrous patches. Elytra with striae marked by weakly impressed lines and rows of fine punctures; intervals with interspersed punctures; along basal margin and swollen humeri with denser, larger punctures. Femora edentate. Metafemur with dorsoposterior edge denticulate; subapically with stridulatory patch. Metatibia in basal 1/3 slightly widened, dorsal edge denticulate. Abdominal ventrites 1–2 concave, subglabrous, with sparse erect subclavate scales; ventrite 5 almost flat, weakly concave, subglabrous. Penis (Fig. 77b) with sides of body subparallel; apex rounded, with sparse setae; apodemes 2.4 × as long as body of penis; transfer apparatus complex, anchor-shaped; ductus ejaculatorius with distinct bulbus.

#### Material examined.

***Holotype*** (MZB): ARC2795 (GenBank # MK260256), N-Sulawesi Prov., Airmadidi, Gn. Klabat, 01°26.430'N 125°00.622'E, 889 m, sifted, 17-V-2012.

#### Distribution.

N-Sulawesi Prov. (Mt Klabat). Elevation ca. 890 m.

#### Biology.

In leaf litter of montane forest.

#### Etymology.

This epithet is a combination of the Latin nouns *rhombus* (rhomb), *forma* (shape) and the 2^nd^ adjectival declension ending -*is*, and refers to the body shape of this species. A variable adjective.

#### Notes.

*Trigonopterusrhombiformis* Riedel, sp. n. was coded as “*Trigonopterus* sp. 387”.

### 78. Trigonopterus rotundatus

Taxon classificationAnimaliaColeopteraCurculionidae

Riedel sp. n.

http://zoobank.org/5F4A869E-8CB6-41C7-BDC2-0BD7AA113756

#### Diagnostic description.

**Figure 78.**
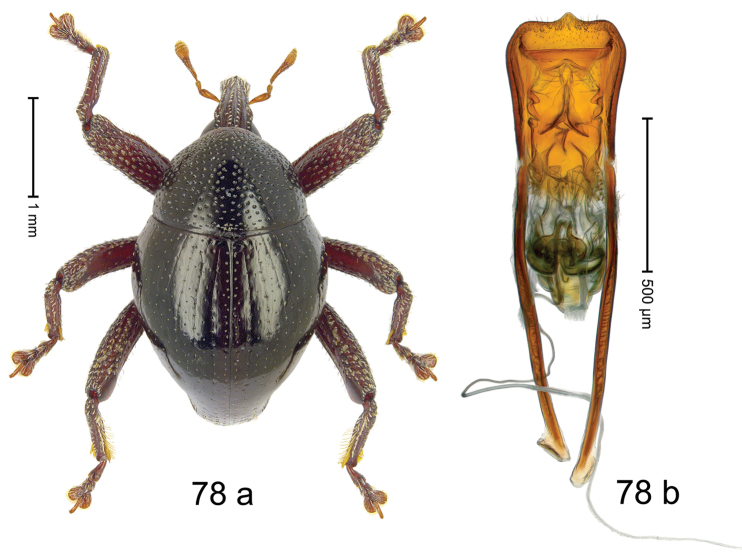
*Trigonopterusrotundatus* Riedel, sp. n., holotype; **a** habitus **b** penis.

***Holotype***, male (Fig. 78a). Length 3.00 mm. Color of antennae ferruginous; legs dark ferruginous; remainder black. Body subovate; in profile dorsally convex. Rostrum dorsally with median carina and pair of lower submedian ridges; intervening furrows each with sparse row of erect scales; epistome indistinct, with weak median denticle. Pronotum with disk densely punctate; interspaces between punctures subglabrous, subequal to punctures´ diameter; median line impunctate. Elytra with striae marked by minute punctures and indistinct hairlines; stria 8 along humerus with seven large punctures; intervals subglabrous, with sparse rows of minute punctures. Femora edentate; anteroventral ridges simple; anterior surface densely coarsely punctate, each puncture with subrecumbent, dorsally erect scale. Metafemur with dorsoposterior edge denticulate; subapically with stridulatory patch. Metatibia ventrally with sparse row of long, stiff setae. Abdominal ventrites 1–2 concave, subglabrous; ventrite 5 weakly concave, almost flat, coarsely punctate. Penis (Fig. 78b) with sides of body diverging; subapically rounded, with short median extension; apodemes 2.1 × as long as body of penis; endophallus with complex sclerites; transfer apparatus complex, largely composed of membranous lobes; ductus ejaculatorius with indistinct bulbus. ***Intraspecific variation***. Length 2.59–3.00 mm. Female rostrum dorsally with median and pair of submedian costae in basal 1/3; anteriorly slender, dorsally subglabrous, with rows of punctures.

#### Material examined.

***Holotype*** (MZB): ARC2848 (GenBank # MK260307), C-Sulawesi Prov., Lake Poso area, Pendolo, Gn. Sampuraga, 02°12.476'S 120°45.506'E, 1050 m, beaten, 31-V-2012. ***Paratypes*** (MZB, SMNK): S-Sulawesi Prov.: 1 ex, ARC3235 (GenBank # MK260306), Mangkutana, 02°20.203'S 120°46.878'E, 903 m, 14-V-2013, sifted; C-Sulawesi Prov., Lake Poso area: 8 exx, ARC3114 (GenBank # MK260310), ARC3115 (GenBank # MK260304), ARC3116 (GenBank # MK260305), Pendolo, Gn. Sampuraga, 02°12.165'S 120°45.567'E to 02°12.308'S 120°45.544'E, 934–1011 m, 13-V-2013, beaten; 1 ex, Pendolo, Gn. Sampuraga, 02°12.165'S 120°45.567'E, 934 m, 13-V-13, sifted; 1 ex, Pendolo, Gn. Sampuraga, 02°12.308'S 120°45.544'E, 1011 m, 13-V-13, sifted; 4 exx, ARC2876 (EMBL # LN884942), ARC2877 (GenBank # MK260309), ARC2878 (GenBank # MK260308), ARC6071 (GenBank # MK260303), Tentena, Taripa, 01°49.858'S 120°47.108'E to 01°50.040'S 120°46.666'E, 896–911 m, 02-VI-2012, beaten.

#### Distribution.

C-Sulawesi Prov. (Pendolo, Taripa); S-Sulawesi Prov. (Mangkutana). Elevation 900–1050 m.

#### Biology.

On foliage in montane forests.

#### Etymology.

This epithet is the Latin participle *rotundatus*, -*a*, -*um* (rounded) and refers to the species´ habitus.

#### Notes.

*Trigonopterusrotundatus* Riedel, sp. n. was coded as “*Trigonopterus* sp. 401”.

### 79. Trigonopterus rotundulus

Taxon classificationAnimaliaColeopteraCurculionidae

Riedel sp. n.

http://zoobank.org/EE30A008-80DF-4875-B7EC-1F8649396690

#### Diagnostic description.

**Figure 79.**
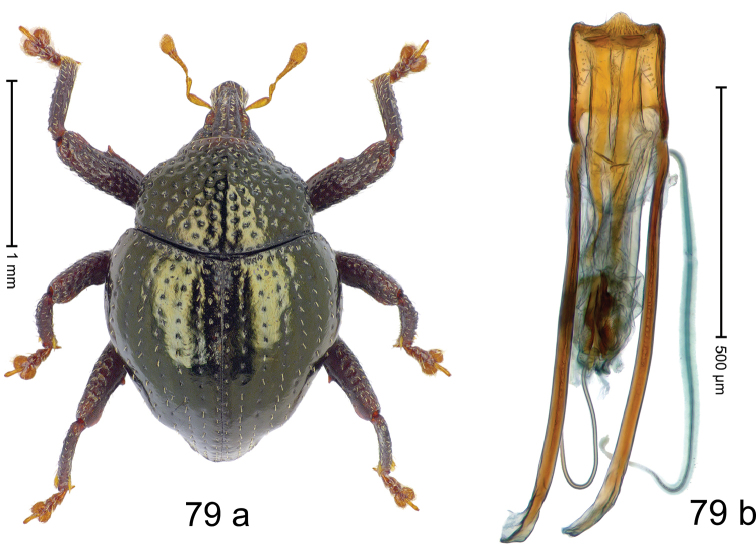
*Trigonopterusrotundulus* Riedel, sp. n., holotype; **a** habitus **b** penis.

***Holotype***, male (Fig. 79a). Length 2.02 mm. Color of antennae and tarsi ferruginous; remainder black, body with bronze luster. Body subglobose; in dorsal aspect with weak constriction between pronotum and elytron; in profile dorsally convex. Rostrum dorsally with median and pair of submedian ridges; intervening furrows with sparse rows of setae; epistome short, posteriorly with transverse, subangulate ridge. Pronotum with disk coarsely punctate; interspaces between punctures subglabrous, subequal to or smaller than punctures´ diameter; median line weakly costate. Elytra with striae marked by small punctures; each puncture bearing short subrecumbent seta; stria 8 near humerus with six larger punctures, externally bordered by costa; intervals subglabrous. Femora dentate; anteroventral ridges crenate, ending with acute tooth. Metafemur with indistinct posteroventral ridge bearing a second acute tooth; subapically with stridulatory patch. Abdominal ventrites 1–2 concave, subglabrous, microreticulate; ventrite 5 flat, sparsely punctate, microreticulate. Penis (Fig. 79b) with sides of body subparallel; apex subtruncate with subtriangular median extension; behind ostium with pair of elongate endophallic sclerites; apodemes 3.2 × as long as body of penis; transfer apparatus spiniform; ductus ejaculatorius with distinct bulbus. ***Intraspecific variation***. Length 1.98–2.02 mm. Female rostrum more slender, in apical 1/2 dorsally subglabrous, with sparse punctures and pair of sublateral furrows.

#### Material examined.

***Holotype*** (MZB): ARC3137 (GenBank # MK260441), C-Sulawesi Prov., Pendolo, Boe, 02°05.405'S 120°38.551'E, 857 m, sifted, 15-V-2013. ***Paratypes*** (MZB, SMNK): S-Sulawesi Prov.: 13 exx, ARC3136 (EMBL # LN884962), ARC3138 (GenBank # MK260443), ARC6017 (GenBank # MK260444), ARC6018 (GenBank # MK260440), ARC6019 (GenBank # MK260442), same data as holotype.

#### Distribution.

C-Sulawesi Prov. (Pendolo). Elevation ca. 860 m.

#### Biology.

In leaf litter of montane forest.

#### Etymology.

This epithet is the diminutive form of the Latin adjective *rotundatus*, -*a*, -*um* (rounded) and refers to the species´ body shape.

#### Notes.

*Trigonopterusrotundulus* Riedel, sp. n. was coded as “*Trigonopterus* sp. 483”.

### 80. Trigonopterus rudis

Taxon classificationAnimaliaColeopteraCurculionidae

Riedel sp. n.

http://zoobank.org/270C4D52-EA7A-4D3A-BC12-F78DA9E126CA

#### Diagnostic description.

**Figure 80.**
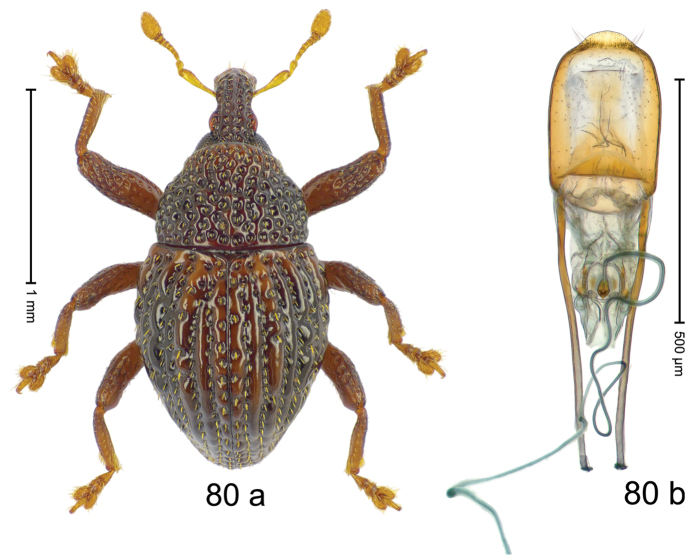
*Trigonopterusrudis* Riedel, sp. n., holotype; **a** habitus **b** penis.

***Holotype***, male (Fig. 80a). Length 1.76 mm. Color of antennae and legs ferruginous; elytra dorsally and near base ferruginous, remainder dark ferruginous to black. Body subovate; in dorsal aspect and in profile with distinct constriction between pronotum and elytron. Rostrum dorsally with median ridge and pair of submedian ridges; intervening furrows with coarse punctures containing each one suberect seta; epistome subglabrous. Pronotum with weak subapical constriction; disk densely, coarsely punctate; interspaces between punctures reticulate-rugose. Elytra with striae deeply impressed, intervals dorsally tuberculate-costate, subglabrous; intervals 1, 3, 5, 7 near base more distinctly swollen; punctures each with stout, suberect seta. Femora edentate. Anteroventral ridges simple; anterior surface of femora punctate-rugose. Metafemur subapically with stridulatory patch. Abdominal ventrite 1 weakly concave, with coarse punctures; ventrite 5 at middle with weak depression, coarsely punctate. Penis (Fig. 80b) with sides subparallel, apex subangulate, laterally with sparse setae; apodemes 2.3 × as long as body of penis; transfer apparatus dentiform, directed basad in repose; ductus ejaculatorius with indistinct bulbus. ***Intraspecific variation***. Length 1.76–1.96 mm. Female rostrum slender, dorsally with submedian and sublateral row of coarse punctures; without epistome. Female abdominal ventrite 5 flat, coarsely punctate.

#### Material examined.

***Holotype*** (MZB): ARC3079 (GenBank # MK260551), S-Sulawesi Prov., Tanah Toraja, Ponding, Baruppu, 02°47.918'S 119°47.408'E, 2233 m, sifted, 04-V-2013. ***Paratypes*** (ARC, MZB, SMNK): 3 exx, ARC3078 (GenBank # MK260549), ARC3080 (GenBank # MK260550), same data as holotype; 1 ex, Ponding, Baruppu, 02°48.010'S 119°47.560'E, 2199 m, sifted, 04-V-2013; 2 exx, Tanah Toraja, Pulu Pulu, 1700 m, 13–16-VIII-1990, sifted; 2 exx, Tanah Toraja, Pulu Pulu, 2400 m, 15–16-VIII-1990, sifted.

#### Distribution.

S-Sulawesi Prov. (Tanah Toraja). Elevation 1700–2400 m.

#### Biology.

In leaf litter of montane forest.

#### Etymology.

This epithet is the Latin adjective *rudis*, -*e* (coarse, rough) and refers to the species´ sculpture.

#### Notes.

*Trigonopterusrudis* Riedel, sp. n. was coded as “*Trigonopterus* sp. 519”.

### 81. Trigonopterus rufipes

Taxon classificationAnimaliaColeopteraCurculionidae

Riedel sp. n.

http://zoobank.org/BB886D1C-80E9-42A6-867B-8DBDEA43F575

#### Diagnostic description.

**Figure 81.**
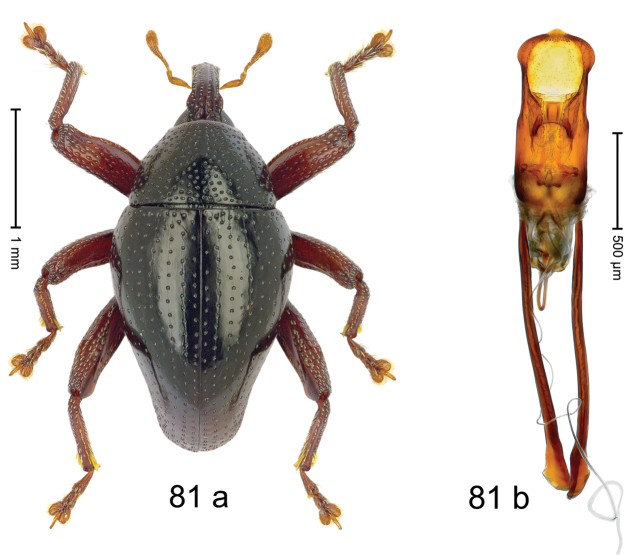
*Trigonopterusrufipes* Riedel, sp. n., holotype; **a** habitus **b** penis.

***Holotype***, male (Fig. 81a). Length 2.84 mm. Color of antennae and legs ferruginous; remainder black. Body subovate; in profile dorsally convex. Rostrum dorsally with median and pair of submedian costae; intervening furrows with sparse rows of suberect setae; epistome indistinct. Pronotum with disk densely punctate with small punctures; interspaces between punctures subglabrous. Elytra with striae marked by small punctures and fine hairlines; stria 8 along humerus with punctures larger; intervals subglabrous, with few interspersed minute punctures. Femora edentate; anteroventral ridges simple. Metafemur dorsally sparsely covered with recumbent silvery scales; dorsoposterior edge simple; subapically with stridulatory patch. Abdominal ventrites 1–2 concave, subglabrous; behind metacoxa with small lateral flange, with sparse suberect scales; ventrite 5 with subglabrous impression, laterally with sparse coarse punctures and sparse erect scales, subapically with dense erect setae. Penis (Fig. 81b) with sides of body subparallel; at middle with shallow constriction; rounded to subangulate apex, without setae; apodemes 2.3 × as long as body of penis; transfer apparatus flagelliform, 1.7 × as long as body of penis; ductus ejaculatorius without bulbus.

#### Material examined.

***Holotype*** (MZB): ARC2999 (EMBL # LN884948), S-Sulawesi Prov., Tanah Toraja, Rantepao, Gn. Karre (= Gn. Wokim), 02°59.021'S 120°02.523'E, 1456 m, beaten, 06-V-2013.

#### Distribution.

S-Sulawesi Prov. (Tanah Toraja). Elevation ca. 1460 m.

#### Biology.

On foliage in montane forests.

#### Etymology.

This epithet is a combination of the Latin adjective *rufus*, -*a*, -*um* (red) and noun pes (foot, leg).

#### Notes.

*Trigonopterusrufipes* Riedel, sp. n. was coded as “*Trigonopterus* sp. 515”.

### 82. Trigonopterus sampunensis

Taxon classificationAnimaliaColeopteraCurculionidae

Riedel sp. n.

http://zoobank.org/B83F5594-7FC8-45BA-897B-BD6D30998C75

#### Diagnostic description.

**Figure 82.**
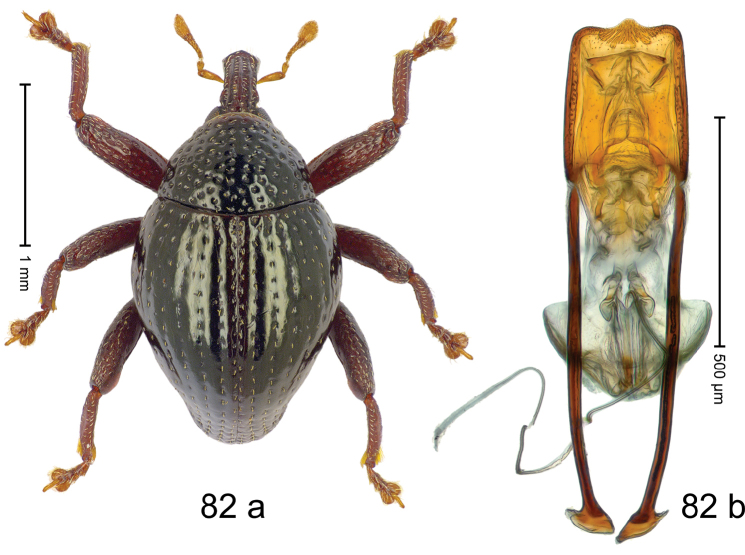
*Trigonopterussampunensis* Riedel, sp. n., holotype; **a** habitus **b** penis.

***Holotype***, male (Fig. 82a). Length 2.14 mm. Color of antennae ferruginous; legs and head dark ferruginous; remainder black. Body subovate; in profile dorsally convex. Rostrum dorsally with median and pair of submedian ridges; intervening furrows with sparse rows of setae; epistome posteriorly with transverse ridge. Pronotum with disk densely punctate; interspaces between punctures subglabrous, subequal to punctures´ diameter; median line impunctate. Elytra with striae marked by small punctures; stria 8 near humerus with slightly larger punctures; suture bordered by row of small punctures; intervals subglabrous. Anteroventral ridge of mesofemur and metafemur ending with small blunt tooth. Metafemur with simple dorsoposterior edge, dorsally with sparse suberect transparent scales; subapically with stridulatory patch. Abdominal ventrites 1–2 concave, subglabrous, microreticulate; ventrite 2 laterally forming flange, in profile subangularly projecting; ventrite 5 subglabrous, sparsely punctate, at middle with round pit. Penis (Fig. 82b) with sides of body subparallel; apex subtruncate with short median extension; apodemes 2.9 × as long as body of penis; transfer apparatus flagelliform, directed basad in repose, subequal in length to body of penis; ductus ejaculatorius with indistinct bulbus. ***Intraspecific variation***. Length 2.04–2.50 mm.

#### Material examined.

***Holotype*** (MZB): ARC2837 (GenBank # MK260258), S-Sulawesi Prov., Pc. Palopo, Gn. Sampuna, 02°56.545'S 120°05.595'E, 1101 m, sifted, 29-V-2012. ***Paratypes*** (MZB, SMNK): S-Sulawesi Prov.: 3 exx, ARC3090 (GenBank # MK260259), ARC3091 (GenBank # MK260257), ARC3092 (GenBank # MK260260), same data as holotype.

#### Distribution.

S-Sulawesi Prov. (Pc. Palopo). Elevation ca. 1100 m.

#### Biology.

In leaf litter of montane forest.

#### Etymology.

This epithet is a Latinized adjective based on the type locality, Mt Sampuna.

#### Notes.

*Trigonopterussampunensis* Riedel, sp. n. was coded as “*Trigonopterus* sp. 388”.

### 83. Trigonopterus sampuragensis

Taxon classificationAnimaliaColeopteraCurculionidae

Riedel sp. n.

http://zoobank.org/B9B6D6BC-9BD4-4486-9B07-40F4BCD0433B

#### Diagnostic description.

**Figure 83.**
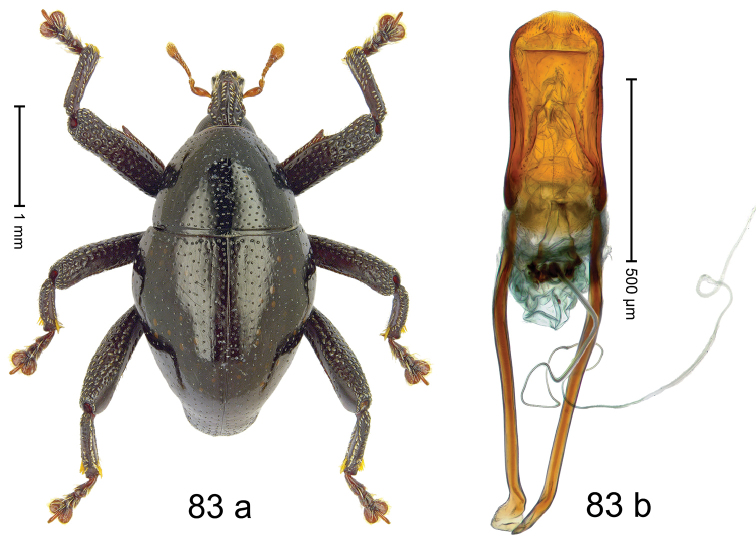
*Trigonopterussampuragensis* Riedel, sp. n., holotype; **a** habitus **b** penis.

***Holotype***, male (Fig. 83a). Length 3.22 mm. Color of antennae ferruginous; tarsi dark ferruginous; remainder black. Body subovate; in dorsal aspect with indistinct constriction between pronotum and elytron; in profile dorsally convex. Rostrum in basal 1/2 dorsally with glabrous median carina and pair of submedian ridges; at level of antennal insertion median carina abruptly terminated by bottom of cup-shaped ridge; with sparse suberect yellowish scales; epistome sparsely punctate, posteriorly with pair of sublateral and pair of submedian denticles. Pronotum with disk densely punctate with small punctures; interspaces between punctures subglabrous. Elytra irregularly punctate with small punctures; striae hardly visible; stria 8 near humerus with larger punctures. Femora with anteroventral ridge terminating with acute tooth, in meso- and metafemur anteroventral ridge crenate; anterior surface of femora coarsely punctate, each puncture with recumbent silvery scale. Metafemur with dorsoposterior edge simple, without denticles; subapically with stridulatory patch. Abdominal ventrites 1–2 concave, subglabrous; ventrite 5 at middle with distinct depression, concave, sparsely punctate, sublaterally densely punctate. Penis (Fig. 83b) with sides of body subparallel, apex rounded, without setae; apodemes 1.9 × as long as body of penis; transfer apparatus supported by lyriform sclerite; ductus ejaculatorius without bulbus. ***Intraspecific variation***. Length 3.22–3.44 mm.

#### Material examined.

***Holotype*** (MZB): ARC2847 (EMBL # LN884936), C-Sulawesi Prov., Pendolo, Gn. Sampuraga, 02°12.476'S 120°45.506'E, 1050 m, beaten, 31-V-2012. ***Paratypes*** (MZB, SMNK): C-Sulawesi Prov.: 4 exx, ARC3112 (GenBank # MK260376), ARC3113 (GenBank # MK260377), Pendolo, Gn. Sampuraga, 02°12.165'S 120°45.567'E to 02°12.308'S 120°45.544'E, 934–1011 m, beaten, 13-V-2013.

#### Distribution.

C-Sulawesi Prov. (Pendolo). Elevation 1010–1050 m.

#### Biology.

On foliage in montane forests.

#### Etymology.

This epithet is a Latinized adjective based on the type locality.

#### Notes.

*Trigonopterussampuragensis* Riedel, sp. n. was coded as “*Trigonopterus* sp. 417”.

### 84. Trigonopterus satyrus

Taxon classificationAnimaliaColeopteraCurculionidae

Riedel sp. n.

http://zoobank.org/CDF5F279-1E5F-4B1B-8EEB-084BCCFD0A35

#### Diagnostic description.

**Figure 84.**
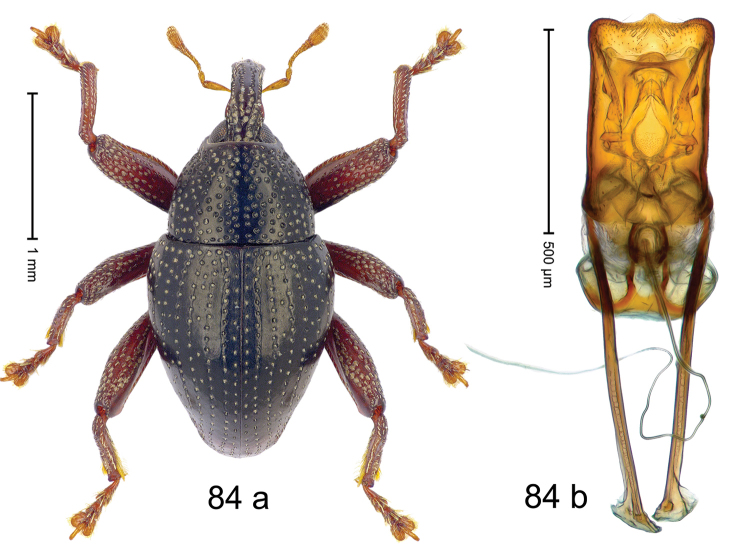
*Trigonopterussatyrus* Riedel, sp. n., holotype; **a** habitus **b** penis.

***Holotype***, male (Fig. 84a). Length 2.24 mm. Color of antennae and legs ferruginous; remainder black. Body subovate; in dorsal aspect with weak constriction between pronotum and elytron; in profile dorsally convex. Rostrum dorsally with somewhat flattened median costa and pair of submedian costae; intervening furrows with sparse rows of recumbent setae; apical 1/3 subglabrous, with few punctures and with sparse setae. Pronotum with disk densely punctate with coarse punctures except along impunctate median line; interspaces between punctures subglabrous, microreticulate, subequal to or smaller than punctures´ diameter. Elytra with striae marked by punctures and fine hairlines; basal margin bordered by transverse row; intervals flat, microreticulate, with few interspersed punctures. Femora edentate; anteroventral ridges simple. Metafemur dorsally with sparse, recumbent, silvery scales; dorsoposterior edge simple; subapically with stridulatory patch. Metatibia ventrally with sparse row of setae. Abdominal ventrites 1–2 concave, subglabrous, sparsely punctate; ventrite 5 at middle with distinct, broadly ovate impression, densely punctate. Penis (Fig. 84b) with sides of body subparallel; apex subtruncate, with median angulate extension, with sparse setae; apodemes 1.8 × as long as body of penis; transfer apparatus spiniform, directed basad in repose, attached to anchor-shaped supporting sclerite; ductus ejaculatorius without bulbus. ***Intraspecific variation***. Length 2.24–2.56 mm. Female rostrum slender, subglabrous, with two submedian rows of punctures, with pair of sublateral furrows.

#### Material examined.

***Holotype*** (MZB): ARC3055 (EMBL # LN884955), S-Sulawesi Prov., Tanah Toraja, Bittuang, Gn. Karoa, 02°55.270'S 119°40.179'E to 02°55.032'S 119°40.365'E, 1800–2000 m, beaten, 10-V-2013. ***Paratype*** (SMNK): 1 ex, ARC3056 (GenBank # MK260542), same data as holotype.

#### Distribution.

S-Sulawesi Prov. (Tanah Toraja). Elevation ca. 1800–2000 m.

#### Biology.

On foliage in montane forests.

#### Etymology.

This epithet is the name of Satyrus, a forest-god of the train of Bacchus. It is treated as a noun in apposition.

#### Notes.

*Trigonopterussatyrus* Riedel, sp. n. was coded as “*Trigonopterus* sp. 516”.

### 85. Trigonopterus scabripes

Taxon classificationAnimaliaColeopteraCurculionidae

Riedel sp. n.

http://zoobank.org/796E679F-62B9-4E62-818D-5618D4C03FBF

#### Diagnostic description.

**Figure 85.**
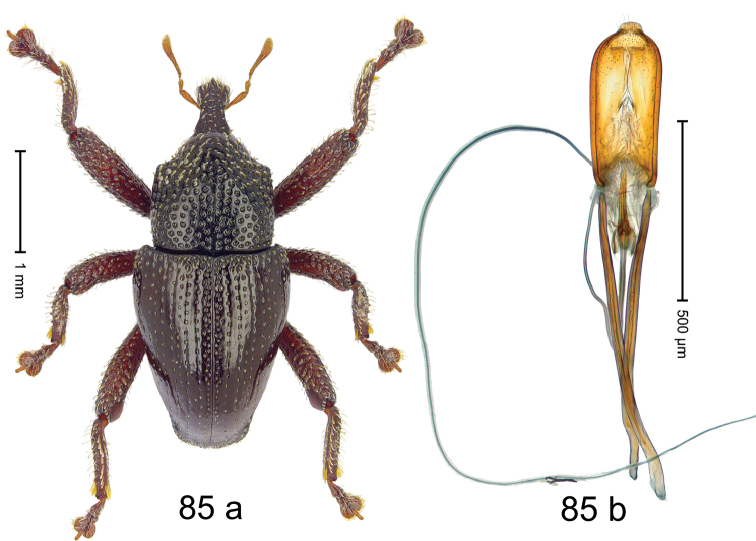
*Trigonopterusscabripes* Riedel, sp. n., holotype; **a** habitus **b** penis.

***Holotype***, male (Fig. 85a). Length 3.14 mm. Color of antennae and legs ferruginous; remainder black. Body elongate; in dorsal aspect with distinct constriction between pronotum and elytron; in profile dorsally flat. Rostrum dorsally with median ridge and pair of submedian ridges; intervening furrows with rows of erect subclavate scales; epistome short, with median denticle, laterally with two denticles on the right and with one denticle on the left of rostral apex. Pronotum with disk subquadrate, anteriorly forming angulation, before converging to weak subapical constriction; densely coarsely punctate; interspaces between punctures subglabrous; in anterior 1/2 with erect subclavate scales; median line weakly costate, impunctate. Elytra densely irregularly punctate; along basal margin and humeri with punctation more densely; striae marked by fine hairlines; apex subtruncate; apex and base with sparse scales. Foreleg relatively long. Femora with small acute tooth; anteroventral ridges of meso- and metafemur weakly crenate; anterior surface coarsely punctate, reticulate, each puncture with long subclavate scale. Metafemur subapically with stridulatory patch. Dorsal edge of tibiae serrate. Abdominal ventrites 1–2 concave, microreticulate, with sparse punctures bearing erect elongate scales; ventrite 5 flat, coarsely punctate, with erect scales. Penis (Fig. 85b) with sides of body subparallel, converging to slightly spatulate, sparsely setose apex; apodemes 2.1 × as long as body of penis; transfer apparatus spiniform, 0.6 × as long as body of penis; ductus ejaculatorius with indistinct bulbus. ***Intraspecific variation***. Length 2.20–3.14 mm. Female rostrum more slender, dorsal ridges less distinct, sometimes punctate-reticulate, epistome indistinct. Female foreleg shorter.

#### Material examined.

***Holotype*** (MZB): ARC3173 (EMBL # LN884968), SE-Sulawesi Prov., N Kolaka, Uluwolo village, 03°48.073'S 121°16.856'E, 182 m, sifted, 21-IV-2013. ***Paratypes*** (MZB, SMNK): SE-Sulawesi Prov.: 39 exx, ARC3174 (GenBank # MK260476), ARC3175 (GenBank # MK260477), ARC3176 (GenBank # MK260478), ARC3177 (GenBank # MK260473), ARC3178 (GenBank # MK260479), ARC6010 (GenBank # MK260474), ARC6011 (GenBank # MK260475), same data as holotype.

#### Distribution.

SE-Sulawesi Prov. (Kolaka). Elevation ca. 180 m.

#### Biology.

In leaf litter of lowland forest.

#### Etymology.

This epithet is a combination of the Latin adjective *scaber*, -*a*, -*um* (rough) and the noun *pes* (foot, leg) and refers to sculpture and vestiture of the species´ legs. It is to be treated as a noun in apposition.

#### Notes.

*Trigonopterusscabripes* Riedel, sp. n. was coded as “*Trigonopterus* sp. 493”.

### 86. Trigonopterus scaphiformis

Taxon classificationAnimaliaColeopteraCurculionidae

Riedel sp. n.

http://zoobank.org/9C1EB881-939C-49D3-A2B3-21CB34FD772D

#### Diagnostic description.

**Figure 86.**
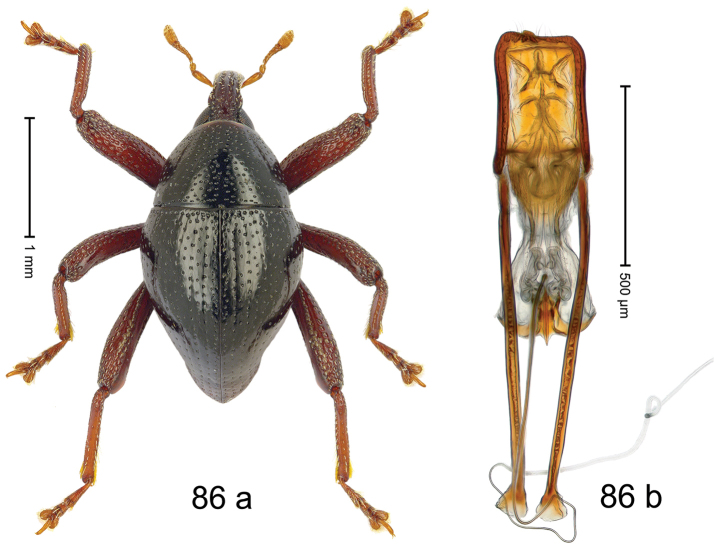
*Trigonopterusscaphiformis* Riedel, sp. n., holotype; **a** habitus **b** penis.

***Holotype***, male (Fig. 86a). Length 2.43 mm. Color of antennae and legs ferruginous; remainder black. Body subovate; in profile dorsally convex. Rostrum dorsally in basal 1/2 with median and pair of submedian costae; intervening furrows with sparse rows of recumbent piliform scales; apical 1/3 subglabrous, sparsely setose. Pronotum with disk densely punctate with small punctures; interspaces between punctures subglabrous. Elytra irregularly punctate with small punctures, along basal margin with transverse row of denser punctures; some striae marked by hardly visible hairlines; intervals flat; stria 8 along humerus with six larger punctures; apex markedly angulate. Legs long. Femora edentate. Meso- and metafemur with anteroventral ridge crenate. Metafemur dorsally with row of recumbent, silvery scales; subapically with stridulatory patch. Metatibia ventrally with row of sparse, thin setae. Abdominal ventrites 1–2 concave, subglabrous; ventrite 5 at middle with ovate subglabrous pit laterally bordered by ridges, subapically with denticle. Penis (Fig. 86b) with sides of body subparallel; apex asymmetrical, with short extension on right side, with sparse setae; apodemes 2.3 × as long as body of penis; transfer apparatus spiniform, directed basad in repose, attached to anchor-shaped supporting sclerite; ductus ejaculatorius without bulbus. ***Intraspecific variation***. Length 2.35–2.43 mm. Female rostrum slender, dorsally subglabrous, with two submedian rows of punctures, with pair of sublateral furrows. Female abdominal ventrite 5 flat, medially subglabrous, laterally punctate and sparsely setose.

#### Material examined.

***Holotype*** (MZB): ARC2997 (GenBank # MK260489), S-Sulawesi Prov., Tanah Toraja, Rantepao, Gn. Karre (= Gn. Wokim), 02°59.021'S 120°02.523'E, 1456 m, beaten, 06-V-2013. ***Paratypes*** (MZB, SMNK): 4 exx, ARC3005 (EMBL # LN884949), ARC3006 (GenBank # MK260492), ARC5990 (GenBank # MK260493), same data as holotype; 5 exx, ARC3040 (GenBank # MK260491), ARC3041 (GenBank # MK260490), ARC3042 (GenBank # MK260488), Tanah Toraja, Bittuang, Gn. Ponding, 02°56.446'S 119°38.075'E, 1625 m, beaten, 09-V-2013; 1 ex, Tanah Toraja, Bittuang, Gn. Ponding, 02°56.446'S 119°38.075'E, 1625 m, sifted, 09-V-2013.

#### Distribution.

S-Sulawesi Prov. (Tanah Toraja). Elevation 1460–1625 m.

#### Biology.

On foliage in montane forests.

#### Etymology.

This epithet is a combination of the Latin nouns *scapha* (boat, skiff), *forma* (shape) and the 2^nd^ adjectival declension ending -*is*, and refers to the body shape of this species.

#### Notes.

*Trigonopterusscaphiformis* Riedel, sp. n. was coded as “*Trigonopterus* sp. 497”.

### 87. Trigonopterus scitulus

Taxon classificationAnimaliaColeopteraCurculionidae

Riedel sp. n.

http://zoobank.org/A4B1DA07-7FAD-4A7F-AED2-C32E9CED6A31

#### Diagnostic description.

**Figure 87.**
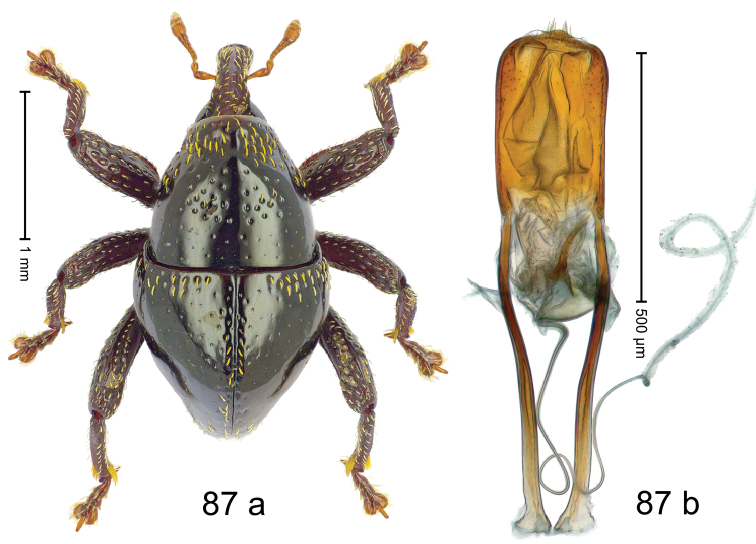
*Trigonopterusscitulus* Riedel, sp. n., holotype; **a** habitus **b** penis.

***Holotype***, male (Fig. 87a). Length 2.26 mm. Color of antennae, tibiae, tarsi and rostrum ferruginous; femora dark ferruginous to black; remainder black. Body subovate; in profile dorsally convex, with distinct constriction between pronotum and elytron. Rostrum dorsally with median subglabrous carina, pair of submedian ridges indistinct; with sparse rows of erect piliform yellow scales; epistome short, subglabrous. Pronotum subglabrous, at middle, subapically and laterally with sparse coarse punctures; subapically with sparse yellowish narrow suberect scales. Elytra subglabrous, with sparse minute punctures, striae obsolete; with sparse recumbent yellowish scales near base, on sutural interval and subapically. Femora edentate; anteroventral ridge distinct, simple. Metafemur with dorsoposterior ridge denticulate, subapically without stridulatory patch, anterior surface coarsely rugose-punctate, each puncture with narrow recumbent scale. Abdominal ventrites 1–2, fused, weakly concave, subglabrous; ventrite 5 with distinct depression, sublaterally with sparse erect piliform scales. Penis (Fig. 87b) with sides of body subparallel, apex subangulate, with sparse short setae; apodemes 2.1 × as long as body of penis; transfer apparatus simple, spiniform; ductus ejaculatorius with indistinct bulbus. ***Intraspecific variation***. Length 2.06–2.26 mm. Female unknown.

#### Material examined.

***Holotype*** (MZB): ARC2788 (GenBank # MK260393), N-Sulawesi Prov., Airmadidi, Gn. Klabat, 01°26.635'N 125°00.823'E, 1031 m, sifted, 16-V-2012. ***Paratype*** (SMNK): 1 ex, ARC2802 (EMBL # LT603141), N-Sulawesi, Airmadidi, Gn. Klabat, 01°26.619'N 125°00.805'E, 1018 m, sifted, 17-V-2012.

#### Distribution.

N-Sulawesi Prov. (Gn. Klabat). Elevation ca. 1020–1030 m.

#### Biology.

In leaf litter of montane forest.

#### Etymology.

This epithet is the Latin adjective *scitulus*, -*a*, -*um* (neat).

#### Notes.

*Trigonopterusscitulus* Riedel, sp. n. was coded as “*Trigonopterus* sp. 422”.

### 88. Trigonopterus selayarensis

Taxon classificationAnimaliaColeopteraCurculionidae

Riedel sp. n.

http://zoobank.org/251C9B66-F9E0-42B4-9DEC-9A0C1DE91E5B

#### Diagnostic description.

**Figure 88.**
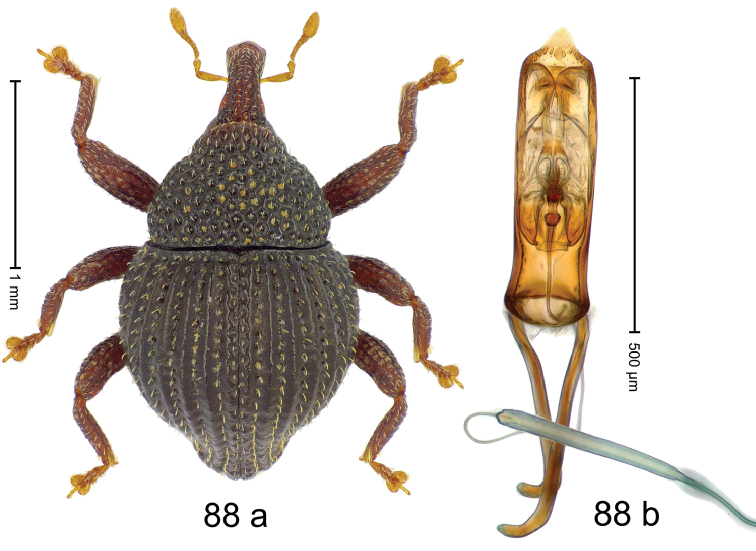
*Trigonopterusselayarensis* Riedel, sp. n., holotype; **a** habitus **b** penis.

***Holotype***, male (Fig. 88a). Length 1.98 mm. Color of antennae ferruginous; legs and head dark ferruginous; remainder black. Body subrotund; in dorsal aspect with distinct constriction between pronotum and elytron, in profile with weaker constriction. Rostrum dorsally with distinct median ridge and pair of sublateral ridges; with sparse rows of suberect setae; epistome short, sparsely punctate, posteriorly with transverse, angulate ridge. Pronotum with subapical constriction; disk coarsely punctate, scabrous; each puncture with erect, curved seta. Elytra with striae deeply impressed; with rows of erect, weakly clavate, curved scales; intervals carinate, subglabrous, microreticulate. Meso- and metafemur with minute blunt denticle, profemur edentate. Metafemur subapically without stridulatory patch. Abdominal ventrite 5 flat, microreticulate, punctate, sparsely setose with long setae. Penis (Fig. 88b) hardly asymmetrical; tip medially extended; basal orifice ventrally simple; apodemes subequal to body of penis (1.0 ×); ductus ejaculatorius with distinct bulbus. ***Intraspecific variation***. Length 1.68–2.14 mm. Body subrotund in larger specimens to subovate in smaller specimens. Female rostrum more slender, dorsally punctate-rugose, epistome indistinct.

#### Material examined.

***Holotype*** (MZB): ARC3186 (GenBank # MK260407), S-Sulawesi Prov., Selayar Is, Pagarangan, 06°18.334'S 120°30.794'E, 545 m, sifted, 24-IV-2013. ***Paratypes*** (MZB, SMNK): 7 exx, ARC3185 (GenBank # MK260409), ARC3187 (GenBank # MK260408), same data as holotype.

#### Distribution.

S-Sulawesi Prov. (Selayar Is). Elevation ca. 545 m.

#### Biology.

In leaf litter of lowland forest.

#### Etymology.

This epithet is a Latinized adjective based on the type locality Selayar Island.

#### Notes.

*Trigonopterusselayarensis* Riedel, sp. n. was coded as “*Trigonopterus* sp. 467”.

### 89. Trigonopterus serripes

Taxon classificationAnimaliaColeopteraCurculionidae

Riedel sp. n.

http://zoobank.org/41B24644-E9B5-4F85-83E6-BC94150CA9CC

#### Diagnostic description.

**Figure 89.**
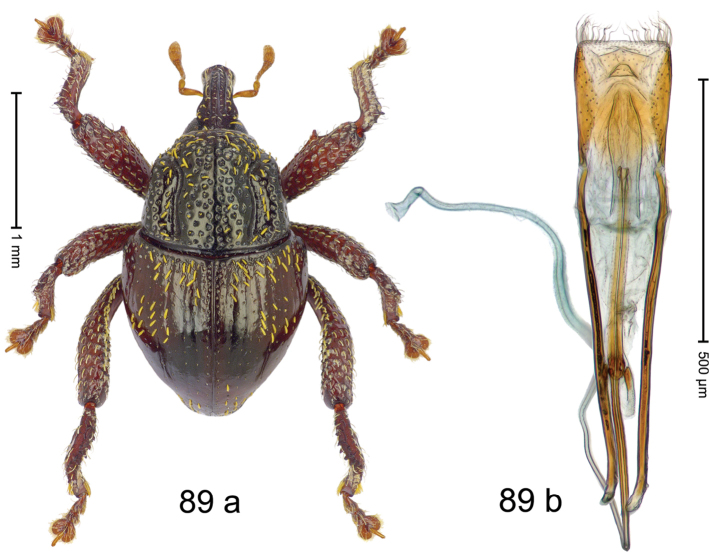
*Trigonopterusserripes* Riedel, sp. n., holotype; **a** habitus **b** Aedeagus.

***Holotype***, male (Fig. 89a). Length 2.28 mm. Color of antennae and legs ferruginous; elytral humeri dark ferruginous; remainder black. Body subovate; in dorsal aspect with moderate constriction between pronotum and elytron; in profile dorsally convex. Rostrum dorsally with median carina and pair of submedian ridges; intervening furrows with sparse, erect scales; epistome short, posteriorly with transverse ridge. Pronotum with lateral edges weakly converging, with weak subapical constriction; disk with pair of distinct longitudinal impressions; medially broadly swollen, densely coarsely punctate; interspaces subglabrous, with narrow median costa; with scattered yellow elongate scales. Elytra sparsely punctate with minute punctures; striae marked by fine hairlines; in basal 1/3, at middle and subapically with sparse yellow recumbent scales. Femora dentate, with acute tooth; anteroventral ridge of metafemur crenate. Metafemur with dorsoposterior edge denticulate; subapically with stridulatory patch. Meso- and metatibia in basal 1/2 widened, dorsal edge serrate. Metatibia curved. Abdominal ventrites 1–2 concave, subglabrous, sparsely punctate, with few scattered piliform scales; ventrite 5 weakly concave, punctate, with sparse erect setae. Penis (Fig. 89b) with sides of body weakly diverging; apex subtruncate, with fringe of long curly setae; apodemes 2.9 × as long as body of penis; transfer apparatus flagelliform, 1.8 × longer than body of penis; ductus ejaculatorius with indistinct bulbus. ***Intraspecific variation***. Length 2.28–2.36 mm. Female rostrum slender, in apical 1/2 with submedian row of punctures and sublateral furrows; epistome simple. Female ventrite 5 flat.

#### Material examined.

***Holotype*** (MZB): ARC3170 (GenBank # MK260591), SE-Sulawesi Prov., N Kolaka, Uluwolo village, 03°48.073'S 121°16.856'E, 182 m, sifted, 21-IV-2013. ***Paratypes*** (MZB, SMNK): 14 exx, ARC3171 (GenBank # MK260590), ARC3172 (GenBank # MK260592), same data as holotype.

#### Distribution.

SE-Sulawesi Prov. (Kolaka). Elevation ca. 180 m.

#### Biology.

In leaf litter of lowland forest.

#### Etymology.

This epithet is a combination of the Latin noun *serra* (a saw) and the noun *pes* (foot, leg) and is to be treated as noun in apposition.

#### Notes.

*Trigonopterusserripes* Riedel, sp. n. was coded as “*Trigonopterus* sp. 533”.

### 90. Trigonopterus seticnemis

Taxon classificationAnimaliaColeopteraCurculionidae

Riedel sp. n.

http://zoobank.org/8AA861C3-1CCC-4EE8-84B3-71ECD1219238

#### Diagnostic description.

**Figure 90.**
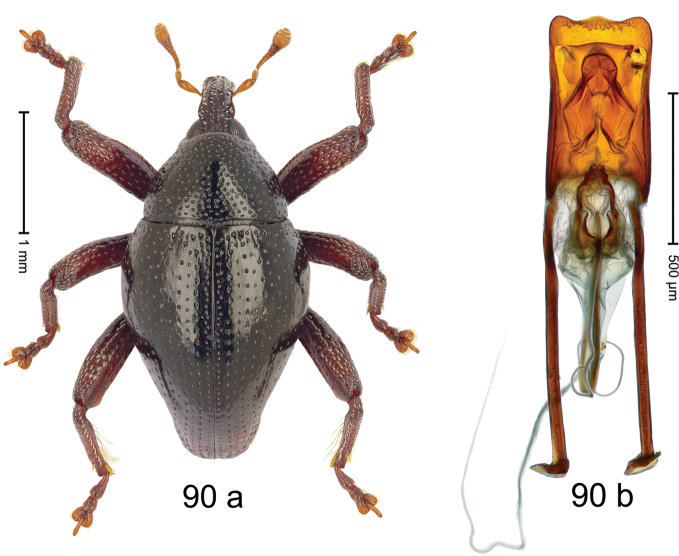
*Trigonopterusseticnemis* Riedel, sp. n., holotype; **a** habitus **b** penis.

***Holotype***, male (Fig. 90a). Length 2.79 mm. Color of antennae and legs ferruginous; remainder black. Body subovate; in dorsal aspect and in profile with weak constriction between pronotum and elytron. Rostrum dorsally with median and pair of submedian costae; intervening furrows with sparse rows of suberect scales; epistome indistinct. Pronotum with disk densely punctate with small punctures; interspaces between punctures subglabrous; median line glabrous. Elytra with striae marked by small punctures; intervals flat, with similar rows of slightly smaller punctures; stria 8 along humerus with short row of six coarse punctures, externally bordered by costa. Femora edentate. Meso- and metafemur with anteroventral ridge crenate. Metafemur dorsally punctate, with recumbent, silvery scales; subapically with stridulatory patch. Metatibia ventrally with sparse row of long setae. Abdominal ventrites 1–2 concave, subglabrous, with sparse coarse punctures each containing a scale; ventrite 5 with median, subglabrous, sparsely punctate impression, sublaterally weakly swollen, coarsely punctate, with sparse scales. Penis (Fig. 90b) with sides of body subparallel; apex subangulate, nude; apodemes 1.8 × as long as body of penis; transfer apparatus flagelliform, 2.0 × longer than body of penis, subapically held by lyriform supporting sclerite; ductus ejaculatorius with indistinct bulbus.

#### Material examined.

***Holotype*** (MZB): ARC2998 (GenBank # MK260528), S-Sulawesi Prov., Tanah Toraja, Rantepao, Gn. Karre (= Gn. Wokim), 02°59.021'S 120°02.523'E, 1456 m, beaten, 06-V-2013.

#### Distribution.

S-Sulawesi Prov. (Tanah Toraja). Elevation ca. 1460 m.

#### Biology.

On foliage in montane forests.

#### Etymology.

This epithet is a combination of the Latin nouns *seta* (bristle) and Greek Latinized noun *cnemis* (greave) and refers to the species´ metatibia.

#### Notes.

*Trigonopterusseticnemis* Riedel, sp. n. was coded as “*Trigonopterus* sp. 508”.

### 91. Trigonopterus silvicola

Taxon classificationAnimaliaColeopteraCurculionidae

Riedel sp. n.

http://zoobank.org/46339435-D59E-4C4E-9462-0FA3375AD0F6

#### Diagnostic description.

**Figure 91.**
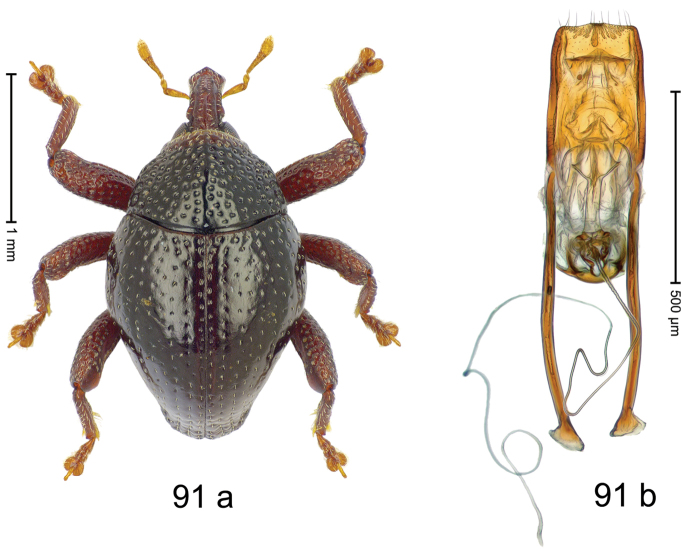
*Trigonopterussilvicola* Riedel, sp. n., holotype; **a** habitus **b** penis.

***Holotype***, male (Fig. 91a). Length 2.20 mm. Color of legs and head ferruginous; remainder black. Body subovate; in dorsal aspect with weak constriction between pronotum and elytron; in profile dorsally convex. Rostrum in front of eye with distinct lateral constriction; dorsally with median and pair of submedian ridges; intervening furrows with sparse rows of subrecumbent setae; epistome short, posteriorly with transverse, angulate ridge. Pronotum with disk densely coarsely punctate; each puncture containing fine, recumbent seta; with impunctate median ridge; towards sides punctures becoming larger, with reticulate interspaces. Elytra with striae marked by small punctures, each with indistinct recumbent seta; intervals subglabrous, with few interspersed punctures. Femora edentate. Meso- and metafemur with anteroventral ridge crenate. Metafemur with dorsoposterior edge weakly denticulate-crenate; subapically with stridulatory patch. Metatibia at middle ventrally with short row of setae. Abdominal ventrite 1 concave, sparsely punctate, microreticulate; ventrite 5 punctate, at middle and sublaterally with shallow depressions. Penis (Fig. 91b) with sides of body subparallel; apex subangulate, with short median extension, sparsely setose; apodemes 2.4 × as long as body of penis; transfer apparatus complex; ductus ejaculatorius without bulbus. ***Intraspecific variation***. Length 2.14–2.24 mm. Female rostrum in apical 1/2 subglabrous with submedian rows of punctures and sublateral furrows; without distinct epistome. Female metatibia ventrally simple, with sparse setae.

#### Material examined.

***Holotype*** (MZB): ARC3270 (GenBank # MK260568), C-Sulawesi Prov., Luwuk, Salodi, Gn. Taluanjang, 00°49.202'S 122°52.395'E, 702 m, sifted, 21-V-2013. ***Paratypes*** (MZB, SMNK): Luwuk, Salodi, Gn. Taluanjang: 5 exx, ARC3271 (GenBank # MK260566), ARC3272 (GenBank # MK260565), ARC3273 (GenBank # MK260567), same data as holotype; 2 exx, 00°49.264'S 122°52.343'E, 759 m, sifted, 21-V-2013; 3 exx, 00°49.307'S 122°52.322'E, 760 m, sifted, 21-V-2013.

#### Distribution.

C-Sulawesi Prov. (Luwuk). Elevation 700–760 m.

#### Biology.

In leaf litter of lower montane forest.

#### Etymology.

This epithet is the Latin noun *silvicola* (sylvan, forest dweller) in apposition.

#### Notes.

*Trigonopterussilvicola* Riedel, sp. n. was coded as “*Trigonopterus* sp. 524”.

### 92. Trigonopterus squalidulus

Taxon classificationAnimaliaColeopteraCurculionidae

Riedel sp. n.

http://zoobank.org/09E96BD0-077B-4C03-BCA1-7DC60A25F499

#### Diagnostic description.

**Figure 92.**
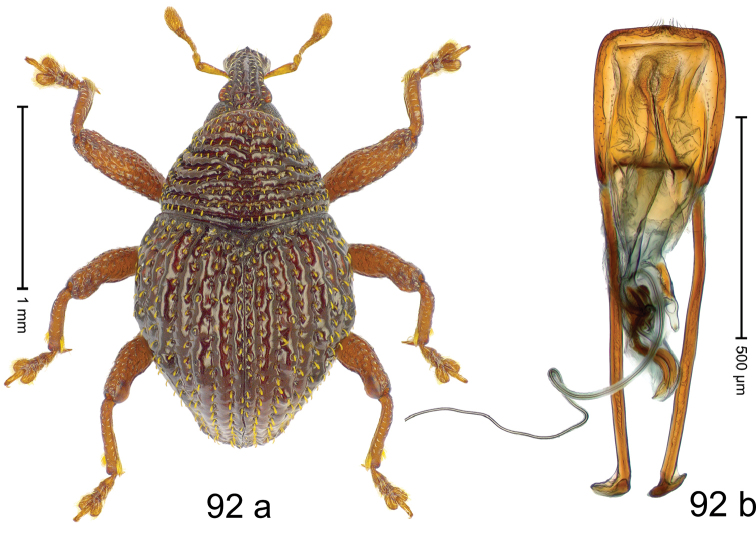
*Trigonopterussqualidulus* Riedel, sp. n., holotype; **a** habitus **b** penis.

***Holotype***, male (Fig. 92a). Length 1.94 mm. Color of antennae and legs ferruginous, remainder dark ferruginous. Body subovate; in dorsal aspect and in profile with distinct constriction between pronotum and elytron. Rostrum dorsally with median and pair of submedian ridges; intervening furrows with rows of coarse punctures and sparse setae; epistome short, posteriorly with transverse, subangulate ridge. Pronotum with weak subapical constriction; disk with transverse, crenulate costae separated by deep furrows; costae with rows of anteriad directed scales. Elytra with striae deeply impressed, with rows of spatulate suberect scales; intervals costate, subglabrous, with sparse punctures; intervals 1, 3, 5, 7 more distinctly swollen, weakly tuberculate. Metathoracic spiracle located on laterally projecting denticle. Femora with anterior surface punctate-rugose. Profemur edentate. Mesofemur and metafemur with small acute tooth; metafemur subapically with stridulatory patch. Mesotibia with enlarged, curved uncus. Abdominal ventrite 1 weakly concave, with coarse punctures; ventrite 5 at middle with shallow depression, coarsely punctate. Penis (Fig. 92b) with sides of body subparallel; apex subangulate, with sparse short setae; apodemes 2.3 × as long as body of penis; endophallus with pair of elongate sclerites; transfer apparatus complex, with asymmetrical S-shaped basal sclerite; ductus ejaculatorius with indistinct bulbus. ***Intraspecific variation***. Length 1.84–2.00 mm. Female rostrum slender, dorsally subglabrous, punctate; epistome indistinct. Female abdominal ventrite 5 flat, basally coarsely punctate.

#### Material examined.

***Holotype*** (MZB): ARC3071 (GenBank # MK260556), Tanah Toraja, Ponding, Baruppu, 02°48.339'S 119°47.880'E, 2113 m, sifted, 04-V-2013. ***Paratypes*** (ARC, MZB, SMNK): 11 exx, ARC3070 (PCR failed), ARC3072 (GenBank # MK260557), same data as holotype; 34 exx, Tanah Toraja, Ponding, Baruppu, 02°48.597'S 119°47.611'E, 1963 m, sifted, 03-V-2013; 1 ex, Tanah Toraja, Ponding - Pulu Pulu, 1800 m, sifted, 13–17-VIII-1990.

#### Distribution.

S-Sulawesi Prov. (Tanah Toraja). Elevation 1800–2100 m.

#### Biology.

In leaf litter of montane forest.

#### Etymology.

This epithet is the diminutive form of the Latin adjective *squalidus*, -*a*, -*um* (rough; dirty) and refers to the species´ small size and its rough integument often incrusted with dirt. A variable adjective as well.

#### Notes.

*Trigonopterussqualidulus* Riedel, sp. n. was coded as “*Trigonopterus* sp. 521”.

### 93. Trigonopterus sulawesiensis

Taxon classificationAnimaliaColeopteraCurculionidae

Riedel sp. n.

http://zoobank.org/5D510F98-56F8-42A5-B1F5-651E88F8F2B5

#### Diagnostic description.

**Figure 93.**
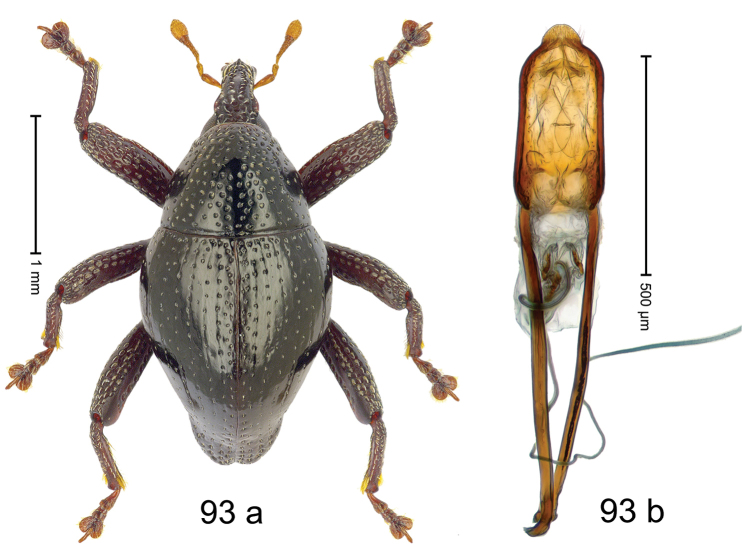
*Trigonopterussulawesiensis* Riedel, sp. n., holotype; **a** habitus **b** penis.

***Holotype***, male (Fig. 93a). Length 2.53 mm. Color of antennae ferruginous; legs dark ferruginous to black; remainder black. Body subovate; in dorsal aspect and in profile with weak constriction between pronotum and elytron. Rostrum dorsally with median and pair of submedian costae, anteriorly punctate; furrows with rows of suberect scales; epistome medially carinate, posteriorly with denticle. Pronotum with disk densely punctate; interspaces between punctures subglabrous, subequal to punctures´ diameter; median line impunctate. Elytra with striae marked by hairlines and rows of fine punctures; basal margin bordered by dense punctures; sutural interval punctate, other intervals subglabrous. Profemur with anteroventral ridge simple; meso- and metafemur with anteroventral ridge crenate, ending with denticle in apical 1/4; anterior surface of femora coarsely punctate, each puncture with narrow recumbent scale. Metafemur with dorsoposterior edge weakly denticulate; subapically with stridulatory patch. Metatibia ventrally with sparse row of long, stiff setae. Abdominal ventrites 1–2 concave, subglabrous; posteriorly sparsely punctate; ventrite 5 almost flat, with median shallow impression, dull, microreticulate, punctate. Penis (Fig. 93b) with sides subparallel in basal 1/2, converging in apical 1/3 to subacute, weakly rounded apex; apodemes 2.7 × as long as body of penis; transfer apparatus spiniform, directed basad in repose; ductus ejaculatorius with indistinct bulbus. ***Intraspecific variation***. Female. Length 2.50 mm. Rostrum in apical 1/2 dorsally subglabrous, with submedian and sublateral rows of punctures. Abdominal ventrite 5 flat, punctate.

#### Material examined.

***Holotype*** (MZB): ARC2913 (GenBank # MK260367), N-Sulawesi Prov., Kotamobagu, Modoinding, Kakenturan, 00°46.951'N 124°30.427'E to 00°47.053'N 124°30.413'E, 1210–1228 m, beaten, 19-V-2012. ***Paratype*** (SMNK): N-Sulawesi Prov.: 1 ex, ARC2914 (GenBank # MK260368), same data as holotype.

#### Distribution.

N-Sulawesi Prov. (Modoinding). Elevation ca. 1210–1230 m.

#### Biology.

On foliage in montane forests.

#### Etymology.

This epithet is a Latinized adjective based on the island of Sulawesi.

#### Notes.

*Trigonopterussulawesiensis* Riedel, sp. n. was coded as “*Trigonopterus* sp. 415”.

### 94. Trigonopterus suturatus

Taxon classificationAnimaliaColeopteraCurculionidae

Riedel sp. n.

http://zoobank.org/C86DBAF5-D075-4255-82BD-6F244DC28CB4

#### Diagnostic description.

**Figure 94.**
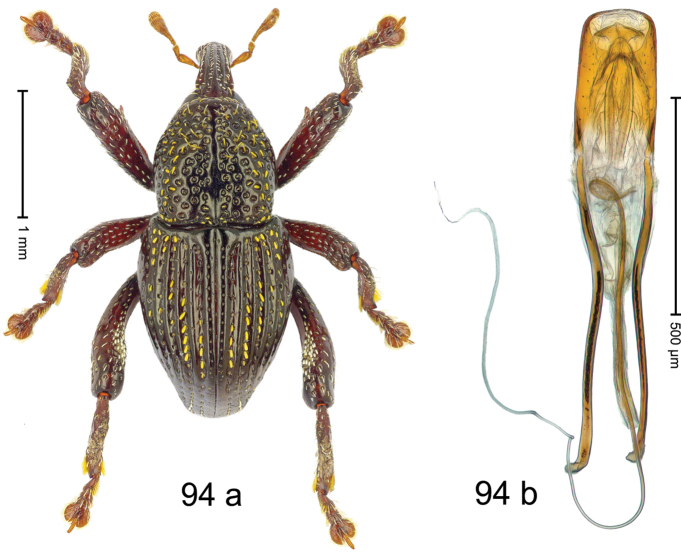
*Trigonopterussuturatus* Riedel, sp. n., holotype; **a** habitus **b** penis.

***Holotype***, male (Fig. 94a). Length 2.58 mm. Color of antennae light ferruginous; legs dark ferruginous; head and elytra dark ferruginous to black; remainder black. Body elongate; in dorsal aspect with constriction between pronotum and elytron; in profile dorsally flat. Rostrum dorsally with median carina and pair of distinct submedian ridges; intervening furrows with sparse, piliform scales; epistome indistinct. Pronotum elongate; disk densely punctate with coarse punctures, reticulate interspaces glabrous; with median costa, in basal 1/2 bordered by shallow submedian depressions; with sparse yellowish scales, anteriorly piliform and erect, sublaterally broader and recumbent. Elytra with striae 1–4 deeply entrenched, laterally with partly irregular punctures; dorsally intervals costate; sutural interval anteriorly markedly swollen, forming transverse costa to humeri; dorsally with sparse, almond-shaped, yellow, recumbent scales. Femora dentate, with acute tooth; anterior surface subglabrous, sparsely punctate, with few scattered recumbent piliform scales. Metafemur dorsally with sparse, almond-shaped, yellow scales; subapically with stridulatory patch. Tibia with dorsal edge subbasally denticulate. Abdominal ventrites 1–2 subglabrous, laterally with sparse coarse punctures; ventrite 5 flat, subglabrous, laterally with sparse coarse punctures. Penis (Fig. 94b) with sides of body subparallel, apex rounded, without setae; apodemes 2.6 × as long as body of penis; transfer apparatus flagelliform, 2.3 × longer than body of penis; ductus ejaculatorius without bulbus. ***Intraspecific variation***. Length 2.58–2.80 mm. Female rostrum slender.

#### Material examined.

***Holotype*** (MZB): ARC3211 (EMBL # LN884976), S-Sulawesi Prov., Mangkutana, 02°20.306'S 120°46.792'E, 909 m, sifted, 14-V-2013. ***Paratypes*** (MZB, SMNK): S-Sulawesi Prov.: 2 exx, ARC3212 (GenBank # MK260462), ARC3232 (GenBank # MK260463), same data as holotype; 1 ex, ARC3141 (GenBank # MK260461), Pendolo, Boe, 02°05.446'S 120°38.519'E, 915 m, 15-V-2013.

#### Distribution.

S-Sulawesi Prov. (Mangkutana), C-Sulawesi Prov. (Pendolo). Elevation ca. 910–915 m.

#### Biology.

In leaf litter of montane forest.

#### Etymology.

This epithet is a Latinized adjective based on the Latin noun *sutura* (seam) and suffix -*atus* (pertaining to) and refers to the swollen sutural interval of the elytra.

#### Notes.

*Trigonopterussuturatus* Riedel, sp. n. was coded as “*Trigonopterus* sp. 488”.

### 95. Trigonopterus tatorensis

Taxon classificationAnimaliaColeopteraCurculionidae

Riedel sp. n.

http://zoobank.org/C66AA391-4843-49C5-8E5A-30F9FB105E3D

#### Diagnostic description.

**Figure 95.**
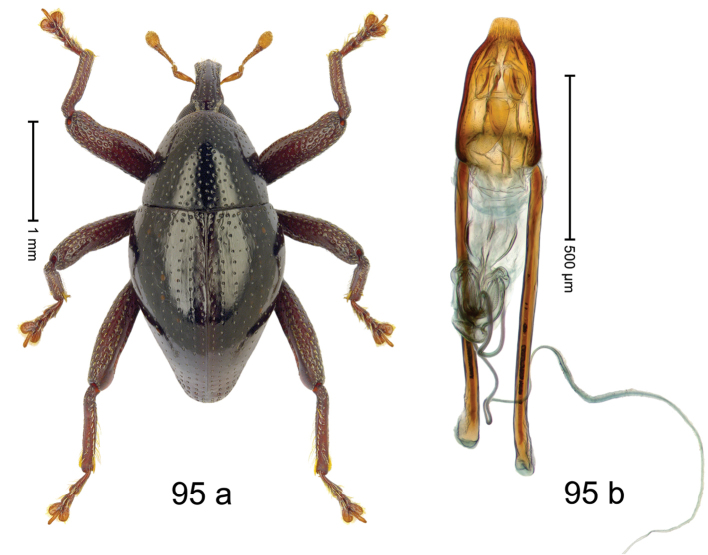
*Trigonopterustatorensis* Riedel, sp. n., holotype; **a** habitus **b** penis.

***Holotype***, male (Fig. 95a). Length 2.91 mm. Color of antennae ferruginous; legs dark ferruginous to black; remainder black. Body subovate; in dorsal aspect and in profile with indistinct constriction between pronotum and elytron. Rostrum dorsally with glabrous median costa, with rows of course punctures and furrows; with sparse suberect setae; epistome indistinct. Pronotum with disk densely punctate; interspaces between punctures subglabrous; above procoxa punctures large, almost confluent. Elytra with striae marked by hairlines and rows of fine punctures; basal margin bordered row of slightly larger punctures; sutural interval punctate, other intervals subglabrous. Profemur with anteroventral ridge indistinct, simple; meso- and metafemur with anteroventral ridge denticulate; anterior surface of femora coarsely punctate, each puncture with narrow recumbent scale. Metafemur with dorsoposterior edge simple, without denticles; subapically with stridulatory patch. Metatibia ventrally with sparse row of long, stiff setae. Abdominal ventrites 1–2 concave, sparsely punctate; ventrite 5 at middle with transverse depression, sparsely punctate, with sparse upcurved scales. Penis (Fig. 95b) with sides converging, apex spatulate, weakly rounded, without setae; ostium elongate; apodemes 2.7 × as long as body of penis; transfer apparatus small, hook-shaped, directed basad in repose, without supporting sclerites; ductus ejaculatorius with indistinct bulbus. ***Intraspecific variation***. Length 2.63–3.00 mm. Female rostrum subglabrous, with submedian and sublateral rows of punctures.

#### Material examined.

***Holotype*** (MZB): ARC2828 (EMBL # LN884935), S-Sulawesi Prov., Pc. Palopo, Gn. Sampuna, 02°56.539'S 120°05.320'E to 02°56.545'S 120°05.595'E, 1038–1101 m, beaten, 29-V-2012. ***Paratypes*** (MZB, SMNK, ZSM): S-Sulawesi Prov.: 228 exx, ARC2829 (GenBank # MK260374), ARC2830 (GenBank # MK260371), ARC2831 (GenBank # MK260370), same data as holotype; 78 exx, ARC6006 (GenBank # MK260373), Pc. Palopo, Gn. Sampuna, 02°56.539'S 120°05.320'E to 02°56.545'S 120°05.595'E, 1038–1101 m, beaten, 02-V-2013; 2 exx, Pc. Palopo, Gn. Sampuna, 02°56.539'S 120°05.320'E, 1038 m, sifted, 29-V-2012; 32 exx, Pc. Palopo, Gn. Sampuna, 800–1050 m, 15–16-IX-1997, beaten; 6 exx, ARC3002 (GenBank # MK260369), ARC3003 (GenBank # MK260375), ARC3004 (GenBank # MK260372), Tanah Toraja, Rantepao, Gn. Karre (= Gn. Wokim), 02°58.846'S 120°02.158'E, position 3, 1396 m, 06-V-2013.

#### Distribution.

S-Sulawesi Prov. (Pc. Palopo, Gn. Karre). Elevation 1100–1400 m.

#### Biology.

On foliage in montane forests.

#### Etymology.

This epithet is a Latinized adjective based on the Indonesian abbreviation of Tanah Toraja “TaTor” and refers to the type locality.

#### Notes.

*Trigonopterustatorensis* Riedel, sp. n. was coded as “*Trigonopterus* sp. 416”.

### 96. Trigonopterus tenuipes

Taxon classificationAnimaliaColeopteraCurculionidae

Riedel sp. n.

http://zoobank.org/D9FDD83C-FE50-44BE-B38D-181863860229

#### Diagnostic description.

**Figure 96.**
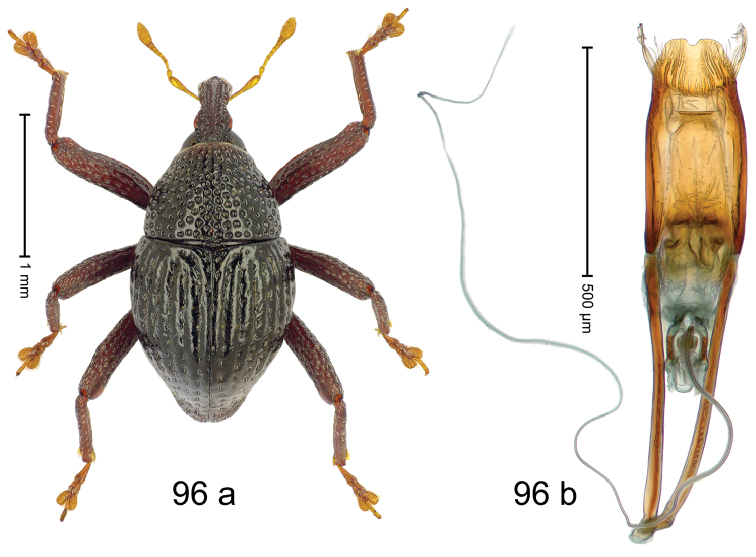
*Trigonopterustenuipes* Riedel, sp. n., holotype; **a** habitus **b** penis.

***Holotype***, male (Fig. 96a). Length 2.00 mm. Color of antennae yellowish; legs and rostrum dark ferruginous; remainder black. Body subovate; in dorsal aspect with weak constriction, in profile with distinct constriction between pronotum and elytron. Rostrum long; at basal 1/3 with constriction; dorsally with median costa and pair of submedian ridges; intervening furrows with rows of punctures containing each one indistinct seta; epistome indistinct, with median ridge. Pronotum with disk densely, coarsely punctate; interspaces between punctures reticulate, subglabrous. Elytra coriaceous, striae deeply impressed, dorsally intervals costate; sutural interval basally curving laterad; laterally striae marked by rows of coarse punctures. Legs long. Profemur with anteroventral ridge simple; meso- and metafemur with indistinct denticle in apical 1/3; anterior surface of femora coarsely punctate, reticulate, weakly microreticulate. Metafemur subapically with stridulatory patch. Abdominal ventrite 1 microreticulate, concave, at middle with coarse punctures; ventrite 5 microreticulate, with shallow concavity, laterally weakly rugose. Penis (Fig. 96b) with sides subparallel, in apical 1/3 weakly converging; subapically with lateral fringes of setae; subtruncate apex nude, with median notch; apodemes 1.3 × as long as body of penis; transfer apparatus short spiniform, directed basad in repose; ductus ejaculatorius with indistinct bulbus. ***Intraspecific variation***. Length 2.00–2.22 mm. Female rostrum with lateral and sublateral rows of punctures, medially with glabrous costa.

#### Material examined.

***Holotype*** (MZB): ARC2761 (GenBank # MK260323), N-Sulawesi Prov., Tomohon, Rurukan, Gn. Mahawu, 01°20.844'N 124°52.253'E to 01°20.751'N 124°52.129'E, 1209–1305 m, beaten, 15-V-2012. ***Paratypes*** (MZB, SMNK): N-Sulawesi Prov.: 3 exx, ARC2758 (GenBank # MK260325), ARC2759 (GenBank # MK260324), Tomohon, Rurukan, Gn. Mahawu, 01°21.409'N 124°51.535'E to 01°21.370'N 124°51.676'E, 1126–1231 m, beaten, 14-V-2012.

#### Distribution.

N-Sulawesi Prov. (Tomohon). Elevation ca. 1130–1210 m.

#### Biology.

On foliage in montane forests.

#### Etymology.

This epithet is a combination of the Latin adjective *tenuis*, -*e* (thin) and the noun *pes* (foot, leg). It refers to the species´ long, slender legs.

#### Notes.

*Trigonopterustenuipes* Riedel, sp. n. was coded as “*Trigonopterus* sp. 405”.

### 97. Trigonopterus tomohonensis

Taxon classificationAnimaliaColeopteraCurculionidae

Riedel sp. n.

http://zoobank.org/DE875D11-AFEC-4E49-BF92-18D134B6B4D6

#### Diagnostic description.

**Figure 97.**
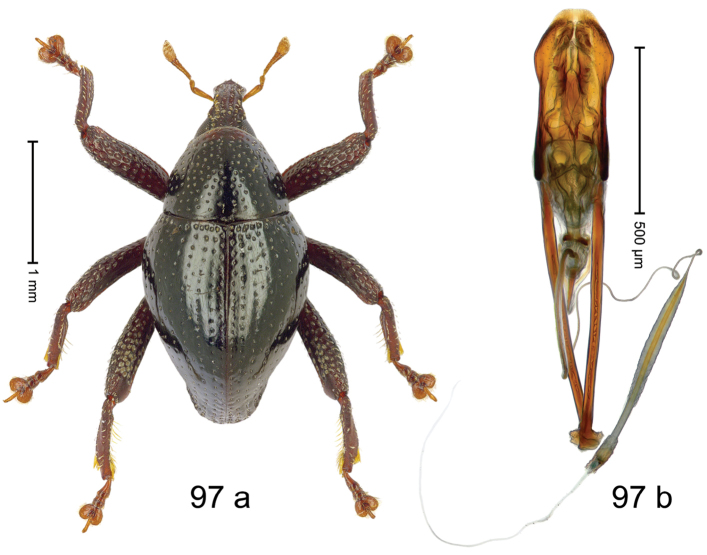
*Trigonopterustomohonensis* Riedel, sp. n., holotype; **a** habitus **b** penis.

***Holotype***, male (Fig. 97a). Length 2.40 mm. Color of antennae ferruginous; legs, rostrum, sutural interval of elytra dark ferruginous; remainder black. Body subovate; in dorsal aspect with weak constriction between pronotum and elytron; in profile dorsally convex. Rostrum dorsally with median and pair of submedian ridges; intervening furrows with rows of indistinct, suberect scales; epistome indistinct, with median denticle. Pronotum with disk densely punctate; interspaces between punctures subglabrous, subequal to punctures´ diameter; median line impunctate. Elytra with striae marked by dense rows of small punctures; basal margin bordered by transverse row of deeper punctures; striae 7–9 near base with large punctures; sutural interval with additional row, other intervals subglabrous. Profemur with anteroventral ridge simple; meso- and metafemur with anteroventral ridge crenate-denticulate, ending with indistinct denticle in apical 1/4; anterior surface of femora coarsely punctate, each puncture with scale. Metafemur with dorsoposterior edge denticulate; subapically with stridulatory patch. Meso- and metatibia ventrally with row of long, stiff setae. Abdominal ventrite 1 concave, subglabrous; ventrite 5 with shallow concavity, densely covered with small upcurved transparent scales. Penis (Fig. 97b) with sides subparallel in basal 1/2, weakly widening before converging in apical 1/3 to subtruncate apex; ostium elongate, narrow, confined to midline; apodemes 2.1 × as long as body of penis; transfer apparatus spiniform, directed basad in repose; ductus ejaculatorius with distinct bulbus. ***Intraspecific variation***. Length 2.40–2.54 mm. Female rostrum slender, dorsally subglabrous, sparsely punctate, epistome indistinct. Female abdominal ventrite 5 flat.

#### Material examined.

***Holotype*** (MZB): ARC2754 (GenBank # MK260321), N-Sulawesi Prov., Tomohon, Rurukan, Gn. Mahawu, 01°21.409'N 124°51.535'E to 01°21.370'N 124°51.676'E, 1126–1231 m, beaten, 14-V-2012. ***Paratypes*** (MZB, SMNK): N-Sulawesi Prov.: 1 ex, ARC2755 (GenBank # MK260316), same data as holotype; 2 exx, ARC2770 (GenBank # MK260313), ARC2772 (GenBank # MK260318), Tomohon, Rurukan, Gn. Mahawu, 01°21.409'N 124°51.535'E, 1126 m, sifted, 14-V-2012; 4 exx, ARC6054 (GenBank # MK260320), ARC6055 (GenBank # MK260322), Gn. Mahawu, Tomohon, 01°20.844'N 124°52.253'E,1209 m, sifted, 15-V-2012; 1 ex, ARC6053 (GenBank # MK260319), Gn. Mahawu, Tomohon, 01°20.791'N 124°52.268'E,1230 m, sifted, 14-V-2012; 8 exx, Gn. Mahawu, Tomohon, 01°20.819'N 124°52.194'E,1278 m, sifted, 15-V-2012; 4 exx, Gn. Mahawu, Tomohon, 01°20.751'N 124°52.129'E, 1305 m, sifted, 15-V-2012; 6 exx, Tomohon, Rurukan, Gn. Mahawu, 1200 m, 30-XI-1999, sifted; 1 ex, Tomohon, Rurukan, Gn. Mahawu, 1150–1200 m, 30-XI/03-XII-1999; 3 exx, Airmadidi, Gn. Klabat, 01°26.430'N 125°00.622'E, 889 m, sifted, 17-V-2012; 1 ex, Airmadidi, Gn. Klabat, 01°26.505'N 125°00.636'E, 923 m, sifted, 16-V-2012; 3 exx, Airmadidi, Gn. Klabat, 01°26.619'N 125°00.805'E, 1018 m, sifted, 17-V-2012; 10 exx, ARC2784 (GenBank # MK260315X), ARC2785 (GenBank # MK260314), ARC2786 (GenBank # MK260317), Airmadidi, Gn. Klabat, 01°26.635'N 125°00.823'E, 1031 m, sifted, 16-V-2012; 2 exx, Airmadidi, Gn. Klabat, 01°26.754'N 125°01.014'E, 1145 m, sifted, 16-V-2012.

#### Distribution.

N-Sulawesi Prov. (Tomohon, Mt Klabat). Elevation 890–1305 m.

#### Biology.

In leaf litter of montane forest.

#### Etymology.

This epithet is a Latinized adjective based on the town of Tomohon.

#### Notes.

*Trigonopterustomohonensis* Riedel, sp. n. was coded as “*Trigonopterus* sp. 403”.

### 98. Trigonopterus toraja

Taxon classificationAnimaliaColeopteraCurculionidae

Riedel sp. n.

http://zoobank.org/FEB62503-6D84-46B2-A69F-DD59ABF96195

#### Diagnostic description.

**Figure 98.**
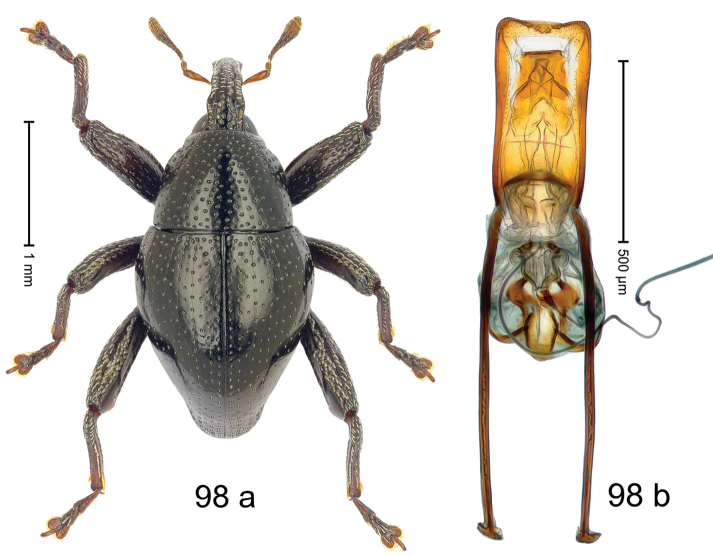
*Trigonopterustoraja* Riedel, sp. n., holotype; **a** habitus **b** penis.

***Holotype***, male (Fig. 98a). Length 2.55 mm. Color of antennae ferruginous, tarsi and tibiae dark ferruginous; remainder black. Body subovate; in profile dorsally convex. Rostrum in basal 1/2 dorsally with median and pair of submedian costae; intervening furrows with sparse rows of recumbent piliform scales; epistome indistinct; apical 1/3 subglabrous, sparsely setose. Pronotum with disk densely punctate with small punctures; interspaces between punctures subglabrous. Elytra irregularly punctate with small punctures, some striae marked by hardly visible hairlines; intervals flat; humerus with rows of coarser punctures. Meso- and metafemur with anteroventral ridge crenate, ending with blunt denticle in apical 1/3. Metafemur dorsally with recumbent, silvery scales; subapically with stridulatory patch. Metatibia ventrally with sparse row of thin setae. Abdominal ventrites 1–2 concave, subglabrous; in profile ventrite 2 subangularly projecting; ventrite 5 flat, punctate. Penis (Fig. 98b) with sides of body subparallel; apex subtruncate, with sparse setae; apodemes 2.0 × as long as body of penis; transfer apparatus spiniform, directed basad in repose, attached to anchor-shaped supporting sclerite; ductus ejaculatorius with indistinct bulbus. ***Intraspecific variation***. Length 2.38–2.63 mm. Color of legs from dark ferruginous to almost black. Female rostrum slender, subglabrous, with two submedian rows of punctures, with pair of sublateral furrows.

#### Material examined.

***Holotype*** (MZB): ARC3054 (GenBank # MK260495), S-Sulawesi Prov., Tanah Toraja, Bittuang, Gn. Karoa, 02°55.270'S 119°40.179'E to 02°55.032'S 119°40.365'E, 1800–2000 m, beaten, 10-V-2013. ***Paratypes*** (MZB, SMNK, ZSM): S-Sulawesi Prov., Tanah Toraja, Bittuang: 27 exx, ARC3053 (EMBL # LN884954), ARC5995 (GenBank # MK260494), ARC5996 (EMBL # MK260501), ARC5997 (EMBL # MK260500), ARC5998 (EMBL # MK260498), same data as holotype; 1 ex Tanah Toraja, Bittuang, Gn. Karoa, 02°55.270'S 119°40.179'E, 1836 m, sifted, 10-V-2013; 71 exx, ARC3033 (GenBank # MK260496), ARC3034 (GenBank # MK260497), ARC3035 (GenBank # MK260499), Gn. Ponding, 02°56.446'S 119°38.075'E, 1625 m, beaten, 09-V-2013.

#### Distribution.

S-Sulawesi Prov. (Tanah Toraja). Elevation 1625–1800 m.

#### Biology.

On foliage in montane forests.

#### Etymology.

The name “Toraja” resorts both to a people and the land inhabited by them. The epithet is a noun in apposition.

#### Notes.

*Trigonopterustoraja* Riedel, sp. n. was coded as “*Trigonopterus* sp. 498”. It is closely related to *T.rantepao* Riedel, sp. n. from which it can be distinguished by the structure of the penis.

### 99. Trigonopterus vicinus

Taxon classificationAnimaliaColeopteraCurculionidae

Riedel sp. n.

http://zoobank.org/7683ED0F-8BCE-40CE-8C63-6C5F5B7FFB44

#### Diagnostic description.

**Figure 99.**
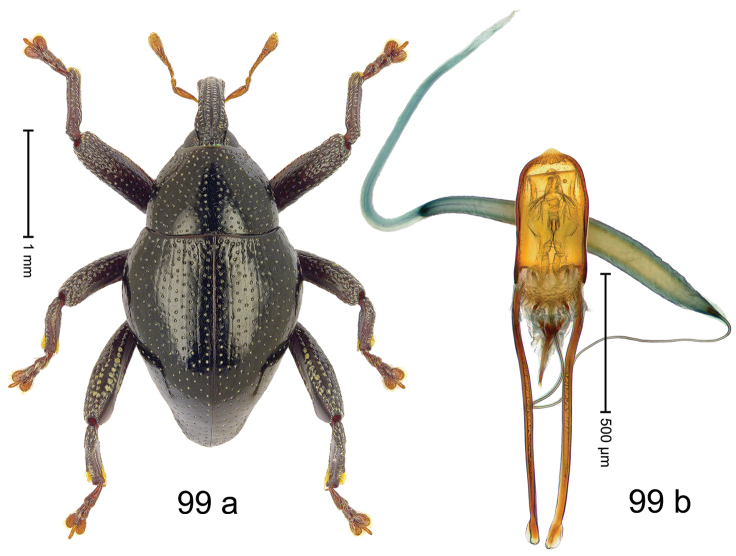
*Trigonopterusvicinus* Riedel, sp. n., holotype; **a** habitus **b** penis.

***Holotype***, male (Fig. 99a). Length 2.78 mm. Color of antennae and tarsi ferruginous; remainder black. Body subovate; in dorsal aspect with weak constriction between pronotum and elytron, in profile dorsally convex. Rostrum dorsally with median carina and pair of submedian ridges; intervening furrows with rows of yellowish piliform scales; in profile forming rounded hump at junction with forehead; epistome indistinct, subglabrous. Pronotum with disk densely punctate with small punctures; interspaces between punctures subglabrous; median line impunctate. Elytra densely irregularly punctate with small punctures; some striae marked by hardly visible hairlines; intervals flat; stria 8 along humerus with row of six large punctures. Femora edentate. Meso- and metafemur with anteroventral ridge crenate; anterior surface of femora densely punctate, each puncture with narrow recumbent scale. Metafemur dorsally with row of coarse punctures and sparse row of subrecumbent silvery scales; subapically with stridulatory patch. Metatibia with dorsal edge in basal 1/2 weakly crenate, in apical 1/3 narrowed, concave. Abdominal ventrite 1 and anterior portion of ventrite 2 concave, subglabrous; ventrite 5 at middle impressed, concave, sparsely punctate, sublaterally and subapically densely punctate with sparse suberect scales. Penis (Fig. 99b) with sides subparallel; apex subangular, weakly pointed, without setae; apodemes 2.0 × as long as body of penis; transfer apparatus spiniform, directed basad in repose; ductus ejaculatorius with distinct, swollen bulbus. ***Intraspecific variation***. Length 2.48–3.03 mm. Female body more slender. Female rostrum slender, from basal hump bent ventrad, almost straight; dorsally subglabrous, with two submedian rows of punctures, with pair of sublateral furrows. Female abdominal ventrite 5 flat, sparsely punctate.

#### Material examined.

***Holotype*** (MZB): ARC2845 (GenBank # MK260355), C-Sulawesi Prov., Pendolo, Gn. Sampuraga, 02°12.476'S 120°45.506'E, 1050 m, beaten, 31-V-2012. ***Paratypes*** (ARC, MZB, SMNK, ZSM): C-Sulawesi Prov.: 70 exx, ARC2843 (GenBank # MK260361), ARC2844 (GenBank # MK260360), ARC2846 (GenBank # MK260356), same data as holotype; 6 exx, Pendolo, Gn. Sampuraga, 02°12.165'S 120°45.567'E to 02°12.308'S 120°45.544'E, 934–1011 m, beaten, 13-V-2013; 39 exx, ARC2870 (GenBank # MK260357), ARC2871 (GenBank # MK260358), ARC2872 (GenBank # MK260359), Tentena, Tonusu, Bada-road, 01°47.316'S 120°30.623'E, 1078 m, beaten, 01-VI-2012; 5 exx, Tentena, Tonusu, Bada-road, 800–1000 m, beaten, 01-IX-1997; 10 exx, ARC3103 (GenBank # MK260354), ARC3104 (GenBank # MK260362), Pendolo, Boe, 02°05.405'S 120°38.551'E to 02°05.446'S 120°38.519'E, 750–950 m, beaten, 15-V-2013.

#### Distribution.

C-Sulawesi Prov. (Lake Poso). Elevation 950–1080 m.

#### Biology.

On foliage in montane forests.

#### Etymology.

This epithet is the Latin adjective *vicinus*, -*a*, -*um* (neighboring, similar) and refers to its sibling species found in close geographical distance.

#### Notes.

*Trigonopterusvicinus* Riedel, sp. n. was coded as “*Trigonopterus* sp. 412”. It is closely related to *T.ejaculatorius* Riedel, sp. n. from which it differs by 17.3% p-distance of *cox1* and can be distinguished by the less marked basal hump of the rostral profile.

### 100. Trigonopterus viduus

Taxon classificationAnimaliaColeopteraCurculionidae

Riedel sp. n.

http://zoobank.org/D0B73B0D-0079-4BD8-BE6D-E5E3ACF0B3B4

#### Diagnostic description.

**Figure 100.**
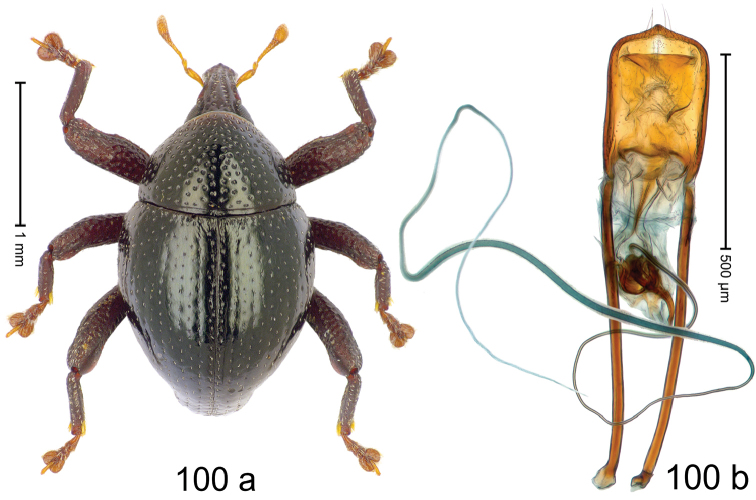
*Trigonopterusviduus* Riedel, sp. n., holotype; **a** habitus **b** penis.

***Holotype***, male (Fig. 100a). Length 2.18 mm. Color of antennae and tarsi ferruginous; legs dark ferruginous; remainder black. Body subovate; in profile dorsally convex. Rostrum dorsally with median and pair of submedian carinae widening posteriorly, extending as glabrous costae onto forehead; intervening furrows with sparse rows of recumbent setae; epistome at middle with denticle. Pronotum with disk densely punctate; interspaces between punctures subglabrous, subequal or smaller than punctures´ diameter; laterally punctures coarse. Elytra with striae marked by small punctures, weakly impressed; intervals subglabrous, with rows of minute punctures; stria 8 in basal 1/2 with larger punctures, externally bordered by costa. Femora with anteroventral ridge crenate, in mesofemur and metafemur ending with tooth; anterior surface coarsely punctate, microreticulate. Metafemur with dorsoposterior edge simple; subapically with stridulatory patch. Abdominal ventrites 1–2 concave, markedly microreticulate, weakly punctate; ventrite 5 at middle with shallow pit, markedly microreticulate, punctate, subapically with squamose scales. Penis (Fig. 100b) with sides of body subparallel; apex subangular, medially extended into small tooth; with few long setae; apodemes 2.3 × as long as body of penis; transfer apparatus S-shaped, basally held by capsule; ductus ejaculatorius with distinct bulbus. ***Intraspecific variation***. Length 1.78–2.28 mm. Female rostrum more slender, epistome indistinct. Female abdominal ventrite 5 flat.

#### Material examined.

***Holotype*** (MZB): ARC3135 (GenBank # MK260581), C-Sulawesi Prov., Pendolo, Boe, 02°05.405'S 120°38.551'E, 857 m, sifted, 15-V-2013. ***Paratypes*** (MZB, SMNK): C-Sulawesi Prov.: 9 exx, ARC6014 (GenBank # MK260579), ARC6015 (GenBank # MK260582), ARC6016 (GenBank # MK260580), same data as holotype; 6 exx, Pendolo, Boe, 02°05.440'S 120°38.537'E, 901 m, sifted, 15-V-2013; 4 exx, Pendolo, Boe, 02°05.446'S 120°38.519'E, 915 m, sifted, 15-V-2013.

#### Distribution.

C-Sulawesi Prov. (Pendolo). Elevation 860–915 m.

#### Biology.

In leaf litter of lower montane forest.

#### Etymology.

This epithet is the Latin adjective *viduus*, -*a*, -*um* (widowed).

#### Notes.

*Trigonopterusviduus* Riedel, sp. n. was coded as “*Trigonopterus* sp. 527”.

### 101. Trigonopterus volcanorum

Taxon classificationAnimaliaColeopteraCurculionidae

Riedel sp. n.

http://zoobank.org/CEECBD3C-1A0F-4F08-A7BD-89E65FA3B294

#### Diagnostic description.

**Figure 101.**
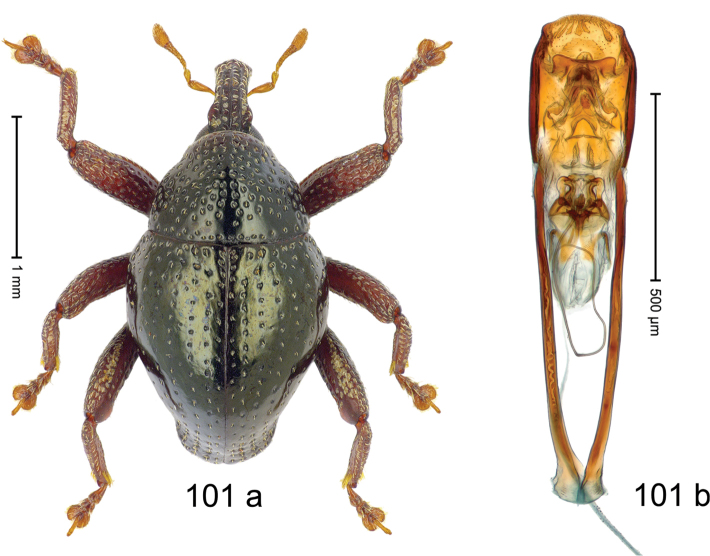
*Trigonopterusvolcanorum* Riedel, sp. n., holotype; **a** habitus **b** penis.

***Holotype***, male (Fig. 101a). Length 2.34 mm. Color of antennae and legs ferruginous; remainder black. Body subovate; in dorsal aspect with weak constriction between pronotum and elytron; in profile dorsally convex. Rostrum dorsally with median and pair of submedian ridges; intervening furrows with sparse rows of subrecumbent setae; epistome short, weakly swollen. Pronotum with disk densely punctate with coarse punctures except along impunctate median line; interspaces between punctures subglabrous, subequal to or smaller than punctures´ diameter; in subbasal 1/4 punctation sparse. Elytra with striae marked by small punctures; basal margin bordered by transverse row of deeper punctures; stria 8 along humerus with row of seven large punctures; sutural interval subbasally swollen, with shortened row of small punctures; intervals subglabrous. Femora edentate. Metafemur with dorsoposterior edge denticulate; subapically with stridulatory patch. Abdominal ventrite 1 cavernous, at middle subglabrous, lateral rims with sparse erect scales; ventrite 2 costate; ventrite 5 subglabrous, punctate, with shallow impression. Penis (Fig. 101b) with sides of body subparallel; apex weakly rounded, without setae; apodemes 2.2 × as long as body of penis; transfer apparatus complex; ductus ejaculatorius with indistinct bulbus. ***Intraspecific variation***. Length 2.20–2.34 mm. Female rostrum slender, in basal 1/4 with median and pair of submedian ridges; anteriorly subglabrous with submedian row of punctures and sublateral pair of furrows; epistome simple.

#### Material examined.

***Holotype*** (MZB): ARC2800 (GenBank # MK260273), N-Sulawesi Prov., Airmadidi, Gn. Klabat, 01°26.988'N 125°01.244'E, 1392 m, sifted, 17-V-2012. ***Paratypes*** (MZB, SMNK): N-Sulawesi Prov.: 1 ex, ARC2801 (GenBank # MK260274), same data as holotype; 1 ex, ARC2792 (GenBank # MK260272), Airmadidi, Gn. Klabat, 01°26.635'N 125°00.823'E, 1031 m, sifted, 16-V-2012; 1 ex, Airmadidi, Gn. Klabat, 01°26.754'N 125°01.014'E, 1145 m, sifted, 16-V-2012; 5 exx, Airmadidi, Gn. Klabat, 01°26.430'N 125°00.622'E, 889 m, sifted, 17-V-2012; 1 ex, ARC2757 (GenBank # MK260271), Tomohon, Rurukan, Gn. Mahawu, 01°21.409'N 124°51.535'E to 01°21.370'N 124°51.676'E, 1126–1231 m, 14-V-2012.

#### Distribution.

N-Sulawesi Prov. (Mt Klabat, Tomohon). Elevation 890–1400 m.

#### Biology.

In leaf litter of montane forest.

#### Etymology.

This epithet is a noun in genitive plural. It refers to the species´ occurrence on two active volcanoes.

#### Notes.

*Trigonopterusvolcanorum* Riedel, sp. n. was coded as “*Trigonopterus* sp. 392”.

### 102. Trigonopterus wangiwangiensis

Taxon classificationAnimaliaColeopteraCurculionidae

Riedel sp. n.

http://zoobank.org/BC999D09-8E00-450F-BE3A-57165F01B4DB

#### Diagnostic description.

**Figure 102.**
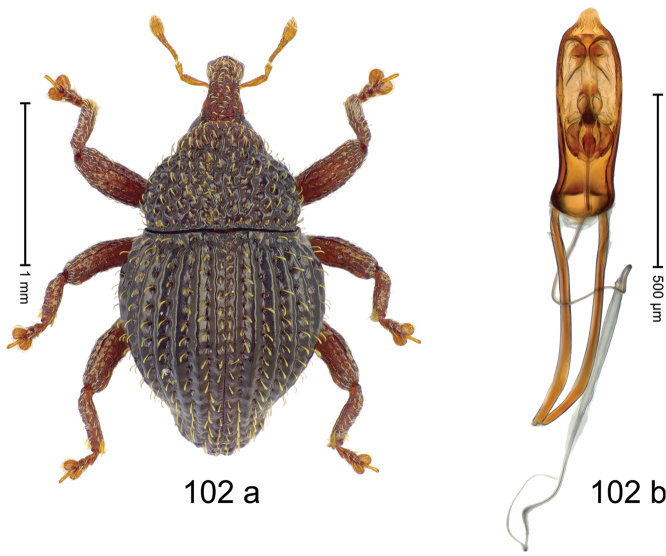
*Trigonopteruswangiwangiensis* Riedel, sp. n., holotype; **a** habitus **b** penis.

***Holotype***, male (Fig. 102a). Length 2.14 mm. Color of antennae ferruginous; legs and head dark ferruginous; remainder black. Body subovate; in dorsal aspect and in profile with distinct constriction between pronotum and elytron. Rostrum dorsally coarsely punctate-rugose, with distinct median ridge; with sparse rows of suberect slender scales; epistome short, posteriorly with transverse ridge. Pronotum with subapical constriction; disk coarsely punctate, scabrous; each puncture with erect, slender-clavate, curved scale. Elytra with striae deeply impressed; each puncture with erect, slender-clavate, curved scale; intervals carinate, subglabrous, weakly microreticulate. Meso- and metafemur with small denticle, profemur edentate. Metafemur subapically without stridulatory patch. Abdominal ventrite 5 flat, microreticulate, sparsely setose. Penis (Fig. 102b) hardly asymmetrical; tip medially weakly extended; basal orifice ventrally simple; apodemes subequal to body (1.1 ×); ductus ejaculatorius with distinct bulbus. ***Intraspecific variation***. Length 2.06–2.14 mm. Female rostrum slender, dorsally in apical 1/2 subglabrous, densely punctate-rugose; epistome simple.

#### Material examined.

***Holotype*** (MZB): ARC2808 (GenBank # MK260248), SE-Sulawesi Prov., Wakatobi Reg., Wangi Wangi Is, Matahora, 05°20.302'S 123°36.680'E, 32 m, 26-V-2012. ***Paratypes*** (MZB, SMNK): 14 exx, ARC2809 (EMBL # LT603142), ARC2810 (GenBank # MK260249), same data as holotype.

#### Distribution.

SE-Sulawesi Prov. (Wangi Wangi Is). Elevation ca. 30 m.

#### Biology.

In thin leaf litter of lowland forest.

#### Etymology.

This epithet is a Latinized adjective based on the type locality Wangi Wangi Island.

#### Notes.

*Trigonopteruswangiwangiensis* Riedel, sp. n. was coded as “*Trigonopterus* sp. 385”.

### 103. Trigonopterus watsoni

Taxon classificationAnimaliaColeopteraCurculionidae

Riedel sp. n.

http://zoobank.org/FAAC571B-2DAB-4A29-BAEA-80853A44DCFD

#### Diagnostic description.

**Figure 103.**
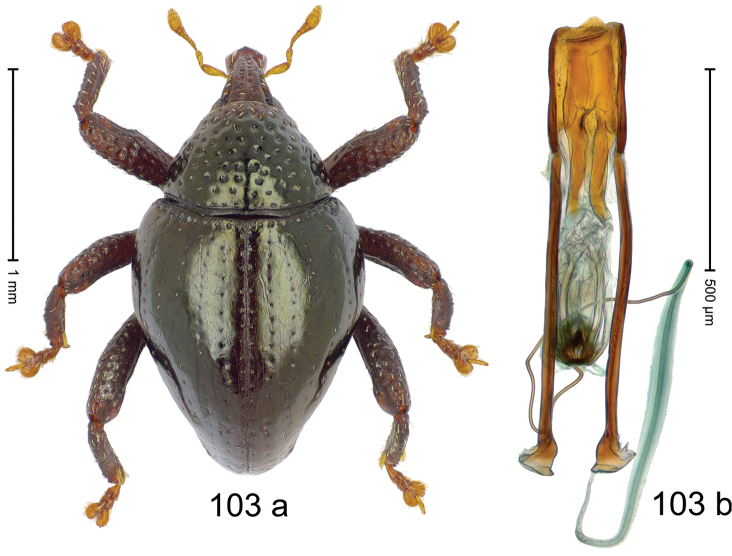
*Trigonopteruswatsoni* Riedel, sp. n., holotype; **a** habitus **b** penis.

***Holotype***, male (Fig. 103a). Length 1.90 mm. Color of antennae and tarsi ferruginous; legs and head dark ferruginous; remainder black, with slight bronze luster. Body subovate; in dorsal aspect with weak constriction between pronotum and elytron; in profile dorsally convex, with very weak constriction between pronotum and elytron. Rostrum dorsally with shortened median and pair of longer submedian ridges; intervening furrows with sparse rows of scales; epistome short, posteriorly with transverse, subangulate ridge. Pronotum with disk coarsely punctate; interspaces between punctures subglabrous. Elytra with striae marked by minute punctures; basal margin bordered by transverse row of deeper punctures; stria 8 in basal 1/2 with larger punctures, externally bordered by costa; intervals flat, subglabrous; apex ventrally subtruncate. Femora dentate. Anteroventral ridge of meso- and metafemur crenate, ending with blunt tooth. Metafemur dorsally denticulate, subapically with stridulatory patch. Abdominal ventrites 1–2 concave, subglabrous, microreticulate; ventrite 5 with sublateral ridges, at middle with impression basally bound by curved transverse ridge, densely punctate. Penis (Fig. 103b) with sides of body subparallel; apex somewhat asymmetrical, with subtriangular median extension; behind ostium with pair of elongate endophallic sclerites; apodemes 2.7 × as long as body of penis; transfer apparatus small, complex; ductus ejaculatorius with distinct bulbus. ***Intraspecific variation***. Length 1.86–1.90 mm. Female rostrum dorsally flattened; apical 1/2 punctate, basal 1/2 with ridges less distinct; epistome indistinct.

#### Material examined.

***Holotype*** (MZB): ARC3181 (EMBL # LN884970), SE-Sulawesi Prov., N Kolaka, Uluwolo village , 03°48.073'S 121°16.856'E, 182 m, sifted, 21-IV-2013. ***Paratype*** (SMNK): 1 ex, ARC3182 (GenBank # MK260445), same data as holotype.

#### Distribution.

SE-Sulawesi Prov. (Kolaka). Elevation ca. 180 m.

#### Biology.

In leaf litter of lowland forest.

#### Etymology.

This species is named in honor of Nobel laureate James D Watson for his contribution in the discovery of the structure of DNA.

#### Notes.

*Trigonopteruswatsoni* Riedel, sp. n. was coded as “*Trigonopterus* sp. 484”.

### 104. Trigonopterus yoda

Taxon classificationAnimaliaColeopteraCurculionidae

Riedel sp. n.

http://zoobank.org/8C98BED0-09DA-4140-BDF4-04A57E765E43

#### Diagnostic description.

**Figure 104.**
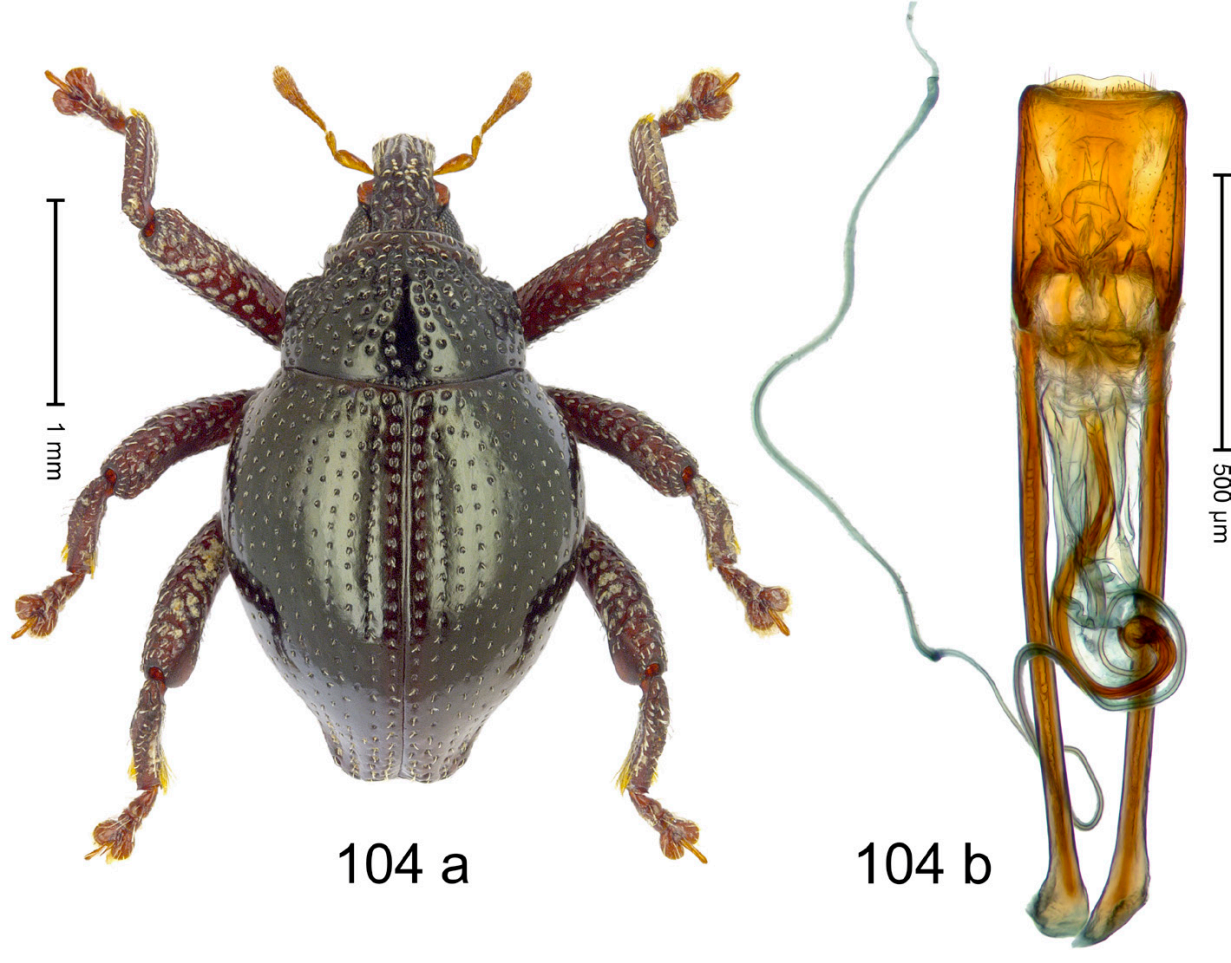
*Trigonopterusyoda* Riedel, sp. n., holotype; **a** habitus **b** penis.

***Holotype***, male (Fig. 104a). Length 2.71 mm. Color of antennae light ferruginous; legs and head dark ferruginous; remainder black with slight bluish luster. Body subovate; in dorsal aspect with distinct constriction between pronotum and elytron; in profile dorsally convex. Rostrum at base markedly bent ventrad; with lateral flanges in front of eyes; dorsally with distinct median carina and pair of submedian ridges; intervening furrows each with row of erect, clavate scales; epistome indistinct. Pronotum laterally with marked subapical constriction; except near base disk coarsely punctate; interspaces subglabrous, reticulate; with subglabrous median ridge. Elytra with striae marked by dense rows of small punctures and weak hairlines; intervals each with a similar row of punctures; interspaces subglabrous; subapically with sparse short setae; apex subtruncate. Femora edentate; anteroventral ridge distinct; anterior surface coarsely punctate, each puncture with subclavate scale. Metafemur dorsally denticulate; subapically with stridulatory patch. Metatibia with dorsal edge denticulate. Abdominal ventrites 1–2 concave, subglabrous, with long erect slightly curved setae; ventrite 5 flat, punctate, microreticulate. Penis (Fig. 104b) with sides of body subparallel; apex with median constriction, with sparse setae; apodemes 2.6 × as long as body of penis; transfer apparatus thick flagelliform, S-shaped, 1.3 × longer than body of penis; ductus ejaculatorius with indistinct bulbus. ***Intraspecific variation***. Length 2.28–2.73 mm. Female rostrum slender, dorsally with ridges less distinct; at base with few erect scales, anteriorly nude.

#### Material examined.

***Holotype*** (MZB): ARC3253 (GenBank # MK260584), C-Sulawesi Prov., Ampana, Bongka, Mire, 01°08.202'S 121°26.673'E, 414 m, sifted, 19-V-2013. ***Paratypes*** (MZB, SMNK): C-Sulawesi Prov., Ampana: 2 exx, ARC3254 (GenBank # MK260587), ARC3255 (GenBank # MK260586), same data as holotype; 19 exx, ARC3244 (GenBank # MK260588), ARC3245 (GenBank # MK260589), ARC3246 (GenBank # MK260585) Tanjung Api, 00°49.855'S 121°36.493'E, 166 m, sifted, 18-V-2013; 1 ex, Tanjung Api, 00°49.687'S 121°36.560'E, 130 m, sifted, 18-V-2013.

#### Distribution.

C-Sulawesi Prov. (Ampana). Elevation 170–410 m.

#### Biology.

In leaf litter of lowland forest.

#### Etymology.

This epithet is a noun in apposition based on the fictional character Yoda in George Lucas´ Star Wars movies. It appears to fit to a small greenish forest-dwelling creature.

#### Notes.

*Trigonopterusyoda* Riedel, sp. n. was coded as “*Trigonopterus* sp. 529”.

## Discussion

Our first study on Papuan *Trigonopterus* established the approach of accelerated taxonomy combining morphology and DNA barcoding by naming only selected representatives of a highly diverse fauna (Riedel et al. 2013a, b). The subsequent revisions of the Sunda Islands (Riedel et al. 2014) and Australia (Riedel and Tänzler 2016) treated all available species of a region. The present study is a compromise of both strategies. It is based on focused, yet limited field-work in Sulawesi. There are more than 30 additional species at hand, mostly represented by female specimens that should not be formally described unless additional material can be produced. *Trigonopterusfulvicornis* (Pascoe), the only previously described species from Sulawesi is not among our recent material although we searched near its type locality, Kendari. Vast areas of Sulawesi have never been sampled for small beetles and our current geographic sampling for *Trigonopterus* thus remains patchy. Many additional species are to be expected with the survey of additional localities in a group that is known for local endemism (Tänzler et al. 2012). Thus, the present work is only a first step and the species total of Sulawesi *Trigonopterus* is likely to double with additional surveys.

As a consequence, an identification key based on morphological characters is useless at this stage because a high proportion of species are still missing. Another complicating factor is the scarceness of distinct morphological characters that remain stable over major groups; instead characters abound that are diagnostic of species or small groups of species, but disappear among larger species groups. In this situation, BLAST searches for *cox1* sequences can give a much better indication if a species is contained in published datasets than a traditional key. Absolute values of sequence divergence warranting species status cannot be provided, nor is a definition of thresholds the subject of this paper. However, based on our experience, an uncorrected *cox1 p*-distance of less than ca. 5% is a first indication that two *Trigonopterus* specimens are conspecific and such a result may be seen as a preliminary identification, especially if genital and other morphological characters confirm this; hits with distances around 10% may point to separate, although related species, while distances exceeding 15% approach a saturation of sequence information and the phylogenetic relationship of a species as resolved by *cox1* becomes increasingly uncertain. Thus, the extent of sequence divergence gives clues on the further procedure, ranging from a final identification (after morphological comparison of two closely related specimens) to the sequencing of additional markers to resolve the phylogenetic position of a new, divergent lineage.

Sulawesi is located at the heart of Wallacea, a transient zone between the Oriental and Australian faunas and thus of great interest to biogeographers (Lohman et al. 2011). Some *Trigonopterus* species were already cited in the datasets of Tänzler et al. (2014, 2016) with manuscript species numbers. A more detailed study on the biogeography of Sulawesi *Trigonopterus* is in preparation and herein we provide names and faces to more than one hundred new species that will be part of the dataset. An approach providing valid names and high-resolution digital images is surely more sustainable than the extended use of morphospecies numbers without descriptions. Sometimes there is a prolonged lag between the publication of biogeographic (and other) research data and the taxonomic information that should provide their base. Too much perfectionism on the taxonomists´ side may raise doubts on their ability to provide their service to other fields of research. Names should become available as soon as possible and not be postponed for ever awaiting a better sample size.

There is also an urgent need for conservation measures and it is hoped that this is highlighted by the availability of species names. Most species of Sulawesi *Trigonopterus* are reported from the type locality only (79 species). A smaller number are from two or more localities with a linear distance of 12–30 km (15 species), or 50–90 km (8 species). Presumably, the average range of a *Trigonopterus* species in Sulawesi measures less than 100 km. The status of two populations of *T.ovatulus* Riedel, sp. n. separated by 160 km is uncertain and both may represent distinct species. There remain two notable species: 1) *T.allotopus* Riedel, previously described from Sumbawa Island, is herein recorded from SE Sulawesi near Kolaka and to date the only species of Sulawesi *Trigonopterus* also found on another island; this appears to be a case of relatively recent dispersal between two islands. 2) *T.inhonestus* Riedel, sp. n., spanning a linear distance of 780 km and ranging over at least three provinces of Sulawesi; this species is also exceptional in its capability of surviving in somewhat degraded habitats.

Thus, most of the *Trigonopterus* species recorded herein are endemic to the island of Sulawesi (with one exception) and most of them appear to occupy ranges of less than 100 km linear distance (three exceptions). With their high endemism, they are also highly endangered by habitat loss. Being flightless and ecologically tied to primary forest they are more vulnerable than many vertebrates or butterflies that may be able to colonize and re-colonize habitats of a wider distribution range. A flightless endemic weevil will vanish with its surrounding forest. Of Sulawesi´s natural forests only 30% are left in good condition, and these limited areas are now under increasing pressure, especially lowland forests (Cannon et. al. 2007). If we look at some of the areas where many of the newly described species have been discovered, the suitable habitats are much reduced and usually without effective protection. For example, at Mt Lompobattang, a hotspot of endemic arthropods, the natural forest below 1.700 m has been replaced by vegetable gardens. In densely populated Tanah Toraja natural forests are restricted to a few remaining areas: reforestation for timber usually leads to pine plantations that are unsuitable habitats for most of the native species. At Mt Mahawu near Tomohon natural forest has almost disappeared within the past 20 years. In fact, all these refugia may be already too small to be gazetted as National Parks or reserves with effective conservation measures, but still they contain (or used to contain) a unique and diverse fauna as exemplified by the newly described *Trigonopterus* weevils. A single forest fire during the dry season may seal the fate of many endemic species of Mt Klabat or Mt Lompobattang. The recent popularity of mountaineering increases the risk of camp fires getting out of control. Clearly, the majority of the largely endemic *Trigonopterus* species of Sulawesi cannot be preserved for the future unless the conservation of isolated remnants of natural forests can be greatly improved.

The *Trigonopterus* fauna of Sulawesi as currently known is composed of only few major phylogenetic lineages (figure S1 of Tänzler et al. 2016; unpublished data): 1) four species of the subgenusMimidotasia Voss; 2) two species of the *T.politus*-group; 3) three species of the *T.curtus*-group; 4) one species of the *T.honestus*-group; and 5) the remainder of all Sulawesi and Sunda species combined forming the “clade G” of Tänzler et al. (2016). This latter clade is speciose and morphologically heterogeneous. Its 180 described species comprise groups of both edaphic and foliage-frequenting lifestyles. They are herein subdivided into 28 separate species groups, owing to a lack of morphological characters that would allow the delineation of larger groups. Thus, based on morphological characters alone, the *Trigonopterus* fauna of Sulawesi appears more heterogeneous than it actually is. It is hoped that the present study encourages additional field work in the area. A higher sampling density would surely allow a better view on what may be one of the largest radiations on the island of Sulawesi.

### Provisional catalogue of species groups of *Trigonopterus* in Sulawesi

**subgenus Mimidotasia Voss**: *T.pauper* Riedel, sp. n., *T.luwukensis* Riedel, sp. n., *T.selayarensis* Riedel, sp. n., *T.wangiwangiensis* Riedel, sp. n.

***T.abnormis*-group**: *T.abnormis* Riedel, sp. n., *T.kolakensis* Riedel, sp. n.

***T.analis*-group**: *T.analis* Riedel, sp. n., *T.pagaranganensis* Riedel, sp. n., *T.pseudanalis* Riedel, sp. n.

***T.arachnobas*-group**: *T.arachnobas* Riedel, sp. n., *T.darwini* Riedel, sp. n., *T.gracilipes* Riedel, sp. n., *T.idefix* Riedel, sp. n., *T.pumilus* Riedel, sp. n., *T.rudis* Riedel, sp. n., *T.tenuipes* Riedel, sp. n.

***T.barbipes*-group**: *T.barbipes* Riedel, sp. n., *T.ejaculatorius* Riedel, sp. n., *T.vicinus* Riedel, sp. n., *T.viduus* Riedel, sp. n.

***T.bornensis*-group**: *T.rotundatus* Riedel, sp. n.

***T.collaris*-group**: *T.collaris* Riedel, sp. n., *T.klabatensis* Riedel, sp. n., *T.paracollaris* Riedel, sp. n.

***T.crenulatus*-group**: *T.costatulus* Riedel, sp. n., *T.crenulatus* Riedel, sp. n., *T.humilis* Riedel, sp. n., *T.squalidulus* Riedel, sp. n.

***T.curtus*-group**: *T.procurtus* Riedel, sp. n., *T.pseudosimulans* Riedel, sp. n., *T.scitulus* Riedel, sp. n.

***T.dimorphus*-group**: *T.reticulatus* Riedel, sp. n., *T.scabripes* Riedel, sp. n., *T.pendolensis* Riedel, sp. n.

***T.fulvicornis*-group**: *T.celebensis* Riedel, sp. n., *T.fulvicornis* (Pascoe, 1885), *T.pseudofulvicornis* Riedel sp. n., *T.seticnemis* Riedel, sp. n.

***T.honestus*-group**: *T.inhonestus* Riedel, sp. n.

***T.incendium*-group**: *T.incendium* Riedel, sp. n.

***T.impressicollis*-group**: *T.adspersus* Riedel sp. n., *T.castaneipennis* Riedel, sp. n., *T.impressicollis* Riedel, sp. n., *T.mesai* Riedel sp. n., *T.serripes* Riedel, sp. n., *T.suturatus* Riedel sp. n.

***T.laevigatus*-group**: *T.invalidus* Riedel, sp. n., *T.jasminae* Riedel, sp. n., *T.laevigatus* Riedel, sp. n.

***T.lampros*-group**: *T.artemis* Riedel, sp. n., *T.carinirostris* Riedel, sp. n., *T.curvipes* Riedel, sp. n., *T.lampros* Riedel, sp. n., *T.yoda* Riedel, sp. n.

***T.manadensis*-group**: *T.ambangensis* Riedel, sp. n., *T.armipes* Riedel, sp. n., *T.cirripes* Riedel, sp. n., *T.heberti* Riedel, sp. n., *T.mahawuensis* Riedel, sp. n., *T.manadensis* Riedel, sp. n., *T.modoindingensis* Riedel, sp. n., *T.pseudomanadensis* Riedel, sp. n., *T.volcanorum* Riedel, sp. n.

***T.minahassae*-group**: *T.indigenus* Riedel, sp. n., *T.minahassae* Riedel, sp. n., *T.prismae* Riedel, sp. n., *T.sampunensis* Riedel, sp. n., *T.silvicola* Riedel, sp. n.

***T.nanus*-group**: *T.hirsutus* Riedel, sp. n. *T.nanus* Riedel, sp. n.

***T.nitidulus*-group**: *T.cricki* Riedel, sp. n., *T.nitidulus* Riedel, sp. n.

***T.ovalipunctatus*-group**: *T.ovalipunctatus* Riedel, sp. n., *T.pseudovalipunctatus* Riedel, sp. n., *T.lompobattangensis* Riedel, sp. n.

***T.ovatulus*-group**: *T.ovatulus* Riedel, sp. n., *T.pseudovatulus* Riedel, sp. n.

***T.palopensis*-group**: *T.asterix* Riedel, sp. n., *T.fuscipes* Riedel, sp. n., *T.kotamobagensis* Riedel, sp. n., *T.latipennis* Riedel, sp. n., *T.matalibaruensis* Riedel, sp. n., *T.moatensis* Riedel, sp. n., *T.palopensis* Riedel, sp. n., *T.rhombiformis* Riedel, sp. n., *T.rufipes* Riedel, sp. n.

***T.politus*-group**: *T.allotopus* Riedel, *T.pseudallotopus* Riedel, sp. n.

***T.posoensis*-group**: *T.obelix* Riedel, sp. n., *T.posoensis* Riedel

***T.relictus*-group**: *T.mangkutanensis* Riedel, sp. n.

***T.rotundulus*-group**: *T.rotundulus* Riedel, sp. n., *T.watsoni* Riedel, sp. n.

***T.saltator*-group**: *T.bonthainensis* Riedel, sp. n.

***T.sampuragensis*-group**: *T.sampuragensis* Riedel, sp. n.

***T.tatorensis*-group**: *T.hypocrita* Riedel, sp. n., *T.incognitus* Riedel, sp. n., *T.tatorensis* Riedel, sp. n., *T.tomohonensis* Riedel, sp. n., *T.sulawesiensis* Riedel, sp. n.

***T.toraja*-group**: *T.ampanensis* Riedel, sp. n., *T.rantepao* Riedel, sp. n., *T.scaphiformis* Riedel, sp. n., *T.toraja* Riedel, sp. n.

## Plates
